# Supplementary material for: Relationships between multiple patient safety outcomes and healthcare and hospital-related risk factors in colorectal resection cases: cross-sectional evidence from a nationwide sample of 232 German hospitals
Source: BMJ Open. 2022 Jul 25;12(7):e058481. doi: 10.1136/bmjopen-2021-058481 (PMC9328106; doi:10.1136/bmjopen-2021-058481)
Supplement: Supplementary data [file bmjopen-2021-058481supp001.pdf]

S1: ICD-10 Codes of Elixhauser Comorbidity Groups [20]

Congestive heart failure: I099, I110, I130, I132, I255, I420, I425, I426, I427, I428, I429, I43, I50, P290

Cardiac arrhythmias: I441, I442, I443, I456, I459, I47, I48, I49, R000, R001, R008, T821, Z450, Z950

Valvular disease : A520, I05, I06, I07, I08, I091, I098, I34, I35, I36, I37, I38, I39, Q230, Q231, Q232, Q233, Z952, Z953 Z954

Pulmonary circulation Disorders: I26, I27, I280, I288, I289

Peripheral vascular disorders: I70, I71, I731, I738, I739, I771, I790, I792, K551, K558, K559, Z958, Z959

Hypertension, uncomplicated: I10

Hypertension, complicated: I11, I12, I13, I15

Paralysis: G041, G114, G801, G802, G81, G82, G830, G831, G832, G833, G834, G839

Other neurological disorders: G10, G11, G12, G13, G20, G21, G22, G254, G255, G312, G318, G319, G32, G35, G36, G37, G40, G41, G931, G934, R470, R56

Chronic pulmonary disease: I278, I279, J40, J41, J42, J43, J44, J45, J46, J47, J60, J61, J62, J63, J64, J65, J66, J67, J684, J701, J703

Diabetes, uncomplicated: E100, E101, E109, E110, E111, E119, E120, E121, E129, E130, E131, E139, E140, E141, E149

Diabetes, complicated: E102, E103, E104, E105, E106, E107, E108, E112, E113, E114, E115, E116, E117, E118, E122, E123, E124, E125, E126, E127, E128, E132, E133, E134, E135, E136, E137, E138, E142, E143, E144, E145, E146, E147, E148

Hypothyroidism: E00, E01, E02, E03, E890

Renal failure: I120, I131, N18, N19, N250, Z490, Z491, Z492, Z940, Z992

Peptic ulcer disease excluding bleeding: K257, K259, K267, K269, K277, K279, K287, K289

AIDS/HIV: B20, B21, B22, B24

Lymphoma: C81, C82, C83, C84, C85, C88, C96, C900, C902

Metastatic cancer: C77, C78, C79, C80

Solid tumor without metastasis: C00, C01, C02, C03, C04, C05, C06, C07, C08, C09, C1 0, C11, C12, C13, C14, C15, C16, C17, C18, C19, C20, C21, C22, C23, C24, C25, C26, C30, C31, C32, C33, C34, C37, C38, C39, C40, C41, C43, C45, C46, C47, C48, C49, C50, C51, C52, C53, C54, C55, C56, C57, C58, C60, C61, C62, C63, C64, C65, C66, C67, C68, C69, C70, C71, C72, C73, C74, C75, C76, C97

Rheumatoid arthritis/collagen vascular diseases: L940, L941, L943, M05, M06, M08, M120, M123, M30, M310, M311, M312, M313, M32, M33, M34, M35, M45, M461, M468, M469

Coagulopathy: D65, D66, D67, D68, D691, D693, D694, D695, D696

Obesity: E66

Weight loss: E40, E41, E42, E43, E44, E45, E46, R634, R64

Fluid and electrolyte disorders: E222, E86, E87

Blood loss anemia: D500

S2: OPS-codes for partial colon resections according to GIQI [21]

OPS-CODE	TRANSLATION (BY AUTHOR)
5-455.01	Partial resection of the colon: segmental resection: open surgery with anastomosis
5-455.02	Partial resection of the colon: segmental resection: open surgery with enterostoma and blind closure
5-455.03	Partial Resection of the Colon: Segment Resection: Open Surgery with Two Enterostomata
5-455.04	Partial Resection of the Colon: Segment Resection: Open Surgery with Anastomosis Anus praeter
5-455.05	Partial Resection of the Colon: Segmental Resection: Laparoscopic with Anastomosis
5-455.06	Partial Resection of the Colon: Segmental Resection: Laparoscopic with Enterostomy
5-455.07	Partial resection of the colon: segmental resection: change laparoscopic - open surgical
5-455.0X	Partial resection of the colon: Segment resection: Other
5-455.11	Partial resection of the colon: multiple segment resections: Open surgery with anastomosis
5-455.12	Partial resection of the colon: multiple segment resections: Open surgery with enterostoma and blind closure
5-455.13	Partial resection of the colon: multiple segment resections: Open surgery with two enterostomata
5-455.14	Partial resection of the colon: multiple segment resections: Open surgery with anastomosis anus praeter
5-455.15	Partial resection of the colon: multiple segment resections: Laparoscopic with anastomosis
5-455.16	Partial resection of the colon: multiple segment resections: Laparoscopic with enterostomy
5-455.17	Partial resection of the colon: multiple segment resections: Transfer laparoscopic - open surgery
5-455.1X	Partial resection of the colon: multiple segment resections: Other
5-455.21	Partial Resection of the Colon: Ileocecal Resection: Open Surgery with Anastomosis
5-455.22	Partial Resection of the Colon: Ileocecal Resection: Open surgery with enterostoma and blind closure
5-455.23	Partial Resection of the Colon: Ileocecal Resection: Open Surgery with Two Enterostomata
5-455.24	Partial Resection of the Colon: Ileocecal Resection: Open Surgery with Anastomosis Anus praeter
5-455.25	Partial Resection of the Colon: Ileocecal Resection: Laparoscopic with Anastomosis
5-455.26	Partial Resection of the Colon: Ileocecal Resection: Laparoscopic with Enterostoma
5-455.27	Partial resection of the colon: ileocecal resection: change laparoscopic - open surgical
5-455.2X	Partial resection of the colon: Ileocecal resection: Other
5-455.31	Partial resection of the colon: Caecal resection: Open surgery
5-455.35	Partial resection of the colon: Caecal resection: Laparoscopic
5-455.37	Partial resection of the colon: Caecal resection: change laparoscopic - open surgical
5-455.41	Partial resection of the colon: Resection of the ascending colon with coecum and right flexure [hemicolectomy right]: Open surgery with anastomosis
5-455.42	Partial Resection of the Colon: Resection of the ascending colon with coecum and right flexure [Hemicolectomy right]: Open surgery with enterostoma and
5-455.43	Partial Resection of the Colon: Resection of the ascending colon with coecum and right flexure [Hemicolectomy right]: Open surgery with two enterostomata
5-455.44	Partial resection of the colon: Resection of the ascending colon with coecum and right flexure [hemicolectomy right]: Open surgery with anastomosis anus praeter
5-455.45	Partial resection of the colon: Resection of the ascending colon with coecum and right flexure [hemicolectomy right]: Laparoscopic with anastomosis
5-455.46	Partial resection of the colon: Resection of the ascending colon with coecum and right flexure [hemicolectomy right]: Laparoscopically with enterostoma
5-455.47	Partial resection of the colon: resection of the ascending colon with coecum and right flexure [hemicolectomy right]: change laparoscopic - open surgical
5-455.4X	Partial resection of the colon: Resection of the ascending colon with coecum and right flexure [right hemicolectomy]: Other
5-455.51	Partial resection of the colon: Resection of the transverse colon: Open surgery with anastomosis
5-455.52	Partial resection of the colon: Resection of the transverse colon: open surgery with enterostoma and blind closure
5-455.53	Partial Resection of the Colon: Resection of the Transverse Colon: Open Surgery with Two Enterostomata
5-455.54	Partial resection of the colon: resection of the transverse colon: open surgery with anastomosis anus praeter
5-455.55	Partial resection of the colon: Resection of the transverse colon: Laparoscopic with anastomosis
5-455.56	Partial Resection of the Colon: Resection of the Transverse Colon: Laparoscopic with Enterostoma
5-455.57	Partial resection of the colon: resection of the transverse colon: change laparoscopic - open surgical
5-455.5X	Partial resection of the colon: Resection of the transverse colon: Other
5-455.61	Partial resection of the colon: resection of the descending colon with left flexure [hemicolectomy left]: open surgery with anastomosis
5-455.62	Partial resection of the colon: resection of the descending colon with left flexure [hemicolectomy left]: open surgery with enterostoma and blind closure
5-455.63	Partial resection of the colon: resection of the descending colon with left flexure [hemicolectomy left]: open surgery with two enterostomata
5-455.64	Partial resection of the colon: resection of the descending colon with left flexure [hemicolectomy left]: open surgery with anastomosis anus praeter
5-455.65	Partial resection of the colon: Resection of the descending colon with left flexure [Hemicolectomy left]: Laparoscopic with anastomosis
5-455.66	Partial Resection of the Colon: Resection of the descending colon with left flexure [Hemicolectomy left]: Laparoscopic with Enterostoma
5-455.67	Partial resection of the colon: resection of the descending colon with left flexure [hemicolectomy left]: change laparoscopic - open surgical
5-455.6X	Partial resection of the colon: Resection of the descending colon with left flexure [Hemicolectomy left]: Other
5-455.71	Partial resection of the colon: sigmoid resection: open surgery with anastomosis
5-455.72	Partial resection of the colon: sigmoid resection: open surgery with enterostoma and blind closure
5-455.73	Partial resection of the colon: sigmoid resection: open surgery with two enterostomata
5-455.74	Partial resection of the colon: sigmoid resection: open surgery with anastomosis anus praeter

5-455.75	Partial resection of the colon: sigmoid resection: laparoscopic with anastomosis
5-455.76	Partial resection of the colon: Sigmoid resection: laparoscopic with enterostoma
5-455.77	Partial resection of the colon: sigmoid resection: change laparoscopic - open surgical
5-455.7X	Partial resection of the colon: Sigmoid resection: Other
5-455.91	Partial resection of the colon: Resection of the ascending colon with coecum and right flexure and transverse colon [right hemicolectomy with transverse resection]:
5-455.92	Partial resection of the colon: Resection of the ascending colon with coecum and right flexure and transverse colon [right hemicolectomy with transverse resection]:
5-455.93	Partial resection of the colon: Resection of the ascending colon with coecum and right flexure and transverse colon [right hemicolectomy with transverse resection]:
5-455.94	Partial resection of the colon: Resection of the ascending colon with coecum and right flexure and transverse colon [right hemicolectomy with transverse resection]:
5-455.95	Partial resection of the colon: Resection of the ascending colon with coecum and right flexure and transverse colon [right hemicolectomy with transverse resection]:
5-455.96	Partial resection of the colon: Resection of the ascending colon with coecum and right flexure and transverse colon [right hemicolectomy with transverse resection]:
5-455.97	Partial resection of the colon: Resection of the ascending colon with coecum and right flexure and transverse colon [right hemicolectomy with transverse resection]:
5-455.9X	Partial resection of the colon: Resection of the ascending colon with coecum and right flexure and transverse colon [right hemicolectomy with transverse resection]:
5-455.A1	Partial resection of the colon: Resection of the descending colon with left flexure and transverse colon [left hemicolectomy with transverse resection]: Open
5-455.A2	Partial resection of the colon: Resection of the descending colon with left flexure and transverse colon [left hemicolectomy with transverse resection]: Open
5-455.A3	Partial resection of the colon: Resection of the descending colon with left flexure and transverse colon [left hemicolectomy with transverse resection]: Open
5-455.A4	Partial resection of the colon: Resection of the descending colon with left flexure and transverse colon [left hemicolectomy with transverse resection]: Open
5-455.A5	Partial Resection of the Colon: Resection of the descending colon with left flexure and transverse colon [Hemicolectomy left with transverse resection]: Laparoscopic
5-455.A6	Partial Resection of the Colon: Resection of the descending colon with left flexure and transverse colon [Hemicolectomy left with transverse resection]: Laparoscopic
5-455.A7	Partial resection of the colon: resection of the descending colon with left flexure and transverse colon [hemicolectomy left with transverse resection]: change
5-455.AX	Partial resection of the colon: Resection of the descending colon with left flexure and transverse colon [left hemicolectomy with transverse resection]: Other
5-455.B1	Partial resection of the colon: resection of the descending colon and sigmoid colon: open surgery with anastomosis
5-455.B2	Partial resection of the colon: resection of the descending colon and sigmoid colon: open surgery with enterostoma and blind closure
5-455.B3	Partial Resection of the Colon: Resection of the descending colon and sigmoid colon: Open surgery with two enterostomata
5-455.B4	Partial Resection of the Colon: Resection of the descending colon and sigmoid colon: Open surgery with anastomosis anus praeter
5-455.B5	Partial resection of the colon: resection of the descending colon and sigmoid colon: laparoscopic with anastomosis
5-455.B6	Partial resection of the colon: resection of the descending colon and sigmoid colon: laparoscopic with enterostoma
5-455.B7	Partial Resection of the Colon: Resection of the descending colon and sigmoid colon: transfer laparoscopically - open surgery
5-455.BX	Partial resection of the colon: Resection of the descending colon and sigmoid colon: Other
5-455.C1	Partial resection of the colon: resection of the ascending, transverse and descending colon with coecum and right and left flexure [hemicolectomy right and left
5-455.C2	Partial resection of the colon: resection of the ascending, transverse and descending colon with coecum and right and left flexure [hemicolectomy right and left
5-455.C3	Partial resection of the colon: resection of the ascending, transverse and descending colon with coecum and right and left flexure [hemicolectomy right and left
5-455.C4	Partial resection of the colon: resection of the ascending, transverse and descending colon with coecum and right and left flexure [hemicolectomy right and left
5-455.C5	Partial resection of the colon: resection of the ascending, transverse and descending colon with coecum and right and left flexure [hemicolectomy right and left
5-455.C6	Partial resection of the colon: resection of the ascending, transverse and descending colon with coecum and right and left flexure [hemicolectomy right and left
5-455.C7	Partial resection of the colon: resection of the ascending, transverse and descending colon with coecum and right and left flexure [hemicolectomy right and left
5-455.CX	Partial resection of the colon: Resection of the ascending, transverse and descending colon with coecum and right and left flexure [hemicolectomy right and left
5-455.D1	Partial resection of the colon: resection of the transverse colon, descending colon with left flexure and sigmoid colon [hemicolectomy left with
5-455.D2	Partial resection of the colon: resection of the transverse colon, descending colon with left flexure and sigmoid colon [hemicolectomy left with
5-455.D3	Partial resection of the colon: resection of the transverse colon, descending colon with left flexure and sigmoid colon [hemicolectomy left with
5-455.D4	Partial resection of the colon: resection of the transverse colon, descending colon with left flexure and sigmoid colon [hemicolectomy left with
5-455.D5	Partial resection of the colon: resection of the transverse colon, descending colon with left flexure and sigmoid colon [hemicolectomy left with
5-455.D6	Partial resection of the colon: resection of the transverse colon, descending colon with left flexure and sigmoid colon [hemicolectomy left with
5-455.D7	Partial resection of the colon: resection of the transverse colon, descending colon with left flexure and sigmoid colon [hemicolectomy left with
5-455.DX	Partial resection of the colon: resection of the transverse colon, descending colon with left flexure and sigmoid colon [left hemicolectomy with
5-455.X1	Partial resection of the colon: Other: Open surgery with anastomosis
5-455.X2	Partial resection of the colon: Other: Open surgery with enterostoma and blind closure
5-455.X3	Partial resection of the colon: Other: Open surgery with two enterostomata
5-455.X4	Partial resection of the colon: Other: Open surgery with anastomosis anus praeter
5-455.X5	Partial resection of the colon: Other: Laparoscopic with anastomosis
5-455.X6	Partial resection of the colon: Other: Laparoscopic with enterostoma
5-455.X7	Partial resection of the colon: Other: transfer laparoscopic - open surgical
5-455.XX	Partial resection of the colon: Other: Other
5-455.Y	Partial resection of the colon: N.n.ref.

S3: OPS-codes for total colon resections according to GIQI [21]

OPS-CODE	TRANSLATION (BY AUTHOR)
5-456.00	(Total) Colectomy and Proctocolectomy: Colectomy: Open surgery with ileostoma
5-456.01	(Total) Colectomy and Proctocolectomy: Colectomy: Open surgery with ileorectal anastomosis with reservoir (pouch)
5-456.02	(Total) Colectomy and Proctocolectomy: Colectomy: Open surgery with ileorectal anastomosis without reservoir (pouch)
5-456.03	(Total) Colectomy and Proctocolectomy: Colectomy: Open surgery with ileoanal anastomosis with reservoir (pouch)
5-456.04	(Total) Colectomy and Proctocolectomy: Colectomy: Open surgery with ileoanal anastomosis without reservoir (pouch)
5-456.05	(Total) Colectomy and Proctocolectomy: Colectomy: Laparoscopic with reservoir anastomosis (Pouch)
5-456.06	(Total) Colectomy and Proctocolectomy: Colectomy: Laparoscopic with Anastomosis without reservoir (Pouch)
5-456.07	(Total) Colectomy and Proctocolectomy: Colectomy: Laparoscopic with Ileostoma
5-456.08	(Total) Colectomy and Proctocolectomy: Colectomy: change laparoscopic - open surgical
5-456.0X	(Total) Colectomy and Proctocolectomy: Colectomy: Other
5-456.10	(Total) Colectomy and Proctocolectomy: Proctocolectomy: Open surgery with ileostoma
5-456.11	(Total) Colectomy and Proctocolectomy: Proctocolectomy: Open surgery with ileorectal anastomosis with reservoir (pouch)
5-456.12	(Total) Colectomy and Proctocolectomy: Proctocolectomy: Open surgery with ileorectal anastomosis without reservoir (pouch)
5-456.13	(Total) Colectomy and Proctocolectomy: Proctocolectomy: Open surgery with ileoanal anastomosis with reservoir (pouch)
5-456.14	(Total) Colectomy and Proctocolectomy: Proctocolectomy: Open surgery with ileoanal anastomosis without reservoir (pouch)
5-456.15	(Total) colectomy and proctocolectomy: Proctocolectomy: Laparoscopic with reservoir anastomosis (pouch)
5-456.16	(Total) Colectomy and Proctocolectomy: Proctocolectomy: Laparoscopic with anastomosis without reservoir (pouch)
5-456.17	(Total) colectomy and proctocolectomy: proctocolectomy: laparoscopic with ileostomy
5-456.18	(Total) colectomy and proctocolectomy: proctocolectomy: change laparoscopic - open surgical
5-456.1X	(Total) colectomy and proctocolectomy: Proctocolectomy: Other
5-456.20	(Total) colectomy and proctocolectomy: colectomy with proctomucosectomy: open surgery with ileostoma
5-456.21	(Total) colectomy and proctocolectomy: colectomy with proctomucosectomy: open surgery with ileorectal anastomosis with reservoir (pouch)
5-456.22	(Total) Colectomy and Proctocolectomy: Colectomy with Proctomucosectomy: Open surgery with ileorectal anastomosis without reservoir (pouch)
5-456.23	(Total) Colectomy and Proctocolectomy: Colectomy with proctomucosectomy: Open surgery with ileoanal anastomosis with reservoir (pouch)
5-456.24	(Total) colectomy and proctocolectomy: colectomy with proctomucosectomy: open surgery with ileoanal anastomosis without reservoir (pouch)
5-456.25	(Total) colectomy and proctocolectomy: colectomy with proctomucosectomy: laparoscopic with reservoir anastomosis (pouch)
5-456.26	(Total) colectomy and proctocolectomy: colectomy with proctomucosectomy: laparoscopic with anastomosis without reservoir (pouch)
5-456.27	(Total) colectomy and proctocolectomy: colectomy with proctomucosectomy: laparoscopic with ileostomy
5-456.28	(Total) colectomy and proctocolectomy: colectomy with proctomucosectomy: change laparoscopic - open surgical
5-456.2X	(total) colectomy and proctocolectomy: colectomy with proctomucosectomy: other
5-456.X0	(Total) colectomy and proctocolectomy: Other: Open surgery with ileostomy
5-456.X1	(Total) colectomy and proctocolectomy: Other: Open surgery with ileorectal anastomosis with reservoir (pouch)
5-456.X2	(total) colectomy and proctocolectomy: Other: Open surgery with ileorectal anastomosis without reservoir (pouch)
5-456.X3	(Total) colectomy and proctocolectomy: Other: Open surgery with ileoanal anastomosis with reservoir (pouch)
5-456.X4	(Total) colectomy and proctocolectomy: Other: Open surgery with ileoanal anastomosis without reservoir (pouch)
5-456.X5	(total) colectomy and proctocolectomy: Other: Laparoscopic with reservoir anastomosis (pouch)
5-456.X6	(Total) colectomy and proctocolectomy: Other: Laparoscopic with anastomosis without reservoir (pouch)
5-456.X7	(Total) colectomy and proctocolectomy: Other: Laparoscopic with ileostomy
5-456.X8	(total) colectomy and proctocolectomy: Other: change laparoscopic - open surgical
5-456.XX	(Total) colectomy and proctocolectomy: Other: Other
5-456.Y	(Total) colectomy and proctocolectomy: N.n.d.

S4: OPS-codes for rectum resections according to GIQI [21]

OPS-CODE	TRANSLATION (BY AUTHOR)
5-484.01	Rectum resection with sphincter preservation: Anterior cuff resection: Open surgery with anastomosis
5-484.02	Rectum resection with sphincter preservation: Anterior cuff resection: Open surgery with enterostoma and blind closure
5-484.05	Rectum resection with sphincter preservation: Anterior cuff resection: Laparoscopic with anastomosis
5-484.06	Rectum resection with sphincter preservation: Anterior cuff resection: Laparoscopic with enterostomy and blind closure
5-484.08	Rectum resection with sphincter preservation: Anterior cuff resection: change laparoscopic - open surgery with anastomosis
5-484.09	Rectum resection with sphincter preservation: Anterior cuff resection: change laparoscopic - open surgery with enterostoma and blind closure
5-484.0X	Rectum resection with sphincter preservation: Anterior cuff resection: Other
5-484.11	Rectum resection with sphincter preservation: Posterior rectotomy [Rectotomia posterior]: Open surgery with anastomosis
5-484.12	Rectum resection with sphincter preservation: Posterior rectotomy [Rectotomia posterior]: Open surgery with enterostoma and blind closure
5-484.15	Rectum resection with sphincter preservation: Posterior cuff resection [Rectotomia posterior]: Laparoscopic with anastomosis
5-484.16	Rectum resection with sphincter preservation: Posterior cuff resection [Rectotomia posterior]: Laparoscopic with enterostoma and blind closure
5-484.18	Rectum resection with sphincter preservation: posterior cuff resection [Rectotomia posterior]: change laparoscopic - open surgery with anastomosis
5-484.19	Rectum resection with sphincter preservation: posterior cuff resection [rectotomia posterior]: change laparoscopically - open surgically with enterostoma and
5-484.1X	Rectum resection with sphincter preservation: Posterior cuff resection [Rectotomia posterior]: Other
5-484.21	Rectum resection with preservation of sphincter: Tubular resection with paraproctica left intact: Open surgery with anastomosis
5-484.22	Rectum resection with preservation of sphincter: Tubular resection with paraproktium left in place: Open surgery with enterostoma and blind closure
5-484.25	Rectum resection with preservation of sphincter: Tubular resection with paraproctica left in place: Laparoscopic with anastomosis
5-484.26	Rectum resection with preservation of sphincter: Tubular resection with paraproktium: Laparoscopic with enterostoma and blind closure
5-484.27	Rectum resection with sphincter preservation: Tubular resection with paraprotic left in place: Peranal
5-484.28	Rectum resection with sphincter preservation: Tubular resection with paraproctium left in place: change laparoscopic - open surgery with anastomosis
5-484.29	Rectum resection with preservation of sphincter: Tubular resection with preservation of paraproktium: change laparoscopically - open surgically with enterostoma and
5-484.2X	Rectum resection with preservation of sphincter: Tubular resection with preservation of paraproktium: Other
5-484.31	Rectum resection with sphincter preservation: Anterior resection: Open surgery with anastomosis
5-484.32	Rectum resection with sphincter preservation: Anterior resection: open surgery with enterostoma and blind closure
5-484.35	Rectum resection with sphincter preservation: Anterior resection: Laparoscopic with anastomosis
5-484.36	Rectum resection with sphincter preservation: Anterior resection: Laparoscopic with enterostoma and blind closure
5-484.38	Rectum resection with sphincter preservation: Anterior resection: change laparoscopic - open surgical with anastomosis
5-484.39	Rectum resection with sphincter preservation: anterior resection: change laparoscopically - open surgically with enterostoma and blind closure
5-484.3X	Rectum resection with sphincter preservation: Anterior resection: Other
5-484.51	Rectum resection with sphincter preservation: deep anterior resection: open surgery with anastomosis
5-484.52	Rectum resection with sphincter preservation: deep anterior resection: open surgery with enterostoma and blind closure
5-484.55	Rectum resection with sphincter preservation: Deep anterior resection: Laparoscopic with anastomosis
5-484.56	Rectum resection with sphincter preservation: Deep anterior resection: Laparoscopic with enterostoma and blind closure
5-484.58	Rectum resection with sphincter preservation: deep anterior resection: change laparoscopic - open surgery with anastomosis
5-484.59	Rectum resection with sphincter preservation: deep anterior resection: change laparoscopic - open surgical with enterostoma and blind closure
5-484.5X	Rectum resection with sphincter preservation: Deep anterior resection: Other
5-484.61	Rectum resection with sphincter preservation: deep anterior resection with peranal anastomosis: open surgery with anastomosis
5-484.65	Rectum resection with sphincter preservation: deep anterior
5-484.68	Rectum resection with sphincter preservation: deep anterior resection with peranal anastomosis: change laparoscopic - open surgical with anastomosis
5-484.6X	Rectum resection with sphincter preservation: Deep anterior resection with peranal anastomosis: Other
5-484.X1	Rectum resection with sphincter preservation: Other: Open surgery with anastomosis
5-484.X2	Rectum resection with sphincter preservation: Other: Open surgery with enterostoma and blind closure
5-484.X5	Rectum resection with sphincter preservation: Other: Laparoscopic with anastomosis
5-484.X6	Rectum resection with sphincter preservation: Other: Laparoscopic with enterostoma and blind closure

5-484.X8	Rectum resection with sphincter preservation: Other: change laparoscopic - open surgery with anastomosis
5-484.X9	Rectum resection with sphincter preservation: Other: change laparoscopically - open surgically with enterostoma and blind closure
5-484.XX	Rectum resection with sphincter preservation: Other: Other
5-484.Y	Rectum resection with sphincter preservation: N.n.ref.
5-485.01	Rectum resection without preservation of sphincter: Abdominoperineal: Open surgery
5-485.02	Rectum resection without sphincter preservation: Abdominoperineal: Combined open surgical-laparoscopic
5-485.0X	Rectum resection without sphincter preservation: Abdominoperineal: Other
5-485.1	Rectum resection without preservation of sphincter: Abdominoperineal with removal of adjacent organs
5-485.21	Rectum resection without sphincter preservation: Sacroiliac abdomen: Open surgery
5-485.22	Rectum resection without sphincter preservation: Sacroiliac abdomen: Combined open surgical-laparoscopic
5-485.2X	Rectum resection without sphincter preservation: Sacroiliac abdomen: Other
5-485.3	Rectum resection without sphincter preservation: Sacral abdominosacral with removal of neighboring organs
5-485.4	Rectum resection without sphincter preservation: sacroperineal
5-485.5	Rectum resection without sphincter preservation: Perineal
5-485.X	Rectum resection without sphincter preservation: Other
5-485.Y	Rectum resection without sphincter preservation: N.n.ref.

S5: Definitions of patient safety outcomes

PATIENT SAFETY OUTCOME	DEFINITION ACCORDING TO ICD-10 GM
IN-HOSPITAL DEATH	Discharge destination: death (included in accounting data derived)
POST-OPERATIVE RESPIRATORY FAILURE [15]	ICD-10: J96 .0 - Respiratory failure, not elsewhere classified
POST-OPERATIVE WOUND INFECTION [15]	ICD-10: T81.4 - Infection following a procedure, not elsewhere classified<br>ICD-10: T82.6 - Infection and inflammatory reaction due to cardiac valve prosthesis<br>ICD-10: T82.7 - Infection and inflammatory reaction due to other cardiac and vascular devices, implants and grafts<br>ICD-10: T83.5 - Infection and inflammatory reaction due to prosthetic device, implant and graft in urinary system<br>ICD-10: T83.6 - Infection and inflammatory reaction due to prosthetic device, implant and graft in genital tract<br>ICD-10: T84.5 - Infection and inflammatory reaction due to internal joint prosthesis<br>ICD-10: T84.6 - Infection and inflammatory reaction due to internal fixation device [any site]<br>ICD-10: T84.7 - Infection and inflammatory reaction due to other internal orthopaedic prosthetic devices, implants and grafts<br>ICD-10: T87.4 - Infection of amputation stump
RENAL FAILURE [15]	ICD-10: N17.0 - Acute renal failure with tubular necrosis<br>ICD-10: N17.1 - Acute renal failure with acute cortical necrosis<br>ICD-10: N17.2 - Acute renal failure with medullary necrosis<br>ICD-10: N17.8 - Other acute renal failure<br>ICD-10: N17.9 - Acute renal failure, unspecified<br>ICD-10: N99.0- Postprocedural renal failure<br>Procedure code: 8-853.7- Continuous, venovenous, pump-driven (CVVH), anticoagulation with heparin or without anticoagulation

S6: Case-, care- and hospital related characteristics

	COLON RESECTIONS		RECTUM RESECTIONS	
**PATIENT SAFETY OUTCOMES**	n	% / Q1; Q3	n	% / Q1; Q3
Outcome in-hospital death				
no	48,914	(90.30 %)	19,525	(95.73 %)
yes	5,254	(9.69 %)	870	(4.26 %)
Outcome post-operative respiratory failure				
no	45,074	(83.21 %)	17,901	(87.77 %)
yes	9,094	(16.78 %)	2,494	(12.22 %)
Outcome post-operative wound infections				
no	48,013	(88.63 %)	18,109	(88.79 %)
yes	6,155	(11.36 %)	2,286	(11.2 %)
Outcome renal failure				
no	45,920	(84.77 %)	18,279	(89.62 %)
yes	8,248	(15.22 %)	2,116	(10.37 %)
**CASE CHARACTERISTICS**				
age (median)	68	(56; 77)	67	(57; 77)
elixhauser groups per case (median)	3	(2; 5)	3	(2; 5)
sex				
male	26,954	(49.76 %)	10,367	(50.83 %)
female	27,214	(50.23 %)	10,028	(49.16 %)
AIDS/HIV				
no	54,142	(99.95 %)	20,383	(99.94 %)
yes	26	(0.04 %)	12	(0.05 %)
alcohol abuse				
no	53,025	(97.88 %)	20,088	(98.49 %)
yes	1,143	(2.11 %)	307	(1.5 %)
blood loss anemia				
no	53,022	(97.88 %)	20,134	(98.72 %)
yes	1,146	(2.11 %)	261	(1.27 %)
cardiac arrhythmias				
no	43,241	(79.82 %)	17,135	(84.01 %)
yes	10,927	(20.17 %)	3,260	(15.98 %)
chronic pulmonary disease				
no	49,042	(90.53 %)	18,792	(92.14 %)
yes	5,126	(9.46 %)	1,603	(7.85 %)
coagulopathy				
no	44,935	(82.95 %)	17,598	(86.28 %)
yes	9,233	(17.04 %)	2,797	(13.71 %)
congestive heart failure				
no	47,447	(87.59 %)	18,608	(91.23 %)
yes	6,721	(12.4 %)	1,787	(8.76 %)
deficiency anemia				
no	51,887	(95.78 %)	19,852	(97.33 %)
yes	2,281	(4.21 %)	543	(2.66 %)
depression				
no	51,232	(94.57 %)	19,318	(94.71 %)
yes	2,936	(5.42 %)	1,077	(5.28 %)
diabetes, complicated				
no	51,887	(95.78 %)	19,754	(96.85 %)
yes	2,281	(4.21 %)	641	(3.14 %)
diabetes, uncomplicated				
no	46,491	(85.82 %)	17,664	(86.6 %)
yes	7,677	(14.17 %)	2,731	(13.39 %)
drug abuse				
no	53,911	(99.52 %)	20,321	(99.63 %)
yes	257	(0.47 %)	74	(0.36 %)
fluid and electrolyte disorders				
no	29,678	(54.78 %)	12,122	(59.43 %)
yes	24,490	(45.21 %)	8,273	(40.56 %)
hypertension, complicated				
no	51,632	(95.31 %)	19,680	(96.49 %)
yes	2,536	(4.68 %)	715	(3.51 %)
hypertension, uncomplicated				
no	28,369	(52.37 %)	10,692	(52.42 %)
yes	25,799	(47.62 %)	9,703	(47.57 %)

hypothyroidism				
no	47,235	(87.2 %)	18,008	(88.29 %)
yes	6,933	(12.79 %)	2,387	(11.7 %)
liver disease				
no	50,277	(92.81 %)	19,433	(95.28 %)
yes	3,891	(7.18 %)	962	(4.71 %)
lymphoma				
no	53,762	(99.25 %)	20,315	(99.6 %)
yes	406	(0.74 %)	80	(0.39 %)
metastatic cancer				
no	43,637	(80.55 %)	15,149	(74.27 %)
yes	10,531	(19.44 %)	5,246	(25.72 %)
obesity				
no	47,727	(88.11 %)	18,046	(88.48 %)
yes	6,441	(11.89 %)	2,349	(11.51 %)
other neurological disorders				
no	51,956	(95.91 %)	19,747	(96.82 %)
yes	2,212	(4.08 %)	648	(3.17 %)
paralysis				
no	52,765	(97.4 %)	19,989	(98.01 %)
yes	1,403	(2.59 %)	406	(1.99 %)
peptic ulcer disease excluding bleeding				
no	54,004	(99.69 %)	20,348	(99.76 %)
yes	164	(0.31 %)	47	(0.23 %)
peripheral vascular disorders				
no	49,190	(90.81 %)	18,228	(89.37 %)
yes	4,978	(9.18 %)	2,167	(10.62 %)
psychoses				
no	53,889	(99.48 %)	20,330	(99.68 %)
yes	279	(0.51 %)	65	(0.31 %)
pulmonary circulation disorders				
no	52,476	(96.87 %)	19,929	(97.71 %)
yes	1,692	(3.12 %)	466	(2.28 %)
renal Diseases				
no	45,739	(84.43 %)	17,896	(87.74 %)
yes	8,429	(15.56 %)	2,499	(12.25 %)
rheumatoid arthritis/ collagen vascular diseases				
no	53,262	(98.32 %)	20,115	(98.62 %)
yes	906	(1.67 %)	280	(1.37 %)
solid tumor without metastasis				
no	28,520	(52.65 %)	6,722	(32.95 %)
yes	25,648	(47.34 %)	13,673	(67.04 %)
valvular disease				
no	51,473	(95.02 %)	19,591	(96.05 %)
yes	2,695	(4.97 %)	804	(3.94 %)
weight loss				
no	46,134	(85.16 %)	17,442	(85.52 %)
yes	8,034	(14.83 %)	2,953	(14.47 %)
**HEALTHCARE CHARACTERISTICS**				
total colon resections				
no	51,310	(94.72 %)	20,173	(98.91 %)
yes	2,858	(5.27 %)	222	(1.08 %)
partial colon resections				
no	2,662	(4.91 %)	17,088	(83.78 %)
yes	51,506	(95.08 %)	3,307	(16.21 %)
rectum resections				
no	50,665	(93.53 %)	-	-
yes	3,503	(6.46 %)	20,395	(100.00 %)
colon and rectum resection				
no	50,665	(93.53 %)	16,892	(82.82 %)
yes	3,503	(6.46 %)	3,503	(17.17 %)
weekend surgery				
no	49,473	(91.33 %)	19,603	(96.11 %)
yes	4,695	(8.66 %)	792	(3.88 %)
admission				
referral	36,129	(66.69 %)	16,249	(79.67 %)
emergency case	16,116	(29.75 %)	3,744	(18.35 %)
transfer from other hospital	1,923	(3.55 %)	402	(1.97 %)

**HOSPITAL CHARACTERISTICS**				
annual volume				
colon resection cases (median)	72	(38; 119)	-	-
total colon resection (median)	1	(0;3)	-	-
rectum resections (median)	-	-	26	(11;42)
urbanization				
urban	124	(59.33 %)	119	(59.50 %)
rural	85	(40.66 %)	81	(40.50 %)
ownership				
public	82	(39.23 %)	80	(40.00 %)
non-profit	41	(19.61 %)	39	(19.50 %)
private	86	(41.14 %)	81	(40.50 %)
university hospital				
no	201	(96.17 %)	192	(96.00 %)
yes	8	(3.82 %)	8	(4.00 %)

S7: Bivariate analysis of case-, care- and hospital-related covariates of patient safety including all 54.168 colon resections in 209 hospitals

	IN-HOSPITAL DEATH		POST-OPERATIVE RESPIRATORY FAILURE		RENAL FAILURE		POST-OPERATIVE WOUND INFECTION	
**PATIENT COVARIATES**	OR	95% CI	OR	95% CI	OR	95% CI	OR	95% CI
sex								
male	Ref.		Ref.		Ref.		Ref.	
female	0.876***	(0.827 - 0.928)	0.828***	(0.790 - 0.868)	0.742***	(0.707 - 0.778)	0.931**	(0.881 - 0.983)
age	1.052***	(1.049 - 1.055)	1.030***	(1.028 - 1.031)	1.037***	(1.035 - 1.039)	1.008***	(1.006 - 1.009)
alcohol abuse	3.845***	(3.363 - 4.396)	3.862***	(3.400 - 4.387)	3.860***	(3.406 - 4.374)	1.945***	(1.669 - 2.266)
blood loss anemia	0.715**	(0.570 - 0.896)	1.355***	(1.159 - 1.585)	1.200*	(1.025 - 1.405)	1.387***	(1.159 - 1.659)
cardiac arrhythmias	4.338***	(4.084 - 4.608)	3.544***	(3.364 - 3.734)	3.995***	(3.791 - 4.209)	1.766***	(1.660 - 1.878)
chronic pulmonary disease	2.025***	(1.867 - 2.196)	2.120***	(1.978 - 2.273)	1.793***	(1.668 - 1.927)	1.554***	(1.431 - 1.689)
coagulopathy	9.015***	(8.439 - 9.630)	6.370***	(6.011 - 6.751)	7.207***	(6.798 - 7.640)	2.483***	(2.331 - 2.646)
congestive heart failure	4.645***	(4.342 - 4.969)	4.294***	(4.042 - 4.563)	4.382***	(4.127 - 4.652)	1.899***	(1.765 - 2.044)
deficiency anemia	1.013	(0.878 - 1.169)	1.448***	(1.302 - 1.610)	1.416***	(1.268 - 1.582)	1.250***	(1.101 - 1.418)
depression	0.888	(0.779 - 1.012)	1.827***	(1.669 - 2.000)	1.372***	(1.244 - 1.513)	1.923***	(1.741 - 2.125)
diabetes, complicated	2.766***	(2.481 - 3.084)	2.339***	(2.121 - 2.578)	2.946***	(2.678 - 3.240)	1.705***	(1.514 - 1.921)
diabetes, uncomplicated	1.485***	(1.378 - 1.600)	1.559***	(1.465 - 1.659)	1.594***	(1.497 - 1.698)	1.300***	(1.208 - 1.399)
fluid and electrolyte disorders	4.072***	(3.809 - 4.354)	4.463***	(4.229 - 4.709)	5.033***	(4.756 - 5.328)	2.650***	(2.497 - 2.812)
hypertension, complicated	2.124***	(1.896 - 2.380)	2.156***	(1.957 - 2.375)	2.446***	(2.225 - 2.688)	1.453***	(1.288 - 1.638)
hypertension, uncomplicated	0.961	(0.906 - 1.020)	1.266***	(1.207 - 1.329)	1.294***	(1.232 - 1.360)	1.198***	(1.132 - 1.267)
hypothyroidism	0.964	(0.883 - 1.052)	1.227***	(1.147 - 1.312)	1.176***	(1.096 - 1.261)	1.316***	(1.219 - 1.421)
liver disease	6.486***	(6.007 - 7.004)	2.844***	(2.635 - 3.069)	4.573***	(4.254 - 4.917)	1.345***	(1.223 - 1.478)
lymphoma	1.869***	(1.441 - 2.424)	1.319*	(1.030 - 1.690)	1.850***	(1.470 - 2.327)	1.246	(0.939 - 1.654)
metastatic cancer	0.948	(0.881 - 1.021)	1.004	(0.946 - 1.065)	0.827***	(0.777 - 0.881)	1.069	(1.000 - 1.143)
obesity	0.780***	(0.707 - 0.860)	1.173***	(1.092 - 1.259)	1.419***	(1.324 - 1.521)	1.656***	(1.533 - 1.789)
other neurological disorders	2.854***	(2.567 - 3.174)	3.417***	(3.109 - 3.754)	2.834***	(2.575 - 3.119)	1.860***	(1.660 - 2.084)
paralysis	2.709***	(2.373 - 3.092)	3.030***	(2.692 - 3.410)	2.435***	(2.156 - 2.751)	1.874***	(1.629 - 2.156)
peripheral vascular disorders	3.572***	(3.316 - 3.848)	2.745***	(2.562 - 2.941)	3.001***	(2.804 - 3.213)	1.741***	(1.606 - 1.889)
psychoses	1.004	(0.676 - 1.492)	2.578***	(1.978 - 3.361)	1.232	(0.903 - 1.679)	1.845***	(1.352 - 2.517)
pulmonary circulation disorders	3.533***	(3.150 - 3.964)	3.959***	(3.562 - 4.401)	3.185***	(2.863 - 3.543)	1.721***	(1.511 - 1.960)
renal failure	2.772***	(2.592 - 2.966)	2.261***	(2.132 - 2.398)	2.982***	(2.815 - 3.158)	1.524***	(1.419 - 1.636)
rheumatoid arthritis/collagen vascular diseases	1.393**	(1.141 - 1.702)	1.283**	(1.079 - 1.524)	1.373***	(1.156 - 1.630)	1.196	(0.980 - 1.461)
solid tumor without metastasis	0.660***	(0.622 - 0.701)	0.845***	(0.806 - 0.886)	0.760***	(0.724 - 0.798)	0.949	(0.898 - 1.002)
valvular disease	2.580***	(2.332 - 2.855)	2.355***	(2.151 - 2.579)	2.632***	(2.409 - 2.876)	1.426***	(1.275 - 1.594)
weight loss	1.439***	(1.328 - 1.560)	2.418***	(2.269 - 2.576)	2.091***	(1.963 - 2.228)	2.230***	(2.073 - 2.398)
**HEALTHCARE COVARIATES**								
admission								
referral	Ref.		Ref.		Ref.		Ref.	
emergency case	3.066***	(2.852 - 3.297)	2.263***	(2.133 - 2.401)	2.409***	(2.260 - 2.567)	1.461***	(1.368 - 1.559)
transfer from other hospital	5.722***	(5.105 - 6.412)	4.200***	(3.768 - 4.680)	4.308***	(3.870 - 4.795)	1.865***	(1.645 - 2.115)
weekend Surgery								
no	Ref.		Ref.		Ref.		Ref.	
yes	2.704***	(2.498 - 2.927)	2.148***	(1.999 - 2.308)	2.254***	(2.099 - 2.419)	1.382***	(1.265 - 1.509)

total colon resection								
no	Ref.		Ref.		Ref.		Ref.	
yes	3.687***	(3.351 - 4.057)	2.395***	(2.186 - 2.625)	2.954***	(2.704 - 3.227)	1.340***	(1.204 - 1.493)
colon and rectum resection								
no	Ref.		Ref.		Ref.		Ref.	
yes	1.114	(0.991 - 1.252)	1.563***	(1.429 - 1.710)	1.390***	(1.267 - 1.525)	1.757***	(1.593 - 1.937)
**HOSPITAL COVARIATES**								
case volume	1.071	(0.978 - 1.173)	0.934	(0.837 - 1.043)	1.044	(0.944 - 1.155)	1.265***	(1.117 - 1.432)
area								
urban	Ref.		Ref.		Ref.		Ref.	
rural	0.830*	(0.711 - 0.969)	0.849	(0.665 - 1.085)	0.760**	(0.624 - 0.927)	0.837	(0.661 - 1.059)
university hospital								
no	Ref.		Ref.		Ref.		Ref.	
yes	1.946***	(1.411 - 2.683)	1.003	(0.555 - 1.814)	1.767*	(1.113 - 2.806)	3.178***	(1.894 - 5.333)
ownership								
public	Ref.		Ref.		Ref.		Ref.	
non-profit	0.774*	(0.634 - 0.945)	0.915	(0.657 - 1.272)	0.691**	(0.534 - 0.895)	0.615**	(0.452 - 0.835)
private	0.748***	(0.636 - 0.880)	1.025	(0.785 - 1.338)	1.226	(0.999 - 1.504)	0.662**	(0.518 - 0.847)

Note: Elixhauser Groups drug abuse, peptic ulcer disease and AIDS/ HIV were not included into the regression due to low case numbers
*** p<0.001
** p <0.01
* p<0.05

S8: Bivariate analysis of case-, care- and hospital-related covariates of patient safety including all 20.395 rectum resections in 200 hospitals

-	**IN-HOSPITAL DEATH**		**POST-OPERATIVE RESPIRATORY FAILURE**		**RENAL FAILURE**		**POST-OPERATIVE WOUND INFECTION**	
**PATIENT COVARIATES**	OR	95% CI	OR	95% CI	OR	95% CI	OR	95% CI
sex								
male	Ref.		Ref.		Ref.		Ref.	
female	0.915	(0.796 - 1.050)	0.913*	(0.836 - 0.998)	0.787***	(0.717 - 0.864)	0.877**	(0.801 - 0.960)
age	1.075***	(1.067 - 1.082)	1.031***	(1.027 - 1.034)	1.039***	(1.035 - 1.043)	1.007***	(1.004 - 1.010)
alcohol abuse	2.767***	(1.915 - 3.997)	2.705***	(2.050 - 3.570)	2.531***	(1.916 - 3.345)	1.826***	(1.356 - 2.459)
blood loss anemia	1.984**	(1.270 - 3.100)	1.769**	(1.256 - 2.493)	2.637***	(1.942 - 3.581)	2.264***	(1.619 - 3.165)
cardiac arrhythmias	5.465***	(4.741 - 6.299)	3.457***	(3.124 - 3.825)	3.777***	(3.410 - 4.183)	1.564***	(1.399 - 1.748)
chronic pulmonary disease	2.213***	(1.820 - 2.690)	2.185***	(1.905 - 2.506)	1.835***	(1.587 - 2.122)	1.690***	(1.463 - 1.952)
coagulopathy	9.352***	(8.020 - 10.906)	5.781***	(5.171 - 6.463)	5.702***	(5.091 - 6.386)	2.680***	(2.401 - 2.993)
congestive heart failure	5.853***	(4.995 - 6.858)	4.275***	(3.781 - 4.834)	4.272***	(3.787 - 4.820)	1.793***	(1.560 - 2.060)
deficiency anemia	1.741**	(1.241 - 2.442)	2.119***	(1.696 - 2.648)	2.275***	(1.810 - 2.858)	1.893***	(1.499 - 2.391)
depression	0.872	(0.637 - 1.192)	1.675***	(1.409 - 1.991)	1.686***	(1.411 - 2.014)	1.701***	(1.436 - 2.014)
diabetes, complicated	3.196***	(2.455 - 4.160)	2.073***	(1.692 - 2.540)	3.187***	(2.635 - 3.854)	1.730***	(1.390 - 2.154)
diabetes, uncomplicated	1.653***	(1.388 - 1.969)	1.613***	(1.435 - 1.813)	1.660***	(1.472 - 1.873)	1.341***	(1.184 - 1.518)
fluid and electrolyte disorders	3.961***	(3.387 - 4.633)	4.043***	(3.660 - 4.467)	4.586***	(4.122 - 5.102)	2.563***	(2.324 - 2.826)
hypertension, complicated	2.656***	(2.033 - 3.470)	2.366***	(1.947 - 2.876)	2.681***	(2.225 - 3.230)	1.480***	(1.188 - 1.844)
hypertension, uncomplicated	1.094	(0.951 - 1.257)	1.358***	(1.241 - 1.486)	1.449***	(1.318 - 1.593)	1.159**	(1.057 - 1.271)
hypothyroidism	0.980	(0.790 - 1.215)	1.164*	(1.019 - 1.329)	1.170*	(1.019 - 1.344)	1.125	(0.981 - 1.290)
liver disease	5.887***	(4.864 - 7.124)	2.298***	(1.937 - 2.725)	3.606***	(3.084 - 4.216)	1.539***	(1.279 - 1.850)
lymphoma	2.350*	(1.112 - 4.967)	1.618	(0.882 - 2.967)	1.766	(0.972 - 3.208)	1.683	(0.946 - 2.992)
metastatic cancer	0.979	(0.835 - 1.148)	1.298***	(1.177 - 1.431)	0.978	(0.879 - 1.087)	1.187***	(1.075 - 1.311)
obesity	0.908	(0.726 - 1.135)	1.500***	(1.320 - 1.704)	1.632***	(1.435 - 1.855)	1.653***	(1.455 - 1.878)
other neurological disorders	3.942***	(3.107 - 5.001)	3.107***	(2.569 - 3.757)	2.277***	(1.860 - 2.788)	1.887***	(1.530 - 2.327)
paralysis	3.060***	(2.244 - 4.173)	2.958***	(2.336 - 3.745)	2.816***	(2.220 - 3.572)	1.849***	(1.425 - 2.399)
peripheral vascular disorders	2.521***	(2.118 - 3.000)	1.673***	(1.469 - 1.905)	1.924***	(1.690 - 2.189)	1.531***	(1.341 - 1.748)
psychoses	0.943	(0.291 - 3.056)	0.843	(0.371 - 1.914)	1.865	(0.969 - 3.592)	1.337	(0.660 - 2.707)
pulmonary circulation disorders	6.535***	(5.123 - 8.336)	4.514***	(3.663 - 5.563)	3.734***	(3.022 - 4.613)	2.041***	(1.619 - 2.573)
renal failure	2.984***	(2.532 - 3.518)	2.416***	(2.147 - 2.718)	3.393***	(3.031 - 3.797)	1.688***	(1.488 - 1.915)
rheumatoid arthritis/collagen vascular diseases	1.521	(0.932 - 2.482)	1.582**	(1.140 - 2.196)	1.471*	(1.043 - 2.076)	0.868	(0.580 - 1.301)
solid tumor without metastasis	0.571***	(0.494 - 0.659)	1.002	(0.906 - 1.107)	0.836***	(0.755 - 0.924)	1.090	(0.984 - 1.207)
valvular disease	3.682***	(2.935 - 4.619)	2.693***	(2.257 - 3.214)	2.803***	(2.351 - 3.341)	1.364**	(1.113 - 1.672)
weight loss	1.408***	(1.165 - 1.702)	2.522***	(2.244 - 2.834)	2.072***	(1.842 - 2.331)	2.079***	(1.843 - 2.346)
**HEALTHCARE COVARIATES**								
admission reason								
referral	Ref.		Ref.		Ref.		Ref.	
emergency case	4.038***	(3.450 - 4.727)	2.200***	(1.963 - 2.465)	2.272***	(2.013 - 2.563)	1.656***	(1.474 - 1.862)
transfer from other hospital	6.550***	(4.887 - 8.780)	3.944***	(3.088 - 5.038)	4.248***	(3.337 - 5.406)	2.208***	(1.705 - 2.861)
weekend surgery								
no	Ref.		Ref.		Ref.		Ref.	
yes	3.693***	(2.927 - 4.659)	2.215***	(1.831 - 2.678)	2.177***	(1.809 - 2.620)	1.281*	(1.030 - 1.593)
colon and rectum resection								

no	Ref.		Ref.		Ref.		Ref.	
yes	4.015***	(3.458 - 4.663)	3.095***	(2.781 - 3.444)	2.706***	(2.428 - 3.016)	2.014***	(1.805 - 2.247)
**HOSPITAL COVARIATES**								
case volume	0.743***	(0.661 - 0.835)	0.828**	(0.730 - 0.940)	0.830***	(0.746 - 0.924)	1.079	(0.946 - 1.232)
area								
urban	Ref.		Ref.		Ref.		Ref.	
rural	0.882	(0.680 - 1.143)	0.925	(0.684 - 1.251)	0.895	(0.709 - 1.129)	0.726*	(0.554 - 0.951)
university hospital								
no	Ref.		Ref.		Ref.		Ref.	
yes	1.439	(0.890 - 2.324)	0.857	(0.435 - 1.690)	1.134	(0.696 - 1.847)	3.037***	(1.778 - 5.186)
ownership								
public	Ref.		Ref.		Ref.		Ref.	
non-profit	0.691*	(0.500 - 0.954)	0.887	(0.594 - 1.324)	0.633**	(0.472 - 0.848)	0.605**	(0.428 - 0.854)
private	0.631***	(0.480 - 0.830)	1.070	(0.773 - 1.482)	1.245	(0.988 - 1.571)	0.673**	(0.508 - 0.893)

Note: Elixhauser Groups drug abuse, peptic ulcer disease and AIDS/ HIV were not included into the regression due to a low case numbers
*** p<0.001
** p <0.01
* p<0.05

S9: Multivariate analysis of case-, care- and hospital-related covariates of patient safety including all 54.168 colon resections in 209 hospitals

	**IN-HOSPITAL DEATH**		**POST-OPERATIVE RESPIRATORY FAILURE**		**RENAL FAILURE**		**POST-OPERATIVE WOUND INFECTION**	
	OR	95% CI	OR	95% CI	OR	95% CI	OR	95% CI
**CASE COVARIATES**								
sex								
male	Ref.		Ref		Ref		Ref	
female	0.937	(0.873 - 1.006)	0.788***	(0.745 - 0.833)	0.683***	(0.645 - 0.725)	0.882***	(0.832 - 0.936)
age	1.050***	(1.046 - 1.053)	1.014***	(1.012 - 1.017)	1.024***	(1.021 - 1.026)	0.998	(0.996 - 1.000)
alcohol abuse	1.484***	(1.250 - 1.761)	1.948***	(1.677 - 2.263)	1.574***	(1.352 - 1.833)	1.304**	(1.106 - 1.539)
blood loss anemia	0.472***	(0.365 - 0.609)	0.982	(0.823 - 1.172)	0.831*	(0.695 - 0.995)	1.154	(0.958 - 1.390)
cardiac arrhythmias	1.640***	(1.518 - 1.772)	1.585***	(1.487 - 1.690)	1.627***	(1.524 - 1.737)	1.183***	(1.100 - 1.271)
chronic pulmonary disease	1.362***	(1.234 - 1.503)	1.459***	(1.347 - 1.581)	1.113*	(1.022 - 1.212)	1.242***	(1.138 - 1.356)
coagulopathy	4.174***	(3.864 - 4.509)	3.117***	(2.920 - 3.327)	3.332***	(3.118 - 3.561)	1.644***	(1.531 - 1.764)
congestive heart failure	1.748***	(1.597 - 1.914)	1.902***	(1.759 - 2.056)	1.678***	(1.551 - 1.815)	1.218***	(1.114 - 1.332)
deficiency anemia	0.778**	(0.660 - 0.918)	1.058	(0.937 - 1.194)	1.096	(0.965 - 1.245)	1.024	(0.897 - 1.169)
depression	0.658***	(0.566 - 0.765)	1.425***	(1.286 - 1.580)	1.070	(0.956 - 1.197)	1.569***	(1.414 - 1.741)
diabetes, complicated	1.297***	(1.131 - 1.487)	1.181**	(1.051 - 1.328)	1.274***	(1.135 - 1.431)	1.191**	(1.045 - 1.358)
diabetes, uncomplicated	1.128*	(1.029 - 1.237)	1.201***	(1.117 - 1.291)	1.151***	(1.067 - 1.241)	1.113**	(1.028 - 1.204)
fluid and electrolyte disorders	1.884***	(1.743 - 2.038)	2.561***	(2.412 - 2.719)	2.882***	(2.704 - 3.072)	1.991***	(1.867 - 2.123)
hypertension, complicated	0.507***	(0.437 - 0.589)	0.735***	(0.649 - 0.832)	0.805***	(0.712 - 0.911)	0.928	(0.807 - 1.067)
hypertension, uncomplicated	0.551***	(0.511 - 0.595)	0.945	(0.890 - 1.003)	0.921*	(0.865 - 0.981)	1.045	(0.980 - 1.115)
hypothyroidism	0.795***	(0.716 - 0.884)	1.110**	(1.027 - 1.200)	1.046	(0.963 - 1.137)	1.206***	(1.112 - 1.309)
liver disease	4.411***	(3.999 - 4.865)	1.373***	(1.253 - 1.506)	2.588***	(2.368 - 2.829)	0.831***	(0.749 - 0.923)
lymphoma	0.983	(0.717 - 1.349)	0.810	(0.613 - 1.070)	1.111	(0.846 - 1.458)	1.029	(0.767 - 1.382)
metastatic cancer	1.524***	(1.376 - 1.687)	1.120**	(1.036 - 1.211)	0.984	(0.906 - 1.070)	1.058	(0.974 - 1.149)
obesity	0.766***	(0.682 - 0.862)	1.165***	(1.073 - 1.265)	1.498***	(1.379 - 1.627)	1.634***	(1.506 - 1.773)
other neurological disorders	1.504***	(1.319 - 1.714)	1.903***	(1.706 - 2.123)	1.515***	(1.352 - 1.699)	1.259***	(1.114 - 1.422)
paralysis	1.481***	(1.261 - 1.740)	1.468***	(1.281 - 1.682)	1.142	(0.989 - 1.318)	1.231**	(1.060 - 1.429)
peripheral vascular disorders	1.926***	(1.757 - 2.111)	1.493***	(1.378 - 1.618)	1.518***	(1.399 - 1.647)	1.239***	(1.136 - 1.351)
psychoses	0.707	(0.449 - 1.113)	2.052***	(1.516 - 2.778)	0.859	(0.600 - 1.229)	1.493*	(1.081 - 2.061)
pulmonary circulation disorders	1.593***	(1.380 - 1.838)	1.864***	(1.649 - 2.108)	1.336***	(1.174 - 1.519)	1.071	(0.933 - 1.231)
renal failure	1.308***	(1.200 - 1.425)	1.112**	(1.035 - 1.196)	1.505***	(1.401 - 1.616)	1.067	(0.984 - 1.156)
rheumatoid arthritis/collagen vascular diseases	1.001	(0.793 - 1.264)	0.971	(0.798 - 1.182)	0.982	(0.806 - 1.197)	1.056	(0.858 - 1.298)
solid tumor without metastasis	0.573***	(0.525 - 0.625)	0.764***	(0.715 - 0.816)	0.707***	(0.660 - 0.757)	0.907**	(0.845 - 0.975)
valvular disease	0.817**	(0.719 - 0.929)	0.862**	(0.772 - 0.961)	0.927	(0.831 - 1.033)	0.927	(0.820 - 1.048)
weight loss	0.891*	(0.809 - 0.980)	1.658***	(1.542 - 1.783)	1.426***	(1.324 - 1.535)	1.774***	(1.643 - 1.916)
**HEALTHCARE COVARIATES**								
admission reason								
referral	Ref.		Ref.		Ref.		Ref.	
emergency case	1.847***	(1.692 - 2.015)	1.413***	(1.320 - 1.513)	1.453***	(1.349 - 1.566)	1.145***	(1.067 - 1.228)
transfer from other hospital	2.528***	(2.193 - 2.915)	1.982***	(1.749 - 2.245)	1.908***	(1.678 - 2.171)	1.223**	(1.071 - 1.397)
weekend surgery								
no	Ref.		Ref.		Ref.		Ref.	
yes	1.669***	(1.515 - 1.839)	1.426***	(1.312 - 1.550)	1.480***	(1.360 - 1.610)	1.080	(0.984 - 1.186)

total colon resection								
no	Ref.		Ref.		Ref.		Ref.	
yes	2.679***	(2.369 - 3.029)	1.639***	(1.472 - 1.825)	2.228***	(1.999 - 2.483)	1.022	(0.913 - 1.143)
colon and rectum resection								
no	Ref.		Ref.		Ref.		Ref.	
yes	1.103	(0.960 - 1.267)	1.524***	(1.378 - 1.686)	1.408***	(1.265 - 1.567)	1.579***	(1.426 - 1.748)
**HOSPITAL COVARIATES**								
case volume	0.968	(0.871 - 1.076)	0.919	(0.807 - 1.047)	0.992	(0.891 - 1.106)	1.168*	(1.030 - 1.325)
area								
urban	Ref.		Ref.		Ref.		Ref.	
rural	1.061	(0.893 - 1.261)	0.863	(0.648 - 1.149)	0.772**	(0.635 - 0.939)	1.032	(0.824 - 1.292)
university hospital								
no	Ref.		Ref.		Ref.		Ref.	
yes	1.303	(0.888 - 1.912)	0.687	(0.338 - 1.397)	1.412	(0.889 - 2.241)	1.981*	(1.171 - 3.352)
ownership								
public	Ref.		Ref.		Ref.		Ref.	
non-profit	1.012	(0.811 - 1.262)	1.057	(0.726 - 1.540)	0.867	(0.670 - 1.122)	0.744*	(0.555 - 0.998)
private	1.244*	(1.026 - 1.507)	1.329	(0.972 - 1.817)	1.937***	(1.563 - 2.400)	0.777*	(0.608 - 0.992)

Note: Elixhauser Groups drug abuse, peptic ulcer disease and AIDS/ HIV were not included into the regression due to a low case numbers
*** $p<0.001$
** $p<0.01$
* $p<0.05$

S10: Multivariate analysis of case-, care- and hospital-related covariates of patient safety including all 20.395 rectum resections in 200 hospitals

	IN-HOSPITAL DEATH		POST-OPERATIVE RESPIRATORY FAILURE		RENAL FAILURE		POST-OPERATIVE WOUND INFECTION	
	OR	95% CI	OR	95% CI	OR	95% CI	OR	95% CI
**CASE COVARIATES**								
sex								
male	Ref.		Ref.		Ref.		Ref.	
female	0.842*	(0.710 - 0.998)	0.842**	(0.759 - 0.934)	0.685***	(0.613 - 0.765)	0.826***	(0.748 - 0.912)
age	1.068***	(1.059 - 1.078)	1.014***	(1.009 - 1.018)	1.021***	(1.016 - 1.026)	0.998	(0.995 - 1.002)
alcohol abuse	1.410	(0.891 - 2.231)	1.656**	(1.200 - 2.286)	1.207	(0.869 - 1.676)	1.143	(0.828 - 1.578)
blood loss anemia	0.829	(0.486 - 1.413)	0.890	(0.607 - 1.305)	1.383	(0.976 - 1.959)	1.579*	(1.113 - 2.241)
cardiac arrhythmias	1.832***	(1.535 - 2.188)	1.662***	(1.471 - 1.878)	1.639***	(1.448 - 1.854)	1.034	(0.909 - 1.176)
chronic pulmonary disease	1.303*	(1.030 - 1.648)	1.510***	(1.291 - 1.766)	1.156	(0.979 - 1.366)	1.369***	(1.175 - 1.594)
coagulopathy	4.309***	(3.600 - 5.158)	3.052***	(2.697 - 3.455)	2.886***	(2.541 - 3.277)	1.770***	(1.570 - 1.996)
congestive heart failure	1.825***	(1.481 - 2.248)	1.849***	(1.583 - 2.159)	1.536***	(1.317 - 1.792)	1.099	(0.929 - 1.300)
deficiency anemia	0.880	(0.593 - 1.304)	1.242	(0.967 - 1.595)	1.357*	(1.052 - 1.750)	1.332*	(1.043 - 1.700)
depression	0.629*	(0.439 - 0.900)	1.222*	(1.005 - 1.486)	1.290*	(1.056 - 1.577)	1.386***	(1.161 - 1.654)
diabetes, complicated	1.584**	(1.145 - 2.192)	1.045	(0.824 - 1.326)	1.441**	(1.153 - 1.801)	1.172	(0.922 - 1.490)
diabetes, uncomplicated	1.124	(0.911 - 1.388)	1.194*	(1.043 - 1.368)	1.191*	(1.036 - 1.369)	1.164*	(1.018 - 1.331)
fluid and electrolyte disorders	1.704***	(1.425 - 2.039)	2.432***	(2.181 - 2.712)	2.855***	(2.542 - 3.206)	1.952***	(1.759 - 2.166)
hypertension, complicated	0.461***	(0.324 - 0.656)	0.757*	(0.591 - 0.970)	0.836	(0.659 - 1.060)	0.911	(0.704 - 1.178)
hypertension, uncomplicated	0.584***	(0.490 - 0.695)	0.980	(0.878 - 1.093)	0.999	(0.890 - 1.121)	0.993	(0.893 - 1.104)
hypothyroidism	0.812	(0.632 - 1.043)	0.993	(0.854 - 1.155)	1.002	(0.856 - 1.174)	1.035	(0.895 - 1.198)
liver disease	4.027***	(3.155 - 5.139)	1.268*	(1.036 - 1.553)	2.312***	(1.920 - 2.783)	1.036	(0.848 - 1.267)
lymphoma	1.308	(0.563 - 3.042)	1.028	(0.532 - 1.986)	0.930	(0.476 - 1.814)	1.320	(0.718 - 2.427)
metastatic cancer	1.376**	(1.118 - 1.694)	1.211**	(1.073 - 1.367)	0.983	(0.863 - 1.121)	1.051	(0.937 - 1.179)
obesity	0.922	(0.708 - 1.202)	1.501***	(1.296 - 1.738)	1.563***	(1.348 - 1.812)	1.638***	(1.431 - 1.876)
other neurological disorders	2.306***	(1.722 - 3.089)	1.879***	(1.507 - 2.343)	1.184	(0.936 - 1.498)	1.376**	(1.098 - 1.725)
paralysis	1.153	(0.785 - 1.695)	1.391*	(1.056 - 1.833)	1.320	(0.999 - 1.744)	1.110	(0.839 - 1.469)
peripheral vascular disorders	1.908***	(1.548 - 2.353)	1.253**	(1.083 - 1.450)	1.369***	(1.181 - 1.587)	1.283***	(1.117 - 1.475)
psychoses	0.867	(0.205 - 3.672)	0.618	(0.246 - 1.551)	2.075	(0.986 - 4.369)	1.072	(0.507 - 2.264)
pulmonary circulation disorders	2.816***	(2.076 - 3.821)	1.935***	(1.522 - 2.460)	1.460**	(1.140 - 1.870)	1.208	(0.941 - 1.551)
renal failure	1.124	(0.917 - 1.377)	1.252**	(1.087 - 1.442)	1.766***	(1.544 - 2.020)	1.278***	(1.108 - 1.473)
rheumatoid arthritis/collagen vascular diseases	0.954	(0.531 - 1.713)	1.248	(0.860 - 1.812)	1.014	(0.683 - 1.507)	0.761	(0.501 - 1.158)
solid tumor without metastasis	0.599***	(0.491 - 0.731)	0.962	(0.846 - 1.094)	0.798***	(0.700 - 0.909)	1.070	(0.946 - 1.211)
valvular disease	1.000	(0.750 - 1.333)	1.075	(0.870 - 1.328)	1.042	(0.844 - 1.287)	0.893	(0.713 - 1.117)
weight loss	0.906	(0.729 - 1.127)	1.810***	(1.590 - 2.060)	1.448***	(1.268 - 1.653)	1.679***	(1.479 - 1.905)
**HEALTHCARE COVARIATES**								
admission reason								
referral	Ref.		Ref.		Ref.		Ref.	
emergency case	2.028***	(1.675 - 2.454)	1.335***	(1.170 - 1.523)	1.342***	(1.169 - 1.540)	1.291***	(1.138 - 1.466)
transfer from other hospital	2.679***	(1.874 - 3.828)	1.859***	(1.406 - 2.459)	1.927***	(1.461 - 2.541)	1.484**	(1.131 - 1.948)
weekend surgery								
no	Ref.		Ref.		Ref.		Ref.	
yes	1.960***	(1.483 - 2.591)	1.427**	(1.150 - 1.770)	1.391**	(1.127 - 1.717)	0.985	(0.784 - 1.238)

colon and rectum resection								
no	Ref.		Ref.		Ref.		Ref.	
yes	2.579***	(2.163 - 3.074)	2.164***	(1.921 - 2.438)	1.859***	(1.645 - 2.100)	1.522***	(1.356 - 1.708)
**HOSPITAL COVARIATES**								
case volume	0.703***	(0.611 - 0.809)	0.844*	(0.725 - 0.982)	0.853**	(0.760 - 0.958)	0.973	(0.854 - 1.109)
area								
urban	Ref.		Ref.		Ref.		Ref.	
rural	1.072	(0.817 - 1.407)	0.904	(0.639 - 1.281)	0.834	(0.670 - 1.037)	0.854	(0.663 - 1.101)
university hospital								
no	Ref.		Ref.		Ref.		Ref.	
yes	1.616	(0.979 - 2.665)	0.853	(0.379 - 1.920)	1.299	(0.829 - 2.037)	2.292**	(1.358 - 3.869)
ownership								
public	Ref.		Ref.		Ref.		Ref.	
non-profit	0.851	(0.614 - 1.179)	1.059	(0.674 - 1.664)	0.762	(0.576 - 1.008)	0.761	(0.553 - 1.048)
private	0.925	(0.682 - 1.254)	1.267	(0.865 - 1.858)	1.597***	(1.256 - 2.030)	0.846	(0.642 - 1.117)

Note: Elixhauser Groups drug abuse, peptic ulcer disease and AIDS/ HIV were not included into the regression due to a low case numbers
*** p<0.001
** p <0.01
* p<0.05

S11: Multivariate analysis of case-, care- and hospital-related covariates of patient safety including 25.805 colon cancer resections in 201 hospitals

	**IN-HOSPITAL DEATH**		**POST-OPERATIVE RESPIRATORY FAILURE**		**RENAL FAILURE**		**POST-OPERATIVE WOUND INFECTION**	
	OR	95% CI	OR	95% CI	OR	95% CI	OR	95% CI
**CASE COVARIATES**								
sex								
male	Ref.		Ref		Ref		Ref	
female	0.867**	(0.780 - 0.963)	0.810***	(0.747 - 0.878)	0.623***	(0.572 - 0.678)	0.879**	(0.807 - 0.959)
age	1.039***	(1.034 - 1.045)	1.008***	(1.005 - 1.012)	1.018***	(1.014 - 1.022)	0.998	(0.994 - 1.002)
alcohol abuse	1.448*	(1.087 - 1.928)	1.273	(0.982 - 1.650)	1.297*	(1.006 - 1.672)	1.222	(0.919 - 1.624)
blood loss anemia	0.552***	(0.405 - 0.752)	0.926	(0.743 - 1.153)	0.720**	(0.576 - 0.899)	1.199	(0.953 - 1.507)
cardiac arrhythmias	1.616***	(1.440 - 1.814)	1.560***	(1.422 - 1.712)	1.639***	(1.492 - 1.800)	1.229***	(1.108 - 1.363)
chronic pulmonary disease	1.329***	(1.142 - 1.546)	1.450***	(1.288 - 1.632)	1.059	(0.931 - 1.204)	1.213**	(1.064 - 1.384)
coagulopathy	4.057***	(3.617 - 4.550)	3.121***	(2.837 - 3.434)	3.066***	(2.780 - 3.383)	1.679***	(1.515 - 1.861)
congestive heart failure	1.766***	(1.539 - 2.026)	1.848***	(1.649 - 2.071)	1.629***	(1.453 - 1.827)	1.143*	(1.000 - 1.306)
deficiency anemia	0.687***	(0.550 - 0.858)	1.027	(0.880 - 1.198)	0.894	(0.756 - 1.057)	0.945	(0.793 - 1.126)
depression	0.760*	(0.610 - 0.946)	1.409***	(1.212 - 1.637)	1.053	(0.891 - 1.243)	1.557***	(1.336 - 1.813)
diabetes, complicated	1.213	(0.986 - 1.491)	0.969	(0.817 - 1.149)	1.351***	(1.150 - 1.587)	1.077	(0.891 - 1.302)
diabetes, uncomplicated	1.100	(0.965 - 1.253)	1.125*	(1.017 - 1.243)	1.090	(0.982 - 1.210)	1.074	(0.963 - 1.198)
fluid and electrolyte disorders	1.873***	(1.670 - 2.100)	2.338***	(2.145 - 2.547)	2.755***	(2.512 - 3.021)	2.038***	(1.857 - 2.236)
hypertension, complicated	0.511***	(0.409 - 0.639)	0.846	(0.711 - 1.008)	0.795*	(0.667 - 0.947)	0.941	(0.770 - 1.151)
hypertension, uncomplicated	0.624***	(0.559 - 0.698)	0.960	(0.881 - 1.047)	0.996	(0.909 - 1.090)	0.997	(0.909 - 1.094)
hypothyroidism	0.879	(0.752 - 1.028)	1.071	(0.955 - 1.201)	1.018	(0.900 - 1.151)	1.200**	(1.064 - 1.354)
liver disease	3.393***	(2.941 - 3.914)	1.375***	(1.202 - 1.571)	2.346***	(2.065 - 2.664)	0.906	(0.777 - 1.056)
lymphoma	0.816	(0.463 - 1.437)	0.766	(0.477 - 1.231)	1.366	(0.891 - 2.093)	1.283	(0.798 - 2.063)
metastatic cancer	1.414***	(1.273 - 1.571)	1.089*	(1.005 - 1.181)	0.986	(0.905 - 1.073)	1.053	(0.966 - 1.148)
obesity	0.745**	(0.621 - 0.894)	1.123	(0.994 - 1.269)	1.431***	(1.266 - 1.616)	1.513***	(1.337 - 1.713)
other neurological disorders	1.337**	(1.080 - 1.655)	1.818***	(1.532 - 2.157)	1.512***	(1.263 - 1.810)	1.214	(0.997 - 1.479)
paralysis	1.754***	(1.367 - 2.250)	1.402**	(1.135 - 1.733)	1.114	(0.889 - 1.395)	1.209	(0.951 - 1.537)
peripheral vascular disorders	1.644***	(1.420 - 1.904)	1.295***	(1.144 - 1.465)	1.290***	(1.138 - 1.462)	1.217**	(1.068 - 1.387)
psychoses	1.170	(0.607 - 2.253)	1.520	(0.948 - 2.437)	1.222	(0.731 - 2.045)	1.359	(0.820 - 2.252)
pulmonary circulation disorders	1.840***	(1.511 - 2.240)	1.977***	(1.675 - 2.333)	1.360***	(1.141 - 1.620)	1.088	(0.898 - 1.318)
renal failure	1.248***	(1.098 - 1.420)	1.162**	(1.047 - 1.289)	1.538***	(1.389 - 1.702)	1.143*	(1.018 - 1.282)
rheumatoid arthritis/collagen vascular diseases	0.883	(0.559 - 1.395)	0.647*	(0.441 - 0.949)	0.894	(0.626 - 1.277)	0.884	(0.599 - 1.305)
solid tumor without metastasis	0.329***	(0.212 - 0.511)	0.806	(0.520 - 1.252)	0.507**	(0.332 - 0.773)	1.526	(0.894 - 2.605)
valvular disease	0.671***	(0.548 - 0.821)	0.875	(0.747 - 1.024)	0.914	(0.781 - 1.069)	0.920	(0.769 - 1.100)
weight loss	1.007	(0.882 - 1.150)	1.586***	(1.434 - 1.753)	1.366***	(1.233 - 1.514)	1.583***	(1.421 - 1.763)
**HEALTHCARE COVARIATES**								
admission reason								
referral	Ref.		Ref.		Ref.		Ref.	
emergency case	1.822***	(1.613 - 2.058)	1.202***	(1.089 - 1.326)	1.407***	(1.266 - 1.564)	1.091	(0.983 - 1.211)
transfer from other hospital	2.491***	(1.962 - 3.161)	1.697***	(1.371 - 2.101)	1.556***	(1.247 - 1.941)	1.389**	(1.112 - 1.734)
weekend surgery								
no	Ref.		Ref.		Ref.		Ref.	
yes	1.949***	(1.650 - 2.303)	1.377***	(1.187 - 1.598)	1.578***	(1.365 - 1.824)	1.049	(0.885 - 1.244)

total colon resection								
no	Ref.		Ref.		Ref.		Ref.	
yes	2.046***	(1.669 - 2.509)	1.691***	(1.420 - 2.014)	1.703***	(1.422 - 2.038)	1.182	(0.986 - 1.417)
colon and rectum resection								
no	Ref.		Ref.		Ref.		Ref.	
yes	1.025	(0.853 - 1.232)	1.617***	(1.422 - 1.837)	1.394***	(1.214 - 1.600)	1.753***	(1.542 - 1.993)
**HOSPITAL COVARIATES**								
case volume	0.926	(0.810 - 1.058)	0.939	(0.795 - 1.110)	0.994	(0.865 - 1.142)	1.146	(0.975 - 1.348)
area								
urban	Ref.		Ref.		Ref.		Ref.	
rural	1.042	(0.857 - 1.267)	0.815	(0.605 - 1.099)	0.793*	(0.639 - 0.983)	0.964	(0.750 - 1.239)
university hospital								
no	Ref.		Ref.		Ref.		Ref.	
yes	1.301	(0.877 - 1.931)	0.650	(0.318 - 1.328)	1.280	(0.790 - 2.076)	2.209**	(1.281 - 3.807)
ownership								
public	Ref.		Ref.		Ref.		Ref.	
non-profit	1.146	(0.898 - 1.463)	1.098	(0.745 - 1.618)	0.876	(0.660 - 1.162)	0.767	(0.557 - 1.056)
private	1.207	(0.970 - 1.502)	1.316	(0.951 - 1.821)	1.993***	(1.572 - 2.526)	0.776	(0.591 - 1.018)

Note: Elixhauser Groups drug abuse, peptic ulcer disease and AIDS/ HIV were not included into the regression due to a low case numbers
*** p<0.001
** p <0.01
* p<0.05

S12: Multivariate analysis of case-, care- and hospital-related covariates of patient safety including 13.703 rectum cancer resections in 197 hospitals

	**IN-HOSPITAL DEATH**		**POST-OPERATIVE RESPIRATORY FAILURE**		**RENAL FAILURE**		**POST-OPERATIVE WOUND INFECTION**	
	OR	95% CI	OR	95% CI	OR	95% CI	OR	95% CI
**CASE COVARIATES**								
sex								
male	Ref.		Ref.		Ref.		Ref.	
female	0.912	(0.738 - 1.127)	0.892	(0.789 - 1.009)	0.685***	(0.599 - 0.783)	0.888	(0.789 - 1.000)
age	1.066***	(1.054 - 1.079)	1.013***	(1.007 - 1.019)	1.019***	(1.013 - 1.026)	0.998	(0.993 - 1.003)
alcohol abuse	1.807*	(1.034 - 3.156)	1.603*	(1.076 - 2.389)	1.156	(0.770 - 1.736)	1.138	(0.768 - 1.687)
blood loss anemia	0.823	(0.426 - 1.589)	0.677	(0.415 - 1.102)	1.231	(0.812 - 1.867)	1.466	(0.961 - 2.236)
cardiac arrhythmias	1.704***	(1.361 - 2.135)	1.714***	(1.484 - 1.981)	1.628***	(1.402 - 1.890)	1.050	(0.901 - 1.223)
chronic pulmonary disease	1.540**	(1.149 - 2.065)	1.473***	(1.218 - 1.781)	1.209	(0.986 - 1.482)	1.389***	(1.153 - 1.673)
coagulopathy	3.691***	(2.941 - 4.632)	2.783***	(2.396 - 3.233)	2.487***	(2.126 - 2.909)	1.723***	(1.493 - 1.990)
congestive heart failure	1.784***	(1.363 - 2.335)	1.798***	(1.488 - 2.172)	1.557***	(1.287 - 1.885)	1.028	(0.837 - 1.262)
deficiency anemia	1.008	(0.631 - 1.610)	1.404*	(1.049 - 1.879)	1.527**	(1.129 - 2.066)	1.269	(0.945 - 1.703)
depression	0.665	(0.418 - 1.058)	1.299*	(1.026 - 1.646)	1.359*	(1.060 - 1.742)	1.449***	(1.170 - 1.793)
diabetes, complicated	1.285	(0.830 - 1.990)	1.061	(0.798 - 1.409)	1.190	(0.906 - 1.562)	1.146	(0.860 - 1.528)
diabetes, uncomplicated	1.060	(0.817 - 1.375)	1.194*	(1.022 - 1.396)	1.075	(0.913 - 1.267)	1.243**	(1.067 - 1.448)
fluid and electrolyte disorders	1.693***	(1.353 - 2.117)	2.162***	(1.903 - 2.456)	2.720***	(2.367 - 3.126)	1.966***	(1.738 - 2.223)
hypertension, complicated	0.590*	(0.379 - 0.918)	0.844	(0.627 - 1.137)	0.844	(0.630 - 1.130)	0.894	(0.652 - 1.226)
hypertension, uncomplicated	0.625***	(0.502 - 0.780)	0.994	(0.873 - 1.132)	1.106	(0.962 - 1.272)	0.995	(0.877 - 1.129)
hypothyroidism	0.878	(0.633 - 1.217)	1.032	(0.860 - 1.239)	0.965	(0.791 - 1.177)	0.995	(0.832 - 1.191)
liver disease	3.342***	(2.461 - 4.538)	1.126	(0.879 - 1.443)	2.363***	(1.894 - 2.949)	1.171	(0.924 - 1.486)
lymphoma	1.440	(0.468 - 4.431)	1.006	(0.429 - 2.360)	1.225	(0.519 - 2.893)	1.918	(0.891 - 4.126)
metastatic cancer	1.280*	(1.035 - 1.581)	1.212**	(1.071 - 1.371)	1.015	(0.889 - 1.158)	1.041	(0.925 - 1.172)
obesity	0.953	(0.684 - 1.330)	1.476***	(1.241 - 1.755)	1.607***	(1.349 - 1.915)	1.570***	(1.333 - 1.849)
other neurological disorders	2.294***	(1.562 - 3.369)	2.146***	(1.634 - 2.818)	1.198	(0.885 - 1.621)	1.598**	(1.207 - 2.116)
paralysis	1.418	(0.866 - 2.321)	1.422*	(1.014 - 1.993)	1.249	(0.878 - 1.776)	1.130	(0.799 - 1.600)
peripheral vascular disorders	1.561**	(1.190 - 2.047)	1.151	(0.970 - 1.367)	1.259*	(1.055 - 1.503)	1.423***	(1.214 - 1.669)
psychoses	0.417	(0.045 - 3.841)	0.477	(0.147 - 1.541)	1.904	(0.756 - 4.792)	1.012	(0.413 - 2.481)
pulmonary circulation disorders	3.547***	(2.491 - 5.049)	2.146***	(1.633 - 2.819)	1.567**	(1.179 - 2.082)	1.234	(0.923 - 1.650)
renal failure	0.942	(0.721 - 1.231)	1.125	(0.949 - 1.334)	1.750***	(1.489 - 2.056)	1.233*	(1.039 - 1.462)
rheumatoid arthritis/collagen vascular diseases	1.041	(0.443 - 2.445)	0.825	(0.462 - 1.476)	0.962	(0.544 - 1.701)	0.600	(0.312 - 1.154)
solid tumor without metastasis	0.105***	(0.039 - 0.282)	0.960	(0.353 - 2.613)	1.074	(0.327 - 3.534)	1.648	(0.542 - 5.013)
valvular disease	0.808	(0.550 - 1.185)	1.099	(0.855 - 1.413)	0.922	(0.712 - 1.193)	0.969	(0.743 - 1.265)
weight loss	0.967	(0.745 - 1.255)	1.761***	(1.517 - 2.045)	1.333***	(1.142 - 1.558)	1.557***	(1.345 - 1.803)
**HEALTHCARE COVARIATES**								
admission reason								
referral	Ref.		Ref.		Ref.		Ref.	
emergency case	1.937***	(1.526 - 2.460)	1.199*	(1.018 - 1.413)	1.257*	(1.055 - 1.498)	1.468***	(1.255 - 1.718)
transfer from other hospital	2.503***	(1.487 - 4.213)	2.160***	(1.497 - 3.118)	1.731**	(1.180 - 2.540)	1.805**	(1.263 - 2.578)
weekend surgery								
no	Ref.		Ref.		Ref.		Ref.	
yes	2.137***	(1.417 - 3.224)	1.384*	(1.024 - 1.870)	1.267	(0.940 - 1.708)	1.148	(0.845 - 1.560)

colon and rectum resection								
no	Ref.		Ref.		Ref.		Ref.	
yes	2.422***	(1.940 - 3.024)	2.085***	(1.806 - 2.408)	1.711***	(1.470 - 1.991)	1.627***	(1.415 - 1.871)
**HOSPITAL COVARIATES**								
case volume	0.678***	(0.577 - 0.796)	0.907	(0.764 - 1.075)	0.835**	(0.730 - 0.955)	0.988	(0.851 - 1.147)
area								
urban	Ref.		Ref.		Ref.		Ref.	
rural	1.164	(0.862 - 1.572)	0.951	(0.661 - 1.369)	0.808	(0.632 - 1.033)	0.843	(0.638 - 1.113)
university hospital								
no	Ref.		Ref.		Ref.		Ref.	
yes	1.512	(0.889 - 2.571)	0.807	(0.355 - 1.837)	1.374	(0.840 - 2.246)	2.195**	(1.267 - 3.801)
ownership								
public	Ref.		Ref.		Ref.		Ref.	
non-profit	1.034	(0.725 - 1.473)	1.089	(0.682 - 1.740)	0.797	(0.580 - 1.095)	0.733	(0.518 - 1.037)
private	0.900	(0.635 - 1.276)	1.238	(0.828 - 1.850)	1.807***	(1.375 - 2.376)	0.853	(0.629 - 1.158)

Note: Elixhauser Groups drug abuse, peptic ulcer disease and AIDS/ HIV were not included into the regression due to a low case numbers
*** p<0.001
** p <0.01
* p<0.05

S13: Multivariate analysis of case-, care- and hospital-related covariates of patient safety including 28.363 non-cancer colon resections in 208 hospitals

	**IN-HOSPITAL DEATH**		**POST-OPERATIVE RESPIRATORY FAILURE**		**RENAL FAILURE**		**POST-OPERATIVE WOUND INFECTION**	
	OR	95% CI	OR	95% CI	OR	95% CI	OR	95% CI
**CASE COVARIATES**								
sex								
male	Ref.		Ref		Ref		Ref	
female	0.988	(0.896 - 1.090)	0.746***	(0.691 - 0.807)	0.748***	(0.689 - 0.811)	0.872***	(0.804 - 0.946)
age	1.056***	(1.051 - 1.060)	1.016***	(1.014 - 1.019)	1.026***	(1.023 - 1.030)	0.997	(0.995 - 1.000)
alcohol abuse	1.483***	(1.190 - 1.847)	2.400***	(1.985 - 2.902)	1.745***	(1.435 - 2.121)	1.350**	(1.099 - 1.657)
blood loss anemia	0.388***	(0.246 - 0.612)	1.296	(0.945 - 1.777)	1.140	(0.831 - 1.565)	1.150	(0.828 - 1.597)
cardiac arrhythmias	1.666***	(1.499 - 1.851)	1.627***	(1.487 - 1.781)	1.605***	(1.464 - 1.760)	1.166**	(1.053 - 1.291)
chronic pulmonary disease	1.405***	(1.231 - 1.603)	1.463***	(1.310 - 1.633)	1.154*	(1.028 - 1.296)	1.264***	(1.123 - 1.422)
coagulopathy	4.166***	(3.747 - 4.632)	2.920***	(2.665 - 3.198)	3.515***	(3.209 - 3.851)	1.602***	(1.451 - 1.769)
congestive heart failure	1.764***	(1.562 - 1.993)	1.972***	(1.770 - 2.197)	1.733***	(1.555 - 1.932)	1.282***	(1.135 - 1.448)
deficiency anemia	0.943	(0.732 - 1.214)	1.219	(0.995 - 1.493)	1.428***	(1.166 - 1.749)	1.188	(0.967 - 1.459)
depression	0.582***	(0.472 - 0.717)	1.488***	(1.288 - 1.719)	1.071	(0.918 - 1.249)	1.564***	(1.354 - 1.807)
diabetes, complicated	1.332**	(1.105 - 1.606)	1.430***	(1.211 - 1.688)	1.187*	(1.005 - 1.404)	1.326**	(1.104 - 1.593)
diabetes, uncomplicated	1.170*	(1.026 - 1.334)	1.304***	(1.172 - 1.450)	1.226***	(1.098 - 1.368)	1.172**	(1.046 - 1.314)
fluid and electrolyte disorders	1.828***	(1.641 - 2.037)	2.715***	(2.495 - 2.955)	2.930***	(2.681 - 3.202)	1.929***	(1.764 - 2.110)
hypertension, complicated	0.527***	(0.430 - 0.645)	0.680***	(0.570 - 0.812)	0.847	(0.712 - 1.008)	0.965	(0.794 - 1.172)
hypertension, uncomplicated	0.489***	(0.440 - 0.543)	0.933	(0.858 - 1.015)	0.861***	(0.789 - 0.940)	1.107*	(1.011 - 1.210)
hypothyroidism	0.729***	(0.631 - 0.842)	1.152*	(1.034 - 1.283)	1.064	(0.949 - 1.192)	1.210***	(1.081 - 1.354)
liver disease	5.708***	(4.967 - 6.559)	1.408***	(1.238 - 1.601)	2.827***	(2.491 - 3.207)	0.802**	(0.694 - 0.925)
lymphoma	1.016	(0.683 - 1.513)	0.858	(0.602 - 1.223)	0.895	(0.624 - 1.284)	0.978	(0.668 - 1.433)
metastatic cancer	-		-		-		-	
obesity	0.768***	(0.657 - 0.897)	1.180**	(1.055 - 1.321)	1.557***	(1.390 - 1.745)	1.737***	(1.557 - 1.938)
other neurological disorders	1.612***	(1.360 - 1.912)	1.949***	(1.686 - 2.253)	1.498***	(1.289 - 1.740)	1.313***	(1.123 - 1.536)
paralysis	1.284*	(1.039 - 1.587)	1.482***	(1.237 - 1.777)	1.124	(0.930 - 1.358)	1.238*	(1.021 - 1.500)
peripheral vascular disorders	2.129***	(1.886 - 2.405)	1.626***	(1.458 - 1.813)	1.675***	(1.500 - 1.871)	1.266***	(1.125 - 1.426)
psychoses	0.518*	(0.279 - 0.963)	2.462***	(1.639 - 3.698)	0.660	(0.405 - 1.074)	1.516	(0.994 - 2.313)
pulmonary circulation disorders	1.348**	(1.094 - 1.662)	1.737***	(1.445 - 2.088)	1.296**	(1.070 - 1.570)	1.040	(0.849 - 1.274)
renal failure	1.381***	(1.228 - 1.553)	1.073	(0.969 - 1.189)	1.494***	(1.351 - 1.653)	0.996	(0.889 - 1.117)
rheumatoid arthritis/collagen vascular diseases	1.046	(0.791 - 1.384)	1.120	(0.885 - 1.416)	0.981	(0.770 - 1.250)	1.143	(0.893 - 1.462)
solid tumor without metastasis	-		-		-		-	
valvular disease	0.954	(0.803 - 1.133)	0.861	(0.738 - 1.004)	0.944	(0.810 - 1.102)	0.942	(0.795 - 1.116)
weight loss	0.799**	(0.696 - 0.917)	1.717***	(1.543 - 1.910)	1.520***	(1.365 - 1.693)	2.013***	(1.803 - 2.248)
**HEALTHCARE COVARIATES**								
admission reason								
referral	Ref.		Ref.		Ref.		Ref.	
emergency case	1.936***	(1.706 - 2.197)	1.727***	(1.564 - 1.906)	1.479***	(1.330 - 1.645)	1.197***	(1.086 - 1.320)
transfer from other hospital	2.610***	(2.169 - 3.140)	2.299***	(1.959 - 2.698)	2.027***	(1.718 - 2.391)	1.202*	(1.015 - 1.423)
weekend surgery								
no	Ref.		Ref.		Ref.		Ref.	
yes	1.577***	(1.397 - 1.780)	1.436***	(1.296 - 1.590)	1.443***	(1.299 - 1.604)	1.095	(0.978 - 1.225)

total colon resection								
no	Ref.		Ref.		Ref.		Ref.	
yes	3.173***	(2.705 - 3.723)	1.604***	(1.395 - 1.843)	2.545***	(2.213 - 2.927)	0.958	(0.830 - 1.107)
colon and rectum resection								
no	Ref.		Ref.		Ref.		Ref.	
yes	1.159	(0.936 - 1.436)	1.320**	(1.116 - 1.562)	1.404***	(1.183 - 1.667)	1.314**	(1.107 - 1.560)
**HOSPITAL COVARIATES**								
case volume	0.961	(0.844 - 1.094)	0.876	(0.753 - 1.018)	0.950	(0.841 - 1.073)	1.135	(0.987 - 1.304)
area								
urban	Ref.		Ref.		Ref.		Ref.	
rural	1.091	(0.886 - 1.345)	0.918	(0.682 - 1.235)	0.775*	(0.631 - 0.953)	1.058	(0.837 - 1.336)
university hospital								
no	Ref.		Ref.		Ref.		Ref.	
yes	1.336	(0.855 - 2.087)	0.712	(0.347 - 1.461)	1.586	(0.999 - 2.520)	1.802*	(1.070 - 3.034)
ownership								
public	Ref.		Ref.		Ref.		Ref.	
non-profit	0.900	(0.688 - 1.177)	1.028	(0.696 - 1.519)	0.836	(0.638 - 1.096)	0.736*	(0.543 - 0.996)
private	1.284*	(1.015 - 1.626)	1.433*	(1.035 - 1.986)	1.893***	(1.505 - 2.380)	0.775	(0.601 - 1.001)

Note: Elixhauser Groups drug abuse, peptic ulcer disease and AIDS/ HIV were not included into the regression due to a low case numbers
*** p<0.001
** p <0.01
* p<0.05

S14: Multivariate analysis of case-, care- and hospital-related covariates of patient safety including 6.692 non-cancer rectum resections in 195 hospitals

	**IN-HOSPITAL DEATH**		**POST-OPERATIVE RESPIRATORY FAILURE**		**RENAL FAILURE**		**POST-OPERATIVE WOUND INFECTION**	
	OR	95% CI	OR	95% CI	OR	95% CI	OR	95% CI
**CASE COVARIATES**								
sex								
male	Ref.		Ref		Ref		Ref	
female	0.733*	(0.542 - 0.992)	0.739**	(0.601 - 0.908)	0.746**	(0.607 - 0.917)	0.651***	(0.539 - 0.786)
age	1.074***	(1.058 - 1.089)	1.013***	(1.006 - 1.021)	1.023***	(1.016 - 1.031)	1.000	(0.994 - 1.006)
alcohol abuse	0.809	(0.348 - 1.882)	1.801*	(1.008 - 3.219)	1.355	(0.754 - 2.434)	1.080	(0.601 - 1.941)
blood loss anemia	0.797	(0.305 - 2.082)	1.735	(0.877 - 3.432)	1.886	(0.970 - 3.668)	1.927*	(1.014 - 3.663)
cardiac arrhythmias	2.130***	(1.569 - 2.892)	1.573***	(1.243 - 1.990)	1.608***	(1.281 - 2.017)	1.015	(0.795 - 1.295)
chronic pulmonary disease	0.908	(0.601 - 1.371)	1.570**	(1.180 - 2.088)	0.977	(0.726 - 1.315)	1.402*	(1.066 - 1.845)
coagulopathy	5.767***	(4.191 - 7.937)	3.713***	(2.934 - 4.697)	3.703***	(2.949 - 4.650)	1.947***	(1.550 - 2.447)
congestive heart failure	1.851***	(1.309 - 2.617)	1.887***	(1.426 - 2.497)	1.543**	(1.180 - 2.017)	1.269	(0.943 - 1.708)
deficiency anemia	0.765	(0.362 - 1.616)	1.008	(0.606 - 1.675)	1.039	(0.640 - 1.689)	1.430	(0.911 - 2.244)
depression	0.591	(0.326 - 1.073)	1.043	(0.723 - 1.505)	1.220	(0.859 - 1.732)	1.208	(0.869 - 1.679)
diabetes, complicated	2.158**	(1.267 - 3.674)	0.990	(0.627 - 1.564)	2.155***	(1.423 - 3.265)	1.292	(0.821 - 2.032)
diabetes, uncomplicated	1.233	(0.838 - 1.815)	1.133	(0.849 - 1.513)	1.549**	(1.176 - 2.038)	0.950	(0.709 - 1.272)
fluid and electrolyte disorders	1.619**	(1.185 - 2.213)	3.048***	(2.458 - 3.779)	3.006***	(2.430 - 3.719)	1.944***	(1.594 - 2.370)
hypertension, complicated	0.345***	(0.189 - 0.628)	0.657	(0.419 - 1.031)	0.910	(0.596 - 1.390)	1.042	(0.663 - 1.638)
hypertension, uncomplicated	0.511***	(0.377 - 0.694)	0.928	(0.751 - 1.147)	0.813	(0.658 - 1.003)	1.041	(0.851 - 1.273)
hypothyroidism	0.742	(0.491 - 1.121)	0.919	(0.695 - 1.214)	1.051	(0.803 - 1.376)	1.093	(0.845 - 1.416)
liver disease	5.966***	(3.793 - 9.384)	1.550*	(1.068 - 2.252)	2.060***	(1.451 - 2.925)	0.795	(0.540 - 1.172)
lymphoma	0.685	(0.151 - 3.108)	0.930	(0.309 - 2.800)	0.656	(0.221 - 1.944)	0.884	(0.308 - 2.539)
metastatic cancer	-		-		-		-	
obesity	0.853	(0.538 - 1.352)	1.534**	(1.152 - 2.043)	1.400*	(1.054 - 1.861)	1.871***	(1.460 - 2.396)
other neurological disorders	2.558***	(1.586 - 4.126)	1.428	(0.968 - 2.106)	1.117	(0.755 - 1.652)	1.113	(0.754 - 1.642)
paralysis	0.798	(0.421 - 1.514)	1.276	(0.780 - 2.088)	1.508	(0.940 - 2.421)	1.158	(0.714 - 1.881)
peripheral vascular disorders	2.505***	(1.743 - 3.599)	1.502**	(1.119 - 2.018)	1.629***	(1.227 - 2.163)	1.048	(0.774 - 1.419)
psychoses	1.703	(0.166 - 17.483)	1.385	(0.317 - 6.052)	3.384	(0.925 - 12.382)	1.386	(0.359 - 5.356)
pulmonary circulation disorders	1.770	(0.965 - 3.246)	1.636	(0.983 - 2.725)	1.114	(0.667 - 1.859)	1.203	(0.725 - 1.997)
renal failure	1.472*	(1.047 - 2.068)	1.633***	(1.252 - 2.129)	1.755***	(1.368 - 2.250)	1.395*	(1.071 - 1.818)
rheumatoid arthritis/collagen vascular diseases	0.864	(0.358 - 2.087)	1.948*	(1.171 - 3.242)	1.083	(0.609 - 1.925)	1.001	(0.571 - 1.754)
solid tumor without metastasis	-		-		-		-	
valvular disease	1.236	(0.771 - 1.982)	0.999	(0.671 - 1.488)	1.300	(0.888 - 1.903)	0.719	(0.469 - 1.104)
weight loss	0.761	(0.507 - 1.143)	1.969***	(1.503 - 2.580)	1.944***	(1.504 - 2.513)	2.204***	(1.709 - 2.843)
**HEALTHCARE COVARIATES**								
admission reason								
referral	Ref.		Ref.		Ref.		Ref.	
emergency case	2.060***	(1.470 - 2.885)	1.590***	(1.253 - 2.018)	1.382**	(1.094 - 1.746)	0.992	(0.797 - 1.234)
transfer from other hospital	2.583***	(1.488 - 4.487)	1.510	(0.962 - 2.369)	1.949**	(1.269 - 2.993)	1.102	(0.713 - 1.704)
weekend surgery								
no	Ref.		Ref.		Ref.		Ref.	
yes	1.770**	(1.179 - 2.658)	1.391*	(1.012 - 1.912)	1.499*	(1.101 - 2.042)	0.870	(0.613 - 1.235)

colon and rectum resection								
no	Ref.		Ref.		Ref.		Ref.	
yes	2.967***	(2.184 - 4.030)	2.183***	(1.750 - 2.725)	2.190***	(1.771 - 2.709)	1.318*	(1.066 - 1.630)
**HOSPITAL COVARIATES**								
case volume	0.777*	(0.614 - 0.983)	0.793*	(0.642 - 0.978)	0.863	(0.733 - 1.015)	0.956	(0.795 - 1.150)
area								
urban	Ref.		Ref.		Ref.		Ref.	
rural	0.851	(0.536 - 1.351)	0.881	(0.580 - 1.337)	0.872	(0.646 - 1.176)	0.865	(0.613 - 1.220)
university hospital								
no	Ref.		Ref.		Ref.		Ref.	
yes	1.529	(0.712 - 3.283)	0.849	(0.354 - 2.034)	1.176	(0.701 - 1.972)	2.375**	(1.316 - 4.287)
ownership								
public	Ref.		Ref.		Ref.		Ref.	
non-profit	0.556*	(0.323 - 0.959)	1.098	(0.652 - 1.849)	0.702	(0.489 - 1.008)	0.775	(0.516 - 1.166)
private	0.981	(0.594 - 1.621)	1.614*	(1.020 - 2.556)	1.331	(0.960 - 1.846)	0.791	(0.546 - 1.147)

Note: Elixhauser Groups drug abuse, peptic ulcer disease and AIDS/ HIV were not included into the regression due to a low case numbers
*** p<0.001
** p <0.01
* p<0.05

S15 – Figure: Interactions between emergency admission and university hospital status

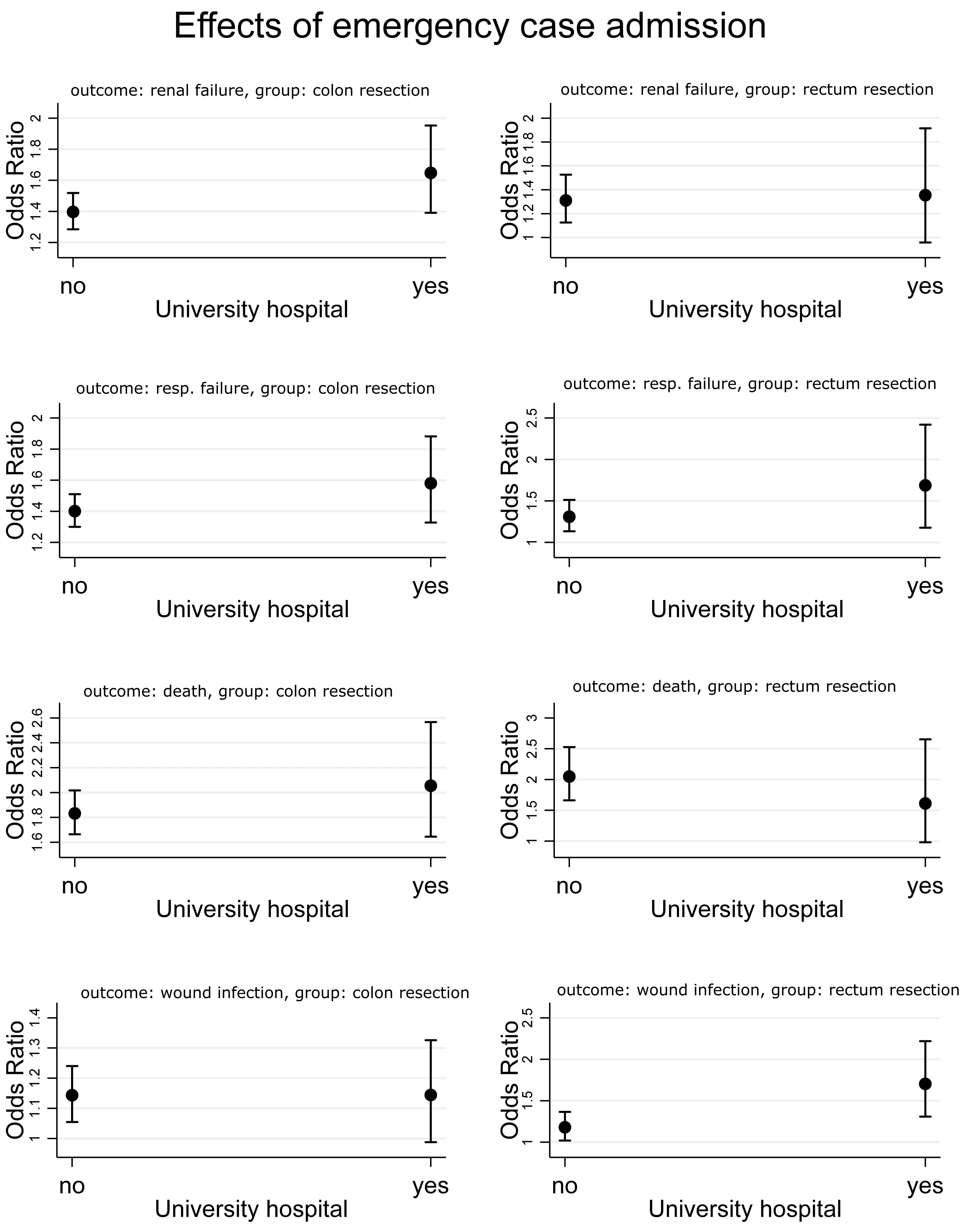
Effects of emergency case admission
outcome: renal failure, group: colon resection
outcome: renal failure, group: rectum resection
outcome: resp. failure, group: colon resection
outcome: resp. failure, group: rectum resection
outcome: death, group: colon resection
outcome: death, group: rectum resection
outcome: wound infection, group: colon resection
outcome: wound infection, group: rectum resection
Odds Ratio
no
yes
University hospital

S16 – Figure: Interactions between transfer from other hospital admission and university hospital status

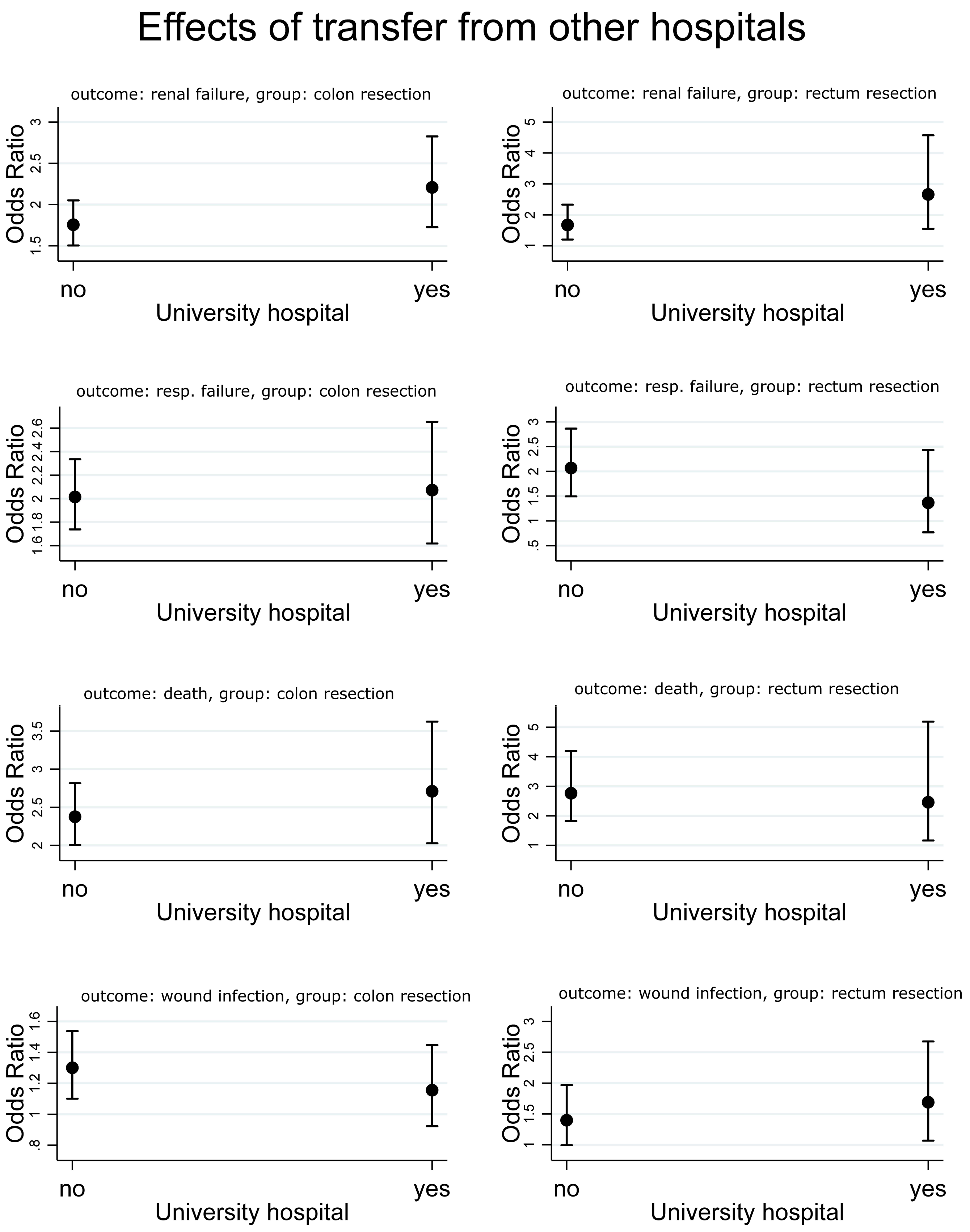
Effects of transfer from other hospitals
outcome: renal failure, group: colon resection
outcome: renal failure, group: rectum resection
outcome: resp. failure, group: colon resection
outcome: resp. failure, group: rectum resection
outcome: death, group: colon resection
outcome: death, group: rectum resection
outcome: wound infection, group: colon resection
outcome: wound infection, group: rectum resection
Odds Ratio
University hospital
no
yes
