# Supplementary material for: Relationships between multiple patient safety outcomes and healthcare and hospital-related risk factors in colorectal resection cases: cross-sectional evidence from a nationwide sample of 232 German hospitals
Source: BMJ Open. 2022 Jul 25;12(7):e058481. doi: 10.1136/bmjopen-2021-058481 (PMC9328106; doi:10.1136/bmjopen-2021-058481)
Supplement: Author's manuscript [file bmjopen-2021-058481.draft_revisions.pdf]

BMJ Open is committed to open peer review. As part of this commitment we make the peer review history of every article we publish publicly available.

When an article is published we post the peer reviewers' comments and the authors' responses online. We also post the versions of the paper that were used during peer review. These are the versions that the peer review comments apply to.

The versions of the paper that follow are the versions that were submitted during the peer review process. They are not the versions of record or the final published versions. They should not be cited or distributed as the published version of this manuscript.

BMJ Open is an open access journal and the full, final, typeset and author-corrected version of record of the manuscript is available on our site with no access controls, subscription charges or pay-per-view fees (http://bmjopen.bmj.com).

If you have any questions on BMJ Open's open peer review process please email info.bmjopen@bmj.com

Patient safety in colorectal resections – evidence from a nationwide sample

Journal:	*BMJ Open*
Manuscript ID	bmjopen-2021-058481
Article Type:	Original research
Date Submitted by the Author:	18-Oct-2021

Complete List of Authors:	Walther, Felix; TU Dresden Faculty of Medicine Carl Gustav Carus, Center for Evidence-based Healthcare; University Hospital Carl Gustav Carus, Quality and Medical Risk Management<br>Schmitt, Jochen; Medical Faculty Carl Gustav Carus,Technical University Dr.esden, Center for Evidence-based Healthcare<br>Eberlein-Gonska, Maria; Quality and Medical Risk Management, University Hospital Carl Gustav Carus Dresden<br>Kuhlen, Ralf; Initiative Qualitätsmedizin e.V.<br>Scriba, Peter; Initiative Qualitätsmedizin e.V.<br>Schoffer, Olaf; Universitätsklinikum Carl Gustav Carus Zentrum für Evidenzbasierte Gesundheitsversorgung,<br>Roessler, Martin; Universitätsklinikum Carl Gustav Carus Zentrum für Evidenzbasierte Gesundheitsversorgung,

Keywords:	Quality in health care < HEALTH SERVICES ADMINISTRATION & MANAGEMENT, Colorectal surgery < SURGERY, Health & safety < HEALTH SERVICES ADMINISTRATION & MANAGEMENT

SCHOLARONE™
Manuscripts

I, the Submitting Author has the right to grant and does grant on behalf of all authors of the Work (as defined in the below author licence), an exclusive licence and/or a non-exclusive licence for contributions from authors who are: i) UK Crown employees; ii) where BMJ has agreed a CC-BY licence shall apply, and/or iii) in accordance with the terms applicable for US Federal Government officers or employees acting as part of their official duties; on a worldwide, perpetual, irrevocable, royalty-free basis to BMJ Publishing Group Ltd ("BMJ") its licensees and where the relevant Journal is co-owned by BMJ to the co-owners of the Journal, to publish the Work in this journal and any other BMJ products and to exploit all rights, as set out in our licence.

The Submitting Author accepts and understands that any supply made under these terms is made by BMJ to the Submitting Author unless you are acting as an employee on behalf of your employer or a postgraduate student of an affiliated institution which is paying any applicable article publishing charge ("APC") for Open Access articles. Where the Submitting Author wishes to make the Work available on an Open Access basis (and intends to pay the relevant APC), the terms of reuse of such Open Access shall be governed by a Creative Commons licence – details of these licences and which Creative Commons licence will apply to this Work are set out in our licence referred to above.

Other than as permitted in any relevant BMJ Author's Self Archiving Policies, I confirm this Work has not been accepted for publication elsewhere, is not being considered for publication elsewhere and does not duplicate material already published. I confirm all authors consent to publication of this Work and authorise the granting of this licence.

For peer review only

Long title: **Patient safety in colorectal resections – evidence from a nationwide sample**

Short: **Patient safety in colorectal resections**

Abbreviated title/ Short running head: **Patient safety in colorectal resections**

Felix Walther[1,2*], Jochen Schmitt[1], Maria Eberlein-Gonska[2], Ralf Kuhlen[3], Peter C. Scriba[3], Olaf Schoffer[1], Martin Roessler[1]

1 Center for Evidence-based Healthcare, Medical Faculty Carl Gustav Carus, TU Dresden, Dresden, Germany

2 Quality and Medical Risk Management, University Hospital Carl Gustav Carus, Dresden, Germany

3 Initiative Qualitätsmedizin e.V., Berlin, Germany

*** Corresponding author**

Felix.walther@ukdd.de

word (manuscript): 2818

Words (abstract): 300 / 300

For peer review only

For peer review only - http://bmjopen.bmj.com/site/about/guidelines.xhtml

Abstract

objectives: Studies analyzing colorectal resections usually focus on a specific outcome (e.g. mortality) and/ or specific risk factors at the individual (e.g. comorbidities) or hospital (e.g. volume) level. Comprehensive evidence across different patient safety outcomes, risk factors and patient groups is still scarce. Therefore the aim of this analysis was to investigate consistent relationships between multiple patient safety outcomes and care and hospital risk factors in colorectal resection cases.

design: Cross-sectional study

setting: German inpatient routine care data of colorectal resections between 2016 to 2018

participants: We analyzed 54,168 colon resection and 20,395 rectum resection cases treated in German hospitals. The German Inpatient Quality Indicators were used to define colon resections and rectum resections transparently.

primary outcome measures: With in-hospital death, post-operative respiratory failure, renal failure and post-operative wound infections we included multiple patient safety outcomes as primary outcomes / dependent variables for our analysis. Care (e.g. weekend surgery), hospital (e.g. volume) and case (e.g. age) characteristics served as independent covariates in a multilevel logistic regression model. The estimated regression coefficients were transferred into odds ratios (OR).

results: Weekend surgery, emergency admissions and transfers from other hospitals were significantly associated (ORs ranged from 1.1 to 2.6) with poor patient safety outcome (death, renal failure, post-operative respiratory failure) in colon resections and rectum resections. Hospital characteristics showed heterogeneous effects. In colon resections hospital volume was associated with insignificant or adverse associations (post-operative wound infections: OR 1.168 (95% CI 1.030 - 1.325)) to multiple patient safety outcomes. In rectum resections hospital volume was protectively associated to death, renal failure and post-operative respiratory failure (ORs ranged from 0.7 to 0.8).

conclusions: Transfer from other hospital and emergency admission are constantly associated with poor patient safety outcome. Hospital variables like volume, ownership or localization did not show consistent relationships to patient safety outcome.

.

Keywords: patient safety; health services research; Quality of Care; colon surgery; rectum surgery

For peer review only

For peer review only - http://bmjopen.bmj.com/site/about/guidelines.xhtml

Strengths and limitations of this study

- Large and current sample providing a broad span of cases, hospital types, ownerships, and locations.
- Comprehensive analysis previously solitary analyzed outcomes and risk factors
- Use of previously validated outcomes that were reported to occur most likely during hospitalization
- Accounting data lack information on patient history, medication, length of anesthesia, staff-to-patient ratios, surgeon volumes, centralization and which of the coded diagnoses had been present on admission.

For peer review only

For peer review only - http://bmjopen.bmj.com/site/about/guidelines.xhtml

Introduction

Assuring and improving patient safety are important objectives in colon and rectum surgery. The measurement and assurance of patient safety is important for patient outcomes, payment, and accreditation. One of the most frequently used outcome indicator in colorectal resections is in-hospital mortality.[1] However, it has been stressed that patient safety is reflected in both mortality and non-mortality outcomes.[2 3] A measurement of outcomes beyond mortality therefore is necessary for a comprehensive assessment of patient safety and care quality.[1] In addition to multiple relevant patient outcomes, various risk factors for adverse events were analyzed in previous studies. Besides patient characteristics (e.g. age, sex, comorbidities), especially the influence of care (e.g. weekend surgery, emergency, transfer from other hospital) and hospital variables (e.g. volume, urbanization degree) were widely discussed. Weekend surgery,[4-6] emergency admission,[7 8] transfer from other hospitals[9-11], and case volume[12 13] were found to have significant effects on patient outcome (particularly mortality) in colorectal surgery. Analogous to patient outcomes, previous studies usually considered only subsets of these risk factors without analyzing them together.

However, a comprehensive analysis of patient safety and its covariates in colorectal resections should take multiple outcomes and multiple risk factors into account.[14] To our knowledge, such comprehensive analyses have rarely been reported. Based on that assumption, our analysis aimed to investigate whether care and hospital characteristics are consistently associated with multiple patient safety outcomes in colorectal resections. Using a 3-year sample (2016-2018) of German inpatient accounting data we investigated relationships between case, care, and hospital characteristics and the patient safety outcomes in-hospital death, post-operative respiratory failure, renal failure and post-operative wound infections in colorectal resections.

Materials and Methods

This explorative cross-sectional analysis was embedded into the IMPRESS study. The IMPRESS study was a cluster-randomized trial evaluating the effects of clinical peer review on mortality in patients ventilated >24h nested in a prospective cohort study of 232 participating hospitals. Details, baseline and first explorative results of the IMPRESS study were published previously.[16 17] The study has been registered at ISCRTN (ISRCTN10188560).[18] The identification of possible covariates of mortality and non-mortality outcomes in colorectal resections was a secondary aim of the IMPRESS study.

For peer review only - http://bmjopen.bmj.com/site/about/guidelines.xhtml

Data sources

The data used in this study were derived from two routine data sets. We used accounting data according to German law regulating stationary accounting (§21 Krankenhausentgeltgesetz) to gather information concerning age, sex, reason of admission, discharge destination, diagnoses/comorbidities (ICD-10-GM) and medical/surgical procedures (OPS codes). We applied the predefined groups of the Elixhauser comorbidity index and its coding modifications for ICD-10 (supplement S1) to adjust for relevant comorbidities. The Elixhauser Comorbidity Index is a score used to adjust for chronic or non-acute comorbidities in routine data sets.[19 20] To assess hospital characteristics (ownership, university hospital status, urbanization) we used data from the German hospital register („Deutsches Krankenhausverzeichnis“).

Study participation, privacy, and ethics

All participating hospitals submitted a written consent on participation prior to the start of the IMPRESS study. The data trust site at Koordinierungszentrum für Klinische Studien (KKS) Dresden ensured the anonymization of the data. The Center for Evidence-Based Healthcare (ZEGV) Dresden analyzed the anonymized data. The ethics committee of the TU Dresden approved the study protocol. The study protocol has been officially registered at the institutional review board (IRB): Office for Human Research Protections (OHRP); identification numbers: IRB00001473 and IORG0001076; date of approval: 24/04/2017

Patient and Public Involvement

It was not appropriate to involve patients or the public in the design, or conduct, or reporting, or dissemination plans of our research. This cross-sectional analysis uses observational routine data based on predefined outcomes and covariates without intervention.

Population

232 hospitals participated in the IMPRESS study (Figure 1). We included all cases with a colon and/ or rectum resection in the participating hospitals in 2016-2018. Due to anonymization, the data do not contain a patient-relation. Therefore patients admitted more than one time and entered the analysis as multiple hospital cases. For each hospital case, all of the documented information in terms of diagnoses and medical/ surgical procedures during hospitalization was available. We used the definitions of the German Inpatient Quality Indicators (GIQI) to define and distinguish partial colon resections (supplement S2), total colon resections

For peer review only - http://bmjopen.bmj.com/site/about/guidelines.xhtml

(supplement S3) and rectum resections (supplement S4).[21] Hospitals without colon or rectum resections were excluded from the analysis.

Outcomes and covariates

Following evidence from a previous study, in-hospital death, respiratory failure, renal failure and wound-infection can be validly operationalized in hospital discharge data.[15] Hence, we analyzed these outcomes in accordance with previously tested case definitions as presented in supplement S5. The outcomes death, respiratory failure, renal failure, and wound infections served as dependent variables.

The independent variables were classified into three groups:

1) case (age, sex, Elixhauser comorbidities)[19 20],
2) care (admission date, surgical procedures/OPS codes, reason for admission, discharge destination) and
3) hospital (ownership, university hospital status, urbanization degree rural/urban).

This study focused on care and hospital variables. Patient variables were primarily used for adjustment.

To adjust for all potentially relevant risk factors available in the data, the estimations included the full set of independent case, care, and hospital variables. At the case level, this included age, gender and Elixhauser comorbidities.[20] At the care level, admission reason (referral/ emergency case/ transfer from other hospital), weekend surgery (identifiable via time stamp of the procedure), and total resection of the colon and resections of the colon and rectum were used. At the hospital level, hospital's case volume, the urbanization of the hospital (rural/ urban), university hospital status, and ownership (public/ non-profit/ private) were analyzed. Case volume entered the regression models in logarithmic form. This transformation of hospital volume data captures that volume-outcome relationships may be more pronounced at low case volumes.[22]

For peer review only

Statistical methods

We described case, care, and hospital characteristics using absolute and relative frequencies in case of categorical variables. For continuous variables, we reported median and 1st and 3rd quartile. Relationships between patient outcomes and case-level, care-level, and hospital-level risk factors were estimated using multilevel logistic regression models. These models contained a random intercept at the hospital level to capture correlation of patient outcomes within hospitals.[23] Estimations were conducted for cases with colon and rectum resection separately in bivariate and multivariate analyses. To foster interpretability of estimated effect sizes, we transformed the estimated regression coefficients into odds ratios. An estimated effect was

For peer review only - http://bmjopen.bmj.com/site/about/guidelines.xhtml

considered as statistically significant if its p-value was below 5%. Statistical analysis was performed using Stata 15.1.

Sensitivity analysis

In the preliminary research of this thesis it became apparent that the literature distinguishes between colorectal cancer surgery and general colorectal resections.[1 12 13 24 25] Therefore, with respect to possible effect modifications, we explored differences between cases with and without a cancer diagnosis. The same applies to reported interactions between admission reason and the status of university hospitals compared to non-university hospitals.[17] Therefore we also reviewed these interactions to prevent possible effect modifications.

Results

Overall, 71,060 cases with colon and/ or rectum resection were included in the analysis. Separating colon resections and rectum resections a total of 54,168 colon resection cases were treated in 209 hospitals. 20,395 cases of rectum resections were treated in 200 hospitals. If both colon and rectum resection were documented (3,503 cases), the case was analyzed for both groups.

>>>Figure 1 – Flowchart of hospitals included for analysis<<<

The minority of included cases received combined colorectal resections (partial and total colon resection, colon and rectum resections). Emergency case admission or transfer from other hospitals were less frequent than referral. Compared to rectum resections, colon resections were more often surgically treated on weekends (8.6% vs. 3.8%), admitted as an emergency case (29.7% vs. 18.3%) or transferred from other hospitals (3.5% vs. 1.9%). The same applies to the rate of poor patient safety outcomes. Colon resection cases revealed higher rates of in-hospital death (9.6% vs. 4.2%), post-operative respiratory failure (16.7% vs. 12.2%), renal failure (15.2% vs. 10.3%) and post-operative wound infections (11.3% vs. 11.2%), than rectum resection cases. The majority of the analyzed hospitals were localized in urban regions (59%). Most of the analyzed hospitals were in private (40%) or public (40%) ownership. The annual median hospital volume was 72 cases for colon resection (Q1 =38; Q3=116) and 26 cases for rectum resections (Q1=11; Q3=42). The median age ranged from 67-68 years in cases with colon and rectum resections (Table 1). Male

For peer review only - http://bmjopen.bmj.com/site/about/guidelines.xhtml

For peer review only

and female sex in colon and rectum resections were approximately equally represented. Descriptive results for the Elixhauser variables are presented in supplement S6.

For peer review only

For peer review only - http://bmjopen.bmj.com/site/about/guidelines.xhtml

Table 1 – Case and hospital characteristics of colon and rectum resections

	COLON RESECTION		RECTUM RESECTION	
	n	% / Q1; Q3	n	% / Q1; Q3
number of cases	54,168	(100.0%)	20,395	(100.0%)
**PATIENT SAFETY OUTCOMES**				
in-hospital death				
no	48,914	(90.31 %)	19,525	(95.73 %)
yes	5,254	(9.68 %)	870	(4.26 %)
post-operative respiratory failure				
no	45,074	(83.21 %)	17,901	(87.77 %)
yes	9,094	(16.78 %)	1,494	(12.22 %)
renal failure				
no	45,920	(84.77 %)	18,279	(89.62 %)
yes	8,248	(15.22 %)	2,116	(10.37 %)
post-operative wound infections				
no	48,013	(88.63 %)	18,109	(88.79 %)
yes	6,155	(11.36 %)	2,286	(11.20 %)
**CARE CHARACTERISTICS**				
colon resection				
total	2,662	(4.91 %)	-	-
partial	51,310	(94.72 %)	-	-
both	196	(0.36 %)	-	-
rectum resection				
no	50,665	(93.53 %)	-	-
yes	3,503	(6.46 %)	20,395	(100.00 %)
colon and rectum resection				
no	50,665	(93.53 %)	16,892	(82.82 %)
yes	3,503	(6.46 %)	3,503	(17.17 %)
weekend surgery				
no	49,473	(91.33 %)	19,603	(96.11 %)
yes	4,695	(8.66 %)	792	(3.88 %)
admission reason				
referral	36,129	(66.69 %)	16,249	(79.67 %)
emergency case	16,116	(29.75 %)	3,744	(18.35 %)
transfer from other hospital	1,923	(3.55 %)	402	(1.97 %)
**HOSPITAL CHARACTERISTICS**				
hospitals included	209	(100.00%)	200	(100.00%)
annual volume				
colon resection cases (median)	72	(38; 119)	-	-
total colon resection (median)	1	(0;3)	-	-
rectum resections (median)	-	-	26	(11;42)
urbanization				
urban	124	(59.33 %)	119	(59.50 %)
rural	85	(40.66 %)	81	(40.50 %)

For peer review only - http://bmjopen.bmj.com/site/about/guidelines.xhtml

ownership				
public	82	(39.23 %)	80	(40.00 %)
non-profit	41	(19.61 %)	39	(19.50 %)
private	86	(41.14 %)	81	(40.50 %)
university hospital				
no	201	(96.17 %)	192	(96.00 %)
yes	8	(3.82 %)	8	(4.00 %)
**CASE CHARACTERISTICS**				
Age				
median	68	(56; 77)	67	(57; 77)
sex				
male	26,954	(49.76 %)	10,367	(50.83 %)
female	27,214	(50.23 %)	10,028	(49.16 %)
Elixhauser comorbidities […][a]				

Legend: Q1: 1st Quartile Q3: 3rd quartile
[a] results of elixhauser comorbidities (e.g. alcohol abuse, blood loss anemia, cardiac arrhythmias, …) are presented in supplement S6

The bivariate analysis provided in supplements S7-S8 was performed to identify unadjusted effects of single covariates on the outcomes. The following multivariable analysis, focusing on care and hospital level (Tables 2-3), was performed to achieve adjusted and statistically robust effects of the covariates.

<u>Care covariates</u>

Admission as an emergency case or transfer from other hospital were significantly associated multiple poor patient safety outcomes for all of the dependent variables in both colon resections and rectum resections. For example the odds of in-hospital death were significantly higher in emergency cases in colon resections (OR 1.84 (95% CI, 1.69-2.01) and rectum resections (OR 2.02 (95% CI, 1.67-2.45) than referral hospital admissions. The same applies to admission as transfer from other hospital. The odds of in-hospital death were significantly higher in colon resections (OR 2.52 (95% CI, 2.19-2.91)) and rectum resections (OR 2.67 (95% CI, 1.87-3.82)). With exception of post-operative wound infections, weekend surgery was significantly associated with a worsening patient safety outcome in colon resections and rectum resections as well.

<u>Hospital covariates</u>

While most of the care-level covariates showed similar effect directions in both case groups, the hospital covariates showed insignificant or heterogeneous effects between colon and rectum resections.

The coefficients on annual case volume of colon resections indicated a significantly higher risk of post-operative wound infections (OR 1.16 (95% CI, 1.03-1.32)) in hospital with higher volumes. The remaining associations between annual case volume of colon resection cases and the patient safety outcomes in-hospital

For peer review only - http://bmjopen.bmj.com/site/about/guidelines.xhtml

death, post-operative respiratory failure and renal failure remained insignificant. A higher annual volume of rectum resection cases was significantly associated with lower risks of in-hospital death (OR 0.70 (95% CI, 0.61-0.80)), post-operative respiratory failure (OR 0.84 (95% CI, 0.72-0.98)) and renal failure (OR 0.85 (95% CI, 0.76-0.95)).

The rural localization showed significantly lower odds of renal failure (OR 0.77 (95% CI, 0.63-0.93)) in patients with colon resections. The associations to the remaining patient safety outcomes in both colon and rectum resections remained insignificant.

Treatment in university hospitals was associated with increased odds of post-operative wound infections in both colon resections (OR 1.98 (95% CI, 1.17-3.35)) and rectum resections (OR 2.29 (95% CI, 1.35-3.86)).

The hospital ownership revealed differences between as well as colon and rectum resections and the patient safety outcomes.

Non-profit (OR, 0.74 (95% CI, 0.55-0.99)) or private (OR 0.77, (95% CI, 0.60-0.99)) ownership was associated with lower risks of post-operative wound infections in colon resections. In contrast, the odds of in-hospital death (OR 1.24, (95% CI, 1.02-1.50)) and renal failure (OR 1.93, (95% CI, 1.56-2.40)) in colon resections were higher in private hospitals. Rectum resections did not show significant associations of ownership and patient safety outcomes except for higher odds of renal failure (OR 1.59, (95% CI, 1.25-2.03)) in private hospitals.

Case covariates

Female sex was consistently associated with better outcomes in both cases with colon and rectum resection, with the exception of an insignificant association with in-hospital death(OR 0.93, (95% CI, 0.87-1.00)) in colon resections. Higher age was significantly associated with higher risks of in-hospital death, post-operative respiratory failure and renal failure in both colon and rectum resections. Regarding post-operative wound infections, age was a borderline-insignificant protective factor in colon (OR 0.99, (95% CI, 0.99-1.00)) and rectum (OR 0.99, (95% CI, 0.99-1.00)) resections. The multivariate results for the Elixhauser groups included in the model can be found in supplements S9-S10. We reviewed differences in results of stratified analyses for cases with and without a cancer diagnosis of colon and rectum resections. Significant effect reversals between these groups were not given. For detailed results of stratified analyses, please refer to supplements S11-S14. The review also did not reveal differences between university and non-university hospitals in terms of emergency admission or transfer from other hospital (supplements S15-S16). Therefore, a stratification between cases with and without cancer or university and non-university hospitals has not been applied

For peer review only

For peer review only - http://bmjopen.bmj.com/site/about/guidelines.xhtml

Table 2 – Multivariate analysis of patient safety outcomes in 54,168 colon resections in 209 hospitals

	IN-HOSPITAL DEATH		POST-OPERATIVE RESPIRATORY FAILURE		RENAL FAILURE		POST-OPERATIVE WOUND INFECTION	
	OR	95% CI	OR	95% CI	OR	95% CI	OR	95% CI
**CARE COVARIATES**								
admission reason								
referral	Ref.		Ref.		Ref.		Ref.	
emergency case	1.847***	(1.692 - 2.015)	1.413***	(1.320 - 1.513)	1.453***	(1.349 - 1.566)	1.145***	(1.067 - 1.228)
transfer from other hospital	2.528***	(2.193 - 2.915)	1.982***	(1.749 - 2.245)	1.908***	(1.678 - 2.171)	1.223**	(1.071 - 1.397)
weekend surgery								
no	Ref.		Ref.		Ref.		Ref.	
yes	1.669***	(1.515 - 1.839)	1.426***	(1.312 - 1.550)	1.480***	(1.360 - 1.610)	1.080	(0.984 - 1.186)
total colon resection								
no	Ref.		Ref.		Ref.		Ref.	
yes	2.679***	(2.369 - 3.029)	1.639***	(1.472 - 1.825)	2.228***	(1.999 - 2.483)	1.022	(0.913 - 1.143)
colon and rectum resection								
no	Ref.		Ref.		Ref.		Ref.	
yes	1.103	(0.960 - 1.267)	1.524***	(1.378 - 1.686)	1.408***	(1.265 - 1.567)	1.579***	(1.426 - 1.748)
**HOSPITAL COVARIATES**								
case volume	0.968	(0.871 - 1.076)	0.919	(0.807 - 1.047)	0.992	(0.891 - 1.106)	1.168*	(1.030 - 1.325)
area								
urban	Ref.		Ref.		Ref.		Ref.	
rural	1.061	(0.893 - 1.261)	0.863	(0.648 - 1.149)	0.772**	(0.635 - 0.939)	1.032	(0.824 - 1.292)
university hospital								
no	Ref.		Ref.		Ref.		Ref.	
yes	1.303	(0.888 - 1.912)	0.687	(0.338 - 1.397)	1.412	(0.889 - 2.241)	1.981*	(1.171 - 3.352)
ownership								
public	Ref.		Ref.		Ref.		Ref.	
non-profit	1.012	(0.811 - 1.262)	1.057	(0.726 - 1.540)	0.867	(0.670 - 1.122)	0.744*	(0.555 - 0.998)
private	1.244*	(1.026 - 1.507)	1.329	(0.972 - 1.817)	1.937***	(1.563 - 2.400)	0.777*	(0.608 - 0.992)
**CASE COVARIATES**								
sex								
male	Ref.		Ref		Ref		Ref	
female	0.937	(0.873 - 1.006)	0.788***	(0.745 - 0.833)	0.683***	(0.645 - 0.725)	0.882***	(0.832 - 0.936)
age	1.050***	(1.046 - 1.053)	1.014***	(1.012 - 1.017)	1.024***	(1.021 - 1.026)	0.998	(0.996 - 1.000)
Elixhauser comorbidities […][a]								

Legend:
*** p<0.001
** p <0.01
* p<0.05
[a] results of elixhauser comorbidities (e.g. alcohol abuse, blood loss anemia, cardiac arrhythmias, chronic pulmonary disease …) are presented in supplement S9

For peer review only - http://bmjopen.bmj.com/site/about/guidelines.xhtml

Table 3 – Multivariate analysis of patient safety outcomes in 20,395 rectum resections in 200 hospitals

	**IN-HOSPITAL DEATH**		**POST-OPERATIVE RESPIRATORY FAILURE**		**RENAL FAILURE**		**POST-OPERATIVE WOUND INFECTION**	
	OR	95% CI	OR	95% CI	OR	95% CI	OR	95% CI
**CARE COVARIATES**								
admission reason								
referral	Ref.		Ref.		Ref.		Ref.	
emergency case	2.028***	(1.675 - 2.454)	1.335***	(1.170 - 1.523)	1.342***	(1.169 - 1.540)	1.291***	(1.138 - 1.466)
transfer from other hospital	2.679***	(1.874 - 3.828)	1.859***	(1.406 - 2.459)	1.927***	(1.461 - 2.541)	1.484**	(1.131 - 1.948)
weekend surgery								
no	Ref.		Ref.		Ref.		Ref.	
yes	1.960***	(1.483 - 2.591)	1.427**	(1.150 - 1.770)	1.391**	(1.127 - 1.717)	0.985	(0.784 - 1.238)
total colon resection								
no	Ref.		Ref.		Ref.		Ref.	
yes	2.579***	(2.163 - 3.074)	2.164***	(1.921 - 2.438)	1.859***	(1.645 - 2.100)	1.522***	(1.356 - 1.708)
**HOSPITAL COVARIATES**								
case volume	0.703***	(0.611 - 0.809)	0.844*	(0.725 - 0.982)	0.853**	(0.760 - 0.958)	0.973	(0.854 - 1.109)
area								
urban	Ref.		Ref.		Ref.		Ref.	
rural	1.072	(0.817 - 1.407)	0.904	(0.639 - 1.281)	0.834	(0.670 - 1.037)	0.854	(0.663 - 1.101)
university hospital								
no	Ref.		Ref.		Ref.		Ref.	
yes	1.616	(0.979 - 2.665)	0.853	(0.379 - 1.920)	1.299	(0.829 - 2.037)	2.292**	(1.358 - 3.869)
ownership								
public	Ref.		Ref.		Ref.		Ref.	
non-profit	0.851	(0.614 - 1.179)	1.059	(0.674 - 1.664)	0.762	(0.576 - 1.008)	0.761	(0.553 - 1.048)
private	0.925	(0.682 - 1.254)	1.267	(0.865 - 1.858)	1.597***	(1.256 - 2.030)	0.846	(0.642 - 1.117)
**CASE COVARIATES**								
sex								
male	Ref.		Ref		Ref		Ref	
female	0.842*	(0.710 - 0.998)	0.842**	(0.759 - 0.934)	0.685***	(0.613 - 0.765)	0.826***	(0.748 - 0.912)
age	1.068***	(1.059 - 1.078)	1.014***	(1.009 - 1.018)	1.021***	(1.016 - 1.026)	0.998	(0.995 - 1.002)
Elixhauser comorbidities […][a]								

Legend:
*** p<0.001
** p <0.01
* p<0.05
[a] results of elixhauser comorbidities (e.g. alcohol abuse, blood loss anemia, cardiac arrhythmias, chronic pulmonary disease …) are presented in supplement S10

For peer review only - http://bmjopen.bmj.com/site/about/guidelines.xhtml

Discussion

In this large observational analysis of 54,168 colon resection cases and 20,395 rectum resection cases we present new and comprehensive findings for patient safety and quality assurance.

Several care-level covariates were significant risk factors for multiple patient safety outcomes in both procedure groups. Preoperative transfer from other hospitals and emergency admission were crucial risk factors for poor patient outcomes in cases with colon and rectum resections. Regarding emergency admission, our findings confirm the recent literature which also showed associations between case-urgency and 30-day-mortality, 5-year survival and morbidities (composite outcome).[7 8] Transfer from other hospitals our findings also do confirm the recent literature as a risk factor for poor patient outcome. The risks of death, complications or extended length of stay were significantly increased in urgent and non-urgent transfers from other hospitals.[9-11] Weekend surgery was associated with higher risks for death, post-operative respiratory and renal failure in both procedure groups either. These findings confirm the literature with regard to mortality in colon[4] and general surgery.[5 6] With regard to rectum resections, the literature reported insignificant effects. These are most likely explained by a small number of included cases.[4] Our findings underline the need for a consideration of care contexts in risk-adjusted quality assurance.

With respect to hospital covariates, this analysis showed conflicting effects. Inconclusive results were found for rural localization, university hospital status and hospital ownership. The estimated effects were insignificant (rural hospitals) or conflicting (volume, ownership) and therefore did not affect the considered patient safety outcomes overwhelmingly. The literature discusses different effects of annual case volume,[12 13 26] rural hospitals,[27-29] ownership[30] or university hospital status[31] with confirming or contradicting results as well. In summary, hospital-level covariates did not show consistent effects on patient safety outcomes in colon resections or rectum resections. This may be due to information not covered by the hospital discharge data. For example staffing,[32 33] expertise[34] or certification[35] may also influence patient outcome. Additionally, some studies did not stratify colon and rectum resections.[36 37] However, the heterogeneity of results for both procedure groups revealed in our study, especially regarding relationships with hospital covariates, indicates the relevance of stratification of colon and rectum resections. The need for stratification instead of adjustment has also been reported for other indications.[17]

With respect to case covariates age, sex and comorbidities were risk factors for poor patient outcome in both patient groups in this analysis and literature too.[24 38]

This study analyzed a large and current sample providing a broad span of cases, hospital types, ownerships, and locations. While previous studies emphasized specific covariates and/or outcomes we considered

For peer review only - http://bmjopen.bmj.com/site/about/guidelines.xhtml

combined sets of previously solitary analyzed outcomes and risk factors and, thus, provided a comprehensive analysis of a rich dataset. Secondary data induce challenges for a reliable operationalization of outcomes. First, the data are anonymized and the anonymization leads into the impossibility to validate the coded diagnoses.[39] Second, accounting data do not include information which of the coded diagnoses had been present on admission. To overcome these shortcomings, this study used a set of previously validated outcomes that were reported to occur most likely during hospitalization.[15] With respect to transfer from other hospital, the recent literature distinguishes between urgent and non-urgent inter-hospital transfers.[9-11] The data included in this analysis do not include details of the reasons for transfer from other hospital. However, our results were adjusted for age, sex, comorbidities and weekend surgery which serve as a proxy for case severity and complexity. The different results depending on adjustment, stratification, bi- and multivariate analyses considered and discussed in this work also underline the need for careful and comprehensive statistical analysis. One weakness of German hospital discharge data is a lack of information on patient history, medication, length of anesthesia, staff-to-patient ratios, surgeon volumes, centralization and comorbidities present on admission.[33 35 40] This lacking information may lead to bias as these covariates may influence the outcome and could not be considered in our study.

Conclusions

This study demonstrated that patient safety in colorectal resections is strongly related to multiple care covariates. In particular, our results implicate that it is necessary to account for admission reasons and weekend surgery when measuring and comparing patient safety outcomes between hospitals. Hence, they should generally be considered as covariates in empirical analyses of patient safety. Therefore a risk adjustment for these covariates in quality assurance measures of colorectal resections also should follow. Hospital volume, ownership, urbanization degree and university hospital status could not be shown as overwhelmingly and conclusive covariates of outcomes in patients with colon or rectum resections. Given these insights from analysis of a large dataset, this paper contributes reliable and comprehensive evidence to the ongoing debate on multiple hospital- and care-related influence factors on patient safety in general.

For peer review only - http://bmjopen.bmj.com/site/about/guidelines.xhtml

Funding: The IMPRESS study is funded by the Innovation Fund of the Joint Federal Committee (Gemeinsamer Bundesausschuss, G-BA), Germany. Funding number: 01VSF16013. Open Access Funding by the Publication Fund of the TU Dresden.

Author contributions:

FW designed the concept, methods and investigation, visualized results and wrote the draft of the underlying analysis. MR undertook the formal analysis. MR and OS curated the data, supervised the methodology and project administration in general and revised the drafts. JS, MEG, PS and RK acquired funding, supervised the concept and investigation and revised the drafts.

Competing interests: Peter C. Scriba and Ralf Kuhlen are members of the scientific advisory board of IQM. Maria Eberlein-Gonska serves as an external expert for IQM. The other authors declare that they have no conflict of interest.

Data availability statement: The data are not publicly available due containing information that could compromise research participant privacy/consent. The consent given from the research participants included the obligation to analyze the data anonymously without disclosure to other parties.

For peer review only

References

1. O'Brien BS, McNally MP, Duncan JE. Controversies surrounding quality measurement in colon and rectal surgery. *Clin Colon Rectal Surg* 2014;27(1):26-31. doi: 10.1055/s-0034-1366916 [published Online First: 2014/03/04]
2. Goodacre S, Campbell M, Carter A. What do hospital mortality rates tell us about quality of care? *Emerg Med J* 2015;32(3):244-7. doi: 10.1136/emermed-2013-203022 [published Online First: 2013/09/26]
3. Lilford R, Pronovost P. Using hospital mortality rates to judge hospital performance: a bad idea that just won't go away. *Bmj* 2010;340:c2016. doi: 10.1136/bmj.c2016 [published Online First: 2010/04/22]
4. Huijts DD, van Groningen JT, Guicherit OR, et al. Weekend Effect in Emergency Colon and Rectal Cancer Surgery: A Prospective Study Using Data From the Dutch ColoRectal Audit. *J Natl Compr Canc Netw* 2018;16(6):735-41. doi: 10.6004/jnccn.2018.7016 [published Online First: 2018/06/13]
5. McCallum IJ, McLean RC, Dixon S, et al. Retrospective analysis of 30-day mortality for emergency general surgery admissions evaluating the weekend effect. *Br J Surg* 2016;103(11):1557-65. doi: 10.1002/bjs.10261 [published Online First: 2016/08/16]
6. O'Leary JD, Wunsch H, Leo AM, et al. Hospital admission on weekends for patients who have surgery and 30-day mortality in Ontario, Canada: A matched cohort study. *PLoS Med* 2019;16(1):e1002731. doi: 10.1371/journal.pmed.1002731 [published Online First: 2019/01/30]
7. Anderson JH, Hole D, McArdle CS. Elective versus emergency surgery for patients with colorectal cancer. *Br J Surg* 1992;79(7):706-9. doi: 10.1002/bjs.1800790739 [published Online First: 1992/07/01]
8. Mullen MG, Michaels AD, Mehaffey JH, et al. Risk Associated With Complications and Mortality After Urgent Surgery vs Elective and Emergency Surgery: Implications for Defining “Quality” and Reporting Outcomes for Urgent Surgery. *JAMA Surgery* 2017;152(8):768-74. doi: 10.1001/jamasurg.2017.0918
9. Chow CJ, Gaertner WB, Jensen CC, et al. Does Hospital Transfer Impact Outcomes After Colorectal Surgery? *Diseases of the Colon & Rectum* 2017;60(2):194-201. doi: 10.1097/dcr.0000000000000765
10. Sharp SP, Ata A, Valerian BT, et al. Complications and surgical outcomes after interhospital transfer vs direct admission in colorectal surgery: A National Surgical Quality Improvement Program analysis. *Am J Surg* 2017;213(6):1031-37. doi: 10.1016/j.amjsurg.2016.08.013 [published Online First: 2016/10/25]
11. Sharp SP, Schuster DJ, Ata A, et al. Impact of Interhospital Transfer on Outcomes in Non-emergency Colorectal Surgery. *World J Surg* 2018;42(5):1542-50. doi: 10.1007/s00268-017-4313-z [published Online First: 2017/10/29]
12. Chioreso C, Del Vecchio N, Schweizer ML, et al. Association Between Hospital and Surgeon Volume and Rectal Cancer Surgery Outcomes in Patients With Rectal Cancer Treated Since 2000: Systematic Literature Review and Meta-analysis. *Dis Colon Rectum* 2018;61(11):1320-32. doi: 10.1097/dcr.0000000000001198 [published Online First: 2018/10/05]
13. Huo YR, Phan K, Morris DL, et al. Systematic review and a meta-analysis of hospital and surgeon volume/outcome relationships in colorectal cancer surgery. *J Gastrointest Oncol* 2017;8(3):534-46. doi: 10.21037/jgo.2017.01.25 [published Online First: 2017/07/25]
14. Almoudaris AM, Burns EM, Bottle A, et al. Single measures of performance do not reflect overall institutional quality in colorectal cancer surgery. *Gut* 2013;62(3):423. doi: 10.1136/gutjnl-2011-301489
15. Maass C, Kuske S, Lessing C, et al. Are administrative data valid when measuring patient safety in hospitals? A comparison of data collection methods using a chart review and administrative data. *Int J Qual Health Care* 2015;27(4):305-13. doi: 10.1093/intqhc/mzv045 [published Online First: 2015/07/03]
16. Schmitt J, Schoffer O, Walther F, et al. Effectiveness of the IQM peer review procedure to improve in-patient care—a pragmatic cluster randomized controlled trial (IMPRESS):

For peer review only - http://bmjopen.bmj.com/site/about/guidelines.xhtml

study design and baseline results. *Journal of Public Health* 2019 doi: 10.1007/s10389-019-01118-9
17. Schoffer O, Roessler M, Walther F, et al. Patient-Level and Hospital-Level Risk Factors for In-Hospital Mortality in Patients Ventilated for More Than 24 Hours: Results of a Nationwide Cohort Study. *Journal of Intensive Care Medicine* 2020;0(0):0885066620942182. doi: 10.1177/0885066620942182
18. Walther F, Schoffer O, Schmitt J. Effectiveness of a collegial consultation procedure to improve in-patient care—a pragmatic cluster randomized controlled trial (ISRCTN10188560): ISRCTN; 2018 [Available from: http://www.isrctn.com/ISRCTN10188560 accessed 01/09/ 2020.
19. Elixhauser A, Steiner C, Harris DR, et al. Comorbidity measures for use with administrative data. *Med Care* 1998;36(1):8-27. doi: 10.1097/00005650-199801000-00004 [published Online First: 1998/02/07]
20. Quan H, Sundararajan V, Halfon P, et al. Coding algorithms for defining comorbidities in ICD-9-CM and ICD-10 administrative data. *Med Care* 2005;43(11):1130-9. doi: 10.1097/01.mlr.0000182534.19832.83 [published Online First: 2005/10/15]
21. Mansky T, Nimptsch U, Cools A, et al. G-IQI – German Inpatient Quality Indicators. Version 5.1 – Band 1. Berlin: Universitätsverlag der TU Berlin 2017.
22. Hentschker C, Mennicken R. The Volume-Outcome Relationship Revisited: Practice Indeed Makes Perfect. *Health Serv Res* 2018;53(1):15-34. doi: 10.1111/1475-6773.12696 [published Online First: 2017/09/05]
23. Snijders TAB, Bosker RJ. Multilevel analysis : an introduction to basic and advanced multilevel modeling. 2nd ed. ed: Los Angeles ; London : SAGE 2012.
24. Byrne BE, Mamidanna R, Vincent CA, et al. Population-based cohort study comparing 30- and 90-day institutional mortality rates after colorectal surgery. *Br J Surg* 2013;100(13):1810-7. doi: 10.1002/bjs.9318 [published Online First: 2013/11/15]
25. Sheikh L, Croft R, Harmston C. Counting the costs of complications in colorectal surgery. *N Z Med J* 2019;132(1497):32-36. [published Online First: 2019/06/21]
26. Link KH, Coy P, Roitman M, et al. Minimum Volume Discussion in the Treatment of Colon and Rectal Cancer: A Review of the Current Status and Relevance of Surgeon and Hospital Volume regarding Result Quality and the Impact on Health Economics. *Visceral Medicine* 2017;33(2):140-47. doi: 10.1159/000456044
27. Hamidi M, Hanna K, Omesiete P, et al. Does it matter where you get your surgery for colorectal cancer? *International Journal of Colorectal Disease* 2019;34(12):2121-27. doi: 10.1007/s00384-019-03436-6
28. Knight M. The Effect of Hospital Organizational Characteristics on Postoperative Complications. *Journal of Patient Safety* 2013;9(4):198-202. doi: 10.1097/PTS.0b013e3182995e5b
29. Pandit V, Jehan F, Zeeshan M, et al. Failure to Rescue in Postoperative Patients With Colon Cancer: Time to Rethink Where You Get Surgery. *Journal of Surgical Research* 2019;234:1-6. doi: https://doi.org/10.1016/j.jss.2018.08.046
30. Morris M, Iacopetta B, Platell C. Comparing survival outcomes for patients with colorectal cancer treated in public and private hospitals. *Med J Aust* 2007;186(6):296-300. [published Online First: 2007/03/21]
31. Juillard C, Lashoher A, Sewell CA, et al. A National Analysis of the Relationship Between Hospital Volume, Academic Center Status, and Surgical Outcomes for Abdominal Hysterectomy Done for Leiomyoma. *Journal of the American College of Surgeons* 2009;208(4):599-606. doi: https://doi.org/10.1016/j.jamcollsurg.2009.01.003
32. Etzioni DA, Young-Fadok TM, Cima RR, et al. Patient survival after surgical treatment of rectal cancer: impact of surgeon and hospital characteristics. *Cancer* 2014;120(16):2472-81. doi: 10.1002/cncr.28746 [published Online First: 2014/05/08]
33. Yasunaga H, Hashimoto H, Horiguchi H, et al. Variation in cancer surgical outcomes associated with physician and nurse staffing: a retrospective observational study using the Japanese Diagnosis Procedure Combination Database. *BMC Health Services Research* 2012;12(1):129. doi: 10.1186/1472-6963-12-129

For peer review only - http://bmjopen.bmj.com/site/about/guidelines.xhtml

34. Hall GM, Shanmugan S, Bleier JI, et al. Colorectal specialization and survival in colorectal cancer. *Colorectal Dis* 2016;18(2):O51-60. doi: 10.1111/codi.13246 [published Online First: 2015/12/29]
35. Trautmann F, Reißfelder C, Pecqueux M, et al. Evidence-based quality standards improve prognosis in colon cancer care. *European Journal of Surgical Oncology* 2018;44(9):1324-30. doi: https://doi.org/10.1016/j.ejso.2018.05.013
36. Kolfschoten NE, Marang-van de Mheen PJ, Wouters MWJM, et al. A Combined Measure of Procedural Volume and Outcome to Assess Hospital Quality of Colorectal Cancer Surgery, a Secondary Analysis of Clinical Audit Data. *PLOS ONE* 2014;9(2):e88737. doi: 10.1371/journal.pone.0088737
37. Liu CJ, Chou YJ, Teng CJ, et al. Association of surgeon volume and hospital volume with the outcome of patients receiving definitive surgery for colorectal cancer: A nationwide population-based study. *Cancer* 2015;121(16):2782-90. doi: 10.1002/cncr.29356 [published Online First: 2015/04/22]
38. Mamidanna R, Burns EM, Bottle A, et al. Reduced Risk of Medical Morbidity and Mortality in Patients Selected for Laparoscopic Colorectal Resection in England: A Population-Based Study. *Archives of Surgery* 2012;147(3):219-27. doi: 10.1001/archsurg.2011.311
39. Ghadban T, Reeh M, Bockhorn M, et al. Decentralized colorectal cancer care in Germany over the last decade is associated with high in-hospital morbidity and mortality. *Cancer Manag Res* 2019;11:2101-07. doi: 10.2147/cmar.S197865 [published Online First: 2019/03/19]
40. Renzulli P, Lowy A, Maibach R, et al. The influence of the surgeon's and the hospital's caseload on survival and local recurrence after colorectal cancer surgery. *Surgery* 2006;139(3):296-304. doi: https://doi.org/10.1016/j.surg.2005.08.023

For peer review only - http://bmjopen.bmj.com/site/about/guidelines.xhtml

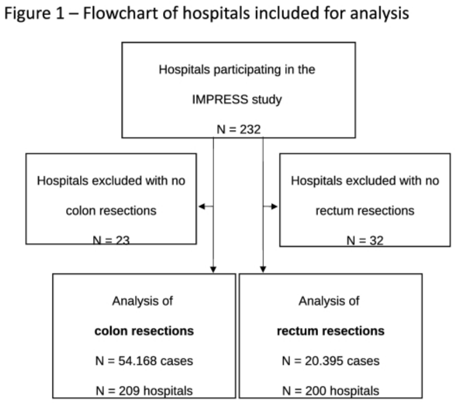

Figure 1 - Flowchart of hospitals included for analysis

338x190mm (300 x 300 DPI)

S1: ICD-10 Codes of Elixhauser Comorbidity Groups[20]

Congestive heart failure: I099, I110, I130, I132, I255, I420, I425, I426, I427, I428, I429, I43, I50, P290

Cardiac arrhythmias: I441, I442, I443, I456, I459, I47, I48, I49, R000, R001, R008, T821, Z450, Z950

Valvular disease : A520, I05, I06, I07, I08, I091, I098, I34, I35, I36, I37, I38, I39, Q230, Q231, Q232, Q233, Z952, Z953 Z954

Pulmonary circulation Disorders: I26, I27, I280, I288, I289

Peripheral vascular disorders: I70, I71, I731, I738, I739, I771, I790, I792, K551, K558, K559, Z958, Z959

Hypertension, uncomplicated: I10

Hypertension, complicated: I11, I12, I13, I15

Paralysis: G041, G114, G801, G802, G81, G82, G830, G831, G832, G833, G834, G839

Other neurological disorders: G10, G11, G12, G13, G20, G21, G22, G254, G255, G312, G318, G319, G32, G35, G36, G37, G40, G41, G931, G934, R470, R56

Chronic pulmonary disease: I278, I279, J40, J41, J42, J43, J44, J45, J46, J47, J60, J61, J62, J63, J64, J65, J66, J67, J684, J701, J703

Diabetes, uncomplicated: E100, E101, E109, E110, E111, E119, E120, E121, E129, E130, E131, E139, E140, E141, E149

Diabetes, complicated: E102, E103, E104, E105, E106, E107, E108, E112, E113, E114, E115, E116, E117, E118, E122, E123, E124, E125, E126, E127, E128, E132, E133, E134, E135, E136, E137, E138, E142, E143, E144, E145, E146, E147, E148

Hypothyroidism: E00, E01, E02, E03, E890

Renal failure: I120, I131, N18, N19, N250, Z490, Z491, Z492, Z940, Z992

Peptic ulcer disease excluding bleeding: K257, K259, K267, K269, K277, K279, K287, K289

AIDS/HIV: B20, B21, B22, B24

Lymphoma: C81, C82, C83, C84, C85, C88, C96, C900, C902

Metastatic cancer: C77, C78, C79, C80

Solid tumor without metastasis: C00, C01, C02, C03, C04, C05, C06, C07, C08, C09, C1 0, C11, C12, C13, C14, C15, C16, C17, C18, C19, C20, C21, C22, C23, C24, C25, C26, C30, C31, C32, C33, C34, C37, C38, C39, C40, C41, C43, C45, C46, C47, C48, C49, C50, C51, C52, C53, C54, C55, C56, C57, C58, C60, C61, C62, C63, C64, C65, C66, C67, C68, C69, C70, C71, C72, C73, C74, C75, C76, C97

Rheumatoid arthritis/collagen vascular diseases: L940, L941, L943, M05, M06, M08, M120, M123, M30, M310, M311, M312, M313, M32, M33, M34, M35, M45, M461, M468, M469

Coagulopathy: D65, D66, D67, D68, D691, D693, D694, D695, D696

Obesity: E66

Weight loss: E40, E41, E42, E43, E44, E45, E46, R634, R64

Fluid and electrolyte disorders: E222, E86, E87

Blood loss anemia: D500

For peer review only

S2. OPS-codes for partial colon resections according to GIQI[21]

OPS-CODE	TRANSLATION (BY AUTHOR)
5-455.01	Partial resection of the colon: segmental resection: open surgery with anastomosis
5-455.02	Partial resection of the colon: segmental resection: open surgery with enterostoma and blind closure
5-455.03	Partial Resection of the Colon: Segment Resection: Open Surgery with Two Enterostomata
5-455.04	Partial Resection of the Colon: Segment Resection: Open Surgery with Anastomosis Anus praeter
5-455.05	Partial Resection of the Colon: Segmental Resection: Laparoscopic with Anastomosis
5-455.06	Partial Resection of the Colon: Segmental Resection: Laparoscopic with Enterostomy
5-455.07	Partial resection of the colon: segmental resection: change laparoscopic - open surgical
5-455.0X	Partial resection of the colon: Segment resection: Other
5-455.11	Partial resection of the colon: multiple segment resections: Open surgery with anastomosis
5-455.12	Partial resection of the colon: multiple segment resections: Open surgery with enterostoma and blind closure
5-455.13	Partial resection of the colon: multiple segment resections: Open surgery with two enterostomata
5-455.14	Partial resection of the colon: multiple segment resections: Open surgery with anastomosis anus praeter
5-455.15	Partial resection of the colon: multiple segment resections: Laparoscopic with anastomosis
5-455.16	Partial resection of the colon: multiple segment resections: Laparoscopic with enterostomy
5-455.17	Partial resection of the colon: multiple segment resections: Transfer laparoscopic - open surgery
5-455.1X	Partial resection of the colon: multiple segment resections: Other
5-455.21	Partial Resection of the Colon: Ileocecal Resection: Open Surgery with Anastomosis
5-455.22	Partial Resection of the Colon: Ileocecal Resection: Open surgery with enterostoma and blind closure
5-455.23	Partial Resection of the Colon: Ileocecal Resection: Open Surgery with Two Enterostomata
5-455.24	Partial Resection of the Colon: Ileocecal Resection: Open Surgery with Anastomosis Anus praeter
5-455.25	Partial Resection of the Colon: Ileocecal Resection: Laparoscopic with Anastomosis
5-455.26	Partial Resection of the Colon: Ileocecal Resection: Laparoscopic with Enterostoma
5-455.27	Partial resection of the colon: ileocecal resection: change laparoscopic - open surgical
5-455.2X	Partial resection of the colon: Ileocecal resection: Other
5-455.31	Partial resection of the colon: Caecal resection: Open surgery
5-455.35	Partial resection of the colon: Caecal resection: Laparoscopic
5-455.37	Partial resection of the colon: Caecal resection: change laparoscopic - open surgical
5-455.41	Partial resection of the colon: Resection of the ascending colon with coecum and right flexure [hemicolectomy right]: Open surgery with anastomosis
5-455.42	Partial Resection of the Colon: Resection of the ascending colon with coecum and right flexure [Hemicolectomy right]: Open surgery with enterostoma and
5-455.43	Partial Resection of the Colon: Resection of the ascending colon with coecum and right flexure [Hemicolectomy right]: Open surgery with two enterostomata
5-455.44	Partial resection of the colon: Resection of the ascending colon with coecum and right flexure [hemicolectomy right]: Open surgery with anastomosis anus praeter
5-455.45	Partial resection of the colon: Resection of the ascending colon with coecum and right flexure [hemicolectomy right]: Laparoscopic with anastomosis
5-455.46	Partial resection of the colon: Resection of the ascending colon with coecum and right flexure [hemicolectomy right]: Laparoscopically with enterostoma
5-455.47	Partial resection of the colon: resection of the ascending colon with coecum and right flexure [hemicolectomy right]: change laparoscopic - open surgical
5-455.4X	Partial resection of the colon: Resection of the ascending colon with coecum and right flexure [right hemicolectomy]: Other
5-455.51	Partial resection of the colon: Resection of the transverse colon: Open surgery with anastomosis
5-455.52	Partial resection of the colon: Resection of the transverse colon: open surgery with enterostoma and blind closure
5-455.53	Partial Resection of the Colon: Resection of the Transverse Colon: Open Surgery with Two Enterostomata
5-455.54	Partial resection of the colon: resection of the transverse colon: open surgery with anastomosis anus praeter
5-455.55	Partial resection of the colon: Resection of the transverse colon: Laparoscopic with anastomosis
5-455.56	Partial Resection of the Colon: Resection of the Transverse Colon: Laparoscopic with Enterostoma
5-455.57	Partial resection of the colon: resection of the transverse colon: change laparoscopic - open surgical
5-455.5X	Partial resection of the colon: Resection of the transverse colon: Other
5-455.61	Partial resection of the colon: resection of the descending colon with left flexure [hemicolectomy left]: open surgery with anastomosis
5-455.62	Partial resection of the colon: resection of the descending colon with left flexure [hemicolectomy left]: open surgery with enterostoma and blind closure
5-455.63	Partial resection of the colon: resection of the descending colon with left flexure [hemicolectomy left]: open surgery with two enterostomata
5-455.64	Partial resection of the colon: resection of the descending colon with left flexure [hemicolectomy left]: open surgery with anastomosis anus praeter
5-455.65	Partial resection of the colon: Resection of the descending colon with left flexure [Hemicolectomy left]: Laparoscopic with anastomosis
5-455.66	Partial Resection of the Colon: Resection of the descending colon with left flexure [Hemicolectomy left]: Laparoscopic with Enterostoma
5-455.67	Partial resection of the colon: resection of the descending colon with left flexure [hemicolectomy left]: change laparoscopic - open surgical
5-455.6X	Partial resection of the colon: Resection of the descending colon with left flexure [Hemicolectomy left]: Other
5-455.71	Partial resection of the colon: sigmoid resection: open surgery with anastomosis
5-455.72	Partial resection of the colon: sigmoid resection: open surgery with enterostoma and blind closure
5-455.73	Partial resection of the colon: sigmoid resection: open surgery with two enterostomata
5-455.74	Partial resection of the colon: sigmoid resection: open surgery with anastomosis anus praeter

For peer review only - http://bmjopen.bmj.com/site/about/guidelines.xhtml

5-455.75	Partial resection of the colon: sigmoid resection: laparoscopic with anastomosis
5-455.76	Partial resection of the colon: Sigmoid resection: laparoscopic with enterostoma
5-455.77	Partial resection of the colon: sigmoid resection: change laparoscopic - open surgical
5-455.7X	Partial resection of the colon: Sigmoid resection: Other
5-455.91	Partial resection of the colon: Resection of the ascending colon with coecum and right flexure and transverse colon [right hemicolectomy with transverse resection]:
5-455.92	Partial resection of the colon: Resection of the ascending colon with coecum and right flexure and transverse colon [right hemicolectomy with transverse resection]:
5-455.93	Partial resection of the colon: Resection of the ascending colon with coecum and right flexure and transverse colon [right hemicolectomy with transverse resection]:
5-455.94	Partial resection of the colon: Resection of the ascending colon with coecum and right flexure and transverse colon [right hemicolectomy with transverse resection]:
5-455.95	Partial resection of the colon: Resection of the ascending colon with coecum and right flexure and transverse colon [right hemicolectomy with transverse resection]:
5-455.96	Partial resection of the colon: Resection of the ascending colon with coecum and right flexure and transverse colon [right hemicolectomy with transverse resection]:
5-455.97	Partial resection of the colon: Resection of the ascending colon with coecum and right flexure and transverse colon [right hemicolectomy with transverse resection]:
5-455.9X	Partial resection of the colon: Resection of the ascending colon with coecum and right flexure and transverse colon [right hemicolectomy with transverse resection]:
5-455.A1	Partial resection of the colon: Resection of the descending colon with left flexure and transverse colon [left hemicolectomy with transverse resection]: Open
5-455.A2	Partial resection of the colon: Resection of the descending colon with left flexure and transverse colon [left hemicolectomy with transverse resection]: Open
5-455.A3	Partial resection of the colon: Resection of the descending colon with left flexure and transverse colon [left hemicolectomy with transverse resection]: Open
5-455.A4	Partial resection of the colon: Resection of the descending colon with left flexure and transverse colon [left hemicolectomy with transverse resection]: Open
5-455.A5	Partial Resection of the Colon: Resection of the descending colon with left flexure and transverse colon [Hemicolectomy left with transverse resection]: Laparoscopic
5-455.A6	Partial Resection of the Colon: Resection of the descending colon with left flexure and transverse colon [Hemicolectomy left with transverse resection]: Laparoscopic
5-455.A7	Partial resection of the colon: resection of the descending colon with left flexure and transverse colon [hemicolectomy left with transverse resection]: change
5-455.AX	Partial resection of the colon: Resection of the descending colon with left flexure and transverse colon [left hemicolectomy with transverse resection]: Other
5-455.B1	Partial resection of the colon: resection of the descending colon and sigmoid colon: open surgery with anastomosis
5-455.B2	Partial resection of the colon: resection of the descending colon and sigmoid colon: open surgery with enterostoma and blind closure
5-455.B3	Partial Resection of the Colon: Resection of the descending colon and sigmoid colon: Open surgery with two enterostomata
5-455.B4	Partial Resection of the Colon: Resection of the descending colon and sigmoid colon: Open surgery with anastomosis anus praeter
5-455.B5	Partial resection of the colon: resection of the descending colon and sigmoid colon: laparoscopic with anastomosis
5-455.B6	Partial resection of the colon: resection of the descending colon and sigmoid colon: laparoscopic with enterostoma
5-455.B7	Partial Resection of the Colon: Resection of the descending colon and sigmoid colon: transfer laparoscopically - open surgery
5-455.BX	Partial resection of the colon: Resection of the descending colon and sigmoid colon: Other
5-455.C1	Partial resection of the colon: resection of the ascending, transverse and descending colon with coecum and right and left flexure [hemicolectomy right and left
5-455.C2	Partial resection of the colon: resection of the ascending, transverse and descending colon with coecum and right and left flexure [hemicolectomy right and left
5-455.C3	Partial resection of the colon: resection of the ascending, transverse and descending colon with coecum and right and left flexure [hemicolectomy right and left
5-455.C4	Partial resection of the colon: resection of the ascending, transverse and descending colon with coecum and right and left flexure [hemicolectomy right and left
5-455.C5	Partial resection of the colon: resection of the ascending, transverse and descending colon with coecum and right and left flexure [hemicolectomy right and left
5-455.C6	Partial resection of the colon: resection of the ascending, transverse and descending colon with coecum and right and left flexure [hemicolectomy right and left
5-455.C7	Partial resection of the colon: resection of the ascending, transverse and descending colon with coecum and right and left flexure [hemicolectomy right and left
5-455.CX	Partial resection of the colon: Resection of the ascending, transverse and descending colon with coecum and right and left flexure [hemicolectomy right and left
5-455.D1	Partial resection of the colon: resection of the transverse colon, descending colon with left flexure and sigmoid colon [hemicolectomy left with
5-455.D2	Partial resection of the colon: resection of the transverse colon, descending colon with left flexure and sigmoid colon [hemicolectomy left with
5-455.D3	Partial resection of the colon: resection of the transverse colon, descending colon with left flexure and sigmoid colon [hemicolectomy left with
5-455.D4	Partial resection of the colon: resection of the transverse colon, descending colon with left flexure and sigmoid colon [hemicolectomy left with
5-455.D5	Partial resection of the colon: resection of the transverse colon, descending colon with left flexure and sigmoid colon [hemicolectomy left with
5-455.D6	Partial resection of the colon: resection of the transverse colon, descending colon with left flexure and sigmoid colon [hemicolectomy left with
5-455.D7	Partial resection of the colon: resection of the transverse colon, descending colon with left flexure and sigmoid colon [hemicolectomy left with
5-455.DX	Partial resection of the colon: resection of the transverse colon, descending colon with left flexure and sigmoid colon [left hemicolectomy with
5-455.X1	Partial resection of the colon: Other: Open surgery with anastomosis
5-455.X2	Partial resection of the colon: Other: Open surgery with enterostoma and blind closure
5-455.X3	Partial resection of the colon: Other: Open surgery with two enterostomata
5-455.X4	Partial resection of the colon: Other: Open surgery with anastomosis anus praeter
5-455.X5	Partial resection of the colon: Other: Laparoscopic with anastomosis
5-455.X6	Partial resection of the colon: Other: Laparoscopic with enterostoma
5-455.X7	Partial resection of the colon: Other: transfer laparoscopic - open surgical
5-455.XX	Partial resection of the colon: Other: Other
5-455.Y	Partial resection of the colon: N.n.ref.

S3: OPS-codes for total colon resections according to GIQI[21]

OPS-CODE	TRANSLATION (BY AUTHOR)
5-456.00	(Total) Colectomy and Proctocolectomy: Colectomy: Open surgery with ileostoma
5-456.01	(Total) Colectomy and Proctocolectomy: Colectomy: Open surgery with ileorectal anastomosis with reservoir (pouch)
5-456.02	(Total) Colectomy and Proctocolectomy: Colectomy: Open surgery with ileorectal anastomosis without reservoir (pouch)
5-456.03	(Total) Colectomy and Proctocolectomy: Colectomy: Open surgery with ileoanal anastomosis with reservoir (pouch)
5-456.04	(Total) Colectomy and Proctocolectomy: Colectomy: Open surgery with ileoanal anastomosis without reservoir (pouch)
5-456.05	(Total) Colectomy and Proctocolectomy: Colectomy: Laparoscopic with reservoir anastomosis (Pouch)
5-456.06	(Total) Colectomy and Proctocolectomy: Colectomy: Laparoscopic with Anastomosis without reservoir (Pouch)
5-456.07	(Total) Colectomy and Proctocolectomy: Colectomy: Laparoscopic with Ileostoma
5-456.08	(Total) Colectomy and Proctocolectomy: Colectomy: change laparoscopic - open surgical
5-456.0X	(Total) Colectomy and Proctocolectomy: Colectomy: Other
5-456.10	(Total) Colectomy and Proctocolectomy: Proctocolectomy: Open surgery with ileostoma
5-456.11	(Total) Colectomy and Proctocolectomy: Proctocolectomy: Open surgery with ileorectal anastomosis with reservoir (pouch)
5-456.12	(Total) Colectomy and Proctocolectomy: Proctocolectomy: Open surgery with ileorectal anastomosis without reservoir (pouch)
5-456.13	(Total) Colectomy and Proctocolectomy: Proctocolectomy: Open surgery with ileoanal anastomosis with reservoir (pouch)
5-456.14	(Total) Colectomy and Proctocolectomy: Proctocolectomy: Open surgery with ileoanal anastomosis without reservoir (pouch)
5-456.15	(Total) colectomy and proctocolectomy: Proctocolectomy: Laparoscopic with reservoir anastomosis (pouch)
5-456.16	(Total) Colectomy and Proctocolectomy: Proctocolectomy: Laparoscopic with anastomosis without reservoir (pouch)
5-456.17	(Total) colectomy and proctocolectomy: proctocolectomy: laparoscopic with ileostomy
5-456.18	(Total) colectomy and proctocolectomy: proctocolectomy: change laparoscopic - open surgical
5-456.1X	(Total) colectomy and proctocolectomy: Proctocolectomy: Other
5-456.20	(Total) colectomy and proctocolectomy: colectomy with proctomucosectomy: open surgery with ileostoma
5-456.21	(Total) colectomy and proctocolectomy: colectomy with proctomucosectomy: open surgery with ileorectal anastomosis with reservoir (pouch)
5-456.22	(Total) Colectomy and Proctocolectomy: Colectomy with Proctomucosectomy: Open surgery with ileorectal anastomosis without reservoir (pouch)
5-456.23	(Total) Colectomy and Proctocolectomy: Colectomy with proctomucosectomy: Open surgery with ileoanal anastomosis with reservoir (pouch)
5-456.24	(Total) colectomy and proctocolectomy: colectomy with proctomucosectomy: open surgery with ileoanal anastomosis without reservoir (pouch)
5-456.25	(Total) colectomy and proctocolectomy: colectomy with proctomucosectomy: laparoscopic with reservoir anastomosis (pouch)
5-456.26	(Total) colectomy and proctocolectomy: colectomy with proctomucosectomy: laparoscopic with anastomosis without reservoir (pouch)
5-456.27	(Total) colectomy and proctocolectomy: colectomy with proctomucosectomy: laparoscopic with ileostomy
5-456.28	(Total) colectomy and proctocolectomy: colectomy with proctomucosectomy: change laparoscopic - open surgical
5-456.2X	(total) colectomy and proctocolectomy: colectomy with proctomucosectomy: other
5-456.X0	(Total) colectomy and proctocolectomy: Other: Open surgery with ileostomy
5-456.X1	(Total) colectomy and proctocolectomy: Other: Open surgery with ileorectal anastomosis with reservoir (pouch)
5-456.X2	(total) colectomy and proctocolectomy: Other: Open surgery with ileorectal anastomosis without reservoir (pouch)
5-456.X3	(Total) colectomy and proctocolectomy: Other: Open surgery with ileoanal anastomosis with reservoir (pouch)
5-456.X4	(Total) colectomy and proctocolectomy: Other: Open surgery with ileoanal anastomosis without reservoir (pouch)
5-456.X5	(total) colectomy and proctocolectomy: Other: Laparoscopic with reservoir anastomosis (pouch)
5-456.X6	(Total) colectomy and proctocolectomy: Other: Laparoscopic with anastomosis without reservoir (pouch)
5-456.X7	(Total) colectomy and proctocolectomy: Other: Laparoscopic with ileostomy
5-456.X8	(total) colectomy and proctocolectomy: Other: change laparoscopic - open surgical
5-456.XX	(Total) colectomy and proctocolectomy: Other: Other
5-456.Y	(Total) colectomy and proctocolectomy: N.n.d.

S4: OPS-codes for rectum resections according to GIQI[21]

OPS-CODE	TRANSLATION (BY AUTHOR)
5-484.01	Rectum resection with sphincter preservation: Anterior cuff resection: Open surgery with anastomosis
5-484.02	Rectum resection with sphincter preservation: Anterior cuff resection: Open surgery with enterostoma and blind closure
5-484.05	Rectum resection with sphincter preservation: Anterior cuff resection: Laparoscopic with anastomosis
5-484.06	Rectum resection with sphincter preservation: Anterior cuff resection: Laparoscopic with enterostomy and blind closure
5-484.08	Rectum resection with sphincter preservation: Anterior cuff resection: change laparoscopic - open surgery with anastomosis
5-484.09	Rectum resection with sphincter preservation: Anterior cuff resection: change laparoscopic - open surgery with enterostoma and blind closure
5-484.0X	Rectum resection with sphincter preservation: Anterior cuff resection: Other
5-484.11	Rectum resection with sphincter preservation: Posterior rectotomy [Rectotomia posterior]: Open surgery with anastomosis
5-484.12	Rectum resection with sphincter preservation: Posterior rectotomy [Rectotomia posterior]: Open surgery with enterostoma and blind closure
5-484.15	Rectum resection with sphincter preservation: Posterior cuff resection [Rectotomia posterior]: Laparoscopic with anastomosis
5-484.16	Rectum resection with sphincter preservation: Posterior cuff resection [Rectotomia posterior]: Laparoscopic with enterostoma and blind closure
5-484.18	Rectum resection with sphincter preservation: posterior cuff resection [Rectotomia posterior]: change laparoscopic - open surgery with anastomosis
5-484.19	Rectum resection with sphincter preservation: posterior cuff resection [rectotomia posterior]: change laparoscopically - open surgically with enterostoma and
5-484.1X	Rectum resection with sphincter preservation: Posterior cuff resection [Rectotomia posterior]: Other
5-484.21	Rectum resection with preservation of sphincter: Tubular resection with paraproctica left intact: Open surgery with anastomosis
5-484.22	Rectum resection with preservation of sphincter: Tubular resection with paraproktium left in place: Open surgery with enterostoma and blind closure
5-484.25	Rectum resection with preservation of sphincter: Tubular resection with paraproctica left in place: Laparoscopic with anastomosis
5-484.26	Rectum resection with preservation of sphincter: Tubular resection with paraproktium: Laparoscopic with enterostoma and blind closure
5-484.27	Rectum resection with sphincter preservation: Tubular resection with paraprotic left in place: Peranal
5-484.28	Rectum resection with sphincter preservation: Tubular resection with paraproctium left in place: change laparoscopic - open surgery with anastomosis
5-484.29	Rectum resection with preservation of sphincter: Tubular resection with preservation of paraproktium: change laparoscopically - open surgically with enterostoma and
5-484.2X	Rectum resection with preservation of sphincter: Tubular resection with preservation of paraproktium: Other
5-484.31	Rectum resection with sphincter preservation: Anterior resection: Open surgery with anastomosis
5-484.32	Rectum resection with sphincter preservation: Anterior resection: open surgery with enterostoma and blind closure
5-484.35	Rectum resection with sphincter preservation: Anterior resection: Laparoscopic with anastomosis
5-484.36	Rectum resection with sphincter preservation: Anterior resection: Laparoscopic with enterostoma and blind closure
5-484.38	Rectum resection with sphincter preservation: Anterior resection: change laparoscopic - open surgical with anastomosis
5-484.39	Rectum resection with sphincter preservation: anterior resection: change laparoscopically - open surgically with enterostoma and blind closure
5-484.3X	Rectum resection with sphincter preservation: Anterior resection: Other
5-484.51	Rectum resection with sphincter preservation: deep anterior resection: open surgery with anastomosis
5-484.52	Rectum resection with sphincter preservation: deep anterior resection: open surgery with enterostoma and blind closure
5-484.55	Rectum resection with sphincter preservation: Deep anterior resection: Laparoscopic with anastomosis
5-484.56	Rectum resection with sphincter preservation: Deep anterior resection: Laparoscopic with enterostoma and blind closure
5-484.58	Rectum resection with sphincter preservation: deep anterior resection: change laparoscopic - open surgery with anastomosis
5-484.59	Rectum resection with sphincter preservation: deep anterior resection: change laparoscopic - open surgical with enterostoma and blind closure
5-484.5X	Rectum resection with sphincter preservation: Deep anterior resection: Other
5-484.61	Rectum resection with sphincter preservation: deep anterior resection with peranal anastomosis: open surgery with anastomosis
5-484.65	Rectum resection with sphincter preservation: deep anterior
5-484.68	Rectum resection with sphincter preservation: deep anterior resection with peranal anastomosis: change laparoscopic - open surgical with anastomosis
5-484.6X	Rectum resection with sphincter preservation: Deep anterior resection with peranal anastomosis: Other
5-484.X1	Rectum resection with sphincter preservation: Other: Open surgery with anastomosis
5-484.X2	Rectum resection with sphincter preservation: Other: Open surgery with enterostoma and blind closure
5-484.X5	Rectum resection with sphincter preservation: Other: Laparoscopic with anastomosis
5-484.X6	Rectum resection with sphincter preservation: Other: Laparoscopic with enterostoma and blind closure

5-484.X8	Rectum resection with sphincter preservation: Other: change laparoscopic - open surgery with anastomosis
5-484.X9	Rectum resection with sphincter preservation: Other: change laparoscopically - open surgically with enterostoma and blind closure
5-484.XX	Rectum resection with sphincter preservation: Other: Other
5-484.Y	Rectum resection with sphincter preservation: N.n.ref.
5-485.01	Rectum resection without preservation of sphincter: Abdominoperineal: Open surgery
5-485.02	Rectum resection without sphincter preservation: Abdominoperineal: Combined open surgical-laparoscopic
5-485.0X	Rectum resection without sphincter preservation: Abdominoperineal: Other
5-485.1	Rectum resection without preservation of sphincter: Abdominoperineal with removal of adjacent organs
5-485.21	Rectum resection without sphincter preservation: Sacroiliac abdomen: Open surgery
5-485.22	Rectum resection without sphincter preservation: Sacroiliac abdomen: Combined open surgical-laparoscopic
5-485.2X	Rectum resection without sphincter preservation: Sacroiliac abdomen: Other
5-485.3	Rectum resection without sphincter preservation: Sacral abdominosacral with removal of neighboring organs
5-485.4	Rectum resection without sphincter preservation: sacroperineal
5-485.5	Rectum resection without sphincter preservation: Perineal
5-485.X	Rectum resection without sphincter preservation: Other
5-485.Y	Rectum resection without sphincter preservation: N.n.ref.

For peer review only

S5: Definitions of patient safety outcomes

PATIENT SAFETY OUTCOME	DEFINITION ACCORDING TO ICD-10 GM
DEATH	Discharge destination: death (included in accounting data derived)
POST-OPERATIVE RESPIRATORY FAILURE[15]	ICD-10: J96 .0 - Respiratory failure, not elsewhere classified
POST-OPERATIVE WOUND INFECTION[15]	ICD-10: T81.4 - Infection following a procedure, not elsewhere classified<br>ICD-10: T82.6 - Infection and inflammatory reaction due to cardiac valve prosthesis<br>ICD-10: T82.7 - Infection and inflammatory reaction due to other cardiac and vascular devices, implants and grafts<br>ICD-10: T83.5 - Infection and inflammatory reaction due to prosthetic device, implant and graft in urinary system<br>ICD-10: T83.6 - Infection and inflammatory reaction due to prosthetic device, implant and graft in genital tract<br>ICD-10: T84.5 - Infection and inflammatory reaction due to internal joint prosthesis<br>ICD-10: T84.6 - Infection and inflammatory reaction due to internal fixation device [any site]<br>ICD-10: T84.7 - Infection and inflammatory reaction due to other internal orthopaedic prosthetic devices, implants and grafts<br>ICD-10: T87.4 - Infection of amputation stump
RENAL FAILURE[15]	ICD-10: N17.0 - Acute renal failure with tubular necrosis<br>ICD-10: N17.1 - Acute renal failure with acute cortical necrosis<br>ICD-10: N17.2 - Acute renal failure with medullary necrosis<br>ICD-10: N17.8 - Other acute renal failure<br>ICD-10: N17.9 - Acute renal failure, unspecified<br>ICD-10: N99.0- Postprocedural renal failure<br>Procedure code: 8-853.7- Continuous, venovenous, pump-driven (CVVH), anticoagulation with heparin or without anticoagulation

For peer review only

S6: Case-, care- and hospital related characteristics

	COLON RESECTIONS		RECTUM RESECTIONS	
**PATIENT SAFETY OUTCOMES**	n	% / Q1; Q3	n	% / Q1; Q3
Outcome death				
no	48,914	(90.30 %)	19,525	(95.73 %)
yes	5,254	(9.69 %)	870	(4.26 %)
Outcome post-operative respiratory failure				
no	45,074	(83.21 %)	17,901	(87.77 %)
yes	9,094	(16.78 %)	2,494	(12.22 %)
Outcome post-operative wound infections				
no	48,013	(88.63 %)	18,109	(88.79 %)
yes	6,155	(11.36 %)	2,286	(11.2 %)
Outcome renal failure				
no	45,920	(84.77 %)	18,279	(89.62 %)
yes	8,248	(15.22 %)	2,116	(10.37 %)
**CASE CHARACTERISTICS**				
age (median)	68	(56; 77)	67	(57; 77)
elixhauser groups per case (median)	3	(2; 5)	3	(2; 5)
sex				
male	26,954	(49.76 %)	10,367	(50.83 %)
female	27,214	(50.23 %)	10,028	(49.16 %)
AIDS/HIV				
no	54,142	(99.95 %)	20,383	(99.94 %)
yes	26	(0.04 %)	12	(0.05 %)
alcohol abuse				
no	53,025	(97.88 %)	20,088	(98.49 %)
yes	1,143	(2.11 %)	307	(1.5 %)
blood loss anemia				
no	53,022	(97.88 %)	20,134	(98.72 %)
yes	1,146	(2.11 %)	261	(1.27 %)
cardiac arrhythmias				
no	43,241	(79.82 %)	17,135	(84.01 %)
yes	10,927	(20.17 %)	3,260	(15.98 %)
chronic pulmonary disease				
no	49,042	(90.53 %)	18,792	(92.14 %)
yes	5,126	(9.46 %)	1,603	(7.85 %)
coagulopathy				
no	44,935	(82.95 %)	17,598	(86.28 %)
yes	9,233	(17.04 %)	2,797	(13.71 %)
congestive heart failure				
no	47,447	(87.59 %)	18,608	(91.23 %)
yes	6,721	(12.4 %)	1,787	(8.76 %)
deficiency anemia				
no	51,887	(95.78 %)	19,852	(97.33 %)
yes	2,281	(4.21 %)	543	(2.66 %)
depression				
no	51,232	(94.57 %)	19,318	(94.71 %)
yes	2,936	(5.42 %)	1,077	(5.28 %)
diabetes, complicated				
no	51,887	(95.78 %)	19,754	(96.85 %)
yes	2,281	(4.21 %)	641	(3.14 %)
diabetes, uncomplicated				
no	46,491	(85.82 %)	17,664	(86.6 %)
yes	7,677	(14.17 %)	2,731	(13.39 %)
drug abuse				
no	53,911	(99.52 %)	20,321	(99.63 %)
yes	257	(0.47 %)	74	(0.36 %)
fluid and electrolyte disorders				
no	29,678	(54.78 %)	12,122	(59.43 %)
yes	24,490	(45.21 %)	8,273	(40.56 %)
hypertension, complicated				
no	51,632	(95.31 %)	19,680	(96.49 %)
yes	2,536	(4.68 %)	715	(3.51 %)
hypertension, uncomplicated				
no	28,369	(52.37 %)	10,692	(52.42 %)
yes	25,799	(47.62 %)	9,703	(47.57 %)

For peer review only

hypothyroidism				
no	47,235	(87.2 %)	18,008	(88.29 %)
yes	6,933	(12.79 %)	2,387	(11.7 %)
liver disease				
no	50,277	(92.81 %)	19,433	(95.28 %)
yes	3,891	(7.18 %)	962	(4.71 %)
lymphoma				
no	53,762	(99.25 %)	20,315	(99.6 %)
yes	406	(0.74 %)	80	(0.39 %)
metastatic cancer				
no	43,637	(80.55 %)	15,149	(74.27 %)
yes	10,531	(19.44 %)	5,246	(25.72 %)
obesity				
no	47,727	(88.11 %)	18,046	(88.48 %)
yes	6,441	(11.89 %)	2,349	(11.51 %)
other neurological disorders				
no	51,956	(95.91 %)	19,747	(96.82 %)
yes	2,212	(4.08 %)	648	(3.17 %)
paralysis				
no	52,765	(97.4 %)	19,989	(98.01 %)
yes	1,403	(2.59 %)	406	(1.99 %)
peptic ulcer disease excluding bleeding				
no	54,004	(99.69 %)	20,348	(99.76 %)
yes	164	(0.31 %)	47	(0.23 %)
peripheral vascular disorders				
no	49,190	(90.81 %)	18,228	(89.37 %)
yes	4,978	(9.18 %)	2,167	(10.62 %)
psychoses				
no	53,889	(99.48 %)	20,330	(99.68 %)
yes	279	(0.51 %)	65	(0.31 %)
pulmonary circulation disorders				
no	52,476	(96.87 %)	19,929	(97.71 %)
yes	1,692	(3.12 %)	466	(2.28 %)
renal Diseases				
no	45,739	(84.43 %)	17,896	(87.74 %)
yes	8,429	(15.56 %)	2,499	(12.25 %)
rheumatoid arthritis/ collagen vascular diseases				
no	53,262	(98.32 %)	20,115	(98.62 %)
yes	906	(1.67 %)	280	(1.37 %)
solid tumor without metastasis				
no	28,520	(52.65 %)	6,722	(32.95 %)
yes	25,648	(47.34 %)	13,673	(67.04 %)
valvular disease				
no	51,473	(95.02 %)	19,591	(96.05 %)
yes	2,695	(4.97 %)	804	(3.94 %)
weight loss				
no	46,134	(85.16 %)	17,442	(85.52 %)
yes	8,034	(14.83 %)	2,953	(14.47 %)
**CARE CHARACTERISTICS**				
total colon resections				
no	51,310	(94.72 %)	20,173	(98.91 %)
yes	2,858	(5.27 %)	222	(1.08 %)
partial colon resections				
no	2,662	(4.91 %)	17,088	(83.78 %)
yes	51,506	(95.08 %)	3,307	(16.21 %)
rectum resections				
no	50,665	(93.53 %)	-	-
yes	3,503	(6.46 %)	20,395	(100.00 %)
colon and rectum resection				
no	50,665	(93.53 %)	16,892	(82.82 %)
yes	3,503	(6.46 %)	3,503	(17.17 %)
weekend surgery				
no	49,473	(91.33 %)	19,603	(96.11 %)
yes	4,695	(8.66 %)	792	(3.88 %)
admission				
referral	36,129	(66.69 %)	16,249	(79.67 %)
emergency case	16,116	(29.75 %)	3,744	(18.35 %)
transfer from other hospital	1,923	(3.55 %)	402	(1.97 %)

For peer review only

**HOSPITAL CHARACTERISTICS**				
annual volume				
colon resection cases (median)	72	(38; 119)	-	-
total colon resection (median)	1	(0;3)	-	-
rectum resections (median)	-	-	26	(11;42)
urbanization				
urban	124	(59.33 %)	119	(59.50 %)
rural	85	(40.66 %)	81	(40.50 %)
ownership				
public	82	(39.23 %)	80	(40.00 %)
non-profit	41	(19.61 %)	39	(19.50 %)
private	86	(41.14 %)	81	(40.50 %)
university hospital				
no	201	(96.17 %)	192	(96.00 %)
yes	8	(3.82 %)	8	(4.00 %)

For peer review only

S7: Bivariate analysis of case-, care- and hospital-related covariates of patient safety including all 54.168 colon resections in 209 hospitals

	DEATH		POST-OPERATIVE RESPIRATORY FAILURE		RENAL FAILURE		POST-OPERATIVE WOUND INFECTION	
	OR	95% CI	OR	95% CI	OR	95% CI	OR	95% CI
**PATIENT COVARIATES**								
sex								
male	Ref.		Ref.		Ref.		Ref.	
female	0.876***	(0.827 - 0.928)	0.828***	(0.790 - 0.868)	0.742***	(0.707 - 0.778)	0.931**	(0.881 - 0.983)
age	1.052***	(1.049 - 1.055)	1.030***	(1.028 - 1.031)	1.037***	(1.035 - 1.039)	1.008***	(1.006 - 1.009)
alcohol abuse	3.845***	(3.363 - 4.396)	3.862***	(3.400 - 4.387)	3.860***	(3.406 - 4.374)	1.945***	(1.669 - 2.266)
blood loss anemia	0.715**	(0.570 - 0.896)	1.355***	(1.159 - 1.585)	1.200*	(1.025 - 1.405)	1.387***	(1.159 - 1.659)
cardiac arrhythmias	4.338***	(4.084 - 4.608)	3.544***	(3.364 - 3.734)	3.995***	(3.791 - 4.209)	1.766***	(1.660 - 1.878)
chronic pulmonary disease	2.025***	(1.867 - 2.196)	2.120***	(1.978 - 2.273)	1.793***	(1.668 - 1.927)	1.554***	(1.431 - 1.689)
coagulopathy	9.015***	(8.439 - 9.630)	6.370***	(6.011 - 6.751)	7.207***	(6.798 - 7.640)	2.483***	(2.331 - 2.646)
congestive heart failure	4.645***	(4.342 - 4.969)	4.294***	(4.042 - 4.563)	4.382***	(4.127 - 4.652)	1.899***	(1.765 - 2.044)
deficiency anemia	1.013	(0.878 - 1.169)	1.448***	(1.302 - 1.610)	1.416***	(1.268 - 1.582)	1.250***	(1.101 - 1.418)
depression	0.888	(0.779 - 1.012)	1.827***	(1.669 - 2.000)	1.372***	(1.244 - 1.513)	1.923***	(1.741 - 2.125)
diabetes, complicated	2.766***	(2.481 - 3.084)	2.339***	(2.121 - 2.578)	2.946***	(2.678 - 3.240)	1.705***	(1.514 - 1.921)
diabetes, uncomplicated	1.485***	(1.378 - 1.600)	1.559***	(1.465 - 1.659)	1.594***	(1.497 - 1.698)	1.300***	(1.208 - 1.399)
fluid and electrolyte disorders	4.072***	(3.809 - 4.354)	4.463***	(4.229 - 4.709)	5.033***	(4.756 - 5.328)	2.650***	(2.497 - 2.812)
hypertension, complicated	2.124***	(1.896 - 2.380)	2.156***	(1.957 - 2.375)	2.446***	(2.225 - 2.688)	1.453***	(1.288 - 1.638)
hypertension, uncomplicated	0.961	(0.906 - 1.020)	1.266***	(1.207 - 1.329)	1.294***	(1.232 - 1.360)	1.198***	(1.132 - 1.267)
hypothyroidism	0.964	(0.883 - 1.052)	1.227***	(1.147 - 1.312)	1.176***	(1.096 - 1.261)	1.316***	(1.219 - 1.421)
liver disease	6.486***	(6.007 - 7.004)	2.844***	(2.635 - 3.069)	4.573***	(4.254 - 4.917)	1.345***	(1.223 - 1.478)
lymphoma	1.869***	(1.441 - 2.424)	1.319*	(1.030 - 1.690)	1.850***	(1.470 - 2.327)	1.246	(0.939 - 1.654)
metastatic cancer	0.948	(0.881 - 1.021)	1.004	(0.946 - 1.065)	0.827***	(0.777 - 0.881)	1.069	(1.000 - 1.143)
obesity	0.780***	(0.707 - 0.860)	1.173***	(1.092 - 1.259)	1.419***	(1.324 - 1.521)	1.656***	(1.533 - 1.789)
other neurological disorders	2.854***	(2.567 - 3.174)	3.417***	(3.109 - 3.754)	2.834***	(2.575 - 3.119)	1.860***	(1.660 - 2.084)
paralysis	2.709***	(2.373 - 3.092)	3.030***	(2.692 - 3.410)	2.435***	(2.156 - 2.751)	1.874***	(1.629 - 2.156)
peripheral vascular disorders	3.572***	(3.316 - 3.848)	2.745***	(2.562 - 2.941)	3.001***	(2.804 - 3.213)	1.741***	(1.606 - 1.889)
psychoses	1.004	(0.676 - 1.492)	2.578***	(1.978 - 3.361)	1.232	(0.903 - 1.679)	1.845***	(1.352 - 2.517)
pulmonary circulation disorders	3.533***	(3.150 - 3.964)	3.959***	(3.562 - 4.401)	3.185***	(2.863 - 3.543)	1.721***	(1.511 - 1.960)
renal failure	2.772***	(2.592 - 2.966)	2.261***	(2.132 - 2.398)	2.982***	(2.815 - 3.158)	1.524***	(1.419 - 1.636)
rheumatoid arthritis/collagen vascular diseases	1.393**	(1.141 - 1.702)	1.283**	(1.079 - 1.524)	1.373***	(1.156 - 1.630)	1.196	(0.980 - 1.461)
solid tumor without metastasis	0.660***	(0.622 - 0.701)	0.845***	(0.806 - 0.886)	0.760***	(0.724 - 0.798)	0.949	(0.898 - 1.002)
valvular disease	2.580***	(2.332 - 2.855)	2.355***	(2.151 - 2.579)	2.632***	(2.409 - 2.876)	1.426***	(1.275 - 1.594)
weight loss	1.439***	(1.328 - 1.560)	2.418***	(2.269 - 2.576)	2.091***	(1.963 - 2.228)	2.230***	(2.073 - 2.398)
**CARE COVARIATES**								
admission								
referral	Ref.		Ref.		Ref.		Ref.	
emergency case	3.066***	(2.852 - 3.297)	2.263***	(2.133 - 2.401)	2.409***	(2.260 - 2.567)	1.461***	(1.368 - 1.559)
transfer from other hospital	5.722***	(5.105 - 6.412)	4.200***	(3.768 - 4.680)	4.308***	(3.870 - 4.795)	1.865***	(1.645 - 2.115)
weekend Surgery								
no	Ref.		Ref.		Ref.		Ref.	
yes	2.704***	(2.498 - 2.927)	2.148***	(1.999 - 2.308)	2.254***	(2.099 - 2.419)	1.382***	(1.265 - 1.509)

total colon resection								
no	Ref.		Ref.		Ref.		Ref.	
yes	3.687***	(3.351 - 4.057)	2.395***	(2.186 - 2.625)	2.954***	(2.704 - 3.227)	1.340***	(1.204 - 1.493)
colon and rectum resection								
no	Ref.		Ref.		Ref.		Ref.	
yes	1.114	(0.991 - 1.252)	1.563***	(1.429 - 1.710)	1.390***	(1.267 - 1.525)	1.757***	(1.593 - 1.937)
**HOSPITAL COVARIATES**								
case volume	1.071	(0.978 - 1.173)	0.934	(0.837 - 1.043)	1.044	(0.944 - 1.155)	1.265***	(1.117 - 1.432)
area								
urban	Ref.		Ref.		Ref.		Ref.	
rural	0.830*	(0.711 - 0.969)	0.849	(0.665 - 1.085)	0.760**	(0.624 - 0.927)	0.837	(0.661 - 1.059)
university hospital								
no	Ref.		Ref.		Ref.		Ref.	
yes	1.946***	(1.411 - 2.683)	1.003	(0.555 - 1.814)	1.767*	(1.113 - 2.806)	3.178***	(1.894 - 5.333)
ownership								
public	Ref.		Ref.		Ref.		Ref.	
non-profit	0.774*	(0.634 - 0.945)	0.915	(0.657 - 1.272)	0.691**	(0.534 - 0.895)	0.615**	(0.452 - 0.835)
private	0.748***	(0.636 - 0.880)	1.025	(0.785 - 1.338)	1.226	(0.999 - 1.504)	0.662**	(0.518 - 0.847)

Note: Elixhauser Groups drug abuse, peptic ulcer disease and AIDS/ HIV were not included into the regression due to low case numbers
*** p<0.001
** p <0.01
* p<0.05

For peer review only

S8: Bivariate analysis of case-, care- and hospital-related covariates of patient safety including all 20.395 rectum resections in 200 hospitals

-	DEATH		POST-OPERATIVE RESPIRATORY FAILURE		RENAL FAILURE		POST-OPERATIVE WOUND INFECTION	
**PATIENT COVARIATES**	OR	95% CI	OR	95% CI	OR	95% CI	OR	95% CI
sex								
male	Ref.		Ref.		Ref.		Ref.	
female	0.915	(0.796 - 1.050)	0.913*	(0.836 - 0.998)	0.787***	(0.717 - 0.864)	0.877**	(0.801 - 0.960)
age	1.075***	(1.067 - 1.082)	1.031***	(1.027 - 1.034)	1.039***	(1.035 - 1.043)	1.007***	(1.004 - 1.010)
alcohol abuse	2.767***	(1.915 - 3.997)	2.705***	(2.050 - 3.570)	2.531***	(1.916 - 3.345)	1.826***	(1.356 - 2.459)
blood loss anemia	1.984**	(1.270 - 3.100)	1.769**	(1.256 - 2.493)	2.637***	(1.942 - 3.581)	2.264***	(1.619 - 3.165)
cardiac arrhythmias	5.465***	(4.741 - 6.299)	3.457***	(3.124 - 3.825)	3.777***	(3.410 - 4.183)	1.564***	(1.399 - 1.748)
chronic pulmonary disease	2.213***	(1.820 - 2.690)	2.185***	(1.905 - 2.506)	1.835***	(1.587 - 2.122)	1.690***	(1.463 - 1.952)
coagulopathy	9.352***	(8.020 - 10.906)	5.781***	(5.171 - 6.463)	5.702***	(5.091 - 6.386)	2.680***	(2.401 - 2.993)
congestive heart failure	5.853***	(4.995 - 6.858)	4.275***	(3.781 - 4.834)	4.272***	(3.787 - 4.820)	1.793***	(1.560 - 2.060)
deficiency anemia	1.741**	(1.241 - 2.442)	2.119***	(1.696 - 2.648)	2.275***	(1.810 - 2.858)	1.893***	(1.499 - 2.391)
depression	0.872	(0.637 - 1.192)	1.675***	(1.409 - 1.991)	1.686***	(1.411 - 2.014)	1.701***	(1.436 - 2.014)
diabetes, complicated	3.196***	(2.455 - 4.160)	2.073***	(1.692 - 2.540)	3.187***	(2.635 - 3.854)	1.730***	(1.390 - 2.154)
diabetes, uncomplicated	1.653***	(1.388 - 1.969)	1.613***	(1.435 - 1.813)	1.660***	(1.472 - 1.873)	1.341***	(1.184 - 1.518)
fluid and electrolyte disorders	3.961***	(3.387 - 4.633)	4.043***	(3.660 - 4.467)	4.586***	(4.122 - 5.102)	2.563***	(2.324 - 2.826)
hypertension, complicated	2.656***	(2.033 - 3.470)	2.366***	(1.947 - 2.876)	2.681***	(2.225 - 3.230)	1.480***	(1.188 - 1.844)
hypertension, uncomplicated	1.094	(0.951 - 1.257)	1.358***	(1.241 - 1.486)	1.449***	(1.318 - 1.593)	1.159**	(1.057 - 1.271)
hypothyroidism	0.980	(0.790 - 1.215)	1.164*	(1.019 - 1.329)	1.170*	(1.019 - 1.344)	1.125	(0.981 - 1.290)
liver disease	5.887***	(4.864 - 7.124)	2.298***	(1.937 - 2.725)	3.606***	(3.084 - 4.216)	1.539***	(1.279 - 1.850)
lymphoma	2.350*	(1.112 - 4.967)	1.618	(0.882 - 2.967)	1.766	(0.972 - 3.208)	1.683	(0.946 - 2.992)
metastatic cancer	0.979	(0.835 - 1.148)	1.298***	(1.177 - 1.431)	0.978	(0.879 - 1.087)	1.187***	(1.075 - 1.311)
obesity	0.908	(0.726 - 1.135)	1.500***	(1.320 - 1.704)	1.632***	(1.435 - 1.855)	1.653***	(1.455 - 1.878)
other neurological disorders	3.942***	(3.107 - 5.001)	3.107***	(2.569 - 3.757)	2.277***	(1.860 - 2.788)	1.887***	(1.530 - 2.327)
paralysis	3.060***	(2.244 - 4.173)	2.958***	(2.336 - 3.745)	2.816***	(2.220 - 3.572)	1.849***	(1.425 - 2.399)
peripheral vascular disorders	2.521***	(2.118 - 3.000)	1.673***	(1.469 - 1.905)	1.924***	(1.690 - 2.189)	1.531***	(1.341 - 1.748)
psychoses	0.943	(0.291 - 3.056)	0.843	(0.371 - 1.914)	1.865	(0.969 - 3.592)	1.337	(0.660 - 2.707)
pulmonary circulation disorders	6.535***	(5.123 - 8.336)	4.514***	(3.663 - 5.563)	3.734***	(3.022 - 4.613)	2.041***	(1.619 - 2.573)
renal failure	2.984***	(2.532 - 3.518)	2.416***	(2.147 - 2.718)	3.393***	(3.031 - 3.797)	1.688***	(1.488 - 1.915)
rheumatoid arthritis/collagen vascular diseases	1.521	(0.932 - 2.482)	1.582**	(1.140 - 2.196)	1.471*	(1.043 - 2.076)	0.868	(0.580 - 1.301)
solid tumor without metastasis	0.571***	(0.494 - 0.659)	1.002	(0.906 - 1.107)	0.836***	(0.755 - 0.924)	1.090	(0.984 - 1.207)
valvular disease	3.682***	(2.935 - 4.619)	2.693***	(2.257 - 3.214)	2.803***	(2.351 - 3.341)	1.364**	(1.113 - 1.672)
weight loss	1.408***	(1.165 - 1.702)	2.522***	(2.244 - 2.834)	2.072***	(1.842 - 2.331)	2.079***	(1.843 - 2.346)
**CARE COVARIATES**								
admission reason								
referral	Ref.		Ref.		Ref.		Ref.	
emergency case	4.038***	(3.450 - 4.727)	2.200***	(1.963 - 2.465)	2.272***	(2.013 - 2.563)	1.656***	(1.474 - 1.862)
transfer from other hospital	6.550***	(4.887 - 8.780)	3.944***	(3.088 - 5.038)	4.248***	(3.337 - 5.406)	2.208***	(1.705 - 2.861)
weekend surgery								
no	Ref.		Ref.		Ref.		Ref.	
yes	3.693***	(2.927 - 4.659)	2.215***	(1.831 - 2.678)	2.177***	(1.809 - 2.620)	1.281*	(1.030 - 1.593)
colon and rectum resection								

no	Ref.		Ref.		Ref.		Ref.	
yes	4.015***	(3.458 - 4.663)	3.095***	(2.781 - 3.444)	2.706***	(2.428 - 3.016)	2.014***	(1.805 - 2.247)
**HOSPITAL COVARIATES**								
case volume	0.743***	(0.661 - 0.835)	0.828**	(0.730 - 0.940)	0.830***	(0.746 - 0.924)	1.079	(0.946 - 1.232)
area								
urban	Ref.		Ref.		Ref.		Ref.	
rural	0.882	(0.680 - 1.143)	0.925	(0.684 - 1.251)	0.895	(0.709 - 1.129)	0.726*	(0.554 - 0.951)
university hospital								
no	Ref.		Ref.		Ref.		Ref.	
yes	1.439	(0.890 - 2.324)	0.857	(0.435 - 1.690)	1.134	(0.696 - 1.847)	3.037***	(1.778 - 5.186)
ownership								
public	Ref.		Ref.		Ref.		Ref.	
non-profit	0.691*	(0.500 - 0.954)	0.887	(0.594 - 1.324)	0.633**	(0.472 - 0.848)	0.605**	(0.428 - 0.854)
private	0.631***	(0.480 - 0.830)	1.070	(0.773 - 1.482)	1.245	(0.988 - 1.571)	0.673**	(0.508 - 0.893)

Note: Elixhauser Groups drug abuse, peptic ulcer disease and AIDS/ HIV were not included into the regression due to a low case numbers
*** p<0.001
** p <0.01
* p<0.05

For peer review only

S9: Multivariate analysis of case-, care- and hospital-related covariates of patient safety including all 54.168 colon resections in 209 hospitals

	DEATH		POST-OPERATIVE RESPIRATORY FAILURE		RENAL FAILURE		POST-OPERATIVE WOUND INFECTION	
	OR	95% CI	OR	95% CI	OR	95% CI	OR	95% CI
**CASE COVARIATES**								
sex								
male	Ref.		Ref		Ref		Ref	
female	0.937	(0.873 - 1.006)	0.788***	(0.745 - 0.833)	0.683***	(0.645 - 0.725)	0.882***	(0.832 - 0.936)
age	1.050***	(1.046 - 1.053)	1.014***	(1.012 - 1.017)	1.024***	(1.021 - 1.026)	0.998	(0.996 - 1.000)
alcohol abuse	1.484***	(1.250 - 1.761)	1.948***	(1.677 - 2.263)	1.574***	(1.352 - 1.833)	1.304**	(1.106 - 1.539)
blood loss anemia	0.472***	(0.365 - 0.609)	0.982	(0.823 - 1.172)	0.831*	(0.695 - 0.995)	1.154	(0.958 - 1.390)
cardiac arrhythmias	1.640***	(1.518 - 1.772)	1.585***	(1.487 - 1.690)	1.627***	(1.524 - 1.737)	1.183***	(1.100 - 1.271)
chronic pulmonary disease	1.362***	(1.234 - 1.503)	1.459***	(1.347 - 1.581)	1.113*	(1.022 - 1.212)	1.242***	(1.138 - 1.356)
coagulopathy	4.174***	(3.864 - 4.509)	3.117***	(2.920 - 3.327)	3.332***	(3.118 - 3.561)	1.644***	(1.531 - 1.764)
congestive heart failure	1.748***	(1.597 - 1.914)	1.902***	(1.759 - 2.056)	1.678***	(1.551 - 1.815)	1.218***	(1.114 - 1.332)
deficiency anemia	0.778**	(0.660 - 0.918)	1.058	(0.937 - 1.194)	1.096	(0.965 - 1.245)	1.024	(0.897 - 1.169)
depression	0.658***	(0.566 - 0.765)	1.425***	(1.286 - 1.580)	1.070	(0.956 - 1.197)	1.569***	(1.414 - 1.741)
diabetes, complicated	1.297***	(1.131 - 1.487)	1.181**	(1.051 - 1.328)	1.274***	(1.135 - 1.431)	1.191**	(1.045 - 1.358)
diabetes, uncomplicated	1.128*	(1.029 - 1.237)	1.201***	(1.117 - 1.291)	1.151***	(1.067 - 1.241)	1.113**	(1.028 - 1.204)
fluid and electrolyte disorders	1.884***	(1.743 - 2.038)	2.561***	(2.412 - 2.719)	2.882***	(2.704 - 3.072)	1.991***	(1.867 - 2.123)
hypertension, complicated	0.507***	(0.437 - 0.589)	0.735***	(0.649 - 0.832)	0.805***	(0.712 - 0.911)	0.928	(0.807 - 1.067)
hypertension, uncomplicated	0.551***	(0.511 - 0.595)	0.945	(0.890 - 1.003)	0.921*	(0.865 - 0.981)	1.045	(0.980 - 1.115)
hypothyroidism	0.795***	(0.716 - 0.884)	1.110**	(1.027 - 1.200)	1.046	(0.963 - 1.137)	1.206***	(1.112 - 1.309)
liver disease	4.411***	(3.999 - 4.865)	1.373***	(1.253 - 1.506)	2.588***	(2.368 - 2.829)	0.831***	(0.749 - 0.923)
lymphoma	0.983	(0.717 - 1.349)	0.810	(0.613 - 1.070)	1.111	(0.846 - 1.458)	1.029	(0.767 - 1.382)
metastatic cancer	1.524***	(1.376 - 1.687)	1.120**	(1.036 - 1.211)	0.984	(0.906 - 1.070)	1.058	(0.974 - 1.149)
obesity	0.766***	(0.682 - 0.862)	1.165***	(1.073 - 1.265)	1.498***	(1.379 - 1.627)	1.634***	(1.506 - 1.773)
other neurological disorders	1.504***	(1.319 - 1.714)	1.903***	(1.706 - 2.123)	1.515***	(1.352 - 1.699)	1.259***	(1.114 - 1.422)
paralysis	1.481***	(1.261 - 1.740)	1.468***	(1.281 - 1.682)	1.142	(0.989 - 1.318)	1.231**	(1.060 - 1.429)
peripheral vascular disorders	1.926***	(1.757 - 2.111)	1.493***	(1.378 - 1.618)	1.518***	(1.399 - 1.647)	1.239***	(1.136 - 1.351)
psychoses	0.707	(0.449 - 1.113)	2.052***	(1.516 - 2.778)	0.859	(0.600 - 1.229)	1.493*	(1.081 - 2.061)
pulmonary circulation disorders	1.593***	(1.380 - 1.838)	1.864***	(1.649 - 2.108)	1.336***	(1.174 - 1.519)	1.071	(0.933 - 1.231)
renal failure	1.308***	(1.200 - 1.425)	1.112**	(1.035 - 1.196)	1.505***	(1.401 - 1.616)	1.067	(0.984 - 1.156)
rheumatoid arthritis/collagen vascular diseases	1.001	(0.793 - 1.264)	0.971	(0.798 - 1.182)	0.982	(0.806 - 1.197)	1.056	(0.858 - 1.298)
solid tumor without metastasis	0.573***	(0.525 - 0.625)	0.764***	(0.715 - 0.816)	0.707***	(0.660 - 0.757)	0.907**	(0.845 - 0.975)
valvular disease	0.817**	(0.719 - 0.929)	0.862**	(0.772 - 0.961)	0.927	(0.831 - 1.033)	0.927	(0.820 - 1.048)
weight loss	0.891*	(0.809 - 0.980)	1.658***	(1.542 - 1.783)	1.426***	(1.324 - 1.535)	1.774***	(1.643 - 1.916)
**CARE COVARIATES**								
admission reason								
referral	Ref.		Ref.		Ref.		Ref.	
emergency case	1.847***	(1.692 - 2.015)	1.413***	(1.320 - 1.513)	1.453***	(1.349 - 1.566)	1.145***	(1.067 - 1.228)
transfer from other hospital	2.528***	(2.193 - 2.915)	1.982***	(1.749 - 2.245)	1.908***	(1.678 - 2.171)	1.223**	(1.071 - 1.397)
weekend surgery								
no	Ref.		Ref.		Ref.		Ref.	
yes	1.669***	(1.515 - 1.839)	1.426***	(1.312 - 1.550)	1.480***	(1.360 - 1.610)	1.080	(0.984 - 1.186)

total colon resection								
no	Ref.		Ref.		Ref.		Ref.	
yes	2.679***	(2.369 - 3.029)	1.639***	(1.472 - 1.825)	2.228***	(1.999 - 2.483)	1.022	(0.913 - 1.143)
colon and rectum resection								
no	Ref.		Ref.		Ref.		Ref.	
yes	1.103	(0.960 - 1.267)	1.524***	(1.378 - 1.686)	1.408***	(1.265 - 1.567)	1.579***	(1.426 - 1.748)
**HOSPITAL COVARIATES**								
case volume	0.968	(0.871 - 1.076)	0.919	(0.807 - 1.047)	0.992	(0.891 - 1.106)	1.168*	(1.030 - 1.325)
area								
urban	Ref.		Ref.		Ref.		Ref.	
rural	1.061	(0.893 - 1.261)	0.863	(0.648 - 1.149)	0.772**	(0.635 - 0.939)	1.032	(0.824 - 1.292)
university hospital								
no	Ref.		Ref.		Ref.		Ref.	
yes	1.303	(0.888 - 1.912)	0.687	(0.338 - 1.397)	1.412	(0.889 - 2.241)	1.981*	(1.171 - 3.352)
ownership								
public	Ref.		Ref.		Ref.		Ref.	
non-profit	1.012	(0.811 - 1.262)	1.057	(0.726 - 1.540)	0.867	(0.670 - 1.122)	0.744*	(0.555 - 0.998)
private	1.244*	(1.026 - 1.507)	1.329	(0.972 - 1.817)	1.937***	(1.563 - 2.400)	0.777*	(0.608 - 0.992)

Note: Elixhauser Groups drug abuse, peptic ulcer disease and AIDS/ HIV were not included into the regression due to a low case numbers

*** p<0.001

** p <0.01

* p<0.05

For peer review only

S10: Multivariate analysis of case-, care- and hospital-related covariates of patient safety including all 20.395 rectum resections in 200 hospitals

	DEATH		POST-OPERATIVE RESPIRATORY FAILURE		RENAL FAILURE		POST-OPERATIVE WOUND INFECTION	
	OR	95% CI	OR	95% CI	OR	95% CI	OR	95% CI
**CASE COVARIATES**								
sex								
male	Ref.		Ref.		Ref.		Ref.	
female	0.842*	(0.710 - 0.998)	0.842**	(0.759 - 0.934)	0.685***	(0.613 - 0.765)	0.826***	(0.748 - 0.912)
age	1.068***	(1.059 - 1.078)	1.014***	(1.009 - 1.018)	1.021***	(1.016 - 1.026)	0.998	(0.995 - 1.002)
alcohol abuse	1.410	(0.891 - 2.231)	1.656**	(1.200 - 2.286)	1.207	(0.869 - 1.676)	1.143	(0.828 - 1.578)
blood loss anemia	0.829	(0.486 - 1.413)	0.890	(0.607 - 1.305)	1.383	(0.976 - 1.959)	1.579*	(1.113 - 2.241)
cardiac arrhythmias	1.832***	(1.535 - 2.188)	1.662***	(1.471 - 1.878)	1.639***	(1.448 - 1.854)	1.034	(0.909 - 1.176)
chronic pulmonary disease	1.303*	(1.030 - 1.648)	1.510***	(1.291 - 1.766)	1.156	(0.979 - 1.366)	1.369***	(1.175 - 1.594)
coagulopathy	4.309***	(3.600 - 5.158)	3.052***	(2.697 - 3.455)	2.886***	(2.541 - 3.277)	1.770***	(1.570 - 1.996)
congestive heart failure	1.825***	(1.481 - 2.248)	1.849***	(1.583 - 2.159)	1.536***	(1.317 - 1.792)	1.099	(0.929 - 1.300)
deficiency anemia	0.880	(0.593 - 1.304)	1.242	(0.967 - 1.595)	1.357*	(1.052 - 1.750)	1.332*	(1.043 - 1.700)
depression	0.629*	(0.439 - 0.900)	1.222*	(1.005 - 1.486)	1.290*	(1.056 - 1.577)	1.386***	(1.161 - 1.654)
diabetes, complicated	1.584**	(1.145 - 2.192)	1.045	(0.824 - 1.326)	1.441**	(1.153 - 1.801)	1.172	(0.922 - 1.490)
diabetes, uncomplicated	1.124	(0.911 - 1.388)	1.194*	(1.043 - 1.368)	1.191*	(1.036 - 1.369)	1.164*	(1.018 - 1.331)
fluid and electrolyte disorders	1.704***	(1.425 - 2.039)	2.432***	(2.181 - 2.712)	2.855***	(2.542 - 3.206)	1.952***	(1.759 - 2.166)
hypertension, complicated	0.461***	(0.324 - 0.656)	0.757*	(0.591 - 0.970)	0.836	(0.659 - 1.060)	0.911	(0.704 - 1.178)
hypertension, uncomplicated	0.584***	(0.490 - 0.695)	0.980	(0.878 - 1.093)	0.999	(0.890 - 1.121)	0.993	(0.893 - 1.104)
hypothyroidism	0.812	(0.632 - 1.043)	0.993	(0.854 - 1.155)	1.002	(0.856 - 1.174)	1.035	(0.895 - 1.198)
liver disease	4.027***	(3.155 - 5.139)	1.268*	(1.036 - 1.553)	2.312***	(1.920 - 2.783)	1.036	(0.848 - 1.267)
lymphoma	1.308	(0.563 - 3.042)	1.028	(0.532 - 1.986)	0.930	(0.476 - 1.814)	1.320	(0.718 - 2.427)
metastatic cancer	1.376**	(1.118 - 1.694)	1.211**	(1.073 - 1.367)	0.983	(0.863 - 1.121)	1.051	(0.937 - 1.179)
obesity	0.922	(0.708 - 1.202)	1.501***	(1.296 - 1.738)	1.563***	(1.348 - 1.812)	1.638***	(1.431 - 1.876)
other neurological disorders	2.306***	(1.722 - 3.089)	1.879***	(1.507 - 2.343)	1.184	(0.936 - 1.498)	1.376**	(1.098 - 1.725)
paralysis	1.153	(0.785 - 1.695)	1.391*	(1.056 - 1.833)	1.320	(0.999 - 1.744)	1.110	(0.839 - 1.469)
peripheral vascular disorders	1.908***	(1.548 - 2.353)	1.253**	(1.083 - 1.450)	1.369***	(1.181 - 1.587)	1.283***	(1.117 - 1.475)
psychoses	0.867	(0.205 - 3.672)	0.618	(0.246 - 1.551)	2.075	(0.986 - 4.369)	1.072	(0.507 - 2.264)
pulmonary circulation disorders	2.816***	(2.076 - 3.821)	1.935***	(1.522 - 2.460)	1.460**	(1.140 - 1.870)	1.208	(0.941 - 1.551)
renal failure	1.124	(0.917 - 1.377)	1.252**	(1.087 - 1.442)	1.766***	(1.544 - 2.020)	1.278***	(1.108 - 1.473)
rheumatoid arthritis/collagen vascular diseases	0.954	(0.531 - 1.713)	1.248	(0.860 - 1.812)	1.014	(0.683 - 1.507)	0.761	(0.501 - 1.158)
solid tumor without metastasis	0.599***	(0.491 - 0.731)	0.962	(0.846 - 1.094)	0.798***	(0.700 - 0.909)	1.070	(0.946 - 1.211)
valvular disease	1.000	(0.750 - 1.333)	1.075	(0.870 - 1.328)	1.042	(0.844 - 1.287)	0.893	(0.713 - 1.117)
weight loss	0.906	(0.729 - 1.127)	1.810***	(1.590 - 2.060)	1.448***	(1.268 - 1.653)	1.679***	(1.479 - 1.905)
**CARE COVARIATES**								
admission reason								
referral	Ref.		Ref.		Ref.		Ref.	
emergency case	2.028***	(1.675 - 2.454)	1.335***	(1.170 - 1.523)	1.342***	(1.169 - 1.540)	1.291***	(1.138 - 1.466)
transfer from other hospital	2.679***	(1.874 - 3.828)	1.859***	(1.406 - 2.459)	1.927***	(1.461 - 2.541)	1.484**	(1.131 - 1.948)
weekend surgery								
no	Ref.		Ref.		Ref.		Ref.	
yes	1.960***	(1.483 - 2.591)	1.427**	(1.150 - 1.770)	1.391**	(1.127 - 1.717)	0.985	(0.784 - 1.238)

colon and rectum resection								
no	Ref.		Ref.		Ref.		Ref.	
yes	2.579***	(2.163 - 3.074)	2.164***	(1.921 - 2.438)	1.859***	(1.645 - 2.100)	1.522***	(1.356 - 1.708)
**HOSPITAL COVARIATES**								
case volume	0.703***	(0.611 - 0.809)	0.844*	(0.725 - 0.982)	0.853**	(0.760 - 0.958)	0.973	(0.854 - 1.109)
area								
urban	Ref.		Ref.		Ref.		Ref.	
rural	1.072	(0.817 - 1.407)	0.904	(0.639 - 1.281)	0.834	(0.670 - 1.037)	0.854	(0.663 - 1.101)
university hospital								
no	Ref.		Ref.		Ref.		Ref.	
yes	1.616	(0.979 - 2.665)	0.853	(0.379 - 1.920)	1.299	(0.829 - 2.037)	2.292**	(1.358 - 3.869)
ownership								
public	Ref.		Ref.		Ref.		Ref.	
non-profit	0.851	(0.614 - 1.179)	1.059	(0.674 - 1.664)	0.762	(0.576 - 1.008)	0.761	(0.553 - 1.048)
private	0.925	(0.682 - 1.254)	1.267	(0.865 - 1.858)	1.597***	(1.256 - 2.030)	0.846	(0.642 - 1.117)

Note: Elixhauser Groups drug abuse, peptic ulcer disease and AIDS/ HIV were not included into the regression due to a low case numbers
*** p<0.001
** p <0.01
* p<0.05

For peer review only

S11: Multivariate analysis of case-, care- and hospital-related covariates of patient safety including 25.805 colon cancer resections in 201 hospitals

	DEATH		POST-OPERATIVE RESPIRATORY FAILURE		RENAL FAILURE		POST-OPERATIVE WOUND INFECTION	
	OR	95% CI	OR	95% CI	OR	95% CI	OR	95% CI
**CASE COVARIATES**								
sex								
male	Ref.		Ref		Ref		Ref	
female	0.867**	(0.780 - 0.963)	0.810***	(0.747 - 0.878)	0.623***	(0.572 - 0.678)	0.879**	(0.807 - 0.959)
age	1.039***	(1.034 - 1.045)	1.008***	(1.005 - 1.012)	1.018***	(1.014 - 1.022)	0.998	(0.994 - 1.002)
alcohol abuse	1.448*	(1.087 - 1.928)	1.273	(0.982 - 1.650)	1.297*	(1.006 - 1.672)	1.222	(0.919 - 1.624)
blood loss anemia	0.552***	(0.405 - 0.752)	0.926	(0.743 - 1.153)	0.720**	(0.576 - 0.899)	1.199	(0.953 - 1.507)
cardiac arrhythmias	1.616***	(1.440 - 1.814)	1.560***	(1.422 - 1.712)	1.639***	(1.492 - 1.800)	1.229***	(1.108 - 1.363)
chronic pulmonary disease	1.329***	(1.142 - 1.546)	1.450***	(1.288 - 1.632)	1.059	(0.931 - 1.204)	1.213**	(1.064 - 1.384)
coagulopathy	4.057***	(3.617 - 4.550)	3.121***	(2.837 - 3.434)	3.066***	(2.780 - 3.383)	1.679***	(1.515 - 1.861)
congestive heart failure	1.766***	(1.539 - 2.026)	1.848***	(1.649 - 2.071)	1.629***	(1.453 - 1.827)	1.143*	(1.000 - 1.306)
deficiency anemia	0.687***	(0.550 - 0.858)	1.027	(0.880 - 1.198)	0.894	(0.756 - 1.057)	0.945	(0.793 - 1.126)
depression	0.760*	(0.610 - 0.946)	1.409***	(1.212 - 1.637)	1.053	(0.891 - 1.243)	1.557***	(1.336 - 1.813)
diabetes, complicated	1.213	(0.986 - 1.491)	0.969	(0.817 - 1.149)	1.351***	(1.150 - 1.587)	1.077	(0.891 - 1.302)
diabetes, uncomplicated	1.100	(0.965 - 1.253)	1.125*	(1.017 - 1.243)	1.090	(0.982 - 1.210)	1.074	(0.963 - 1.198)
fluid and electrolyte disorders	1.873***	(1.670 - 2.100)	2.338***	(2.145 - 2.547)	2.755***	(2.512 - 3.021)	2.038***	(1.857 - 2.236)
hypertension, complicated	0.511***	(0.409 - 0.639)	0.846	(0.711 - 1.008)	0.795*	(0.667 - 0.947)	0.941	(0.770 - 1.151)
hypertension, uncomplicated	0.624***	(0.559 - 0.698)	0.960	(0.881 - 1.047)	0.996	(0.909 - 1.090)	0.997	(0.909 - 1.094)
hypothyroidism	0.879	(0.752 - 1.028)	1.071	(0.955 - 1.201)	1.018	(0.900 - 1.151)	1.200**	(1.064 - 1.354)
liver disease	3.393***	(2.941 - 3.914)	1.375***	(1.202 - 1.571)	2.346***	(2.065 - 2.664)	0.906	(0.777 - 1.056)
lymphoma	0.816	(0.463 - 1.437)	0.766	(0.477 - 1.231)	1.366	(0.891 - 2.093)	1.283	(0.798 - 2.063)
metastatic cancer	1.414***	(1.273 - 1.571)	1.089*	(1.005 - 1.181)	0.986	(0.905 - 1.073)	1.053	(0.966 - 1.148)
obesity	0.745**	(0.621 - 0.894)	1.123	(0.994 - 1.269)	1.431***	(1.266 - 1.616)	1.513***	(1.337 - 1.713)
other neurological disorders	1.337**	(1.080 - 1.655)	1.818***	(1.532 - 2.157)	1.512***	(1.263 - 1.810)	1.214	(0.997 - 1.479)
paralysis	1.754***	(1.367 - 2.250)	1.402**	(1.135 - 1.733)	1.114	(0.889 - 1.395)	1.209	(0.951 - 1.537)
peripheral vascular disorders	1.644***	(1.420 - 1.904)	1.295***	(1.144 - 1.465)	1.290***	(1.138 - 1.462)	1.217**	(1.068 - 1.387)
psychoses	1.170	(0.607 - 2.253)	1.520	(0.948 - 2.437)	1.222	(0.731 - 2.045)	1.359	(0.820 - 2.252)
pulmonary circulation disorders	1.840***	(1.511 - 2.240)	1.977***	(1.675 - 2.333)	1.360***	(1.141 - 1.620)	1.088	(0.898 - 1.318)
renal failure	1.248***	(1.098 - 1.420)	1.162**	(1.047 - 1.289)	1.538***	(1.389 - 1.702)	1.143*	(1.018 - 1.282)
rheumatoid arthritis/collagen vascular diseases	0.883	(0.559 - 1.395)	0.647*	(0.441 - 0.949)	0.894	(0.626 - 1.277)	0.884	(0.599 - 1.305)
solid tumor without metastasis	0.329***	(0.212 - 0.511)	0.806	(0.520 - 1.252)	0.507**	(0.332 - 0.773)	1.526	(0.894 - 2.605)
valvular disease	0.671***	(0.548 - 0.821)	0.875	(0.747 - 1.024)	0.914	(0.781 - 1.069)	0.920	(0.769 - 1.100)
weight loss	1.007	(0.882 - 1.150)	1.586***	(1.434 - 1.753)	1.366***	(1.233 - 1.514)	1.583***	(1.421 - 1.763)
**CARE COVARIATES**								
admission reason								
referral	Ref.		Ref.		Ref.		Ref.	
emergency case	1.822***	(1.613 - 2.058)	1.202***	(1.089 - 1.326)	1.407***	(1.266 - 1.564)	1.091	(0.983 - 1.211)
transfer from other hospital	2.491***	(1.962 - 3.161)	1.697***	(1.371 - 2.101)	1.556***	(1.247 - 1.941)	1.389**	(1.112 - 1.734)
weekend surgery								
no	Ref.		Ref.		Ref.		Ref.	
yes	1.949***	(1.650 - 2.303)	1.377***	(1.187 - 1.598)	1.578***	(1.365 - 1.824)	1.049	(0.885 - 1.244)

total colon resection								
no	Ref.		Ref.		Ref.		Ref.	
yes	2.046***	(1.669 - 2.509)	1.691***	(1.420 - 2.014)	1.703***	(1.422 - 2.038)	1.182	(0.986 - 1.417)
colon and rectum resection								
no	Ref.		Ref.		Ref.		Ref.	
yes	1.025	(0.853 - 1.232)	1.617***	(1.422 - 1.837)	1.394***	(1.214 - 1.600)	1.753***	(1.542 - 1.993)
**HOSPITAL COVARIATES**								
case volume	0.926	(0.810 - 1.058)	0.939	(0.795 - 1.110)	0.994	(0.865 - 1.142)	1.146	(0.975 - 1.348)
area								
urban	Ref.		Ref.		Ref.		Ref.	
rural	1.042	(0.857 - 1.267)	0.815	(0.605 - 1.099)	0.793*	(0.639 - 0.983)	0.964	(0.750 - 1.239)
university hospital								
no	Ref.		Ref.		Ref.		Ref.	
yes	1.301	(0.877 - 1.931)	0.650	(0.318 - 1.328)	1.280	(0.790 - 2.076)	2.209**	(1.281 - 3.807)
ownership								
public	Ref.		Ref.		Ref.		Ref.	
non-profit	1.146	(0.898 - 1.463)	1.098	(0.745 - 1.618)	0.876	(0.660 - 1.162)	0.767	(0.557 - 1.056)
private	1.207	(0.970 - 1.502)	1.316	(0.951 - 1.821)	1.993***	(1.572 - 2.526)	0.776	(0.591 - 1.018)

Note: Elixhauser Groups drug abuse, peptic ulcer disease and AIDS/ HIV were not included into the regression due to a low case numbers
*** $p<0.001$
** $p <0.01$
* $p<0.05$

For peer review only

S12: Multivariate analysis of case-, care- and hospital-related covariates of patient safety including 13.703 rectum cancer resections in 197 hospitals

	DEATH		POST-OPERATIVE RESPIRATORY FAILURE		RENAL FAILURE		POST-OPERATIVE WOUND INFECTION	
	OR	95% CI	OR	95% CI	OR	95% CI	OR	95% CI
**CASE COVARIATES**								
sex								
male	Ref.		Ref.		Ref.		Ref.	
female	0.912	(0.738 - 1.127)	0.892	(0.789 - 1.009)	0.685***	(0.599 - 0.783)	0.888	(0.789 - 1.000)
age	1.066***	(1.054 - 1.079)	1.013***	(1.007 - 1.019)	1.019***	(1.013 - 1.026)	0.998	(0.993 - 1.003)
alcohol abuse	1.807*	(1.034 - 3.156)	1.603*	(1.076 - 2.389)	1.156	(0.770 - 1.736)	1.138	(0.768 - 1.687)
blood loss anemia	0.823	(0.426 - 1.589)	0.677	(0.415 - 1.102)	1.231	(0.812 - 1.867)	1.466	(0.961 - 2.236)
cardiac arrhythmias	1.704***	(1.361 - 2.135)	1.714***	(1.484 - 1.981)	1.628***	(1.402 - 1.890)	1.050	(0.901 - 1.223)
chronic pulmonary disease	1.540**	(1.149 - 2.065)	1.473***	(1.218 - 1.781)	1.209	(0.986 - 1.482)	1.389***	(1.153 - 1.673)
coagulopathy	3.691***	(2.941 - 4.632)	2.783***	(2.396 - 3.233)	2.487***	(2.126 - 2.909)	1.723***	(1.493 - 1.990)
congestive heart failure	1.784***	(1.363 - 2.335)	1.798***	(1.488 - 2.172)	1.557***	(1.287 - 1.885)	1.028	(0.837 - 1.262)
deficiency anemia	1.008	(0.631 - 1.610)	1.404*	(1.049 - 1.879)	1.527**	(1.129 - 2.066)	1.269	(0.945 - 1.703)
depression	0.665	(0.418 - 1.058)	1.299*	(1.026 - 1.646)	1.359*	(1.060 - 1.742)	1.449***	(1.170 - 1.793)
diabetes, complicated	1.285	(0.830 - 1.990)	1.061	(0.798 - 1.409)	1.190	(0.906 - 1.562)	1.146	(0.860 - 1.528)
diabetes, uncomplicated	1.060	(0.817 - 1.375)	1.194*	(1.022 - 1.396)	1.075	(0.913 - 1.267)	1.243**	(1.067 - 1.448)
fluid and electrolyte disorders	1.693***	(1.353 - 2.117)	2.162***	(1.903 - 2.456)	2.720***	(2.367 - 3.126)	1.966***	(1.738 - 2.223)
hypertension, complicated	0.590*	(0.379 - 0.918)	0.844	(0.627 - 1.137)	0.844	(0.630 - 1.130)	0.894	(0.652 - 1.226)
hypertension, uncomplicated	0.625***	(0.502 - 0.780)	0.994	(0.873 - 1.132)	1.106	(0.962 - 1.272)	0.995	(0.877 - 1.129)
hypothyroidism	0.878	(0.633 - 1.217)	1.032	(0.860 - 1.239)	0.965	(0.791 - 1.177)	0.995	(0.832 - 1.191)
liver disease	3.342***	(2.461 - 4.538)	1.126	(0.879 - 1.443)	2.363***	(1.894 - 2.949)	1.171	(0.924 - 1.486)
lymphoma	1.440	(0.468 - 4.431)	1.006	(0.429 - 2.360)	1.225	(0.519 - 2.893)	1.918	(0.891 - 4.126)
metastatic cancer	1.280*	(1.035 - 1.581)	1.212**	(1.071 - 1.371)	1.015	(0.889 - 1.158)	1.041	(0.925 - 1.172)
obesity	0.953	(0.684 - 1.330)	1.476***	(1.241 - 1.755)	1.607***	(1.349 - 1.915)	1.570***	(1.333 - 1.849)
other neurological disorders	2.294***	(1.562 - 3.369)	2.146***	(1.634 - 2.818)	1.198	(0.885 - 1.621)	1.598**	(1.207 - 2.116)
paralysis	1.418	(0.866 - 2.321)	1.422*	(1.014 - 1.993)	1.249	(0.878 - 1.776)	1.130	(0.799 - 1.600)
peripheral vascular disorders	1.561**	(1.190 - 2.047)	1.151	(0.970 - 1.367)	1.259*	(1.055 - 1.503)	1.423***	(1.214 - 1.669)
psychoses	0.417	(0.045 - 3.841)	0.477	(0.147 - 1.541)	1.904	(0.756 - 4.792)	1.012	(0.413 - 2.481)
pulmonary circulation disorders	3.547***	(2.491 - 5.049)	2.146***	(1.633 - 2.819)	1.567**	(1.179 - 2.082)	1.234	(0.923 - 1.650)
renal failure	0.942	(0.721 - 1.231)	1.125	(0.949 - 1.334)	1.750***	(1.489 - 2.056)	1.233*	(1.039 - 1.462)
rheumatoid arthritis/collagen vascular diseases	1.041	(0.443 - 2.445)	0.825	(0.462 - 1.476)	0.962	(0.544 - 1.701)	0.600	(0.312 - 1.154)
solid tumor without metastasis	0.105***	(0.039 - 0.282)	0.960	(0.353 - 2.613)	1.074	(0.327 - 3.534)	1.648	(0.542 - 5.013)
valvular disease	0.808	(0.550 - 1.185)	1.099	(0.855 - 1.413)	0.922	(0.712 - 1.193)	0.969	(0.743 - 1.265)
weight loss	0.967	(0.745 - 1.255)	1.761***	(1.517 - 2.045)	1.333***	(1.142 - 1.558)	1.557***	(1.345 - 1.803)
**CARE COVARIATES**								
admission reason								
referral	Ref.		Ref.		Ref.		Ref.	
emergency case	1.937***	(1.526 - 2.460)	1.199*	(1.018 - 1.413)	1.257*	(1.055 - 1.498)	1.468***	(1.255 - 1.718)
transfer from other hospital	2.503***	(1.487 - 4.213)	2.160***	(1.497 - 3.118)	1.731**	(1.180 - 2.540)	1.805**	(1.263 - 2.578)
weekend surgery								
no	Ref.		Ref.		Ref.		Ref.	
yes	2.137***	(1.417 - 3.224)	1.384*	(1.024 - 1.870)	1.267	(0.940 - 1.708)	1.148	(0.845 - 1.560)

colon and rectum resection								
no	Ref.		Ref.		Ref.		Ref.	
yes	2.422***	(1.940 - 3.024)	2.085***	(1.806 - 2.408)	1.711***	(1.470 - 1.991)	1.627***	(1.415 - 1.871)
**HOSPITAL COVARIATES**								
case volume	0.678***	(0.577 - 0.796)	0.907	(0.764 - 1.075)	0.835**	(0.730 - 0.955)	0.988	(0.851 - 1.147)
area								
urban	Ref.		Ref.		Ref.		Ref.	
rural	1.164	(0.862 - 1.572)	0.951	(0.661 - 1.369)	0.808	(0.632 - 1.033)	0.843	(0.638 - 1.113)
university hospital								
no	Ref.		Ref.		Ref.		Ref.	
yes	1.512	(0.889 - 2.571)	0.807	(0.355 - 1.837)	1.374	(0.840 - 2.246)	2.195**	(1.267 - 3.801)
ownership								
public	Ref.		Ref.		Ref.		Ref.	
non-profit	1.034	(0.725 - 1.473)	1.089	(0.682 - 1.740)	0.797	(0.580 - 1.095)	0.733	(0.518 - 1.037)
private	0.900	(0.635 - 1.276)	1.238	(0.828 - 1.850)	1.807***	(1.375 - 2.376)	0.853	(0.629 - 1.158)

Note: Elixhauser Groups drug abuse, peptic ulcer disease and AIDS/ HIV were not included into the regression due to a low case numbers
*** p<0.001
** p <0.01
* p<0.05

For peer review only

S13: Multivariate analysis of case-, care- and hospital-related covariates of patient safety including 28.363 non-cancer colon resections in 208 hospitals

	DEATH		POST-OPERATIVE RESPIRATORY FAILURE		RENAL FAILURE		POST-OPERATIVE WOUND INFECTION	
	OR	95% CI	OR	95% CI	OR	95% CI	OR	95% CI
**CASE COVARIATES**								
sex								
male	Ref.		Ref		Ref		Ref	
female	0.988	(0.896 - 1.090)	0.746***	(0.691 - 0.807)	0.748***	(0.689 - 0.811)	0.872***	(0.804 - 0.946)
age	1.056***	(1.051 - 1.060)	1.016***	(1.014 - 1.019)	1.026***	(1.023 - 1.030)	0.997	(0.995 - 1.000)
alcohol abuse	1.483***	(1.190 - 1.847)	2.400***	(1.985 - 2.902)	1.745***	(1.435 - 2.121)	1.350**	(1.099 - 1.657)
blood loss anemia	0.388***	(0.246 - 0.612)	1.296	(0.945 - 1.777)	1.140	(0.831 - 1.565)	1.150	(0.828 - 1.597)
cardiac arrhythmias	1.666***	(1.499 - 1.851)	1.627***	(1.487 - 1.781)	1.605***	(1.464 - 1.760)	1.166**	(1.053 - 1.291)
chronic pulmonary disease	1.405***	(1.231 - 1.603)	1.463***	(1.310 - 1.633)	1.154*	(1.028 - 1.296)	1.264***	(1.123 - 1.422)
coagulopathy	4.166***	(3.747 - 4.632)	2.920***	(2.665 - 3.198)	3.515***	(3.209 - 3.851)	1.602***	(1.451 - 1.769)
congestive heart failure	1.764***	(1.562 - 1.993)	1.972***	(1.770 - 2.197)	1.733***	(1.555 - 1.932)	1.282***	(1.135 - 1.448)
deficiency anemia	0.943	(0.732 - 1.214)	1.219	(0.995 - 1.493)	1.428***	(1.166 - 1.749)	1.188	(0.967 - 1.459)
depression	0.582***	(0.472 - 0.717)	1.488***	(1.288 - 1.719)	1.071	(0.918 - 1.249)	1.564***	(1.354 - 1.807)
diabetes, complicated	1.332**	(1.105 - 1.606)	1.430***	(1.211 - 1.688)	1.187*	(1.005 - 1.404)	1.326**	(1.104 - 1.593)
diabetes, uncomplicated	1.170*	(1.026 - 1.334)	1.304***	(1.172 - 1.450)	1.226***	(1.098 - 1.368)	1.172**	(1.046 - 1.314)
fluid and electrolyte disorders	1.828***	(1.641 - 2.037)	2.715***	(2.495 - 2.955)	2.930***	(2.681 - 3.202)	1.929***	(1.764 - 2.110)
hypertension, complicated	0.527***	(0.430 - 0.645)	0.680***	(0.570 - 0.812)	0.847	(0.712 - 1.008)	0.965	(0.794 - 1.172)
hypertension, uncomplicated	0.489***	(0.440 - 0.543)	0.933	(0.858 - 1.015)	0.861***	(0.789 - 0.940)	1.107*	(1.011 - 1.210)
hypothyroidism	0.729***	(0.631 - 0.842)	1.152*	(1.034 - 1.283)	1.064	(0.949 - 1.192)	1.210***	(1.081 - 1.354)
liver disease	5.708***	(4.967 - 6.559)	1.408***	(1.238 - 1.601)	2.827***	(2.491 - 3.207)	0.802**	(0.694 - 0.925)
lymphoma	1.016	(0.683 - 1.513)	0.858	(0.602 - 1.223)	0.895	(0.624 - 1.284)	0.978	(0.668 - 1.433)
metastatic cancer	-		-		-		-	
obesity	0.768***	(0.657 - 0.897)	1.180**	(1.055 - 1.321)	1.557***	(1.390 - 1.745)	1.737***	(1.557 - 1.938)
other neurological disorders	1.612***	(1.360 - 1.912)	1.949***	(1.686 - 2.253)	1.498***	(1.289 - 1.740)	1.313***	(1.123 - 1.536)
paralysis	1.284*	(1.039 - 1.587)	1.482***	(1.237 - 1.777)	1.124	(0.930 - 1.358)	1.238*	(1.021 - 1.500)
peripheral vascular disorders	2.129***	(1.886 - 2.405)	1.626***	(1.458 - 1.813)	1.675***	(1.500 - 1.871)	1.266***	(1.125 - 1.426)
psychoses	0.518*	(0.279 - 0.963)	2.462***	(1.639 - 3.698)	0.660	(0.405 - 1.074)	1.516	(0.994 - 2.313)
pulmonary circulation disorders	1.348**	(1.094 - 1.662)	1.737***	(1.445 - 2.088)	1.296**	(1.070 - 1.570)	1.040	(0.849 - 1.274)
renal failure	1.381***	(1.228 - 1.553)	1.073	(0.969 - 1.189)	1.494***	(1.351 - 1.653)	0.996	(0.889 - 1.117)
rheumatoid arthritis/collagen vascular diseases	1.046	(0.791 - 1.384)	1.120	(0.885 - 1.416)	0.981	(0.770 - 1.250)	1.143	(0.893 - 1.462)
solid tumor without metastasis	-		-		-		-	
valvular disease	0.954	(0.803 - 1.133)	0.861	(0.738 - 1.004)	0.944	(0.810 - 1.102)	0.942	(0.795 - 1.116)
weight loss	0.799**	(0.696 - 0.917)	1.717***	(1.543 - 1.910)	1.520***	(1.365 - 1.693)	2.013***	(1.803 - 2.248)
**CARE COVARIATES**								
admission reason								
referral	Ref.		Ref.		Ref.		Ref.	
emergency case	1.936***	(1.706 - 2.197)	1.727***	(1.564 - 1.906)	1.479***	(1.330 - 1.645)	1.197***	(1.086 - 1.320)
transfer from other hospital	2.610***	(2.169 - 3.140)	2.299***	(1.959 - 2.698)	2.027***	(1.718 - 2.391)	1.202*	(1.015 - 1.423)
weekend surgery								
no	Ref.		Ref.		Ref.		Ref.	
yes	1.577***	(1.397 - 1.780)	1.436***	(1.296 - 1.590)	1.443***	(1.299 - 1.604)	1.095	(0.978 - 1.225)

total colon resection								
no	Ref.		Ref.		Ref.		Ref.	
yes	3.173***	(2.705 - 3.723)	1.604***	(1.395 - 1.843)	2.545***	(2.213 - 2.927)	0.958	(0.830 - 1.107)
colon and rectum resection								
no	Ref.		Ref.		Ref.		Ref.	
yes	1.159	(0.936 - 1.436)	1.320**	(1.116 - 1.562)	1.404***	(1.183 - 1.667)	1.314**	(1.107 - 1.560)
**HOSPITAL COVARIATES**								
case volume	0.961	(0.844 - 1.094)	0.876	(0.753 - 1.018)	0.950	(0.841 - 1.073)	1.135	(0.987 - 1.304)
area								
urban	Ref.		Ref.		Ref.		Ref.	
rural	1.091	(0.886 - 1.345)	0.918	(0.682 - 1.235)	0.775*	(0.631 - 0.953)	1.058	(0.837 - 1.336)
university hospital								
no	Ref.		Ref.		Ref.		Ref.	
yes	1.336	(0.855 - 2.087)	0.712	(0.347 - 1.461)	1.586	(0.999 - 2.520)	1.802*	(1.070 - 3.034)
ownership								
public	Ref.		Ref.		Ref.		Ref.	
non-profit	0.900	(0.688 - 1.177)	1.028	(0.696 - 1.519)	0.836	(0.638 - 1.096)	0.736*	(0.543 - 0.996)
private	1.284*	(1.015 - 1.626)	1.433*	(1.035 - 1.986)	1.893***	(1.505 - 2.380)	0.775	(0.601 - 1.001)

Note: Elixhauser Groups drug abuse, peptic ulcer disease and AIDS/ HIV were not included into the regression due to a low case numbers

*** p<0.001

** p <0.01

* p<0.05

For peer review only

S14: Multivariate analysis of case-, care- and hospital-related covariates of patient safety including 6.692 non-cancer rectum resections in 195 hospitals

	**DEATH**		**POST-OPERATIVE RESPIRATORY FAILURE**		**RENAL FAILURE**		**POST-OPERATIVE WOUND INFECTION**	
	OR	95% CI	OR	95% CI	OR	95% CI	OR	95% CI
**CASE COVARIATES**								
sex								
male	Ref.		Ref		Ref		Ref	
female	0.733*	(0.542 - 0.992)	0.739**	(0.601 - 0.908)	0.746**	(0.607 - 0.917)	0.651***	(0.539 - 0.786)
age	1.074***	(1.058 - 1.089)	1.013***	(1.006 - 1.021)	1.023***	(1.016 - 1.031)	1.000	(0.994 - 1.006)
alcohol abuse	0.809	(0.348 - 1.882)	1.801*	(1.008 - 3.219)	1.355	(0.754 - 2.434)	1.080	(0.601 - 1.941)
blood loss anemia	0.797	(0.305 - 2.082)	1.735	(0.877 - 3.432)	1.886	(0.970 - 3.668)	1.927*	(1.014 - 3.663)
cardiac arrhythmias	2.130***	(1.569 - 2.892)	1.573***	(1.243 - 1.990)	1.608***	(1.281 - 2.017)	1.015	(0.795 - 1.295)
chronic pulmonary disease	0.908	(0.601 - 1.371)	1.570**	(1.180 - 2.088)	0.977	(0.726 - 1.315)	1.402*	(1.066 - 1.845)
coagulopathy	5.767***	(4.191 - 7.937)	3.713***	(2.934 - 4.697)	3.703***	(2.949 - 4.650)	1.947***	(1.550 - 2.447)
congestive heart failure	1.851***	(1.309 - 2.617)	1.887***	(1.426 - 2.497)	1.543**	(1.180 - 2.017)	1.269	(0.943 - 1.708)
deficiency anemia	0.765	(0.362 - 1.616)	1.008	(0.606 - 1.675)	1.039	(0.640 - 1.689)	1.430	(0.911 - 2.244)
depression	0.591	(0.326 - 1.073)	1.043	(0.723 - 1.505)	1.220	(0.859 - 1.732)	1.208	(0.869 - 1.679)
diabetes, complicated	2.158**	(1.267 - 3.674)	0.990	(0.627 - 1.564)	2.155***	(1.423 - 3.265)	1.292	(0.821 - 2.032)
diabetes, uncomplicated	1.233	(0.838 - 1.815)	1.133	(0.849 - 1.513)	1.549**	(1.176 - 2.038)	0.950	(0.709 - 1.272)
fluid and electrolyte disorders	1.619**	(1.185 - 2.213)	3.048***	(2.458 - 3.779)	3.006***	(2.430 - 3.719)	1.944***	(1.594 - 2.370)
hypertension, complicated	0.345***	(0.189 - 0.628)	0.657	(0.419 - 1.031)	0.910	(0.596 - 1.390)	1.042	(0.663 - 1.638)
hypertension, uncomplicated	0.511***	(0.377 - 0.694)	0.928	(0.751 - 1.147)	0.813	(0.658 - 1.003)	1.041	(0.851 - 1.273)
hypothyroidism	0.742	(0.491 - 1.121)	0.919	(0.695 - 1.214)	1.051	(0.803 - 1.376)	1.093	(0.845 - 1.416)
liver disease	5.966***	(3.793 - 9.384)	1.550*	(1.068 - 2.252)	2.060***	(1.451 - 2.925)	0.795	(0.540 - 1.172)
lymphoma	0.685	(0.151 - 3.108)	0.930	(0.309 - 2.800)	0.656	(0.221 - 1.944)	0.884	(0.308 - 2.539)
metastatic cancer	-		-		-		-	
obesity	0.853	(0.538 - 1.352)	1.534**	(1.152 - 2.043)	1.400*	(1.054 - 1.861)	1.871***	(1.460 - 2.396)
other neurological disorders	2.558***	(1.586 - 4.126)	1.428	(0.968 - 2.106)	1.117	(0.755 - 1.652)	1.113	(0.754 - 1.642)
paralysis	0.798	(0.421 - 1.514)	1.276	(0.780 - 2.088)	1.508	(0.940 - 2.421)	1.158	(0.714 - 1.881)
peripheral vascular disorders	2.505***	(1.743 - 3.599)	1.502**	(1.119 - 2.018)	1.629***	(1.227 - 2.163)	1.048	(0.774 - 1.419)
psychoses	1.703	(0.166 - 17.483)	1.385	(0.317 - 6.052)	3.384	(0.925 - 12.382)	1.386	(0.359 - 5.356)
pulmonary circulation disorders	1.770	(0.965 - 3.246)	1.636	(0.983 - 2.725)	1.114	(0.667 - 1.859)	1.203	(0.725 - 1.997)
renal failure	1.472*	(1.047 - 2.068)	1.633***	(1.252 - 2.129)	1.755***	(1.368 - 2.250)	1.395*	(1.071 - 1.818)
rheumatoid arthritis/collagen vascular diseases	0.864	(0.358 - 2.087)	1.948*	(1.171 - 3.242)	1.083	(0.609 - 1.925)	1.001	(0.571 - 1.754)
solid tumor without metastasis	-		-		-		-	
valvular disease	1.236	(0.771 - 1.982)	0.999	(0.671 - 1.488)	1.300	(0.888 - 1.903)	0.719	(0.469 - 1.104)
weight loss	0.761	(0.507 - 1.143)	1.969***	(1.503 - 2.580)	1.944***	(1.504 - 2.513)	2.204***	(1.709 - 2.843)
**CARE COVARIATES**								
admission reason								
referral	Ref.		Ref.		Ref.		Ref.	
emergency case	2.060***	(1.470 - 2.885)	1.590***	(1.253 - 2.018)	1.382**	(1.094 - 1.746)	0.992	(0.797 - 1.234)
transfer from other hospital	2.583***	(1.488 - 4.487)	1.510	(0.962 - 2.369)	1.949**	(1.269 - 2.993)	1.102	(0.713 - 1.704)
weekend surgery								
no	Ref.		Ref.		Ref.		Ref.	
yes	1.770**	(1.179 - 2.658)	1.391*	(1.012 - 1.912)	1.499*	(1.101 - 2.042)	0.870	(0.613 - 1.235)

colon and rectum resection								
no	Ref.		Ref.		Ref.		Ref.	
yes	2.967***	(2.184 - 4.030)	2.183***	(1.750 - 2.725)	2.190***	(1.771 - 2.709)	1.318*	(1.066 - 1.630)
**HOSPITAL COVARIATES**								
case volume	0.777*	(0.614 - 0.983)	0.793*	(0.642 - 0.978)	0.863	(0.733 - 1.015)	0.956	(0.795 - 1.150)
area								
urban	Ref.		Ref.		Ref.		Ref.	
rural	0.851	(0.536 - 1.351)	0.881	(0.580 - 1.337)	0.872	(0.646 - 1.176)	0.865	(0.613 - 1.220)
university hospital								
no	Ref.		Ref.		Ref.		Ref.	
yes	1.529	(0.712 - 3.283)	0.849	(0.354 - 2.034)	1.176	(0.701 - 1.972)	2.375**	(1.316 - 4.287)
ownership								
public	Ref.		Ref.		Ref.		Ref.	
non-profit	0.556*	(0.323 - 0.959)	1.098	(0.652 - 1.849)	0.702	(0.489 - 1.008)	0.775	(0.516 - 1.166)
private	0.981	(0.594 - 1.621)	1.614*	(1.020 - 2.556)	1.331	(0.960 - 1.846)	0.791	(0.546 - 1.147)

Note: Elixhauser Groups drug abuse, peptic ulcer disease and AIDS/ HIV were not included into the regression due to a low case numbers
*** p<0.001
** p <0.01
* p<0.05

For peer review only

S15 – Figure: Interactions between emergency admission and university hospital status

S16 – Figure: Interactions between transfer from other hospital admission and university hospital status

For peer review only

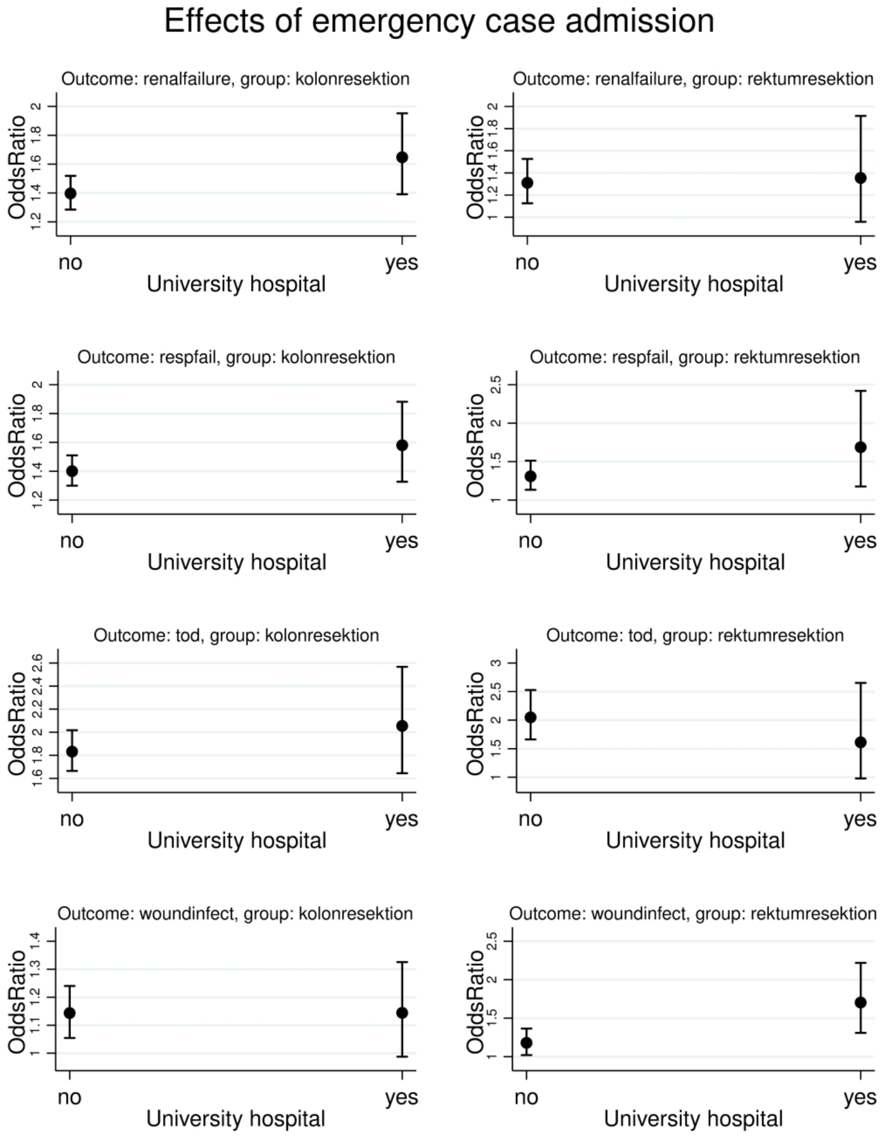

S15 - Figure: Interactions between emergency admission and university hospital status

406x508mm (300 x 300 DPI)

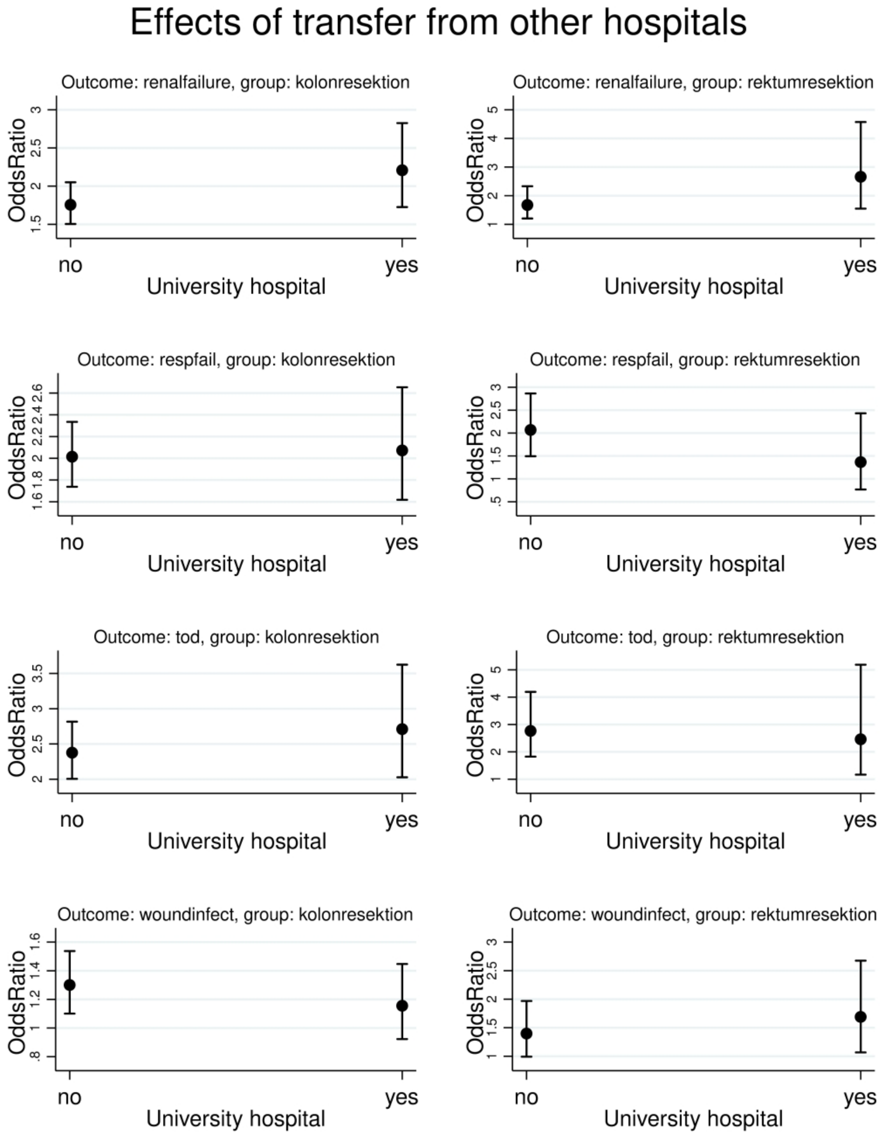

S16 - Figure: Interactions between transfer from other hospital admission and university hospital status

406x508mm (300 x 300 DPI)

Reporting checklist for cross sectional study.

Based on the STROBE cross sectional guidelines.

Instructions to authors

Complete this checklist by entering the page numbers from your manuscript where readers will find each of the items listed below.

Your article may not currently address all the items on the checklist. Please modify your text to include the missing information. If you are certain that an item does not apply, please write "n/a" and provide a short explanation.

Upload your completed checklist as an extra file when you submit to a journal.

In your methods section, say that you used the STROBE cross sectionalreporting guidelines, and cite them as:

von Elm E, Altman DG, Egger M, Pocock SJ, Gotzsche PC, Vandenbroucke JP. The Strengthening the Reporting of Observational Studies in Epidemiology (STROBE) Statement: guidelines for reporting observational studies.

		Reporting Item	Page Number
**Title and abstract**			
Title	#1a	Indicate the study's design with a commonly used term in the title or the abstract	2
Abstract	#1b	Provide in the abstract an informative and balanced summary of what was done and what was found	2
**Introduction**			
Background / rationale	#2	Explain the scientific background and rationale for the investigation being reported	4
Objectives	#3	State specific objectives, including any prespecified hypotheses	4
**Methods**			
Study design	#4	Present key elements of study design early in the paper	4
Setting	#5	Describe the setting, locations, and relevant dates, including periods of recruitment, exposure, follow-up, and data collection	5
Eligibility criteria	#6a	Give the eligibility criteria, and the sources and methods of selection of participants.	4-6
	#7	Clearly define all outcomes, exposures, predictors, potential confounders, and effect modifiers. Give diagnostic criteria, if applicable	5-6
Data sources / measurement	#8	For each variable of interest give sources of data and details of methods of assessment (measurement). Describe comparability of assessment methods	5

For peer review only

		if there is more than one group. Give information separately for for exposed and unexposed groups if applicable.	
Bias	#9	Describe any efforts to address potential sources of bias	6-7
Study size	#10	Explain how the study size was arrived at	5-6
Quantitative variables	#11	Explain how quantitative variables were handled in the analyses. If applicable, describe which groupings were chosen, and why	6-7
Statistical methods	#12a	Describe all statistical methods, including those used to control for confounding	6-7
Statistical methods	#12b	Describe any methods used to examine subgroups and interactions	6-7
Statistical methods	#12c	Explain how missing data were addressed	N/A
Statistical methods	#12d	If applicable, describe analytical methods taking account of sampling strategy	N/A
Statistical methods	#12e	Describe any sensitivity analyses	7
**Results**			
Participants	#13a	Report numbers of individuals at each stage of study—eg numbers potentially eligible, examined for eligibility, confirmed eligible, included in the study, completing follow-up, and analysed. Give information separately for for exposed and unexposed groups if applicable.	7
Participants	#13b	Give reasons for non-participation at each stage	N/A
Participants	#13c	Consider use of a flow diagram	7/ Fig 1
Descriptive data	#14a	Give characteristics of study participants (eg demographic, clinical, social) and information on exposures and potential confounders. Give information separately for exposed and unexposed groups if applicable.	7-8
Descriptive data	#14b	Indicate number of participants with missing data for each variable of interest	N/A
Outcome data	#15	Report numbers of outcome events or summary measures. Give information separately for exposed and unexposed groups if applicable.	7-8 / Fig 1
Main results	#16a	Give unadjusted estimates and, if applicable, confounder-adjusted estimates and their precision (eg, 95% confidence interval). Make clear which confounders were adjusted for and why they were included	10-11 / Table 2-3, Supp. 7-16)
Main results	#16b	Report category boundaries when continuous variables were categorized	N/A
Main results	#16c	If relevant, consider translating estimates of relative risk into absolute risk for a meaningful time period	N/A

For peer review only

Other analyses	#17	Report other analyses done—e.g., analyses of subgroups and interactions, and sensitivity analyses	11
**Discussion**			
Key results	#18	Summarise key results with reference to study objectives	14
Limitations	#19	Discuss limitations of the study, taking into account sources of potential bias or imprecision. Discuss both direction and magnitude of any potential bias.	14-15
Interpretation	#20	Give a cautious overall interpretation considering objectives, limitations, multiplicity of analyses, results from similar studies, and other relevant evidence.	14-15
Generalisability	#21	Discuss the generalisability (external validity) of the study results	14-15
**Other Information**			
Funding	#22	Give the source of funding and the role of the funders for the present study and, if applicable, for the original study on which the present article is based	16

Notes:

- 15: 7-8 / Fig 1
- 16a: 10-11 / Table 2-3, Supp. 7-16) The STROBE checklist is distributed under the terms of the Creative Commons Attribution License CC-BY. This checklist was completed on 15. October 2021 using https://www.goodreports.org/, a tool made by the EQUATOR Network in collaboration with Penelope.ai

For peer review only

BMJ Open

Healthcare-related risk factors are more relevant for patient safety than hospital-related factors with regard to colorectal resections - cross-sectional evidence from a nationwide sample of 232 German hospitals

Journal:	*BMJ Open*
Manuscript ID	bmjopen-2021-058481.R1
Article Type:	Original research
Date Submitted by the Author:	24-May-2022
Complete List of Authors:	Walther, Felix; TU Dresden Faculty of Medicine Carl Gustav Carus, Center for Evidence-based Healthcare; University Hospital Carl Gustav Carus, Quality and Medical Risk Management<br>Schmitt, Jochen; TU Dresden Faculty of Medicine Carl Gustav Carus, Center for Evidence-based Healthcare<br>Eberlein-Gonska, Maria; University Hospital Carl Gustav Carus, Quality and Medical Risk Management<br>Kuhlen, Ralf; Initiative Qualitätsmedizin e.V.<br>Scriba, Peter; Initiative Qualitätsmedizin e.V.<br>Schoffer, Olaf; TU Dresden Faculty of Medicine Carl Gustav Carus, Center for Evidence-based Healthcare<br>Roessler, Martin; TU Dresden Faculty of Medicine Carl Gustav Carus, Center for Evidence-based Healthcare
**Primary Subject Heading**:	Health services research
Secondary Subject Heading:	Surgery
Keywords:	Quality in health care < HEALTH SERVICES ADMINISTRATION & MANAGEMENT, Colorectal surgery < SURGERY, Health & safety < HEALTH SERVICES ADMINISTRATION & MANAGEMENT

SCHOLARONE™ Manuscripts

I, the Submitting Author has the right to grant and does grant on behalf of all authors of the Work (as defined in the below author licence), an exclusive licence and/or a non-exclusive licence for contributions from authors who are: i) UK Crown employees; ii) where BMJ has agreed a CC-BY licence shall apply, and/or iii) in accordance with the terms applicable for US Federal Government officers or employees acting as part of their official duties; on a worldwide, perpetual, irrevocable, royalty-free basis to BMJ Publishing Group Ltd ("BMJ") its licensees and where the relevant Journal is co-owned by BMJ to the co-owners of the Journal, to publish the Work in this journal and any other BMJ products and to exploit all rights, as set out in our licence.

The Submitting Author accepts and understands that any supply made under these terms is made by BMJ to the Submitting Author unless you are acting as an employee on behalf of your employer or a postgraduate student of an affiliated institution which is paying any applicable article publishing charge ("APC") for Open Access articles. Where the Submitting Author wishes to make the Work available on an Open Access basis (and intends to pay the relevant APC), the terms of reuse of such Open Access shall be governed by a Creative Commons licence – details of these licences and which Creative Commons licence will apply to this Work are set out in our licence referred to above.

Other than as permitted in any relevant BMJ Author's Self Archiving Policies, I confirm this Work has not been accepted for publication elsewhere, is not being considered for publication elsewhere and does not duplicate material already published. I confirm all authors consent to publication of this Work and authorise the granting of this licence.

For peer review only

Long title: **Healthcare-related risk factors are more relevant for patient safety than hospital-related factors with regard to colorectal resections - cross-sectional evidence from a nationwide sample of 232 German hospitals**

Abbreviated title/ Short running head: **Patient safety in colorectal resections**

Felix Walther[1,2*], Jochen Schmitt[1], Maria Eberlein-Gonska[2], Ralf Kuhlen[3], Peter C. Scriba[3], Olaf Schoffer[1], Martin Roessler[1]

1 Center for Evidence-based Healthcare, Medical Faculty Carl Gustav Carus, TU Dresden, Dresden, Germany

2 Quality and Medical Risk Management, University Hospital Carl Gustav Carus, Dresden, Germany

3 Initiative Qualitätsmedizin e.V., Berlin, Germany

*** Corresponding author**

Felix.walther@ukdd.de

word (manuscript): 3022

Words (abstract): 300 / 300

For peer review only - http://bmjopen.bmj.com/site/about/guidelines.xhtml

Abstract

objectives: Studies analyzing colorectal resections usually focus on a specific outcome (e.g. mortality) and/ or specific risk factors at the individual (e.g. comorbidities) or hospital (e.g. volume) level. Comprehensive evidence across different patient safety outcomes, risk factors and patient groups is still scarce. Therefore the aim of this analysis was to investigate consistent relationships between multiple patient safety outcomes and healthcare and hospital risk factors in colorectal resection cases.

design: Cross-sectional study

setting: German inpatient routine care data of colorectal resections between 2016 to 2018

participants: We analyzed 54,168 colon resection and 20,395 rectum resection cases treated in German hospitals. The German Inpatient Quality Indicators were used to define colon resections and rectum resections transparently.

primary outcome measures: With in-hospital death, post-operative respiratory failure, renal failure and post-operative wound infections we included multiple patient safety outcomes as primary outcomes / dependent variables for our analysis. Healthcare (e.g. weekend surgery), hospital (e.g. volume) and case (e.g. age) characteristics served as independent covariates in a multilevel logistic regression model. The estimated regression coefficients were transferred into odds ratios (OR).

results: Weekend surgery, emergency admissions and transfers from other hospitals were significantly associated (ORs ranged from 1.1 to 2.6) with poor patient safety outcome (death, renal failure, post-operative respiratory failure) in colon resections and rectum resections. Hospital characteristics showed heterogeneous effects. In colon resections hospital volume was associated with insignificant or adverse associations (post-operative wound infections: OR 1.168 (95% CI 1.030 - 1.325)) to multiple patient safety outcomes. In rectum resections hospital volume was protectively associated to death, renal failure and post-operative respiratory failure (ORs ranged from 0.7 to 0.8).

conclusions: Transfer from other hospital and emergency admission are constantly associated with poor patient safety outcome. Hospital variables like volume, ownership or localization did not show consistent relationships to patient safety outcome.

.

Keywords: patient safety; health services research; Quality of Care; colon surgery; rectum surgery

For peer review only

For peer review only - http://bmjopen.bmj.com/site/about/guidelines.xhtml

Strengths and limitations of this study

- Large and current sample providing a broad span of cases, hospital types, ownerships, and locations.
- Comprehensive analysis previously solitary analyzed outcomes and risk factors
- Use of previously validated outcomes that were reported to occur most likely during hospitalization
- Accounting data lack information on patient history, medication, length of anesthesia, staff-to-patient ratios, surgeon volumes, centralization and which of the coded diagnoses had been present on admission.

For peer review only

For peer review only - http://bmjopen.bmj.com/site/about/guidelines.xhtml

Introduction

Measuring, assuring and improving patient safety are important objectives in colorectal resections. It is important for patient outcome, payment, and accreditation. One of the most frequently used outcome indicator in colorectal resections is in-hospital mortality.[1] However, it has been stressed that patient safety is reflected in both mortality and non-mortality outcomes.[2 3] A measurement of outcomes beyond mortality therefore is necessary for a comprehensive assessment of patient safety and care quality.[1] In addition to multiple relevant patient outcomes, various risk factors for adverse events were analyzed in previous studies. Besides patient characteristics (e.g. age, sex, comorbidities), especially the influence of healthcare (e.g. weekend surgery, emergency, transfer from other hospital) and hospital variables (e.g. volume, urbanization degree) were widely discussed. Weekend surgery,[4-6] emergency admission,[7 8] transfer from other hospitals[9-11], and case volume[12 13] were found to have significant effects on particularly mortality in colorectal resections. Analogous to patient outcomes, previous studies usually considered only subsets of these risk factors without analyzing them together.

A comprehensive analysis of patient safety and its covariates in colorectal resections should take multiple outcomes and multiple risk factors into account.[14] To our knowledge, such comprehensive analyses have rarely been reported. Based on that assumption, our analysis aimed to investigate whether healthcare and hospital characteristics are overwhelmingly associated with multiple patient safety outcomes in colorectal resections. Using a 3-year sample (2016-2018) of German inpatient accounting data we investigated relationships between case, healthcare, and hospital characteristics and the patient safety outcomes in-hospital death, post-operative respiratory failure, renal failure and post-operative wound infections in colorectal resections.

Materials and Methods

This explorative cross-sectional analysis was embedded into the IMPRESS study. The IMPRESS study was a cluster-randomized trial evaluating the effects of clinical peer review on mortality in patients ventilated >24h nested in a prospective cohort study of 232 participating hospitals. Details, baseline and first explorative results of the IMPRESS study were published previously.[15-18] The study has been registered at ISCRTN (ISRCTN10188560).[19] The identification of possible covariates of mortality and non-mortality outcomes in colorectal resections was a secondary aim of the IMPRESS study.

For peer review only

For peer review only - http://bmjopen.bmj.com/site/about/guidelines.xhtml

Data sources

The data used in this study were derived from two routine data sets. We used accounting data according to German law regulating stationary accounting (§21 Krankenhausentgeltgesetz) to gather information concerning age, sex, reason of admission, discharge destination, diagnoses/comorbidities (ICD-10-GM) and medical/surgical procedures (OPS codes). We applied the predefined groups of the Elixhauser comorbidity index and its coding modifications for ICD-10 (supplement S1) to adjust for relevant comorbidities. The Elixhauser Comorbidity Index is a score used to adjust for chronic or non-acute comorbidities in routine data sets.[20 21] To assess hospital characteristics (ownership, university hospital status, urbanization) we used data from the German hospital register („Deutsches Krankenhausverzeichnis“).

Study participation, privacy, and ethics

All participating hospitals submitted a written consent on participation prior to the start of the IMPRESS study. The data trust site at Koordinierungszentrum für Klinische Studien (KKS) Dresden ensured the anonymization of the data. The Center for Evidence-Based Healthcare (ZEGV) Dresden analyzed the anonymized data. The ethics committee of the TU Dresden approved the study protocol. The study protocol has been officially registered at the institutional review board (IRB): Office for Human Research Protections (OHRP); identification numbers: IRB00001473 and IORG0001076; date of approval: 24/04/2017

Patient and Public Involvement

This cross-sectional analysis uses observational routine data based on predefined outcomes and covariates without intervention and did not involve patients or the public in the design, or conduct, or reporting, or dissemination plans of our research.

Population

232 hospitals participated in the IMPRESS study (Figure 1). We included all cases with a colon and/ or rectum resection in the participating hospitals in 2016-2018. Due to anonymization, the data do not contain a patient-relation. Therefore patients admitted more than once entered the analysis as multiple hospital cases. For each hospital case, all of the documented information in terms of diagnoses and medical/ surgical procedures during hospitalization was available. We used the definitions of the German Inpatient Quality Indicators (GIQI) to define and distinguish partial colon resections (supplement S2), total colon resections (supplement S3) and rectum resections (supplement S4).[22] Hospitals without colon or rectum resections were excluded.

For peer review only - http://bmjopen.bmj.com/site/about/guidelines.xhtml

Outcomes and covariates

Following evidence from a previous study, in-hospital death, respiratory failure, renal failure and wound-infection can be validly operationalized in hospital discharge data.[23] Hence, we analyzed these outcomes in accordance with previously tested case definitions as presented in supplement S5. The outcomes death, respiratory failure, renal failure, and wound infections served as dependent variables.

The independent variables were classified into three groups:

1) case (age, sex, Elixhauser comorbidities)[20 21],
2) healthcare (admission date, surgical procedures/OPS codes, reason for admission, discharge destination) and
3) hospital (ownership, university hospital status, urbanization degree rural/urban).

This study focused on healthcare and hospital variables. Patient variables were primarily used for adjustment. To adjust for all potentially relevant risk factors available in the data, the estimations included the full set of independent case, healthcare, and hospital variables. At the case level, this included age, sex and Elixhauser comorbidities.[21] At the healthcare level, admission reason (referral/ emergency case/ transfer from other hospital), weekend surgery (identifiable via time stamp of the procedure), and total resection of the colon and resections of the colon and rectum were used. At the hospital level, hospital's case volume, the urbanization of the hospital (rural/ urban), university hospital status, and ownership (public/ non-profit/ private) were analyzed. Case volume entered the regression models in logarithmic form. This transformation of hospital volume data captures that volume-outcome relationships may be more pronounced at low case volumes.[24]

Statistical methods

We described case, healthcare, and hospital characteristics using absolute and relative frequencies in case of categorical variables. For continuous variables, we reported median and 1st and 3rd quartile. Relationships between patient outcomes and case-level, healthcare-level, and hospital-level risk factors were estimated using multilevel logistic regression models. These models contained a random intercept at the hospital level to capture correlation of patient outcomes within hospitals.[25] Estimations were conducted for cases with colon and rectum resection separately in bivariate and multivariate analyses. To foster interpretability of estimated effect sizes, we transformed the estimated regression coefficients into odds ratios. An estimated effect was considered as statistically significant if its p-value was below 5%. Statistical analysis was performed using Stata 15.1.

For peer review only - http://bmjopen.bmj.com/site/about/guidelines.xhtml

Sensitivity analysis

In the preliminary research of this thesis it became apparent that the literature distinguishes between colorectal cancer surgery and general colorectal resections.[1,12,13,26,27] Therefore, with respect to possible effect modifications, we explored differences between cases with and without a cancer diagnosis. The same applies to reported interactions between admission reason and the status of university hospitals compared to non-university hospitals.[16] Therefore we also reviewed these interactions to prevent possible effect modifications.

Results

Overall, 71,060 cases with colon and/ or rectum resection were included in the analysis. Separating colon resections and rectum resections a total of 54,168 colon resection cases were treated in 209 hospitals. 20,395 cases of rectum resections were treated in 200 hospitals. If both colon and rectum resection were documented (3,503 cases), the case was analyzed for both groups.

>>>Figure 1 – Flowchart of hospitals included for analysis<<<

The minority of included cases received combined colorectal resections (partial and total colon, colon and rectum). Emergency case admission or transfer from other hospitals were less frequent than referral. Compared to rectum resections, colon resections were more often surgically treated on weekends (8.6% vs. 3.8%), admitted as an emergency case (29.7% vs. 18.3%) or transferred from other hospitals (3.5% vs. 1.9%). The same applies to the rate of poor patient safety outcomes. Colon resection cases revealed higher rates of in-hospital death (9.6% vs. 4.2%), post-operative respiratory failure (16.7% vs. 12.2%), renal failure (15.2% vs. 10.3%) and post-operative wound infections (11.3% vs. 11.2%), than rectum resection cases.
The majority of the analyzed hospitals were localized in urban regions (59%). Most were in private (40%) or public (40%) ownership. The annual median hospital case volume was 72 (Q1 =38; Q3=116) for colon and 26 (Q1=11; Q3=42) for rectum resections.
The median age ranged from 67-68 years (Table 1). Male and female sex in colon and rectum resections were approximately equally represented. For Elixhauser comorbidities, the most frequent codes were solid tumor without metastasis (colon: 47.3%, rectum: 67.0%) uncomplicated hypertension (Colon/ rectum: 47%) and fluid and electrolyte disorders (colon: 45%, rectum: in both procedure groups. Descriptive results for all Elixhauser comorbidities are presented in supplement S6.

For peer review only

For peer review only - http://bmjopen.bmj.com/site/about/guidelines.xhtml

191 **Table 1 – Case and hospital characteristics of colon and rectum resections**

	COLON RESECTION		RECTUM RESECTION	
	n	% / Q1; Q3	n	% / Q1; Q3
number of cases	54,168	(100.0%)	20,395	(100.0%)
**PATIENT SAFETY OUTCOMES**				
in-hospital death				
no	48,914	(90.31 %)	19,525	(95.73 %)
yes	5,254	(9.68 %)	870	(4.26 %)
post-operative respiratory failure				
no	45,074	(83.21 %)	17,901	(87.77 %)
yes	9,094	(16.78 %)	2,494	(12.22 %)
renal failure				
no	45,920	(84.77 %)	18,279	(89.62 %)
yes	8,248	(15.22 %)	2,116	(10.37 %)
post-operative wound infections				
no	48,013	(88.63 %)	18,109	(88.79 %)
yes	6,155	(11.36 %)	2,286	(11.20 %)
**HEALTHCARE CHARACTERISTICS**				
colon resection				
total	2,662	(4.91 %)	-	-
partial	51,310	(94.72 %)	-	-
both	196	(0.36 %)	-	-
rectum resection				
no	50,665	(93.53 %)	-	-
yes	3,503	(6.46 %)	20,395	(100.00 %)
colon and rectum resection				
no	50,665	(93.53 %)	16,892	(82.82 %)
yes	3,503	(6.46 %)	3,503	(17.17 %)
weekend surgery				
no	49,473	(91.33 %)	19,603	(96.11 %)
yes	4,695	(8.66 %)	792	(3.88 %)
admission reason				
referral	36,129	(66.69 %)	16,249	(79.67 %)
emergency case	16,116	(29.75 %)	3,744	(18.35 %)
transfer from other hospital	1,923	(3.55 %)	402	(1.97 %)
**HOSPITAL CHARACTERISTICS**				
hospitals included	209	(100.00%)	200	(100.00%)
annual volume				
colon resection cases (median)	72	(38; 119)	-	-
total colon resection (median)	1	(0;3)	-	-
rectum resections (median)	-	-	26	(11;42)
urbanization				
urban	124	(59.33 %)	119	(59.50 %)
rural	85	(40.66 %)	81	(40.50 %)

For peer review only - http://bmjopen.bmj.com/site/about/guidelines.xhtml

ownership				
public	82	(39.23 %)	80	(40.00 %)
non-profit	41	(19.61 %)	39	(19.50 %)
private	86	(41.14 %)	81	(40.50 %)
university hospital				
no	201	(96.17 %)	192	(96.00 %)
yes	8	(3.82 %)	8	(4.00 %)
**CASE CHARACTERISTICS**				
Age				
median	68	(56; 77)	67	(57; 77)
sex				
male	26,954	(49.76 %)	10,367	(50.83 %)
female	27,214	(50.23 %)	10,028	(49.16 %)
Elixhauser comorbidities […][a]				

Legend: Q1: 1st Quartile Q3: 3rd quartile

[a] results of elixhauser comorbidities (e.g. alcohol abuse, blood loss anemia, cardiac arrhythmias, …) are presented in supplement S6

For peer review only

For peer review only - http://bmjopen.bmj.com/site/about/guidelines.xhtml

The bivariate analysis provided in supplements S7-S8 was performed to identify unadjusted effects of single covariates on the outcomes. The following multivariable analysis, focusing on healthcare and hospital level (Tables 2-3), was performed to achieve adjusted and robust effects.

Healthcare covariates

Admission as an emergency case or transfer from other hospital were associated to multiple poor patient safety outcomes in both groups. For example higher odds of in-hospital death were related to emergency admission in colon (OR 1.84 (95% CI, 1.69-2.01) and rectum resections (OR 2.02 (95% CI, 1.67-2.45) than referral hospital admissions. The same applies transfer from other hospital. The odds of in-hospital death were higher in colon (OR 2.52 (95% CI, 2.19-2.91)) and rectum resections (OR 2.67 (95% CI, 1.87-3.82)). Except of post-operative wound infections, weekend surgery was associated with worsening patient safety outcome in both groups.

Hospital covariates

While most of the healthcare-level covariates showed similar associations in both groups, hospital covariates showed insignificant or heterogeneous effects.

A higher annual case volume of colon resections indicated a higher risk of post-operative wound infections (OR 1.16 (95% CI, 1.03-1.32)). The remaining associations between annual case volume of colon resections and patient safety outcomes remained insignificant. A higher annual volume of rectum resections was associated with lower risks of in-hospital death (OR 0.70 (95% CI, 0.61-0.80)), post-operative respiratory failure (OR 0.84 (95% CI, 0.72-0.98)) and renal failure (OR 0.85 (95% CI, 0.76-0.95)).

Rural localization showed lower odds of renal failure (OR 0.77 (95% CI, 0.63-0.93)) in patients with colon resections solely.

Treatment in university hospitals was associated with increased odds of post-operative wound infections in colon (OR 1.98 (95% CI, 1.17-3.35)) and rectum resections (OR 2.29 (95% CI, 1.35-3.86)) compared to treatment in non-university hospitals.

The hospital ownership revealed differences between both groups and patient safety outcomes.

Non-profit (OR, 0.74 (95% CI, 0.55-0.99)) or private (OR 0.77, (95% CI, 0.60-0.99)) ownership was associated with lower risks of post-operative wound infections in colon resections. In contrast, odds of in-hospital death (OR 1.24, (95% CI, 1.02-1.50)) and renal failure (OR 1.93, (95% CI, 1.56-2.40)) in colon resections were higher in private hospitals. Rectum resections did not show significant associations of ownership and patient safety outcomes except for higher odds of renal failure (OR 1.59, (95% CI, 1.25-2.03)) in private hospitals.

For peer review only - http://bmjopen.bmj.com/site/about/guidelines.xhtml

Case covariates

Female sex was consistently associated with better outcomes in both groups, except of a borderline-insignificant association with in-hospital death (OR 0.93, (95% CI, 0.87-1.00)) in colon resections. Age was associated with higher risks of in-hospital death, post-operative respiratory failure and renal failure in both groups. Regarding post-operative wound infections, age was a borderline-insignificant protective factor in colon (OR 0.99, (95% CI, 0.99-1.00)) and rectum (OR 0.99, (95% CI, 0.99-1.00)) resections. Of all Elixhauser comorbidities analysed, coagulopathies showed highest ORs for poor patient safety outcomes associated including higher ORs of death in colon (OR 4.17 (95% CI 3.864 - 4.509)) or rectum (OR 4.30 (95% CI 3.600 - 5.158)) resections for example. The same applies to other patient safety outcomes like post-operative respiratory failure in colon (OR 3.117 (95% CI 2.920 - 3.327)) or rectum (OR 3.052 (95% CI 2.697 - 3.455)) resections, renal failure in colon (OR 3.332 (95% CI 3.118 - 3.561)) and rectum (OR 2.886 (95% CI 2.541 - 3.277)) resections and post-operative wound infections in colon (OR 1.644 (95% CI 1.531 - 1.764)) or rectum (OR 1.770 (95% CI 1.570 - 1.996)) resections either. Along with coagulopathies, fluid and electrolyte disorders, peripheral vascular disorders, congestive heart failure, chronic pulmonary disease, cardiac arrhythmias and pulmonary circulation disorders were also associated with multiple poor patient outcomes in both procedure groups. The multivariate results for the remaining Elixhauser groups can be found in supplements S9-S10.

We reviewed differences in results of stratified analyses for cases with and without a cancer of colon and rectum resections. Significant effect reversals were not given (supplements S11-S14). The review also did not reveal differences between university and non-university hospitals in terms of emergency admission or transfer from other hospital (supplements S15-S16). Therefore, a stratification between cases with and without cancer or university and non-university hospitals has not been applied.

For peer review only - http://bmjopen.bmj.com/site/about/guidelines.xhtml

Table 2 – Multivariate analysis of patient safety outcomes in 54,168 colon resections in 209 hospitals

	IN-HOSPITAL DEATH		POST-OPERATIVE RESPIRATORY FAILURE		RENAL FAILURE		POST-OPERATIVE WOUND INFECTION	
	OR	95% CI	OR	95% CI	OR	95% CI	OR	95% CI
**HEALTHCARE COVARIATES**								
admission reason								
referral	Ref.		Ref.		Ref.		Ref.	
emergency case	1.847***	(1.692 - 2.015)	1.413***	(1.320 - 1.513)	1.453***	(1.349 - 1.566)	1.145***	(1.067 - 1.228)
transfer from other hospital	2.528***	(2.193 - 2.915)	1.982***	(1.749 - 2.245)	1.908***	(1.678 - 2.171)	1.223**	(1.071 - 1.397)
weekend surgery								
no	Ref.		Ref.		Ref.		Ref.	
yes	1.669***	(1.515 - 1.839)	1.426***	(1.312 - 1.550)	1.480***	(1.360 - 1.610)	1.080	(0.984 - 1.186)
total colon resection								
no	Ref.		Ref.		Ref.		Ref.	
yes	2.679***	(2.369 - 3.029)	1.639***	(1.472 - 1.825)	2.228***	(1.999 - 2.483)	1.022	(0.913 - 1.143)
colon and rectum resection								
no	Ref.		Ref.		Ref.		Ref.	
yes	1.103	(0.960 - 1.267)	1.524***	(1.378 - 1.686)	1.408***	(1.265 - 1.567)	1.579***	(1.426 - 1.748)
**HOSPITAL COVARIATES**								
case volume	0.968	(0.871 - 1.076)	0.919	(0.807 - 1.047)	0.992	(0.891 - 1.106)	1.168*	(1.030 - 1.325)
area								
urban	Ref.		Ref.		Ref.		Ref.	
rural	1.061	(0.893 - 1.261)	0.863	(0.648 - 1.149)	0.772**	(0.635 - 0.939)	1.032	(0.824 - 1.292)
university hospital								
no	Ref.		Ref.		Ref.		Ref.	
yes	1.303	(0.888 - 1.912)	0.687	(0.338 - 1.397)	1.412	(0.889 - 2.241)	1.981*	(1.171 - 3.352)
ownership								
public	Ref.		Ref.		Ref.		Ref.	
non-profit	1.012	(0.811 - 1.262)	1.057	(0.726 - 1.540)	0.867	(0.670 - 1.122)	0.744*	(0.555 - 0.998)
private	1.244*	(1.026 - 1.507)	1.329	(0.972 - 1.817)	1.937***	(1.563 - 2.400)	0.777*	(0.608 - 0.992)
**CASE COVARIATES**								
sex								
male	Ref.		Ref		Ref		Ref	
female	0.937	(0.873 - 1.006)	0.788***	(0.745 - 0.833)	0.683***	(0.645 - 0.725)	0.882***	(0.832 - 0.936)
age	1.050***	(1.046 - 1.053)	1.014***	(1.012 - 1.017)	1.024***	(1.021 - 1.026)	0.998	(0.996 - 1.000)
Elixhauser comorbidities […][a]								

Legend:
*** p<0.001
** p <0.01
* p<0.05
[a] results of elixhauser comorbidities (e.g. alcohol abuse, blood loss anemia, cardiac arrhythmias, chronic pulmonary disease …) are presented in supplement S9

For peer review only - http://bmjopen.bmj.com/site/about/guidelines.xhtml

Table 3 – Multivariate analysis of patient safety outcomes in 20,395 rectum resections in 200 hospitals

	IN-HOSPITAL DEATH		POST-OPERATIVE RESPIRATORY FAILURE		RENAL FAILURE		POST-OPERATIVE WOUND INFECTION	
	OR	95% CI	OR	95% CI	OR	95% CI	OR	95% CI
**HEALTHCARE COVARIATES**								
admission reason								
referral	Ref.		Ref.		Ref.		Ref.	
emergency case	2.028***	(1.675 - 2.454)	1.335***	(1.170 - 1.523)	1.342***	(1.169 - 1.540)	1.291***	(1.138 - 1.466)
transfer from other hospital	2.679***	(1.874 - 3.828)	1.859***	(1.406 - 2.459)	1.927***	(1.461 - 2.541)	1.484**	(1.131 - 1.948)
weekend surgery								
no	Ref.		Ref.		Ref.		Ref.	
yes	1.960***	(1.483 - 2.591)	1.427**	(1.150 - 1.770)	1.391**	(1.127 - 1.717)	0.985	(0.784 - 1.238)
total colon resection								
no	Ref.		Ref.		Ref.		Ref.	
yes	2.579***	(2.163 - 3.074)	2.164***	(1.921 - 2.438)	1.859***	(1.645 - 2.100)	1.522***	(1.356 - 1.708)
**HOSPITAL COVARIATES**								
case volume	0.703***	(0.611 - 0.809)	0.844*	(0.725 - 0.982)	0.853**	(0.760 - 0.958)	0.973	(0.854 - 1.109)
area								
urban	Ref.		Ref.		Ref.		Ref.	
rural	1.072	(0.817 - 1.407)	0.904	(0.639 - 1.281)	0.834	(0.670 - 1.037)	0.854	(0.663 - 1.101)
university hospital								
no	Ref.		Ref.		Ref.		Ref.	
yes	1.616	(0.979 - 2.665)	0.853	(0.379 - 1.920)	1.299	(0.829 - 2.037)	2.292**	(1.358 - 3.869)
ownership								
public	Ref.		Ref.		Ref.		Ref.	
non-profit	0.851	(0.614 - 1.179)	1.059	(0.674 - 1.664)	0.762	(0.576 - 1.008)	0.761	(0.553 - 1.048)
private	0.925	(0.682 - 1.254)	1.267	(0.865 - 1.858)	1.597***	(1.256 - 2.030)	0.846	(0.642 - 1.117)
**CASE COVARIATES**								
sex								
male	Ref.		Ref		Ref		Ref	
female	0.842*	(0.710 - 0.998)	0.842**	(0.759 - 0.934)	0.685***	(0.613 - 0.765)	0.826***	(0.748 - 0.912)
age	1.068***	(1.059 - 1.078)	1.014***	(1.009 - 1.018)	1.021***	(1.016 - 1.026)	0.998	(0.995 - 1.002)
Elixhauser comorbidities […][a]								

Legend:
*** p<0.001
** p <0.01
* p<0.05
[a] results of elixhauser comorbidities (e.g. alcohol abuse, blood loss anemia, cardiac arrhythmias, chronic pulmonary disease …) are presented in supplement S10

For peer review

For peer review only - http://bmjopen.bmj.com/site/about/guidelines.xhtml

Discussion

This large cross-sectional analysis of 54,168 colon resections and 20,395 rectum resections presents new and comprehensive findings for patient safety.

Healthcare-level covariates were significant risk factors for multiple patient safety outcomes. Preoperative transfer from other hospitals and emergency admission as possible proxy for case urgency were crucial for poor patient safety outcome in both groups. These findings confirm recent literature reporting associations between emergency admissions or transfers from other hospitals and 30-day-mortality, 5-year survival, complications, length of stay or morbidities.[7-11] Weekend surgery was associated with higher risks for death, post-operative respiratory and renal failure in both groups supported by the literature of mortality in colon[4] and general surgery.[5 6] Regarding to rectum resections, the literature reported insignificant effects. These are most likely explained by a small number of included cases.[4] These findings underline the need for the consideration of healthcare contexts in risk-adjusted quality assurance.

The hospital covariates in this analysis showed conflicting effects. Inconclusive results were found for rural localization, university hospital status and hospital ownership. The estimated effects were insignificant (rural hospitals) or conflicting (volume, ownership) and therefore did not affect the considered patient safety outcomes overwhelmingly. The literature discusses the influence of case volume,[12 13 17 28 29] rural hospitals,[30-32] ownership[29 33], university hospital status[34] or hospital size in general[29] with confirming or contradicting results often explained by e.g. patient case-mix, staffing or surgeon experience differing between hospital sizes.[29 35] This may be due to outcome-relevant information not included in accounting data like staffing,[36 37] expertise[38] or certification[39]. For example a German study reported insignificant associations between ownership and post-operative wound infections after colon surgery.[40] The differences between our analyses are the procedure-definitions (partial/ total colon resections vs. open/ laparoscopic colon procedure), the sample size (54,168 colon resections vs. 28,291 colon procedures) and the data. The accounting data used in our analysis include individual information on age, sex and comorbidities. Infection surveillance data used by Schröder et al. does not include individual patient data on age, sex or severity of a patient's illness.[40]

Additionally, some studies did not stratify colon and rectum resections.[41 42] However, the heterogeneous results for both procedure groups indicates the relevance of stratification as already reported for other indications.[16]

With respect to case covariates age, sex and comorbidities like coagulopathies, heart diseases, lung diseases or fluid and electrolyte disorders were risk factors for poor patient outcome in both groups in this analysis and literature too.[26 43-48]

For peer review only - http://bmjopen.bmj.com/site/about/guidelines.xhtml

There are several strengths to this study. This study analyzed a large and current sample providing a broad span of cases, hospital types, ownerships, and locations. While previous studies emphasized specific covariates and/or outcomes we considered combined sets of previously solitarily analyzed outcomes and risk factors and, thus, provided a comprehensive analysis. The applied multilevel-regression is able to simultaneously analyze individual covariates like comorbidities and also covariates of hospital level like annual case volume. It also considers relationships between covariates (e.g. weekend surgery and emergency admissions).[25]

There are several limitations to this study. Secondary data induce challenges for a reliable operationalization of outcomes. First, the data are anonymized and the anonymization leads into the impossibility to validate the coded diagnoses.[49] Second, accounting data do not include information which of the coded diagnoses had been present on admission. To overcome these shortcomings, this study used a set of previously validated outcomes that were reported to occur most likely during hospitalization.[23] With respect to transfer from other hospital, recent literature distinguishes between urgent and non-urgent inter-hospital transfers.[9-11] The data included in this analysis do not include details of the reasons for transfer from other hospital. However, our results were adjusted for age, sex, comorbidities and weekend surgery representing severity and complexity. The different results depending on adjustment, stratification, bi- and multivariate analyses underline the need for careful and comprehensive statistical analysis. One weakness of German hospital discharge data is a lack of information on patient history, medication, length of anesthesia, staff-to-patient ratios, surgeon volumes, acuity/ reasons for interhospital transfers, validity of coding, centralization and comorbidities present on admission.[37 39 50] This lacking information may lead to bias as these covariates may influence the outcome and could not be considered in our study. To overcome these challenges we sought to consistently and referencably define colon and rectum resections,[22] outcomes[23] and comorbidities[20] for transparency. The advantage of this proceeding poses boundaries. These definitions do not involve specific distinctions referring to procedure (e.g. type, localization) or comorbidities (e.g. bowel disease). To keep transparency we decided to avoid own definitions of procedures or comorbidities. An additional limitation is the limited possibility to analyse detailed subgroups (e.g. case volume stratified by ownership, weekend surgeries stratified by admission) in models using a large set of covariates. It poses the risk of insignificance or separation due to smaller sample size of detailed subgroup-populations and outcomes.[51]

For peer review only - http://bmjopen.bmj.com/site/about/guidelines.xhtml

For peer review only

Conclusions

This study demonstrated that patient safety in colorectal resections is strongly particularly related to healthcare covariates. Our results implicate a need to account for admission reasons and weekend surgery when measuring and comparing patient safety. Therefore a risk adjustment for these covariates in quality assurance measures should follow. Hospital volume, ownership, urbanization degree and university hospital status could not be shown as overwhelming and conclusive covariates of patient safety in colorectal resections. Given these insights from an analysis of a large dataset, this paper contributes reliable and comprehensive evidence to the ongoing debate on hospital- and healthcare-related influence on patient safety in general.

For peer review only

For peer review only - http://bmjopen.bmj.com/site/about/guidelines.xhtml

Funding: The IMPRESS study is funded by the Innovation Fund of the Joint Federal Committee (Gemeinsamer Bundesausschuss, G-BA), Germany. Funding number: 01VSF16013. Open Access Funding by the Publication Fund of the TU Dresden.

Author contributions:

FW designed the concept, methods and investigation, visualized results and wrote the draft of the underlying analysis. MR undertook the formal analysis. MR and OS curated the data, supervised the methodology and project administration in general and revised the drafts. JS, MEG, PS and RK acquired funding, supervised the concept and investigation and revised the drafts.

Competing interests: Peter C. Scriba and Ralf Kuhlen are members of the scientific advisory board of IQM. Maria Eberlein-Gonska serves as an external expert for IQM. The other authors declare that they have no conflict of interest.

Data availability statement: The data are not publicly available due containing information that could compromise research participant privacy/consent. The consent given from the research participants included the obligation to analyze the data anonymously without disclosure to other parties.

For peer review only

References

1. O'Brien BS, McNally MP, Duncan JE. Controversies surrounding quality measurement in colon and rectal surgery. *Clin Colon Rectal Surg* 2014;27(1):26-31. doi: 10.1055/s-0034-1366916 [published Online First: 2014/03/04]

2. Goodacre S, Campbell M, Carter A. What do hospital mortality rates tell us about quality of care? *Emerg Med J* 2015;32(3):244-7. doi: 10.1136/emermed-2013-203022 [published Online First: 2013/09/26]

3. Lilford R, Pronovost P. Using hospital mortality rates to judge hospital performance: a bad idea that just won't go away. *Bmj* 2010;340:c2016. doi: 10.1136/bmj.c2016 [published Online First: 2010/04/22]

4. Huijts DD, van Groningen JT, Guicherit OR, et al. Weekend Effect in Emergency Colon and Rectal Cancer Surgery: A Prospective Study Using Data From the Dutch ColoRectal Audit. *J Natl Compr Canc Netw* 2018;16(6):735-41. doi: 10.6004/jnccn.2018.7016 [published Online First: 2018/06/13]

5. McCallum IJ, McLean RC, Dixon S, et al. Retrospective analysis of 30-day mortality for emergency general surgery admissions evaluating the weekend effect. *Br J Surg* 2016;103(11):1557-65. doi: 10.1002/bjs.10261 [published Online First: 2016/08/16]

6. O'Leary JD, Wunsch H, Leo AM, et al. Hospital admission on weekends for patients who have surgery and 30-day mortality in Ontario, Canada: A matched cohort study. *PLoS Med* 2019;16(1):e1002731. doi: 10.1371/journal.pmed.1002731 [published Online First: 2019/01/30]

7. Anderson JH, Hole D, McArdle CS. Elective versus emergency surgery for patients with colorectal cancer. *Br J Surg* 1992;79(7):706-9. doi: 10.1002/bjs.1800790739 [published Online First: 1992/07/01]

8. Mullen MG, Michaels AD, Mehaffey JH, et al. Risk Associated With Complications and Mortality After Urgent Surgery vs Elective and Emergency Surgery: Implications for Defining "Quality" and Reporting Outcomes for Urgent Surgery. *JAMA Surgery* 2017;152(8):768-74. doi: 10.1001/jamasurg.2017.0918

9. Chow CJ, Gaertner WB, Jensen CC, et al. Does Hospital Transfer Impact Outcomes After Colorectal Surgery? *Diseases of the Colon & Rectum* 2017;60(2):194-201. doi: 10.1097/dcr.0000000000000765

10. Sharp SP, Ata A, Valerian BT, et al. Complications and surgical outcomes after interhospital transfer vs direct admission in colorectal surgery: A National Surgical Quality Improvement Program analysis. *Am J Surg* 2017;213(6):1031-37. doi: 10.1016/j.amjsurg.2016.08.013 [published Online First: 2016/10/25]

For peer review only - http://bmjopen.bmj.com/site/about/guidelines.xhtml

11. Sharp SP, Schuster DJ, Ata A, et al. Impact of Interhospital Transfer on Outcomes in Non-emergency Colorectal Surgery. *World J Surg* 2018;42(5):1542-50. doi: 10.1007/s00268-017-4313-z [published Online First: 2017/10/29]

12. Chioreso C, Del Vecchio N, Schweizer ML, et al. Association Between Hospital and Surgeon Volume and Rectal Cancer Surgery Outcomes in Patients With Rectal Cancer Treated Since 2000: Systematic Literature Review and Meta-analysis. *Dis Colon Rectum* 2018;61(11):1320-32. doi: 10.1097/dcr.0000000000001198 [published Online First: 2018/10/05]

13. Huo YR, Phan K, Morris DL, et al. Systematic review and a meta-analysis of hospital and surgeon volume/outcome relationships in colorectal cancer surgery. *J Gastrointest Oncol* 2017;8(3):534-46. doi: 10.21037/jgo.2017.01.25 [published Online First: 2017/07/25]

14. Almoudaris AM, Burns EM, Bottle A, et al. Single measures of performance do not reflect overall institutional quality in colorectal cancer surgery. *Gut* 2013;62(3):423. doi: 10.1136/gutjnl-2011-301489

15. Schmitt J, Schoffer O, Walther F, et al. Effectiveness of the IQM peer review procedure to improve in-patient care—a pragmatic cluster randomized controlled trial (IMPRESS): study design and baseline results. *Journal of Public Health* 2019 doi: 10.1007/s10389-019-01118-9

16. Schoffer O, Roessler M, Walther F, et al. Patient-Level and Hospital-Level Risk Factors for In-Hospital Mortality in Patients Ventilated for More Than 24 Hours: Results of a Nationwide Cohort Study. *Journal of Intensive Care Medicine* 2020;0(0):0885066620942182. doi: 10.1177/0885066620942182

17. Roessler M, Walther F, Eberlein-Gonska M, et al. Exploring relationships between in-hospital mortality and hospital case volume using random forest: results of a cohort study based on a nationwide sample of German hospitals, 2016-2018. *BMC Health Serv Res* 2022;22(1):1. doi: 10.1186/s12913-021-07414-z [published Online First: 2022/01/04]

18. Schmitt J, Roessler M, Scriba P, et al. Effect of clinical peer review on mortality in patients ventilated for more than 24 hours: a cluster randomised controlled trial. *BMJ Qual Saf* 2022 doi: 10.1136/bmjqs-2021-013864 [published Online First: 2022/04/07]

19. Walther F, Schoffer O, Schmitt J. Effectiveness of a collegial consultation procedure to improve in-patient care—a pragmatic cluster randomized controlled trial (ISRCTN10188560): ISRCTN; 2018 [Available from: http://www.isrctn.com/ISRCTN10188560 accessed 01/09/ 2020.

For peer review only

20. Elixhauser A, Steiner C, Harris DR, et al. Comorbidity measures for use with administrative data. *Med Care* 1998;36(1):8-27. doi: 10.1097/00005650-199801000-00004 [published Online First: 1998/02/07]

21. Quan H, Sundararajan V, Halfon P, et al. Coding algorithms for defining comorbidities in ICD-9-CM and ICD-10 administrative data. *Med Care* 2005;43(11):1130-9. doi: 10.1097/01.mlr.0000182534.19832.83 [published Online First: 2005/10/15]

22. Mansky T, Nimptsch U, Cools A, et al. G-IQI – German Inpatient Quality Indicators. Version 5.1 – Band 1. Berlin: Universitätsverlag der TU Berlin 2017.

23. Maass C, Kuske S, Lessing C, et al. Are administrative data valid when measuring patient safety in hospitals? A comparison of data collection methods using a chart review and administrative data. *Int J Qual Health Care* 2015;27(4):305-13. doi: 10.1093/intqhc/mzv045 [published Online First: 2015/07/03]

24. Hentschker C, Mennicken R. The Volume-Outcome Relationship Revisited: Practice Indeed Makes Perfect. *Health Serv Res* 2018;53(1):15-34. doi: 10.1111/1475-6773.12696 [published Online First: 2017/09/05]

25. Snijders TAB, Bosker RJ. Multilevel analysis : an introduction to basic and advanced multilevel modeling. 2nd ed. ed: Los Angeles ; London : SAGE 2012.

26. Byrne BE, Mamidanna R, Vincent CA, et al. Population-based cohort study comparing 30- and 90-day institutional mortality rates after colorectal surgery. *Br J Surg* 2013;100(13):1810-7. doi: 10.1002/bjs.9318 [published Online First: 2013/11/15]

27. Sheikh L, Croft R, Harmston C. Counting the costs of complications in colorectal surgery. *N Z Med J* 2019;132(1497):32-36. [published Online First: 2019/06/21]

28. Link KH, Coy P, Roitman M, et al. Minimum Volume Discussion in the Treatment of Colon and Rectal Cancer: A Review of the Current Status and Relevance of Surgeon and Hospital Volume regarding Result Quality and the Impact on Health Economics. *Visceral Medicine* 2017;33(2):140-47. doi: 10.1159/000456044

29. Malheiro R, Peleteiro B, Correia S. Beyond the operating room: do hospital characteristics have an impact on surgical site infections after colorectal surgery? A systematic review. *Antimicrob Resist Infect Control* 2021;10(1):139. doi: 10.1186/s13756-021-01007-5 [published Online First: 2021/10/02]

For peer review only

For peer review only - http://bmjopen.bmj.com/site/about/guidelines.xhtml

30. Hamidi M, Hanna K, Omesiete P, et al. Does it matter where you get your surgery for colorectal cancer? *International Journal of Colorectal Disease* 2019;34(12):2121-27. doi: 10.1007/s00384-019-03436-6

31. Knight M. The Effect of Hospital Organizational Characteristics on Postoperative Complications. *Journal of Patient Safety* 2013;9(4):198-202. doi: 10.1097/PTS.0b013e3182995e5b

32. Pandit V, Jehan F, Zeeshan M, et al. Failure to Rescue in Postoperative Patients With Colon Cancer: Time to Rethink Where You Get Surgery. *Journal of Surgical Research* 2019;234:1-6. doi: https://doi.org/10.1016/j.jss.2018.08.046

33. Morris M, Iacopetta B, Platell C. Comparing survival outcomes for patients with colorectal cancer treated in public and private hospitals. *Med J Aust* 2007;186(6):296-300. [published Online First: 2007/03/21]

34. Juillard C, Lashoher A, Sewell CA, et al. A National Analysis of the Relationship Between Hospital Volume, Academic Center Status, and Surgical Outcomes for Abdominal Hysterectomy Done for Leiomyoma. *Journal of the American College of Surgeons* 2009;208(4):599-606. doi: https://doi.org/10.1016/j.jamcollsurg.2009.01.003

35. Tserenpuntsag B, Haley V, Van Antwerpen C, et al. Surgical Site Infection Risk Factors Identified for Patients Undergoing Colon Procedures, New York State 2009–2010. *Infection Control & Hospital Epidemiology* 2014;35(8):1006-12. doi: 10.1086/677156 [published Online First: 2016/05/10]

36. Etzioni DA, Young-Fadok TM, Cima RR, et al. Patient survival after surgical treatment of rectal cancer: impact of surgeon and hospital characteristics. *Cancer* 2014;120(16):2472-81. doi: 10.1002/cncr.28746 [published Online First: 2014/05/08]

37. Yasunaga H, Hashimoto H, Horiguchi H, et al. Variation in cancer surgical outcomes associated with physician and nurse staffing: a retrospective observational study using the Japanese Diagnosis Procedure Combination Database. *BMC Health Services Research* 2012;12(1):129. doi: 10.1186/1472-6963-12-129

38. Hall GM, Shanmugan S, Bleier JI, et al. Colorectal specialization and survival in colorectal cancer. *Colorectal Dis* 2016;18(2):O51-60. doi: 10.1111/codi.13246 [published Online First: 2015/12/29]

39. Trautmann F, Reißfelder C, Pecqueux M, et al. Evidence-based quality standards improve prognosis in colon cancer care. *European Journal of Surgical Oncology* 2018;44(9):1324-30. doi: https://doi.org/10.1016/j.ejso.2018.05.013

For peer review only - http://bmjopen.bmj.com/site/about/guidelines.xhtml

40. Schröder C, Behnke M, Geffers C, et al. Hospital ownership: a risk factor for nosocomial infection rates? *Journal of Hospital Infection* 2018;100(1):76-82. doi: https://doi.org/10.1016/j.jhin.2018.01.019

41. Kolfschoten NE, Marang-van de Mheen PJ, Wouters MWJM, et al. A Combined Measure of Procedural Volume and Outcome to Assess Hospital Quality of Colorectal Cancer Surgery, a Secondary Analysis of Clinical Audit Data. *PLOS ONE* 2014;9(2):e88737. doi: 10.1371/journal.pone.0088737

42. Liu CJ, Chou YJ, Teng CJ, et al. Association of surgeon volume and hospital volume with the outcome of patients receiving definitive surgery for colorectal cancer: A nationwide population-based study. *Cancer* 2015;121(16):2782-90. doi: 10.1002/cncr.29356 [published Online First: 2015/04/22]

43. Mamidanna R, Burns EM, Bottle A, et al. Reduced Risk of Medical Morbidity and Mortality in Patients Selected for Laparoscopic Colorectal Resection in England: A Population-Based Study. *Archives of Surgery* 2012;147(3):219-27. doi: 10.1001/archsurg.2011.311

44. Marietta M, Facchini L, Pedrazzi P, et al. Pathophysiology of Bleeding in Surgery. *Transplantation Proceedings* 2006;38(3):812-14. doi: https://doi.org/10.1016/j.transproceed.2006.01.047

45. Miller TE, Mythen M, Shaw AD, et al. Association between perioperative fluid management and patient outcomes: a multicentre retrospective study. *Br J Anaesth* 2021;126(3):720-29. doi: 10.1016/j.bja.2020.10.031 [published Online First: 2020/12/16]

46. Flynn DE, Mao D, Yerkovich ST, et al. The impact of comorbidities on post-operative complications following colorectal cancer surgery. *PLOS ONE* 2020;15(12):e0243995. doi: 10.1371/journal.pone.0243995

47. Inokuchi M, Kato K, Sugita H, et al. Impact of comorbidities on postoperative complications in patients undergoing laparoscopy-assisted gastrectomy for gastric cancer. *BMC Surgery* 2014;14(1):97. doi: 10.1186/1471-2482-14-97

48. Chang H-R, Shih S-C, Lin F-M. Impact of Comorbidities on the Outcomes of Older Patients Receiving Rectal Cancer Surgery. *International Journal of Gerontology* 2012;6(4):285-89. doi: https://doi.org/10.1016/j.ijge.2012.05.006

49. Ghadban T, Reeh M, Bockhorn M, et al. Decentralized colorectal cancer care in Germany over the last decade is associated with high in-hospital morbidity and mortality. *Cancer Manag Res* 2019;11:2101-07. doi: 10.2147/cmar.S197865 [published Online First: 2019/03/19]

50. Renzulli P, Lowy A, Maibach R, et al. The influence of the surgeon's and the hospital's caseload on survival and local recurrence after colorectal cancer surgery. *Surgery* 2006;139(3):296-304. doi: https://doi.org/10.1016/j.surg.2005.08.023

For peer review only - http://bmjopen.bmj.com/site/about/guidelines.xhtml

51. Mansournia MA, Geroldinger A, Greenland S, et al. Separation in Logistic Regression: Causes, Consequences, and Control. *American Journal of Epidemiology* 2017;187(4):864-70. doi: 10.1093/aje/kwx299

For peer review only

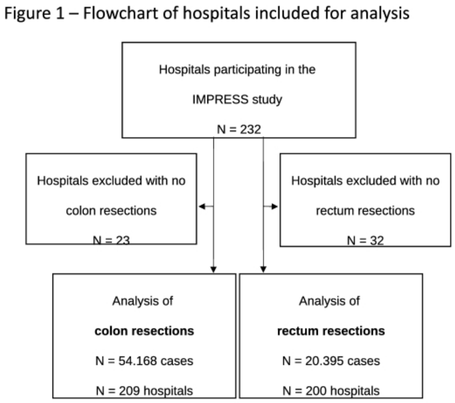

Figure 1 - Flowchart of hospitals included for analysis

338x190mm (600 x 600 DPI)

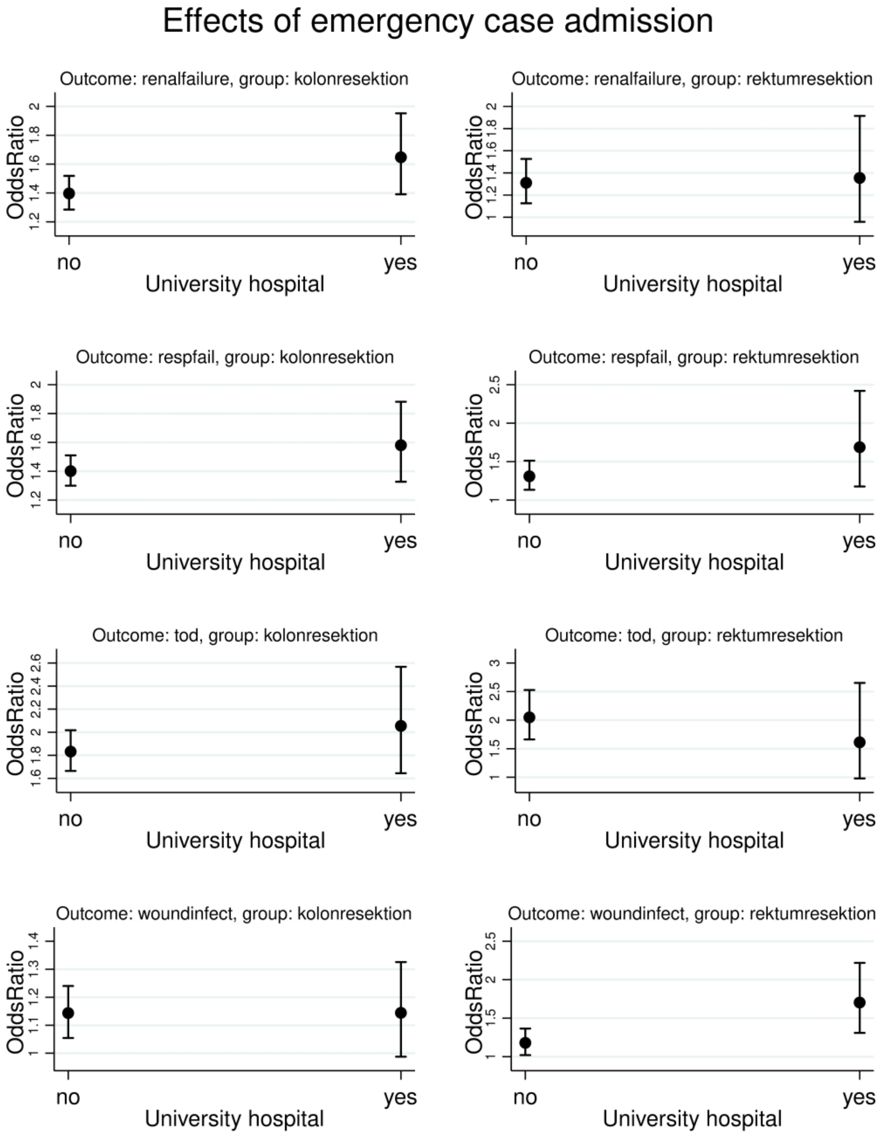

S15 - Figure: Interactions between emergency admission and university hospital status

406x508mm (600 x 600 DPI)

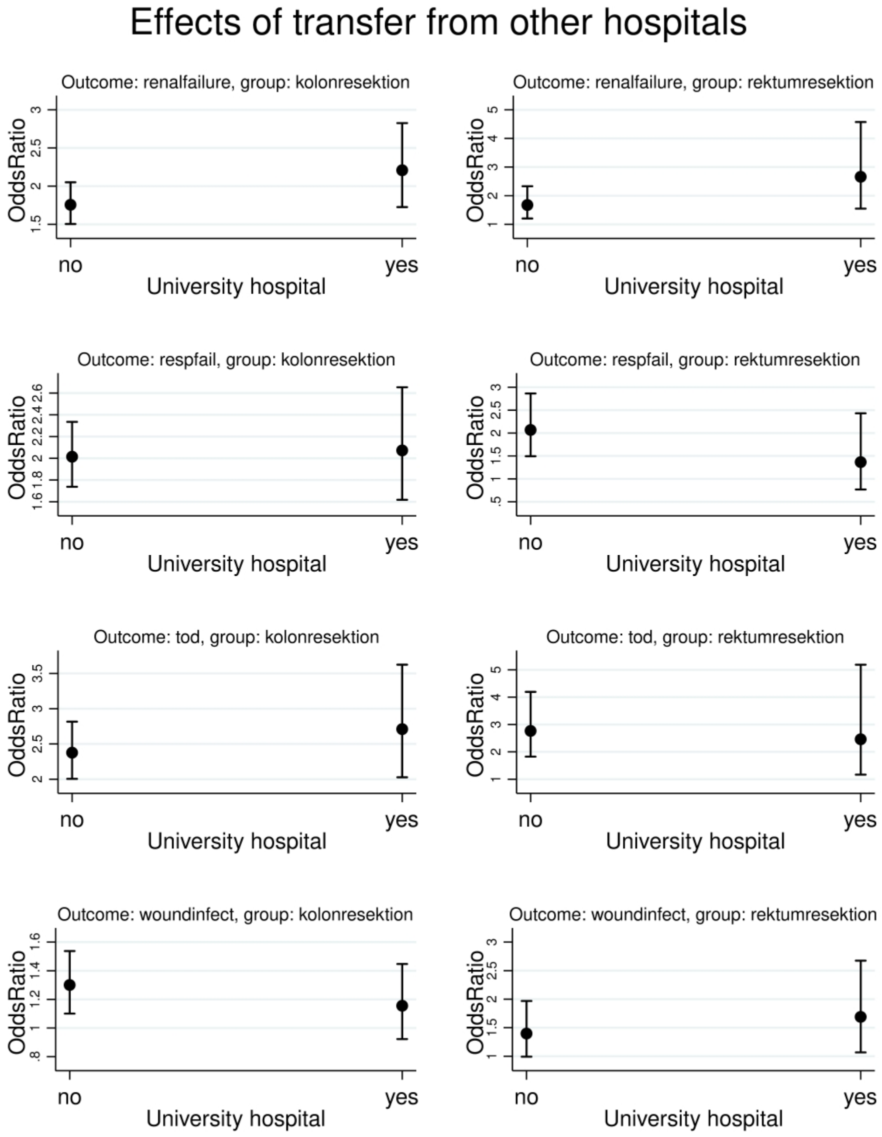

S16 - Figure: Interactions between transfer from other hospital admission and university hospital status

406x508mm (600 x 600 DPI)

S1: ICD-10 Codes of Elixhauser Comorbidity Groups[20]

Congestive heart failure: I099, I110, I130, I132, I255, I420, I425, I426, I427, I428, I429, I43, I50, P290

Cardiac arrhythmias: I441, I442, I443, I456, I459, I47, I48, I49, R000, R001, R008, T821, Z450, Z950

Valvular disease : A520, I05, I06, I07, I08, I091, I098, I34, I35, I36, I37, I38, I39, Q230, Q231, Q232, Q233, Z952, Z953 Z954

Pulmonary circulation Disorders: I26, I27, I280, I288, I289

Peripheral vascular disorders: I70, I71, I731, I738, I739, I771, I790, I792, K551, K558, K559, Z958, Z959

Hypertension, uncomplicated: I10

Hypertension, complicated: I11, I12, I13, I15

Paralysis: G041, G114, G801, G802, G81, G82, G830, G831, G832, G833, G834, G839

Other neurological disorders: G10, G11, G12, G13, G20, G21, G22, G254, G255, G312, G318, G319, G32, G35, G36, G37, G40, G41, G931, G934, R470, R56

Chronic pulmonary disease: I278, I279, J40, J41, J42, J43, J44, J45, J46, J47, J60, J61, J62, J63, J64, J65, J66, J67, J684, J701, J703

Diabetes, uncomplicated: E100, E101, E109, E110, E111, E119, E120, E121, E129, E130, E131, E139, E140, E141, E149

Diabetes, complicated: E102, E103, E104, E105, E106, E107, E108, E112, E113, E114, E115, E116, E117, E118, E122, E123, E124, E125, E126, E127, E128, E132, E133, E134, E135, E136, E137, E138, E142, E143, E144, E145, E146, E147, E148

Hypothyroidism: E00, E01, E02, E03, E890

Renal failure: I120, I131, N18, N19, N250, Z490, Z491, Z492, Z940, Z992

Peptic ulcer disease excluding bleeding: K257, K259, K267, K269, K277, K279, K287, K289

AIDS/HIV: B20, B21, B22, B24

Lymphoma: C81, C82, C83, C84, C85, C88, C96, C900, C902

Metastatic cancer: C77, C78, C79, C80

Solid tumor without metastasis: C00, C01, C02, C03, C04, C05, C06, C07, C08, C09, C1 0, C11, C12, C13, C14, C15, C16, C17, C18, C19, C20, C21, C22, C23, C24, C25, C26, C30, C31, C32, C33, C34, C37, C38, C39, C40, C41, C43, C45, C46, C47, C48, C49, C50, C51, C52, C53, C54, C55, C56, C57, C58, C60, C61, C62, C63, C64, C65, C66, C67, C68, C69, C70, C71, C72, C73, C74, C75, C76, C97

Rheumatoid arthritis/collagen vascular diseases: L940, L941, L943, M05, M06, M08, M120, M123, M30, M310, M311, M312, M313, M32, M33, M34, M35, M45, M461, M468, M469

Coagulopathy: D65, D66, D67, D68, D691, D693, D694, D695, D696

Obesity: E66

Weight loss: E40, E41, E42, E43, E44, E45, E46, R634, R64

Fluid and electrolyte disorders: E222, E86, E87

Blood loss anemia: D500

For peer review only

S2. OPS-codes for partial colon resections according to GIQI[21]

OPS-CODE	TRANSLATION (BY AUTHOR)
5-455.01	Partial resection of the colon: segmental resection: open surgery with anastomosis
5-455.02	Partial resection of the colon: segmental resection: open surgery with enterostoma and blind closure
5-455.03	Partial Resection of the Colon: Segment Resection: Open Surgery with Two Enterostomata
5-455.04	Partial Resection of the Colon: Segment Resection: Open Surgery with Anastomosis Anus praeter
5-455.05	Partial Resection of the Colon: Segmental Resection: Laparoscopic with Anastomosis
5-455.06	Partial Resection of the Colon: Segmental Resection: Laparoscopic with Enterostomy
5-455.07	Partial resection of the colon: segmental resection: change laparoscopic - open surgical
5-455.0X	Partial resection of the colon: Segment resection: Other
5-455.11	Partial resection of the colon: multiple segment resections: Open surgery with anastomosis
5-455.12	Partial resection of the colon: multiple segment resections: Open surgery with enterostoma and blind closure
5-455.13	Partial resection of the colon: multiple segment resections: Open surgery with two enterostomata
5-455.14	Partial resection of the colon: multiple segment resections: Open surgery with anastomosis anus praeter
5-455.15	Partial resection of the colon: multiple segment resections: Laparoscopic with anastomosis
5-455.16	Partial resection of the colon: multiple segment resections: Laparoscopic with enterostomy
5-455.17	Partial resection of the colon: multiple segment resections: Transfer laparoscopic - open surgery
5-455.1X	Partial resection of the colon: multiple segment resections: Other
5-455.21	Partial Resection of the Colon: Ileocecal Resection: Open Surgery with Anastomosis
5-455.22	Partial Resection of the Colon: Ileocecal Resection: Open surgery with enterostoma and blind closure
5-455.23	Partial Resection of the Colon: Ileocecal Resection: Open Surgery with Two Enterostomata
5-455.24	Partial Resection of the Colon: Ileocecal Resection: Open Surgery with Anastomosis Anus praeter
5-455.25	Partial Resection of the Colon: Ileocecal Resection: Laparoscopic with Anastomosis
5-455.26	Partial Resection of the Colon: Ileocecal Resection: Laparoscopic with Enterostoma
5-455.27	Partial resection of the colon: ileocecal resection: change laparoscopic - open surgical
5-455.2X	Partial resection of the colon: Ileocecal resection: Other
5-455.31	Partial resection of the colon: Caecal resection: Open surgery
5-455.35	Partial resection of the colon: Caecal resection: Laparoscopic
5-455.37	Partial resection of the colon: Caecal resection: change laparoscopic - open surgical
5-455.41	Partial resection of the colon: Resection of the ascending colon with coecum and right flexure [hemicolectomy right]: Open surgery with anastomosis
5-455.42	Partial Resection of the Colon: Resection of the ascending colon with coecum and right flexure [Hemicolectomy right]: Open surgery with enterostoma and
5-455.43	Partial Resection of the Colon: Resection of the ascending colon with coecum and right flexure [Hemicolectomy right]: Open surgery with two enterostomata
5-455.44	Partial resection of the colon: Resection of the ascending colon with coecum and right flexure [hemicolectomy right]: Open surgery with anastomosis anus praeter
5-455.45	Partial resection of the colon: Resection of the ascending colon with coecum and right flexure [hemicolectomy right]: Laparoscopic with anastomosis
5-455.46	Partial resection of the colon: Resection of the ascending colon with coecum and right flexure [hemicolectomy right]: Laparoscopically with enterostoma
5-455.47	Partial resection of the colon: resection of the ascending colon with coecum and right flexure [hemicolectomy right]: change laparoscopic - open surgical
5-455.4X	Partial resection of the colon: Resection of the ascending colon with coecum and right flexure [right hemicolectomy]: Other
5-455.51	Partial resection of the colon: Resection of the transverse colon: Open surgery with anastomosis
5-455.52	Partial resection of the colon: Resection of the transverse colon: open surgery with enterostoma and blind closure
5-455.53	Partial Resection of the Colon: Resection of the Transverse Colon: Open Surgery with Two Enterostomata
5-455.54	Partial resection of the colon: resection of the transverse colon: open surgery with anastomosis anus praeter
5-455.55	Partial resection of the colon: Resection of the transverse colon: Laparoscopic with anastomosis
5-455.56	Partial Resection of the Colon: Resection of the Transverse Colon: Laparoscopic with Enterostoma
5-455.57	Partial resection of the colon: resection of the transverse colon: change laparoscopic - open surgical
5-455.5X	Partial resection of the colon: Resection of the transverse colon: Other
5-455.61	Partial resection of the colon: resection of the descending colon with left flexure [hemicolectomy left]: open surgery with anastomosis
5-455.62	Partial resection of the colon: resection of the descending colon with left flexure [hemicolectomy left]: open surgery with enterostoma and blind closure
5-455.63	Partial resection of the colon: resection of the descending colon with left flexure [hemicolectomy left]: open surgery with two enterostomata
5-455.64	Partial resection of the colon: resection of the descending colon with left flexure [hemicolectomy left]: open surgery with anastomosis anus praeter
5-455.65	Partial resection of the colon: Resection of the descending colon with left flexure [Hemicolectomy left]: Laparoscopic with anastomosis
5-455.66	Partial Resection of the Colon: Resection of the descending colon with left flexure [Hemicolectomy left]: Laparoscopic with Enterostoma
5-455.67	Partial resection of the colon: resection of the descending colon with left flexure [hemicolectomy left]: change laparoscopic - open surgical
5-455.6X	Partial resection of the colon: Resection of the descending colon with left flexure [Hemicolectomy left]: Other
5-455.71	Partial resection of the colon: sigmoid resection: open surgery with anastomosis
5-455.72	Partial resection of the colon: sigmoid resection: open surgery with enterostoma and blind closure
5-455.73	Partial resection of the colon: sigmoid resection: open surgery with two enterostomata
5-455.74	Partial resection of the colon: sigmoid resection: open surgery with anastomosis anus praeter

5-455.75	Partial resection of the colon: sigmoid resection: laparoscopic with anastomosis
5-455.76	Partial resection of the colon: Sigmoid resection: laparoscopic with enterostoma
5-455.77	Partial resection of the colon: sigmoid resection: change laparoscopic - open surgical
5-455.7X	Partial resection of the colon: Sigmoid resection: Other
5-455.91	Partial resection of the colon: Resection of the ascending colon with coecum and right flexure and transverse colon [right hemicolectomy with transverse resection]:
5-455.92	Partial resection of the colon: Resection of the ascending colon with coecum and right flexure and transverse colon [right hemicolectomy with transverse resection]:
5-455.93	Partial resection of the colon: Resection of the ascending colon with coecum and right flexure and transverse colon [right hemicolectomy with transverse resection]:
5-455.94	Partial resection of the colon: Resection of the ascending colon with coecum and right flexure and transverse colon [right hemicolectomy with transverse resection]:
5-455.95	Partial resection of the colon: Resection of the ascending colon with coecum and right flexure and transverse colon [right hemicolectomy with transverse resection]:
5-455.96	Partial resection of the colon: Resection of the ascending colon with coecum and right flexure and transverse colon [right hemicolectomy with transverse resection]:
5-455.97	Partial resection of the colon: Resection of the ascending colon with coecum and right flexure and transverse colon [right hemicolectomy with transverse resection]:
5-455.9X	Partial resection of the colon: Resection of the ascending colon with coecum and right flexure and transverse colon [right hemicolectomy with transverse resection]:
5-455.A1	Partial resection of the colon: Resection of the descending colon with left flexure and transverse colon [left hemicolectomy with transverse resection]: Open
5-455.A2	Partial resection of the colon: Resection of the descending colon with left flexure and transverse colon [left hemicolectomy with transverse resection]: Open
5-455.A3	Partial resection of the colon: Resection of the descending colon with left flexure and transverse colon [left hemicolectomy with transverse resection]: Open
5-455.A4	Partial resection of the colon: Resection of the descending colon with left flexure and transverse colon [left hemicolectomy with transverse resection]: Open
5-455.A5	Partial Resection of the Colon: Resection of the descending colon with left flexure and transverse colon [Hemicolectomy left with transverse resection]: Laparoscopic
5-455.A6	Partial Resection of the Colon: Resection of the descending colon with left flexure and transverse colon [Hemicolectomy left with transverse resection]: Laparoscopic
5-455.A7	Partial resection of the colon: resection of the descending colon with left flexure and transverse colon [hemicolectomy left with transverse resection]: change
5-455.AX	Partial resection of the colon: Resection of the descending colon with left flexure and transverse colon [left hemicolectomy with transverse resection]: Other
5-455.B1	Partial resection of the colon: resection of the descending colon and sigmoid colon: open surgery with anastomosis
5-455.B2	Partial resection of the colon: resection of the descending colon and sigmoid colon: open surgery with enterostoma and blind closure
5-455.B3	Partial Resection of the Colon: Resection of the descending colon and sigmoid colon: Open surgery with two enterostomata
5-455.B4	Partial Resection of the Colon: Resection of the descending colon and sigmoid colon: Open surgery with anastomosis anus praeter
5-455.B5	Partial resection of the colon: resection of the descending colon and sigmoid colon: laparoscopic with anastomosis
5-455.B6	Partial resection of the colon: resection of the descending colon and sigmoid colon: laparoscopic with enterostoma
5-455.B7	Partial Resection of the Colon: Resection of the descending colon and sigmoid colon: transfer laparoscopically - open surgery
5-455.BX	Partial resection of the colon: Resection of the descending colon and sigmoid colon: Other
5-455.C1	Partial resection of the colon: resection of the ascending, transverse and descending colon with coecum and right and left flexure [hemicolectomy right and left
5-455.C2	Partial resection of the colon: resection of the ascending, transverse and descending colon with coecum and right and left flexure [hemicolectomy right and left
5-455.C3	Partial resection of the colon: resection of the ascending, transverse and descending colon with coecum and right and left flexure [hemicolectomy right and left
5-455.C4	Partial resection of the colon: resection of the ascending, transverse and descending colon with coecum and right and left flexure [hemicolectomy right and left
5-455.C5	Partial resection of the colon: resection of the ascending, transverse and descending colon with coecum and right and left flexure [hemicolectomy right and left
5-455.C6	Partial Resection of the Colon: resection of the ascending, transverse and descending colon with coecum and right and left flexure [hemicolectomy right and left
5-455.C7	Partial resection of the colon: resection of the ascending, transverse and descending colon with coecum and right and left flexure [hemicolectomy right and left
5-455.CX	Partial resection of the colon: Resection of the ascending, transverse and descending colon with coecum and right and left flexure [hemicolectomy right and left
5-455.D1	Partial resection of the colon: resection of the transverse colon, descending colon with left flexure and sigmoid colon [hemicolectomy left with
5-455.D2	Partial resection of the colon: resection of the transverse colon, descending colon with left flexure and sigmoid colon [hemicolectomy left with
5-455.D3	Partial resection of the colon: resection of the transverse colon, descending colon with left flexure and sigmoid colon [hemicolectomy left with
5-455.D4	Partial resection of the colon: resection of the transverse colon, descending colon with left flexure and sigmoid colon [hemicolectomy left with
5-455.D5	Partial resection of the colon: resection of the transverse colon, descending colon with left flexure and sigmoid colon [hemicolectomy left with
5-455.D6	Partial resection of the colon: resection of the transverse colon, descending colon with left flexure and sigmoid colon [hemicolectomy left with
5-455.D7	Partial resection of the colon: resection of the transverse colon, descending colon with left flexure and sigmoid colon [hemicolectomy left with
5-455.DX	Partial resection of the colon: resection of the transverse colon, descending colon with left flexure and sigmoid colon [left hemicolectomy with
5-455.X1	Partial resection of the colon: Other: Open surgery with anastomosis
5-455.X2	Partial resection of the colon: Other: Open surgery with enterostoma and blind closure
5-455.X3	Partial resection of the colon: Other: Open surgery with two enterostomata
5-455.X4	Partial resection of the colon: Other: Open surgery with anastomosis anus praeter
5-455.X5	Partial resection of the colon: Other: Laparoscopic with anastomosis
5-455.X6	Partial resection of the colon: Other: Laparoscopic with enterostoma
5-455.X7	Partial resection of the colon: Other: transfer laparoscopic - open surgical
5-455.XX	Partial resection of the colon: Other: Other
5-455.Y	Partial resection of the colon: N.n.ref.

S3: OPS-codes for total colon resections according to GIQI[21]

OPS-CODE	TRANSLATION (BY AUTHOR)
5-456.00	(Total) Colectomy and Proctocolectomy: Colectomy: Open surgery with ileostoma
5-456.01	(Total) Colectomy and Proctocolectomy: Colectomy: Open surgery with ileorectal anastomosis with reservoir (pouch)
5-456.02	(Total) Colectomy and Proctocolectomy: Colectomy: Open surgery with ileorectal anastomosis without reservoir (pouch)
5-456.03	(Total) Colectomy and Proctocolectomy: Colectomy: Open surgery with ileoanal anastomosis with reservoir (pouch)
5-456.04	(Total) Colectomy and Proctocolectomy: Colectomy: Open surgery with ileoanal anastomosis without reservoir (pouch)
5-456.05	(Total) Colectomy and Proctocolectomy: Colectomy: Laparoscopic with reservoir anastomosis (Pouch)
5-456.06	(Total) Colectomy and Proctocolectomy: Colectomy: Laparoscopic with Anastomosis without reservoir (Pouch)
5-456.07	(Total) Colectomy and Proctocolectomy: Colectomy: Laparoscopic with Ileostoma
5-456.08	(Total) Colectomy and Proctocolectomy: Colectomy: change laparoscopic - open surgical
5-456.0X	(Total) Colectomy and Proctocolectomy: Colectomy: Other
5-456.10	(Total) Colectomy and Proctocolectomy: Proctocolectomy: Open surgery with ileostoma
5-456.11	(Total) Colectomy and Proctocolectomy: Proctocolectomy: Open surgery with ileorectal anastomosis with reservoir (pouch)
5-456.12	(Total) Colectomy and Proctocolectomy: Proctocolectomy: Open surgery with ileorectal anastomosis without reservoir (pouch)
5-456.13	(Total) Colectomy and Proctocolectomy: Proctocolectomy: Open surgery with ileoanal anastomosis with reservoir (pouch)
5-456.14	(Total) Colectomy and Proctocolectomy: Proctocolectomy: Open surgery with ileoanal anastomosis without reservoir (pouch)
5-456.15	(Total) colectomy and proctocolectomy: Proctocolectomy: Laparoscopic with reservoir anastomosis (pouch)
5-456.16	(Total) Colectomy and Proctocolectomy: Proctocolectomy: Laparoscopic with anastomosis without reservoir (pouch)
5-456.17	(Total) colectomy and proctocolectomy: proctocolectomy: laparoscopic with ileostomy
5-456.18	(Total) colectomy and proctocolectomy: proctocolectomy: change laparoscopic - open surgical
5-456.1X	(Total) colectomy and proctocolectomy: Proctocolectomy: Other
5-456.20	(Total) colectomy and proctocolectomy: colectomy with proctomucosectomy: open surgery with ileostoma
5-456.21	(Total) colectomy and proctocolectomy: colectomy with proctomucosectomy: open surgery with ileorectal anastomosis with reservoir (pouch)
5-456.22	(Total) Colectomy and Proctocolectomy: Colectomy with Proctomucosectomy: Open surgery with ileorectal anastomosis without reservoir (pouch)
5-456.23	(Total) Colectomy and Proctocolectomy: Colectomy with proctomucosectomy: Open surgery with ileoanal anastomosis with reservoir (pouch)
5-456.24	(Total) colectomy and proctocolectomy: colectomy with proctomucosectomy: open surgery with ileoanal anastomosis without reservoir (pouch)
5-456.25	(Total) colectomy and proctocolectomy: colectomy with proctomucosectomy: laparoscopic with reservoir anastomosis (pouch)
5-456.26	(Total) colectomy and proctocolectomy: colectomy with proctomucosectomy: laparoscopic with anastomosis without reservoir (pouch)
5-456.27	(Total) colectomy and proctocolectomy: colectomy with proctomucosectomy: laparoscopic with ileostomy
5-456.28	(Total) colectomy and proctocolectomy: colectomy with proctomucosectomy: change laparoscopic - open surgical
5-456.2X	(total) colectomy and proctocolectomy: colectomy with proctomucosectomy: other
5-456.X0	(Total) colectomy and proctocolectomy: Other: Open surgery with ileostomy
5-456.X1	(Total) colectomy and proctocolectomy: Other: Open surgery with ileorectal anastomosis with reservoir (pouch)
5-456.X2	(total) colectomy and proctocolectomy: Other: Open surgery with ileorectal anastomosis without reservoir (pouch)
5-456.X3	(Total) colectomy and proctocolectomy: Other: Open surgery with ileoanal anastomosis with reservoir (pouch)
5-456.X4	(Total) colectomy and proctocolectomy: Other: Open surgery with ileoanal anastomosis without reservoir (pouch)
5-456.X5	(total) colectomy and proctocolectomy: Other: Laparoscopic with reservoir anastomosis (pouch)
5-456.X6	(Total) colectomy and proctocolectomy: Other: Laparoscopic with anastomosis without reservoir (pouch)
5-456.X7	(Total) colectomy and proctocolectomy: Other: Laparoscopic with ileostomy
5-456.X8	(total) colectomy and proctocolectomy: Other: change laparoscopic - open surgical
5-456.XX	(Total) colectomy and proctocolectomy: Other: Other
5-456.Y	(Total) colectomy and proctocolectomy: N.n.d.

For peer review only - http://bmjopen.bmj.com/site/about/guidelines.xhtml

S4: OPS-codes for rectum resections according to GIQI[21]

OPS-CODE	TRANSLATION (BY AUTHOR)
5-484.01	Rectum resection with sphincter preservation: Anterior cuff resection: Open surgery with anastomosis
5-484.02	Rectum resection with sphincter preservation: Anterior cuff resection: Open surgery with enterostoma and blind closure
5-484.05	Rectum resection with sphincter preservation: Anterior cuff resection: Laparoscopic with anastomosis
5-484.06	Rectum resection with sphincter preservation: Anterior cuff resection: Laparoscopic with enterostomy and blind closure
5-484.08	Rectum resection with sphincter preservation: Anterior cuff resection: change laparoscopic - open surgery with anastomosis
5-484.09	Rectum resection with sphincter preservation: Anterior cuff resection: change laparoscopic - open surgery with enterostoma and blind closure
5-484.0X	Rectum resection with sphincter preservation: Anterior cuff resection: Other
5-484.11	Rectum resection with sphincter preservation: Posterior rectotomy [Rectotomia posterior]: Open surgery with anastomosis
5-484.12	Rectum resection with sphincter preservation: Posterior rectotomy [Rectotomia posterior]: Open surgery with enterostoma and blind closure
5-484.15	Rectum resection with sphincter preservation: Posterior cuff resection [Rectotomia posterior]: Laparoscopic with anastomosis
5-484.16	Rectum resection with sphincter preservation: Posterior cuff resection [Rectotomia posterior]: Laparoscopic with enterostoma and blind closure
5-484.18	Rectum resection with sphincter preservation: posterior cuff resection [Rectotomia posterior]: change laparoscopic - open surgery with anastomosis
5-484.19	Rectum resection with sphincter preservation: posterior cuff resection [rectotomia posterior]: change laparoscopically - open surgically with enterostoma and
5-484.1X	Rectum resection with sphincter preservation: Posterior cuff resection [Rectotomia posterior]: Other
5-484.21	Rectum resection with preservation of sphincter: Tubular resection with paraproctica left intact: Open surgery with anastomosis
5-484.22	Rectum resection with preservation of sphincter: Tubular resection with paraproktium left in place: Open surgery with enterostoma and blind closure
5-484.25	Rectum resection with preservation of sphincter: Tubular resection with paraproctica left in place: Laparoscopic with anastomosis
5-484.26	Rectum resection with preservation of sphincter: Tubular resection with paraproktium: Laparoscopic with enterostoma and blind closure
5-484.27	Rectum resection with sphincter preservation: Tubular resection with paraprotic left in place: Peranal
5-484.28	Rectum resection with sphincter preservation: Tubular resection with paraproctium left in place: change laparoscopic - open surgery with anastomosis
5-484.29	Rectum resection with preservation of sphincter: Tubular resection with preservation of paraproktium: change laparoscopically - open surgically with enterostoma and
5-484.2X	Rectum resection with preservation of sphincter: Tubular resection with preservation of paraproktium: Other
5-484.31	Rectum resection with sphincter preservation: Anterior resection: Open surgery with anastomosis
5-484.32	Rectum resection with sphincter preservation: Anterior resection: open surgery with enterostoma and blind closure
5-484.35	Rectum resection with sphincter preservation: Anterior resection: Laparoscopic with anastomosis
5-484.36	Rectum resection with sphincter preservation: Anterior resection: Laparoscopic with enterostoma and blind closure
5-484.38	Rectum resection with sphincter preservation: Anterior resection: change laparoscopic - open surgical with anastomosis
5-484.39	Rectum resection with sphincter preservation: anterior resection: change laparoscopically - open surgically with enterostoma and blind closure
5-484.3X	Rectum resection with sphincter preservation: Anterior resection: Other
5-484.51	Rectum resection with sphincter preservation: deep anterior resection: open surgery with anastomosis
5-484.52	Rectum resection with sphincter preservation: deep anterior resection: open surgery with enterostoma and blind closure
5-484.55	Rectum resection with sphincter preservation: Deep anterior resection: Laparoscopic with anastomosis
5-484.56	Rectum resection with sphincter preservation: Deep anterior resection: Laparoscopic with enterostoma and blind closure
5-484.58	Rectum resection with sphincter preservation: deep anterior resection: change laparoscopic - open surgery with anastomosis
5-484.59	Rectum resection with sphincter preservation: deep anterior resection: change laparoscopic - open surgical with enterostoma and blind closure
5-484.5X	Rectum resection with sphincter preservation: Deep anterior resection: Other
5-484.61	Rectum resection with sphincter preservation: deep anterior resection with peranal anastomosis: open surgery with anastomosis
5-484.65	Rectum resection with sphincter preservation: deep anterior
5-484.68	Rectum resection with sphincter preservation: deep anterior resection with peranal anastomosis: change laparoscopic - open surgical with anastomosis
5-484.6X	Rectum resection with sphincter preservation: Deep anterior resection with peranal anastomosis: Other
5-484.X1	Rectum resection with sphincter preservation: Other: Open surgery with anastomosis
5-484.X2	Rectum resection with sphincter preservation: Other: Open surgery with enterostoma and blind closure
5-484.X5	Rectum resection with sphincter preservation: Other: Laparoscopic with anastomosis
5-484.X6	Rectum resection with sphincter preservation: Other: Laparoscopic with enterostoma and blind closure

5-484.X8	Rectum resection with sphincter preservation: Other: change laparoscopic - open surgery with anastomosis
5-484.X9	Rectum resection with sphincter preservation: Other: change laparoscopically - open surgically with enterostoma and blind closure
5-484.XX	Rectum resection with sphincter preservation: Other: Other
5-484.Y	Rectum resection with sphincter preservation: N.n.ref.
5-485.01	Rectum resection without preservation of sphincter: Abdominoperineal: Open surgery
5-485.02	Rectum resection without sphincter preservation: Abdominoperineal: Combined open surgical-laparoscopic
5-485.0X	Rectum resection without sphincter preservation: Abdominoperineal: Other
5-485.1	Rectum resection without preservation of sphincter: Abdominoperineal with removal of adjacent organs
5-485.21	Rectum resection without sphincter preservation: Sacroiliac abdomen: Open surgery
5-485.22	Rectum resection without sphincter preservation: Sacroiliac abdomen: Combined open surgical-laparoscopic
5-485.2X	Rectum resection without sphincter preservation: Sacroiliac abdomen: Other
5-485.3	Rectum resection without sphincter preservation: Sacral abdominosacral with removal of neighboring organs
5-485.4	Rectum resection without sphincter preservation: sacroperineal
5-485.5	Rectum resection without sphincter preservation: Perineal
5-485.X	Rectum resection without sphincter preservation: Other
5-485.Y	Rectum resection without sphincter preservation: N.n.ref.

For peer review only

S5: Definitions of patient safety outcomes

PATIENT SAFETY OUTCOME	DEFINITION ACCORDING TO ICD-10 GM
IN-HOSPITAL DEATH	Discharge destination: death (included in accounting data derived)
POST-OPERATIVE RESPIRATORY FAILURE[15]	ICD-10: J96 .0 - Respiratory failure, not elsewhere classified
POST-OPERATIVE WOUND INFECTION[15]	ICD-10: T81.4 - Infection following a procedure, not elsewhere classified<br>ICD-10: T82.6 - Infection and inflammatory reaction due to cardiac valve prosthesis<br>ICD-10: T82.7 - Infection and inflammatory reaction due to other cardiac and vascular devices, implants and grafts<br>ICD-10: T83.5 - Infection and inflammatory reaction due to prosthetic device, implant and graft in urinary system<br>ICD-10: T83.6 - Infection and inflammatory reaction due to prosthetic device, implant and graft in genital tract<br>ICD-10: T84.5 - Infection and inflammatory reaction due to internal joint prosthesis<br>ICD-10: T84.6 - Infection and inflammatory reaction due to internal fixation device [any site]<br>ICD-10: T84.7 - Infection and inflammatory reaction due to other internal orthopaedic prosthetic devices, implants and grafts<br>ICD-10: T87.4 - Infection of amputation stump
RENAL FAILURE[15]	ICD-10: N17.0 - Acute renal failure with tubular necrosis<br>ICD-10: N17.1 - Acute renal failure with acute cortical necrosis<br>ICD-10: N17.2 - Acute renal failure with medullary necrosis<br>ICD-10: N17.8 - Other acute renal failure<br>ICD-10: N17.9 - Acute renal failure, unspecified<br>ICD-10: N99.0- Postprocedural renal failure<br>Procedure code: 8-853.7- Continuous, venovenous, pump-driven (CVVH), anticoagulation with heparin or without anticoagulation

For peer review only

S6: Case-, care- and hospital related characteristics

	COLON RESECTIONS		RECTUM RESECTIONS	
**PATIENT SAFETY OUTCOMES**	n	% / Q1; Q3	n	% / Q1; Q3
Outcome in-hospital death				
no	48,914	(90.30 %)	19,525	(95.73 %)
yes	5,254	(9.69 %)	870	(4.26 %)
Outcome post-operative respiratory failure				
no	45,074	(83.21 %)	17,901	(87.77 %)
yes	9,094	(16.78 %)	2,494	(12.22 %)
Outcome post-operative wound infections				
no	48,013	(88.63 %)	18,109	(88.79 %)
yes	6,155	(11.36 %)	2,286	(11.2 %)
Outcome renal failure				
no	45,920	(84.77 %)	18,279	(89.62 %)
yes	8,248	(15.22 %)	2,116	(10.37 %)
**CASE CHARACTERISTICS**				
age (median)	68	(56; 77)	67	(57; 77)
elixhauser groups per case (median)	3	(2; 5)	3	(2; 5)
sex				
male	26,954	(49.76 %)	10,367	(50.83 %)
female	27,214	(50.23 %)	10,028	(49.16 %)
AIDS/HIV				
no	54,142	(99.95 %)	20,383	(99.94 %)
yes	26	(0.04 %)	12	(0.05 %)
alcohol abuse				
no	53,025	(97.88 %)	20,088	(98.49 %)
yes	1,143	(2.11 %)	307	(1.5 %)
blood loss anemia				
no	53,022	(97.88 %)	20,134	(98.72 %)
yes	1,146	(2.11 %)	261	(1.27 %)
cardiac arrhythmias				
no	43,241	(79.82 %)	17,135	(84.01 %)
yes	10,927	(20.17 %)	3,260	(15.98 %)
chronic pulmonary disease				
no	49,042	(90.53 %)	18,792	(92.14 %)
yes	5,126	(9.46 %)	1,603	(7.85 %)
coagulopathy				
no	44,935	(82.95 %)	17,598	(86.28 %)
yes	9,233	(17.04 %)	2,797	(13.71 %)
congestive heart failure				
no	47,447	(87.59 %)	18,608	(91.23 %)
yes	6,721	(12.4 %)	1,787	(8.76 %)
deficiency anemia				
no	51,887	(95.78 %)	19,852	(97.33 %)
yes	2,281	(4.21 %)	543	(2.66 %)
depression				
no	51,232	(94.57 %)	19,318	(94.71 %)
yes	2,936	(5.42 %)	1,077	(5.28 %)
diabetes, complicated				
no	51,887	(95.78 %)	19,754	(96.85 %)
yes	2,281	(4.21 %)	641	(3.14 %)
diabetes, uncomplicated				
no	46,491	(85.82 %)	17,664	(86.6 %)
yes	7,677	(14.17 %)	2,731	(13.39 %)
drug abuse				
no	53,911	(99.52 %)	20,321	(99.63 %)
yes	257	(0.47 %)	74	(0.36 %)
fluid and electrolyte disorders				
no	29,678	(54.78 %)	12,122	(59.43 %)
yes	24,490	(45.21 %)	8,273	(40.56 %)
hypertension, complicated				
no	51,632	(95.31 %)	19,680	(96.49 %)
yes	2,536	(4.68 %)	715	(3.51 %)
hypertension, uncomplicated				
no	28,369	(52.37 %)	10,692	(52.42 %)
yes	25,799	(47.62 %)	9,703	(47.57 %)

For peer review only - http://bmjopen.bmj.com/site/about/guidelines.xhtml

hypothyroidism				
no	47,235	(87.2 %)	18,008	(88.29 %)
yes	6,933	(12.79 %)	2,387	(11.7 %)
liver disease				
no	50,277	(92.81 %)	19,433	(95.28 %)
yes	3,891	(7.18 %)	962	(4.71 %)
lymphoma				
no	53,762	(99.25 %)	20,315	(99.6 %)
yes	406	(0.74 %)	80	(0.39 %)
metastatic cancer				
no	43,637	(80.55 %)	15,149	(74.27 %)
yes	10,531	(19.44 %)	5,246	(25.72 %)
obesity				
no	47,727	(88.11 %)	18,046	(88.48 %)
yes	6,441	(11.89 %)	2,349	(11.51 %)
other neurological disorders				
no	51,956	(95.91 %)	19,747	(96.82 %)
yes	2,212	(4.08 %)	648	(3.17 %)
paralysis				
no	52,765	(97.4 %)	19,989	(98.01 %)
yes	1,403	(2.59 %)	406	(1.99 %)
peptic ulcer disease excluding bleeding				
no	54,004	(99.69 %)	20,348	(99.76 %)
yes	164	(0.31 %)	47	(0.23 %)
peripheral vascular disorders				
no	49,190	(90.81 %)	18,228	(89.37 %)
yes	4,978	(9.18 %)	2,167	(10.62 %)
psychoses				
no	53,889	(99.48 %)	20,330	(99.68 %)
yes	279	(0.51 %)	65	(0.31 %)
pulmonary circulation disorders				
no	52,476	(96.87 %)	19,929	(97.71 %)
yes	1,692	(3.12 %)	466	(2.28 %)
renal Diseases				
no	45,739	(84.43 %)	17,896	(87.74 %)
yes	8,429	(15.56 %)	2,499	(12.25 %)
rheumatoid arthritis/ collagen vascular diseases				
no	53,262	(98.32 %)	20,115	(98.62 %)
yes	906	(1.67 %)	280	(1.37 %)
solid tumor without metastasis				
no	28,520	(52.65 %)	6,722	(32.95 %)
yes	25,648	(47.34 %)	13,673	(67.04 %)
valvular disease				
no	51,473	(95.02 %)	19,591	(96.05 %)
yes	2,695	(4.97 %)	804	(3.94 %)
weight loss				
no	46,134	(85.16 %)	17,442	(85.52 %)
yes	8,034	(14.83 %)	2,953	(14.47 %)
**HEALTHCARE CHARACTERISTICS**				
total colon resections				
no	51,310	(94.72 %)	20,173	(98.91 %)
yes	2,858	(5.27 %)	222	(1.08 %)
partial colon resections				
no	2,662	(4.91 %)	17,088	(83.78 %)
yes	51,506	(95.08 %)	3,307	(16.21 %)
rectum resections				
no	50,665	(93.53 %)	-	-
yes	3,503	(6.46 %)	20,395	(100.00 %)
colon and rectum resection				
no	50,665	(93.53 %)	16,892	(82.82 %)
yes	3,503	(6.46 %)	3,503	(17.17 %)
weekend surgery				
no	49,473	(91.33 %)	19,603	(96.11 %)
yes	4,695	(8.66 %)	792	(3.88 %)
admission				
referral	36,129	(66.69 %)	16,249	(79.67 %)
emergency case	16,116	(29.75 %)	3,744	(18.35 %)
transfer from other hospital	1,923	(3.55 %)	402	(1.97 %)

**HOSPITAL CHARACTERISTICS**				
annual volume				
colon resection cases (median)	72	(38; 119)	-	-
total colon resection (median)	1	(0;3)	-	-
rectum resections (median)	-	-	26	(11;42)
urbanization				
urban	124	(59.33 %)	119	(59.50 %)
rural	85	(40.66 %)	81	(40.50 %)
ownership				
public	82	(39.23 %)	80	(40.00 %)
non-profit	41	(19.61 %)	39	(19.50 %)
private	86	(41.14 %)	81	(40.50 %)
university hospital				
no	201	(96.17 %)	192	(96.00 %)
yes	8	(3.82 %)	8	(4.00 %)

For peer review only

S7: Bivariate analysis of case-, care- and hospital-related covariates of patient safety including all 54.168 colon resections in 209 hospitals

	IN-HOSPITAL DEATH		POST-OPERATIVE RESPIRATORY FAILURE		RENAL FAILURE		POST-OPERATIVE WOUND INFECTION	
	OR	95% CI	OR	95% CI	OR	95% CI	OR	95% CI
**PATIENT COVARIATES**								
sex								
male	Ref.		Ref.		Ref.		Ref.	
female	0.876***	(0.827 - 0.928)	0.828***	(0.790 - 0.868)	0.742***	(0.707 - 0.778)	0.931**	(0.881 - 0.983)
age	1.052***	(1.049 - 1.055)	1.030***	(1.028 - 1.031)	1.037***	(1.035 - 1.039)	1.008***	(1.006 - 1.009)
alcohol abuse	3.845***	(3.363 - 4.396)	3.862***	(3.400 - 4.387)	3.860***	(3.406 - 4.374)	1.945***	(1.669 - 2.266)
blood loss anemia	0.715**	(0.570 - 0.896)	1.355***	(1.159 - 1.585)	1.200*	(1.025 - 1.405)	1.387***	(1.159 - 1.659)
cardiac arrhythmias	4.338***	(4.084 - 4.608)	3.544***	(3.364 - 3.734)	3.995***	(3.791 - 4.209)	1.766***	(1.660 - 1.878)
chronic pulmonary disease	2.025***	(1.867 - 2.196)	2.120***	(1.978 - 2.273)	1.793***	(1.668 - 1.927)	1.554***	(1.431 - 1.689)
coagulopathy	9.015***	(8.439 - 9.630)	6.370***	(6.011 - 6.751)	7.207***	(6.798 - 7.640)	2.483***	(2.331 - 2.646)
congestive heart failure	4.645***	(4.342 - 4.969)	4.294***	(4.042 - 4.563)	4.382***	(4.127 - 4.652)	1.899***	(1.765 - 2.044)
deficiency anemia	1.013	(0.878 - 1.169)	1.448***	(1.302 - 1.610)	1.416***	(1.268 - 1.582)	1.250***	(1.101 - 1.418)
depression	0.888	(0.779 - 1.012)	1.827***	(1.669 - 2.000)	1.372***	(1.244 - 1.513)	1.923***	(1.741 - 2.125)
diabetes, complicated	2.766***	(2.481 - 3.084)	2.339***	(2.121 - 2.578)	2.946***	(2.678 - 3.240)	1.705***	(1.514 - 1.921)
diabetes, uncomplicated	1.485***	(1.378 - 1.600)	1.559***	(1.465 - 1.659)	1.594***	(1.497 - 1.698)	1.300***	(1.208 - 1.399)
fluid and electrolyte disorders	4.072***	(3.809 - 4.354)	4.463***	(4.229 - 4.709)	5.033***	(4.756 - 5.328)	2.650***	(2.497 - 2.812)
hypertension, complicated	2.124***	(1.896 - 2.380)	2.156***	(1.957 - 2.375)	2.446***	(2.225 - 2.688)	1.453***	(1.288 - 1.638)
hypertension, uncomplicated	0.961	(0.906 - 1.020)	1.266***	(1.207 - 1.329)	1.294***	(1.232 - 1.360)	1.198***	(1.132 - 1.267)
hypothyroidism	0.964	(0.883 - 1.052)	1.227***	(1.147 - 1.312)	1.176***	(1.096 - 1.261)	1.316***	(1.219 - 1.421)
liver disease	6.486***	(6.007 - 7.004)	2.844***	(2.635 - 3.069)	4.573***	(4.254 - 4.917)	1.345***	(1.223 - 1.478)
lymphoma	1.869***	(1.441 - 2.424)	1.319*	(1.030 - 1.690)	1.850***	(1.470 - 2.327)	1.246	(0.939 - 1.654)
metastatic cancer	0.948	(0.881 - 1.021)	1.004	(0.946 - 1.065)	0.827***	(0.777 - 0.881)	1.069	(1.000 - 1.143)
obesity	0.780***	(0.707 - 0.860)	1.173***	(1.092 - 1.259)	1.419***	(1.324 - 1.521)	1.656***	(1.533 - 1.789)
other neurological disorders	2.854***	(2.567 - 3.174)	3.417***	(3.109 - 3.754)	2.834***	(2.575 - 3.119)	1.860***	(1.660 - 2.084)
paralysis	2.709***	(2.373 - 3.092)	3.030***	(2.692 - 3.410)	2.435***	(2.156 - 2.751)	1.874***	(1.629 - 2.156)
peripheral vascular disorders	3.572***	(3.316 - 3.848)	2.745***	(2.562 - 2.941)	3.001***	(2.804 - 3.213)	1.741***	(1.606 - 1.889)
psychoses	1.004	(0.676 - 1.492)	2.578***	(1.978 - 3.361)	1.232	(0.903 - 1.679)	1.845***	(1.352 - 2.517)
pulmonary circulation disorders	3.533***	(3.150 - 3.964)	3.959***	(3.562 - 4.401)	3.185***	(2.863 - 3.543)	1.721***	(1.511 - 1.960)
renal failure	2.772***	(2.592 - 2.966)	2.261***	(2.132 - 2.398)	2.982***	(2.815 - 3.158)	1.524***	(1.419 - 1.636)
rheumatoid arthritis/collagen vascular diseases	1.393**	(1.141 - 1.702)	1.283**	(1.079 - 1.524)	1.373***	(1.156 - 1.630)	1.196	(0.980 - 1.461)
solid tumor without metastasis	0.660***	(0.622 - 0.701)	0.845***	(0.806 - 0.886)	0.760***	(0.724 - 0.798)	0.949	(0.898 - 1.002)
valvular disease	2.580***	(2.332 - 2.855)	2.355***	(2.151 - 2.579)	2.632***	(2.409 - 2.876)	1.426***	(1.275 - 1.594)
weight loss	1.439***	(1.328 - 1.560)	2.418***	(2.269 - 2.576)	2.091***	(1.963 - 2.228)	2.230***	(2.073 - 2.398)
**HEALTHCARE COVARIATES**								
admission								
referral	Ref.		Ref.		Ref.		Ref.	
emergency case	3.066***	(2.852 - 3.297)	2.263***	(2.133 - 2.401)	2.409***	(2.260 - 2.567)	1.461***	(1.368 - 1.559)
transfer from other hospital	5.722***	(5.105 - 6.412)	4.200***	(3.768 - 4.680)	4.308***	(3.870 - 4.795)	1.865***	(1.645 - 2.115)
weekend Surgery								
no	Ref.		Ref.		Ref.		Ref.	
yes	2.704***	(2.498 - 2.927)	2.148***	(1.999 - 2.308)	2.254***	(2.099 - 2.419)	1.382***	(1.265 - 1.509)

total colon resection								
no	Ref.		Ref.		Ref.		Ref.	
yes	3.687***	(3.351 - 4.057)	2.395***	(2.186 - 2.625)	2.954***	(2.704 - 3.227)	1.340***	(1.204 - 1.493)
colon and rectum resection								
no	Ref.		Ref.		Ref.		Ref.	
yes	1.114	(0.991 - 1.252)	1.563***	(1.429 - 1.710)	1.390***	(1.267 - 1.525)	1.757***	(1.593 - 1.937)
**HOSPITAL COVARIATES**								
case volume	1.071	(0.978 - 1.173)	0.934	(0.837 - 1.043)	1.044	(0.944 - 1.155)	1.265***	(1.117 - 1.432)
area								
urban	Ref.		Ref.		Ref.		Ref.	
rural	0.830*	(0.711 - 0.969)	0.849	(0.665 - 1.085)	0.760**	(0.624 - 0.927)	0.837	(0.661 - 1.059)
university hospital								
no	Ref.		Ref.		Ref.		Ref.	
yes	1.946***	(1.411 - 2.683)	1.003	(0.555 - 1.814)	1.767*	(1.113 - 2.806)	3.178***	(1.894 - 5.333)
ownership								
public	Ref.		Ref.		Ref.		Ref.	
non-profit	0.774*	(0.634 - 0.945)	0.915	(0.657 - 1.272)	0.691**	(0.534 - 0.895)	0.615**	(0.452 - 0.835)
private	0.748***	(0.636 - 0.880)	1.025	(0.785 - 1.338)	1.226	(0.999 - 1.504)	0.662**	(0.518 - 0.847)

Note: Elixhauser Groups drug abuse, peptic ulcer disease and AIDS/ HIV were not included into the regression due to low case numbers
*** p<0.001
** p <0.01
* p<0.05

For peer review only

S8: Bivariate analysis of case-, care- and hospital-related covariates of patient safety including all 20.395 rectum resections in 200 hospitals

-	IN-HOSPITAL DEATH		POST-OPERATIVE RESPIRATORY FAILURE		RENAL FAILURE		POST-OPERATIVE WOUND INFECTION	
**PATIENT COVARIATES**	OR	95% CI	OR	95% CI	OR	95% CI	OR	95% CI
sex								
male	Ref.		Ref.		Ref.		Ref.	
female	0.915	(0.796 - 1.050)	0.913*	(0.836 - 0.998)	0.787***	(0.717 - 0.864)	0.877**	(0.801 - 0.960)
age	1.075***	(1.067 - 1.082)	1.031***	(1.027 - 1.034)	1.039***	(1.035 - 1.043)	1.007***	(1.004 - 1.010)
alcohol abuse	2.767***	(1.915 - 3.997)	2.705***	(2.050 - 3.570)	2.531***	(1.916 - 3.345)	1.826***	(1.356 - 2.459)
blood loss anemia	1.984**	(1.270 - 3.100)	1.769**	(1.256 - 2.493)	2.637***	(1.942 - 3.581)	2.264***	(1.619 - 3.165)
cardiac arrhythmias	5.465***	(4.741 - 6.299)	3.457***	(3.124 - 3.825)	3.777***	(3.410 - 4.183)	1.564***	(1.399 - 1.748)
chronic pulmonary disease	2.213***	(1.820 - 2.690)	2.185***	(1.905 - 2.506)	1.835***	(1.587 - 2.122)	1.690***	(1.463 - 1.952)
coagulopathy	9.352***	(8.020 - 10.906)	5.781***	(5.171 - 6.463)	5.702***	(5.091 - 6.386)	2.680***	(2.401 - 2.993)
congestive heart failure	5.853***	(4.995 - 6.858)	4.275***	(3.781 - 4.834)	4.272***	(3.787 - 4.820)	1.793***	(1.560 - 2.060)
deficiency anemia	1.741**	(1.241 - 2.442)	2.119***	(1.696 - 2.648)	2.275***	(1.810 - 2.858)	1.893***	(1.499 - 2.391)
depression	0.872	(0.637 - 1.192)	1.675***	(1.409 - 1.991)	1.686***	(1.411 - 2.014)	1.701***	(1.436 - 2.014)
diabetes, complicated	3.196***	(2.455 - 4.160)	2.073***	(1.692 - 2.540)	3.187***	(2.635 - 3.854)	1.730***	(1.390 - 2.154)
diabetes, uncomplicated	1.653***	(1.388 - 1.969)	1.613***	(1.435 - 1.813)	1.660***	(1.472 - 1.873)	1.341***	(1.184 - 1.518)
fluid and electrolyte disorders	3.961***	(3.387 - 4.633)	4.043***	(3.660 - 4.467)	4.586***	(4.122 - 5.102)	2.563***	(2.324 - 2.826)
hypertension, complicated	2.656***	(2.033 - 3.470)	2.366***	(1.947 - 2.876)	2.681***	(2.225 - 3.230)	1.480***	(1.188 - 1.844)
hypertension, uncomplicated	1.094	(0.951 - 1.257)	1.358***	(1.241 - 1.486)	1.449***	(1.318 - 1.593)	1.159**	(1.057 - 1.271)
hypothyroidism	0.980	(0.790 - 1.215)	1.164*	(1.019 - 1.329)	1.170*	(1.019 - 1.344)	1.125	(0.981 - 1.290)
liver disease	5.887***	(4.864 - 7.124)	2.298***	(1.937 - 2.725)	3.606***	(3.084 - 4.216)	1.539***	(1.279 - 1.850)
lymphoma	2.350*	(1.112 - 4.967)	1.618	(0.882 - 2.967)	1.766	(0.972 - 3.208)	1.683	(0.946 - 2.992)
metastatic cancer	0.979	(0.835 - 1.148)	1.298***	(1.177 - 1.431)	0.978	(0.879 - 1.087)	1.187***	(1.075 - 1.311)
obesity	0.908	(0.726 - 1.135)	1.500***	(1.320 - 1.704)	1.632***	(1.435 - 1.855)	1.653***	(1.455 - 1.878)
other neurological disorders	3.942***	(3.107 - 5.001)	3.107***	(2.569 - 3.757)	2.277***	(1.860 - 2.788)	1.887***	(1.530 - 2.327)
paralysis	3.060***	(2.244 - 4.173)	2.958***	(2.336 - 3.745)	2.816***	(2.220 - 3.572)	1.849***	(1.425 - 2.399)
peripheral vascular disorders	2.521***	(2.118 - 3.000)	1.673***	(1.469 - 1.905)	1.924***	(1.690 - 2.189)	1.531***	(1.341 - 1.748)
psychoses	0.943	(0.291 - 3.056)	0.843	(0.371 - 1.914)	1.865	(0.969 - 3.592)	1.337	(0.660 - 2.707)
pulmonary circulation disorders	6.535***	(5.123 - 8.336)	4.514***	(3.663 - 5.563)	3.734***	(3.022 - 4.613)	2.041***	(1.619 - 2.573)
renal failure	2.984***	(2.532 - 3.518)	2.416***	(2.147 - 2.718)	3.393***	(3.031 - 3.797)	1.688***	(1.488 - 1.915)
rheumatoid arthritis/collagen vascular diseases	1.521	(0.932 - 2.482)	1.582**	(1.140 - 2.196)	1.471*	(1.043 - 2.076)	0.868	(0.580 - 1.301)
solid tumor without metastasis	0.571***	(0.494 - 0.659)	1.002	(0.906 - 1.107)	0.836***	(0.755 - 0.924)	1.090	(0.984 - 1.207)
valvular disease	3.682***	(2.935 - 4.619)	2.693***	(2.257 - 3.214)	2.803***	(2.351 - 3.341)	1.364**	(1.113 - 1.672)
weight loss	1.408***	(1.165 - 1.702)	2.522***	(2.244 - 2.834)	2.072***	(1.842 - 2.331)	2.079***	(1.843 - 2.346)
**HEALTHCARE COVARIATES**								
admission reason								
referral	Ref.		Ref.		Ref.		Ref.	
emergency case	4.038***	(3.450 - 4.727)	2.200***	(1.963 - 2.465)	2.272***	(2.013 - 2.563)	1.656***	(1.474 - 1.862)
transfer from other hospital	6.550***	(4.887 - 8.780)	3.944***	(3.088 - 5.038)	4.248***	(3.337 - 5.406)	2.208***	(1.705 - 2.861)
weekend surgery								
no	Ref.		Ref.		Ref.		Ref.	
yes	3.693***	(2.927 - 4.659)	2.215***	(1.831 - 2.678)	2.177***	(1.809 - 2.620)	1.281*	(1.030 - 1.593)
colon and rectum resection								

no	Ref.		Ref.		Ref.		Ref.	
yes	4.015***	(3.458 - 4.663)	3.095***	(2.781 - 3.444)	2.706***	(2.428 - 3.016)	2.014***	(1.805 - 2.247)
**HOSPITAL COVARIATES**								
case volume	0.743***	(0.661 - 0.835)	0.828**	(0.730 - 0.940)	0.830***	(0.746 - 0.924)	1.079	(0.946 - 1.232)
area								
urban	Ref.		Ref.		Ref.		Ref.	
rural	0.882	(0.680 - 1.143)	0.925	(0.684 - 1.251)	0.895	(0.709 - 1.129)	0.726*	(0.554 - 0.951)
university hospital								
no	Ref.		Ref.		Ref.		Ref.	
yes	1.439	(0.890 - 2.324)	0.857	(0.435 - 1.690)	1.134	(0.696 - 1.847)	3.037***	(1.778 - 5.186)
ownership								
public	Ref.		Ref.		Ref.		Ref.	
non-profit	0.691*	(0.500 - 0.954)	0.887	(0.594 - 1.324)	0.633**	(0.472 - 0.848)	0.605**	(0.428 - 0.854)
private	0.631***	(0.480 - 0.830)	1.070	(0.773 - 1.482)	1.245	(0.988 - 1.571)	0.673**	(0.508 - 0.893)

Note: Elixhauser Groups drug abuse, peptic ulcer disease and AIDS/ HIV were not included into the regression due to a low case numbers

*** p<0.001

** p <0.01

* p<0.05

For peer review only

S9: Multivariate analysis of case-, care- and hospital-related covariates of patient safety including all 54.168 colon resections in 209 hospitals

	IN-HOSPITAL DEATH		POST-OPERATIVE RESPIRATORY FAILURE		RENAL FAILURE		POST-OPERATIVE WOUND INFECTION	
	OR	95% CI	OR	95% CI	OR	95% CI	OR	95% CI
**CASE COVARIATES**								
sex								
male	Ref.		Ref		Ref		Ref	
female	0.937	(0.873 - 1.006)	0.788***	(0.745 - 0.833)	0.683***	(0.645 - 0.725)	0.882***	(0.832 - 0.936)
age	1.050***	(1.046 - 1.053)	1.014***	(1.012 - 1.017)	1.024***	(1.021 - 1.026)	0.998	(0.996 - 1.000)
alcohol abuse	1.484***	(1.250 - 1.761)	1.948***	(1.677 - 2.263)	1.574***	(1.352 - 1.833)	1.304**	(1.106 - 1.539)
blood loss anemia	0.472***	(0.365 - 0.609)	0.982	(0.823 - 1.172)	0.831*	(0.695 - 0.995)	1.154	(0.958 - 1.390)
cardiac arrhythmias	1.640***	(1.518 - 1.772)	1.585***	(1.487 - 1.690)	1.627***	(1.524 - 1.737)	1.183***	(1.100 - 1.271)
chronic pulmonary disease	1.362***	(1.234 - 1.503)	1.459***	(1.347 - 1.581)	1.113*	(1.022 - 1.212)	1.242***	(1.138 - 1.356)
coagulopathy	4.174***	(3.864 - 4.509)	3.117***	(2.920 - 3.327)	3.332***	(3.118 - 3.561)	1.644***	(1.531 - 1.764)
congestive heart failure	1.748***	(1.597 - 1.914)	1.902***	(1.759 - 2.056)	1.678***	(1.551 - 1.815)	1.218***	(1.114 - 1.332)
deficiency anemia	0.778**	(0.660 - 0.918)	1.058	(0.937 - 1.194)	1.096	(0.965 - 1.245)	1.024	(0.897 - 1.169)
depression	0.658***	(0.566 - 0.765)	1.425***	(1.286 - 1.580)	1.070	(0.956 - 1.197)	1.569***	(1.414 - 1.741)
diabetes, complicated	1.297***	(1.131 - 1.487)	1.181**	(1.051 - 1.328)	1.274***	(1.135 - 1.431)	1.191**	(1.045 - 1.358)
diabetes, uncomplicated	1.128*	(1.029 - 1.237)	1.201***	(1.117 - 1.291)	1.151***	(1.067 - 1.241)	1.113**	(1.028 - 1.204)
fluid and electrolyte disorders	1.884***	(1.743 - 2.038)	2.561***	(2.412 - 2.719)	2.882***	(2.704 - 3.072)	1.991***	(1.867 - 2.123)
hypertension, complicated	0.507***	(0.437 - 0.589)	0.735***	(0.649 - 0.832)	0.805***	(0.712 - 0.911)	0.928	(0.807 - 1.067)
hypertension, uncomplicated	0.551***	(0.511 - 0.595)	0.945	(0.890 - 1.003)	0.921*	(0.865 - 0.981)	1.045	(0.980 - 1.115)
hypothyroidism	0.795***	(0.716 - 0.884)	1.110**	(1.027 - 1.200)	1.046	(0.963 - 1.137)	1.206***	(1.112 - 1.309)
liver disease	4.411***	(3.999 - 4.865)	1.373***	(1.253 - 1.506)	2.588***	(2.368 - 2.829)	0.831***	(0.749 - 0.923)
lymphoma	0.983	(0.717 - 1.349)	0.810	(0.613 - 1.070)	1.111	(0.846 - 1.458)	1.029	(0.767 - 1.382)
metastatic cancer	1.524***	(1.376 - 1.687)	1.120**	(1.036 - 1.211)	0.984	(0.906 - 1.070)	1.058	(0.974 - 1.149)
obesity	0.766***	(0.682 - 0.862)	1.165***	(1.073 - 1.265)	1.498***	(1.379 - 1.627)	1.634***	(1.506 - 1.773)
other neurological disorders	1.504***	(1.319 - 1.714)	1.903***	(1.706 - 2.123)	1.515***	(1.352 - 1.699)	1.259***	(1.114 - 1.422)
paralysis	1.481***	(1.261 - 1.740)	1.468***	(1.281 - 1.682)	1.142	(0.989 - 1.318)	1.231**	(1.060 - 1.429)
peripheral vascular disorders	1.926***	(1.757 - 2.111)	1.493***	(1.378 - 1.618)	1.518***	(1.399 - 1.647)	1.239***	(1.136 - 1.351)
psychoses	0.707	(0.449 - 1.113)	2.052***	(1.516 - 2.778)	0.859	(0.600 - 1.229)	1.493*	(1.081 - 2.061)
pulmonary circulation disorders	1.593***	(1.380 - 1.838)	1.864***	(1.649 - 2.108)	1.336***	(1.174 - 1.519)	1.071	(0.933 - 1.231)
renal failure	1.308***	(1.200 - 1.425)	1.112**	(1.035 - 1.196)	1.505***	(1.401 - 1.616)	1.067	(0.984 - 1.156)
rheumatoid arthritis/collagen vascular diseases	1.001	(0.793 - 1.264)	0.971	(0.798 - 1.182)	0.982	(0.806 - 1.197)	1.056	(0.858 - 1.298)
solid tumor without metastasis	0.573***	(0.525 - 0.625)	0.764***	(0.715 - 0.816)	0.707***	(0.660 - 0.757)	0.907**	(0.845 - 0.975)
valvular disease	0.817**	(0.719 - 0.929)	0.862**	(0.772 - 0.961)	0.927	(0.831 - 1.033)	0.927	(0.820 - 1.048)
weight loss	0.891*	(0.809 - 0.980)	1.658***	(1.542 - 1.783)	1.426***	(1.324 - 1.535)	1.774***	(1.643 - 1.916)
**HEALTHCARE COVARIATES**								
admission reason								
referral	Ref.		Ref.		Ref.		Ref.	
emergency case	1.847***	(1.692 - 2.015)	1.413***	(1.320 - 1.513)	1.453***	(1.349 - 1.566)	1.145***	(1.067 - 1.228)
transfer from other hospital	2.528***	(2.193 - 2.915)	1.982***	(1.749 - 2.245)	1.908***	(1.678 - 2.171)	1.223**	(1.071 - 1.397)
weekend surgery								
no	Ref.		Ref.		Ref.		Ref.	
yes	1.669***	(1.515 - 1.839)	1.426***	(1.312 - 1.550)	1.480***	(1.360 - 1.610)	1.080	(0.984 - 1.186)

total colon resection								
no	Ref.		Ref.		Ref.		Ref.	
yes	2.679***	(2.369 - 3.029)	1.639***	(1.472 - 1.825)	2.228***	(1.999 - 2.483)	1.022	(0.913 - 1.143)
colon and rectum resection								
no	Ref.		Ref.		Ref.		Ref.	
yes	1.103	(0.960 - 1.267)	1.524***	(1.378 - 1.686)	1.408***	(1.265 - 1.567)	1.579***	(1.426 - 1.748)
**HOSPITAL COVARIATES**								
case volume	0.968	(0.871 - 1.076)	0.919	(0.807 - 1.047)	0.992	(0.891 - 1.106)	1.168*	(1.030 - 1.325)
area								
urban	Ref.		Ref.		Ref.		Ref.	
rural	1.061	(0.893 - 1.261)	0.863	(0.648 - 1.149)	0.772**	(0.635 - 0.939)	1.032	(0.824 - 1.292)
university hospital								
no	Ref.		Ref.		Ref.		Ref.	
yes	1.303	(0.888 - 1.912)	0.687	(0.338 - 1.397)	1.412	(0.889 - 2.241)	1.981*	(1.171 - 3.352)
ownership								
public	Ref.		Ref.		Ref.		Ref.	
non-profit	1.012	(0.811 - 1.262)	1.057	(0.726 - 1.540)	0.867	(0.670 - 1.122)	0.744*	(0.555 - 0.998)
private	1.244*	(1.026 - 1.507)	1.329	(0.972 - 1.817)	1.937***	(1.563 - 2.400)	0.777*	(0.608 - 0.992)

Note: Elixhauser Groups drug abuse, peptic ulcer disease and AIDS/ HIV were not included into the regression due to a low case numbers

*** p<0.001

** p <0.01

* p<0.05

For peer review only

S10: Multivariate analysis of case-, care- and hospital-related covariates of patient safety including all 20.395 rectum resections in 200 hospitals

	IN-HOSPITAL DEATH		POST-OPERATIVE RESPIRATORY FAILURE		RENAL FAILURE		POST-OPERATIVE WOUND INFECTION	
	OR	95% CI	OR	95% CI	OR	95% CI	OR	95% CI
**CASE COVARIATES**								
sex								
male	Ref.		Ref.		Ref.		Ref.	
female	0.842*	(0.710 - 0.998)	0.842**	(0.759 - 0.934)	0.685***	(0.613 - 0.765)	0.826***	(0.748 - 0.912)
age	1.068***	(1.059 - 1.078)	1.014***	(1.009 - 1.018)	1.021***	(1.016 - 1.026)	0.998	(0.995 - 1.002)
alcohol abuse	1.410	(0.891 - 2.231)	1.656**	(1.200 - 2.286)	1.207	(0.869 - 1.676)	1.143	(0.828 - 1.578)
blood loss anemia	0.829	(0.486 - 1.413)	0.890	(0.607 - 1.305)	1.383	(0.976 - 1.959)	1.579*	(1.113 - 2.241)
cardiac arrhythmias	1.832***	(1.535 - 2.188)	1.662***	(1.471 - 1.878)	1.639***	(1.448 - 1.854)	1.034	(0.909 - 1.176)
chronic pulmonary disease	1.303*	(1.030 - 1.648)	1.510***	(1.291 - 1.766)	1.156	(0.979 - 1.366)	1.369***	(1.175 - 1.594)
coagulopathy	4.309***	(3.600 - 5.158)	3.052***	(2.697 - 3.455)	2.886***	(2.541 - 3.277)	1.770***	(1.570 - 1.996)
congestive heart failure	1.825***	(1.481 - 2.248)	1.849***	(1.583 - 2.159)	1.536***	(1.317 - 1.792)	1.099	(0.929 - 1.300)
deficiency anemia	0.880	(0.593 - 1.304)	1.242	(0.967 - 1.595)	1.357*	(1.052 - 1.750)	1.332*	(1.043 - 1.700)
depression	0.629*	(0.439 - 0.900)	1.222*	(1.005 - 1.486)	1.290*	(1.056 - 1.577)	1.386***	(1.161 - 1.654)
diabetes, complicated	1.584**	(1.145 - 2.192)	1.045	(0.824 - 1.326)	1.441**	(1.153 - 1.801)	1.172	(0.922 - 1.490)
diabetes, uncomplicated	1.124	(0.911 - 1.388)	1.194*	(1.043 - 1.368)	1.191*	(1.036 - 1.369)	1.164*	(1.018 - 1.331)
fluid and electrolyte disorders	1.704***	(1.425 - 2.039)	2.432***	(2.181 - 2.712)	2.855***	(2.542 - 3.206)	1.952***	(1.759 - 2.166)
hypertension, complicated	0.461***	(0.324 - 0.656)	0.757*	(0.591 - 0.970)	0.836	(0.659 - 1.060)	0.911	(0.704 - 1.178)
hypertension, uncomplicated	0.584***	(0.490 - 0.695)	0.980	(0.878 - 1.093)	0.999	(0.890 - 1.121)	0.993	(0.893 - 1.104)
hypothyroidism	0.812	(0.632 - 1.043)	0.993	(0.854 - 1.155)	1.002	(0.856 - 1.174)	1.035	(0.895 - 1.198)
liver disease	4.027***	(3.155 - 5.139)	1.268*	(1.036 - 1.553)	2.312***	(1.920 - 2.783)	1.036	(0.848 - 1.267)
lymphoma	1.308	(0.563 - 3.042)	1.028	(0.532 - 1.986)	0.930	(0.476 - 1.814)	1.320	(0.718 - 2.427)
metastatic cancer	1.376**	(1.118 - 1.694)	1.211**	(1.073 - 1.367)	0.983	(0.863 - 1.121)	1.051	(0.937 - 1.179)
obesity	0.922	(0.708 - 1.202)	1.501***	(1.296 - 1.738)	1.563***	(1.348 - 1.812)	1.638***	(1.431 - 1.876)
other neurological disorders	2.306***	(1.722 - 3.089)	1.879***	(1.507 - 2.343)	1.184	(0.936 - 1.498)	1.376**	(1.098 - 1.725)
paralysis	1.153	(0.785 - 1.695)	1.391*	(1.056 - 1.833)	1.320	(0.999 - 1.744)	1.110	(0.839 - 1.469)
peripheral vascular disorders	1.908***	(1.548 - 2.353)	1.253**	(1.083 - 1.450)	1.369***	(1.181 - 1.587)	1.283***	(1.117 - 1.475)
psychoses	0.867	(0.205 - 3.672)	0.618	(0.246 - 1.551)	2.075	(0.986 - 4.369)	1.072	(0.507 - 2.264)
pulmonary circulation disorders	2.816***	(2.076 - 3.821)	1.935***	(1.522 - 2.460)	1.460**	(1.140 - 1.870)	1.208	(0.941 - 1.551)
renal failure	1.124	(0.917 - 1.377)	1.252**	(1.087 - 1.442)	1.766***	(1.544 - 2.020)	1.278***	(1.108 - 1.473)
rheumatoid arthritis/collagen vascular diseases	0.954	(0.531 - 1.713)	1.248	(0.860 - 1.812)	1.014	(0.683 - 1.507)	0.761	(0.501 - 1.158)
solid tumor without metastasis	0.599***	(0.491 - 0.731)	0.962	(0.846 - 1.094)	0.798***	(0.700 - 0.909)	1.070	(0.946 - 1.211)
valvular disease	1.000	(0.750 - 1.333)	1.075	(0.870 - 1.328)	1.042	(0.844 - 1.287)	0.893	(0.713 - 1.117)
weight loss	0.906	(0.729 - 1.127)	1.810***	(1.590 - 2.060)	1.448***	(1.268 - 1.653)	1.679***	(1.479 - 1.905)
**HEALTHCARE COVARIATES**								
admission reason								
referral	Ref.		Ref.		Ref.		Ref.	
emergency case	2.028***	(1.675 - 2.454)	1.335***	(1.170 - 1.523)	1.342***	(1.169 - 1.540)	1.291***	(1.138 - 1.466)
transfer from other hospital	2.679***	(1.874 - 3.828)	1.859***	(1.406 - 2.459)	1.927***	(1.461 - 2.541)	1.484**	(1.131 - 1.948)
weekend surgery								
no	Ref.		Ref.		Ref.		Ref.	
yes	1.960***	(1.483 - 2.591)	1.427**	(1.150 - 1.770)	1.391**	(1.127 - 1.717)	0.985	(0.784 - 1.238)

colon and rectum resection								
no	Ref.		Ref.		Ref.		Ref.	
yes	2.579***	(2.163 - 3.074)	2.164***	(1.921 - 2.438)	1.859***	(1.645 - 2.100)	1.522***	(1.356 - 1.708)
**HOSPITAL COVARIATES**								
case volume	0.703***	(0.611 - 0.809)	0.844*	(0.725 - 0.982)	0.853**	(0.760 - 0.958)	0.973	(0.854 - 1.109)
area								
urban	Ref.		Ref.		Ref.		Ref.	
rural	1.072	(0.817 - 1.407)	0.904	(0.639 - 1.281)	0.834	(0.670 - 1.037)	0.854	(0.663 - 1.101)
university hospital								
no	Ref.		Ref.		Ref.		Ref.	
yes	1.616	(0.979 - 2.665)	0.853	(0.379 - 1.920)	1.299	(0.829 - 2.037)	2.292**	(1.358 - 3.869)
ownership								
public	Ref.		Ref.		Ref.		Ref.	
non-profit	0.851	(0.614 - 1.179)	1.059	(0.674 - 1.664)	0.762	(0.576 - 1.008)	0.761	(0.553 - 1.048)
private	0.925	(0.682 - 1.254)	1.267	(0.865 - 1.858)	1.597***	(1.256 - 2.030)	0.846	(0.642 - 1.117)

Note: Elixhauser Groups drug abuse, peptic ulcer disease and AIDS/ HIV were not included into the regression due to a low case numbers
*** p<0.001
** p <0.01
* p<0.05

For peer review only

S11: Multivariate analysis of case-, care- and hospital-related covariates of patient safety including 25.805 colon cancer resections in 201 hospitals

	IN-HOSPITAL DEATH		POST-OPERATIVE RESPIRATORY FAILURE		RENAL FAILURE		POST-OPERATIVE WOUND INFECTION	
	OR	95% CI	OR	95% CI	OR	95% CI	OR	95% CI
**CASE COVARIATES**								
sex								
male	Ref.		Ref		Ref		Ref	
female	0.867**	(0.780 - 0.963)	0.810***	(0.747 - 0.878)	0.623***	(0.572 - 0.678)	0.879**	(0.807 - 0.959)
age	1.039***	(1.034 - 1.045)	1.008***	(1.005 - 1.012)	1.018***	(1.014 - 1.022)	0.998	(0.994 - 1.002)
alcohol abuse	1.448*	(1.087 - 1.928)	1.273	(0.982 - 1.650)	1.297*	(1.006 - 1.672)	1.222	(0.919 - 1.624)
blood loss anemia	0.552***	(0.405 - 0.752)	0.926	(0.743 - 1.153)	0.720**	(0.576 - 0.899)	1.199	(0.953 - 1.507)
cardiac arrhythmias	1.616***	(1.440 - 1.814)	1.560***	(1.422 - 1.712)	1.639***	(1.492 - 1.800)	1.229***	(1.108 - 1.363)
chronic pulmonary disease	1.329***	(1.142 - 1.546)	1.450***	(1.288 - 1.632)	1.059	(0.931 - 1.204)	1.213**	(1.064 - 1.384)
coagulopathy	4.057***	(3.617 - 4.550)	3.121***	(2.837 - 3.434)	3.066***	(2.780 - 3.383)	1.679***	(1.515 - 1.861)
congestive heart failure	1.766***	(1.539 - 2.026)	1.848***	(1.649 - 2.071)	1.629***	(1.453 - 1.827)	1.143*	(1.000 - 1.306)
deficiency anemia	0.687***	(0.550 - 0.858)	1.027	(0.880 - 1.198)	0.894	(0.756 - 1.057)	0.945	(0.793 - 1.126)
depression	0.760*	(0.610 - 0.946)	1.409***	(1.212 - 1.637)	1.053	(0.891 - 1.243)	1.557***	(1.336 - 1.813)
diabetes, complicated	1.213	(0.986 - 1.491)	0.969	(0.817 - 1.149)	1.351***	(1.150 - 1.587)	1.077	(0.891 - 1.302)
diabetes, uncomplicated	1.100	(0.965 - 1.253)	1.125*	(1.017 - 1.243)	1.090	(0.982 - 1.210)	1.074	(0.963 - 1.198)
fluid and electrolyte disorders	1.873***	(1.670 - 2.100)	2.338***	(2.145 - 2.547)	2.755***	(2.512 - 3.021)	2.038***	(1.857 - 2.236)
hypertension, complicated	0.511***	(0.409 - 0.639)	0.846	(0.711 - 1.008)	0.795*	(0.667 - 0.947)	0.941	(0.770 - 1.151)
hypertension, uncomplicated	0.624***	(0.559 - 0.698)	0.960	(0.881 - 1.047)	0.996	(0.909 - 1.090)	0.997	(0.909 - 1.094)
hypothyroidism	0.879	(0.752 - 1.028)	1.071	(0.955 - 1.201)	1.018	(0.900 - 1.151)	1.200**	(1.064 - 1.354)
liver disease	3.393***	(2.941 - 3.914)	1.375***	(1.202 - 1.571)	2.346***	(2.065 - 2.664)	0.906	(0.777 - 1.056)
lymphoma	0.816	(0.463 - 1.437)	0.766	(0.477 - 1.231)	1.366	(0.891 - 2.093)	1.283	(0.798 - 2.063)
metastatic cancer	1.414***	(1.273 - 1.571)	1.089*	(1.005 - 1.181)	0.986	(0.905 - 1.073)	1.053	(0.966 - 1.148)
obesity	0.745**	(0.621 - 0.894)	1.123	(0.994 - 1.269)	1.431***	(1.266 - 1.616)	1.513***	(1.337 - 1.713)
other neurological disorders	1.337**	(1.080 - 1.655)	1.818***	(1.532 - 2.157)	1.512***	(1.263 - 1.810)	1.214	(0.997 - 1.479)
paralysis	1.754***	(1.367 - 2.250)	1.402**	(1.135 - 1.733)	1.114	(0.889 - 1.395)	1.209	(0.951 - 1.537)
peripheral vascular disorders	1.644***	(1.420 - 1.904)	1.295***	(1.144 - 1.465)	1.290***	(1.138 - 1.462)	1.217**	(1.068 - 1.387)
psychoses	1.170	(0.607 - 2.253)	1.520	(0.948 - 2.437)	1.222	(0.731 - 2.045)	1.359	(0.820 - 2.252)
pulmonary circulation disorders	1.840***	(1.511 - 2.240)	1.977***	(1.675 - 2.333)	1.360***	(1.141 - 1.620)	1.088	(0.898 - 1.318)
renal failure	1.248***	(1.098 - 1.420)	1.162**	(1.047 - 1.289)	1.538***	(1.389 - 1.702)	1.143*	(1.018 - 1.282)
rheumatoid arthritis/collagen vascular diseases	0.883	(0.559 - 1.395)	0.647*	(0.441 - 0.949)	0.894	(0.626 - 1.277)	0.884	(0.599 - 1.305)
solid tumor without metastasis	0.329***	(0.212 - 0.511)	0.806	(0.520 - 1.252)	0.507**	(0.332 - 0.773)	1.526	(0.894 - 2.605)
valvular disease	0.671***	(0.548 - 0.821)	0.875	(0.747 - 1.024)	0.914	(0.781 - 1.069)	0.920	(0.769 - 1.100)
weight loss	1.007	(0.882 - 1.150)	1.586***	(1.434 - 1.753)	1.366***	(1.233 - 1.514)	1.583***	(1.421 - 1.763)
**HEALTHCARE COVARIATES**								
admission reason								
referral	Ref.		Ref.		Ref.		Ref.	
emergency case	1.822***	(1.613 - 2.058)	1.202***	(1.089 - 1.326)	1.407***	(1.266 - 1.564)	1.091	(0.983 - 1.211)
transfer from other hospital	2.491***	(1.962 - 3.161)	1.697***	(1.371 - 2.101)	1.556***	(1.247 - 1.941)	1.389**	(1.112 - 1.734)
weekend surgery								
no	Ref.		Ref.		Ref.		Ref.	
yes	1.949***	(1.650 - 2.303)	1.377***	(1.187 - 1.598)	1.578***	(1.365 - 1.824)	1.049	(0.885 - 1.244)

total colon resection								
no	Ref.		Ref.		Ref.		Ref.	
yes	2.046***	(1.669 - 2.509)	1.691***	(1.420 - 2.014)	1.703***	(1.422 - 2.038)	1.182	(0.986 - 1.417)
colon and rectum resection								
no	Ref.		Ref.		Ref.		Ref.	
yes	1.025	(0.853 - 1.232)	1.617***	(1.422 - 1.837)	1.394***	(1.214 - 1.600)	1.753***	(1.542 - 1.993)
**HOSPITAL COVARIATES**								
case volume	0.926	(0.810 - 1.058)	0.939	(0.795 - 1.110)	0.994	(0.865 - 1.142)	1.146	(0.975 - 1.348)
area								
urban	Ref.		Ref.		Ref.		Ref.	
rural	1.042	(0.857 - 1.267)	0.815	(0.605 - 1.099)	0.793*	(0.639 - 0.983)	0.964	(0.750 - 1.239)
university hospital								
no	Ref.		Ref.		Ref.		Ref.	
yes	1.301	(0.877 - 1.931)	0.650	(0.318 - 1.328)	1.280	(0.790 - 2.076)	2.209**	(1.281 - 3.807)
ownership								
public	Ref.		Ref.		Ref.		Ref.	
non-profit	1.146	(0.898 - 1.463)	1.098	(0.745 - 1.618)	0.876	(0.660 - 1.162)	0.767	(0.557 - 1.056)
private	1.207	(0.970 - 1.502)	1.316	(0.951 - 1.821)	1.993***	(1.572 - 2.526)	0.776	(0.591 - 1.018)

Note: Elixhauser Groups drug abuse, peptic ulcer disease and AIDS/ HIV were not included into the regression due to a low case numbers
*** p<0.001
** p <0.01
* p<0.05

For peer review only

S12: Multivariate analysis of case-, care- and hospital-related covariates of patient safety including 13.703 rectum cancer resections in 197 hospitals

	IN-HOSPITAL DEATH		POST-OPERATIVE RESPIRATORY FAILURE		RENAL FAILURE		POST-OPERATIVE WOUND INFECTION	
	OR	95% CI	OR	95% CI	OR	95% CI	OR	95% CI
**CASE COVARIATES**								
sex								
male	Ref.		Ref.		Ref.		Ref.	
female	0.912	(0.738 - 1.127)	0.892	(0.789 - 1.009)	0.685***	(0.599 - 0.783)	0.888	(0.789 - 1.000)
age	1.066***	(1.054 - 1.079)	1.013***	(1.007 - 1.019)	1.019***	(1.013 - 1.026)	0.998	(0.993 - 1.003)
alcohol abuse	1.807*	(1.034 - 3.156)	1.603*	(1.076 - 2.389)	1.156	(0.770 - 1.736)	1.138	(0.768 - 1.687)
blood loss anemia	0.823	(0.426 - 1.589)	0.677	(0.415 - 1.102)	1.231	(0.812 - 1.867)	1.466	(0.961 - 2.236)
cardiac arrhythmias	1.704***	(1.361 - 2.135)	1.714***	(1.484 - 1.981)	1.628***	(1.402 - 1.890)	1.050	(0.901 - 1.223)
chronic pulmonary disease	1.540**	(1.149 - 2.065)	1.473***	(1.218 - 1.781)	1.209	(0.986 - 1.482)	1.389***	(1.153 - 1.673)
coagulopathy	3.691***	(2.941 - 4.632)	2.783***	(2.396 - 3.233)	2.487***	(2.126 - 2.909)	1.723***	(1.493 - 1.990)
congestive heart failure	1.784***	(1.363 - 2.335)	1.798***	(1.488 - 2.172)	1.557***	(1.287 - 1.885)	1.028	(0.837 - 1.262)
deficiency anemia	1.008	(0.631 - 1.610)	1.404*	(1.049 - 1.879)	1.527**	(1.129 - 2.066)	1.269	(0.945 - 1.703)
depression	0.665	(0.418 - 1.058)	1.299*	(1.026 - 1.646)	1.359*	(1.060 - 1.742)	1.449***	(1.170 - 1.793)
diabetes, complicated	1.285	(0.830 - 1.990)	1.061	(0.798 - 1.409)	1.190	(0.906 - 1.562)	1.146	(0.860 - 1.528)
diabetes, uncomplicated	1.060	(0.817 - 1.375)	1.194*	(1.022 - 1.396)	1.075	(0.913 - 1.267)	1.243**	(1.067 - 1.448)
fluid and electrolyte disorders	1.693***	(1.353 - 2.117)	2.162***	(1.903 - 2.456)	2.720***	(2.367 - 3.126)	1.966***	(1.738 - 2.223)
hypertension, complicated	0.590*	(0.379 - 0.918)	0.844	(0.627 - 1.137)	0.844	(0.630 - 1.130)	0.894	(0.652 - 1.226)
hypertension, uncomplicated	0.625***	(0.502 - 0.780)	0.994	(0.873 - 1.132)	1.106	(0.962 - 1.272)	0.995	(0.877 - 1.129)
hypothyroidism	0.878	(0.633 - 1.217)	1.032	(0.860 - 1.239)	0.965	(0.791 - 1.177)	0.995	(0.832 - 1.191)
liver disease	3.342***	(2.461 - 4.538)	1.126	(0.879 - 1.443)	2.363***	(1.894 - 2.949)	1.171	(0.924 - 1.486)
lymphoma	1.440	(0.468 - 4.431)	1.006	(0.429 - 2.360)	1.225	(0.519 - 2.893)	1.918	(0.891 - 4.126)
metastatic cancer	1.280*	(1.035 - 1.581)	1.212**	(1.071 - 1.371)	1.015	(0.889 - 1.158)	1.041	(0.925 - 1.172)
obesity	0.953	(0.684 - 1.330)	1.476***	(1.241 - 1.755)	1.607***	(1.349 - 1.915)	1.570***	(1.333 - 1.849)
other neurological disorders	2.294***	(1.562 - 3.369)	2.146***	(1.634 - 2.818)	1.198	(0.885 - 1.621)	1.598**	(1.207 - 2.116)
paralysis	1.418	(0.866 - 2.321)	1.422*	(1.014 - 1.993)	1.249	(0.878 - 1.776)	1.130	(0.799 - 1.600)
peripheral vascular disorders	1.561**	(1.190 - 2.047)	1.151	(0.970 - 1.367)	1.259*	(1.055 - 1.503)	1.423***	(1.214 - 1.669)
psychoses	0.417	(0.045 - 3.841)	0.477	(0.147 - 1.541)	1.904	(0.756 - 4.792)	1.012	(0.413 - 2.481)
pulmonary circulation disorders	3.547***	(2.491 - 5.049)	2.146***	(1.633 - 2.819)	1.567**	(1.179 - 2.082)	1.234	(0.923 - 1.650)
renal failure	0.942	(0.721 - 1.231)	1.125	(0.949 - 1.334)	1.750***	(1.489 - 2.056)	1.233*	(1.039 - 1.462)
rheumatoid arthritis/collagen vascular diseases	1.041	(0.443 - 2.445)	0.825	(0.462 - 1.476)	0.962	(0.544 - 1.701)	0.600	(0.312 - 1.154)
solid tumor without metastasis	0.105***	(0.039 - 0.282)	0.960	(0.353 - 2.613)	1.074	(0.327 - 3.534)	1.648	(0.542 - 5.013)
valvular disease	0.808	(0.550 - 1.185)	1.099	(0.855 - 1.413)	0.922	(0.712 - 1.193)	0.969	(0.743 - 1.265)
weight loss	0.967	(0.745 - 1.255)	1.761***	(1.517 - 2.045)	1.333***	(1.142 - 1.558)	1.557***	(1.345 - 1.803)
**HEALTHCARE COVARIATES**								
admission reason								
referral	Ref.		Ref.		Ref.		Ref.	
emergency case	1.937***	(1.526 - 2.460)	1.199*	(1.018 - 1.413)	1.257*	(1.055 - 1.498)	1.468***	(1.255 - 1.718)
transfer from other hospital	2.503***	(1.487 - 4.213)	2.160***	(1.497 - 3.118)	1.731**	(1.180 - 2.540)	1.805**	(1.263 - 2.578)
weekend surgery								
no	Ref.		Ref.		Ref.		Ref.	
yes	2.137***	(1.417 - 3.224)	1.384*	(1.024 - 1.870)	1.267	(0.940 - 1.708)	1.148	(0.845 - 1.560)

colon and rectum resection								
no	Ref.		Ref.		Ref.		Ref.	
yes	2.422***	(1.940 - 3.024)	2.085***	(1.806 - 2.408)	1.711***	(1.470 - 1.991)	1.627***	(1.415 - 1.871)
**HOSPITAL COVARIATES**								
case volume	0.678***	(0.577 - 0.796)	0.907	(0.764 - 1.075)	0.835**	(0.730 - 0.955)	0.988	(0.851 - 1.147)
area								
urban	Ref.		Ref.		Ref.		Ref.	
rural	1.164	(0.862 - 1.572)	0.951	(0.661 - 1.369)	0.808	(0.632 - 1.033)	0.843	(0.638 - 1.113)
university hospital								
no	Ref.		Ref.		Ref.		Ref.	
yes	1.512	(0.889 - 2.571)	0.807	(0.355 - 1.837)	1.374	(0.840 - 2.246)	2.195**	(1.267 - 3.801)
ownership								
public	Ref.		Ref.		Ref.		Ref.	
non-profit	1.034	(0.725 - 1.473)	1.089	(0.682 - 1.740)	0.797	(0.580 - 1.095)	0.733	(0.518 - 1.037)
private	0.900	(0.635 - 1.276)	1.238	(0.828 - 1.850)	1.807***	(1.375 - 2.376)	0.853	(0.629 - 1.158)

Note: Elixhauser Groups drug abuse, peptic ulcer disease and AIDS/ HIV were not included into the regression due to a low case numbers
*** p<0.001
** p <0.01
* p<0.05

For peer review only

S13: Multivariate analysis of case-, care- and hospital-related covariates of patient safety including 28.363 non-cancer colon resections in 208 hospitals

	IN-HOSPITAL DEATH		POST-OPERATIVE RESPIRATORY FAILURE		RENAL FAILURE		POST-OPERATIVE WOUND INFECTION	
	OR	95% CI	OR	95% CI	OR	95% CI	OR	95% CI
**CASE COVARIATES**								
sex								
male	Ref.		Ref		Ref		Ref	
female	0.988	(0.896 - 1.090)	0.746***	(0.691 - 0.807)	0.748***	(0.689 - 0.811)	0.872***	(0.804 - 0.946)
age	1.056***	(1.051 - 1.060)	1.016***	(1.014 - 1.019)	1.026***	(1.023 - 1.030)	0.997	(0.995 - 1.000)
alcohol abuse	1.483***	(1.190 - 1.847)	2.400***	(1.985 - 2.902)	1.745***	(1.435 - 2.121)	1.350**	(1.099 - 1.657)
blood loss anemia	0.388***	(0.246 - 0.612)	1.296	(0.945 - 1.777)	1.140	(0.831 - 1.565)	1.150	(0.828 - 1.597)
cardiac arrhythmias	1.666***	(1.499 - 1.851)	1.627***	(1.487 - 1.781)	1.605***	(1.464 - 1.760)	1.166**	(1.053 - 1.291)
chronic pulmonary disease	1.405***	(1.231 - 1.603)	1.463***	(1.310 - 1.633)	1.154*	(1.028 - 1.296)	1.264***	(1.123 - 1.422)
coagulopathy	4.166***	(3.747 - 4.632)	2.920***	(2.665 - 3.198)	3.515***	(3.209 - 3.851)	1.602***	(1.451 - 1.769)
congestive heart failure	1.764***	(1.562 - 1.993)	1.972***	(1.770 - 2.197)	1.733***	(1.555 - 1.932)	1.282***	(1.135 - 1.448)
deficiency anemia	0.943	(0.732 - 1.214)	1.219	(0.995 - 1.493)	1.428***	(1.166 - 1.749)	1.188	(0.967 - 1.459)
depression	0.582***	(0.472 - 0.717)	1.488***	(1.288 - 1.719)	1.071	(0.918 - 1.249)	1.564***	(1.354 - 1.807)
diabetes, complicated	1.332**	(1.105 - 1.606)	1.430***	(1.211 - 1.688)	1.187*	(1.005 - 1.404)	1.326**	(1.104 - 1.593)
diabetes, uncomplicated	1.170*	(1.026 - 1.334)	1.304***	(1.172 - 1.450)	1.226***	(1.098 - 1.368)	1.172**	(1.046 - 1.314)
fluid and electrolyte disorders	1.828***	(1.641 - 2.037)	2.715***	(2.495 - 2.955)	2.930***	(2.681 - 3.202)	1.929***	(1.764 - 2.110)
hypertension, complicated	0.527***	(0.430 - 0.645)	0.680***	(0.570 - 0.812)	0.847	(0.712 - 1.008)	0.965	(0.794 - 1.172)
hypertension, uncomplicated	0.489***	(0.440 - 0.543)	0.933	(0.858 - 1.015)	0.861***	(0.789 - 0.940)	1.107*	(1.011 - 1.210)
hypothyroidism	0.729***	(0.631 - 0.842)	1.152*	(1.034 - 1.283)	1.064	(0.949 - 1.192)	1.210***	(1.081 - 1.354)
liver disease	5.708***	(4.967 - 6.559)	1.408***	(1.238 - 1.601)	2.827***	(2.491 - 3.207)	0.802**	(0.694 - 0.925)
lymphoma	1.016	(0.683 - 1.513)	0.858	(0.602 - 1.223)	0.895	(0.624 - 1.284)	0.978	(0.668 - 1.433)
metastatic cancer	-		-		-		-	
obesity	0.768***	(0.657 - 0.897)	1.180**	(1.055 - 1.321)	1.557***	(1.390 - 1.745)	1.737***	(1.557 - 1.938)
other neurological disorders	1.612***	(1.360 - 1.912)	1.949***	(1.686 - 2.253)	1.498***	(1.289 - 1.740)	1.313***	(1.123 - 1.536)
paralysis	1.284*	(1.039 - 1.587)	1.482***	(1.237 - 1.777)	1.124	(0.930 - 1.358)	1.238*	(1.021 - 1.500)
peripheral vascular disorders	2.129***	(1.886 - 2.405)	1.626***	(1.458 - 1.813)	1.675***	(1.500 - 1.871)	1.266***	(1.125 - 1.426)
psychoses	0.518*	(0.279 - 0.963)	2.462***	(1.639 - 3.698)	0.660	(0.405 - 1.074)	1.516	(0.994 - 2.313)
pulmonary circulation disorders	1.348**	(1.094 - 1.662)	1.737***	(1.445 - 2.088)	1.296**	(1.070 - 1.570)	1.040	(0.849 - 1.274)
renal failure	1.381***	(1.228 - 1.553)	1.073	(0.969 - 1.189)	1.494***	(1.351 - 1.653)	0.996	(0.889 - 1.117)
rheumatoid arthritis/collagen vascular diseases	1.046	(0.791 - 1.384)	1.120	(0.885 - 1.416)	0.981	(0.770 - 1.250)	1.143	(0.893 - 1.462)
solid tumor without metastasis	-		-		-		-	
valvular disease	0.954	(0.803 - 1.133)	0.861	(0.738 - 1.004)	0.944	(0.810 - 1.102)	0.942	(0.795 - 1.116)
weight loss	0.799**	(0.696 - 0.917)	1.717***	(1.543 - 1.910)	1.520***	(1.365 - 1.693)	2.013***	(1.803 - 2.248)
**HEALTHCARE COVARIATES**								
admission reason								
referral	Ref.		Ref.		Ref.		Ref.	
emergency case	1.936***	(1.706 - 2.197)	1.727***	(1.564 - 1.906)	1.479***	(1.330 - 1.645)	1.197***	(1.086 - 1.320)
transfer from other hospital	2.610***	(2.169 - 3.140)	2.299***	(1.959 - 2.698)	2.027***	(1.718 - 2.391)	1.202*	(1.015 - 1.423)
weekend surgery								
no	Ref.		Ref.		Ref.		Ref.	
yes	1.577***	(1.397 - 1.780)	1.436***	(1.296 - 1.590)	1.443***	(1.299 - 1.604)	1.095	(0.978 - 1.225)

total colon resection								
no	Ref.		Ref.		Ref.		Ref.	
yes	3.173***	(2.705 - 3.723)	1.604***	(1.395 - 1.843)	2.545***	(2.213 - 2.927)	0.958	(0.830 - 1.107)
colon and rectum resection								
no	Ref.		Ref.		Ref.		Ref.	
yes	1.159	(0.936 - 1.436)	1.320**	(1.116 - 1.562)	1.404***	(1.183 - 1.667)	1.314**	(1.107 - 1.560)
**HOSPITAL COVARIATES**								
case volume	0.961	(0.844 - 1.094)	0.876	(0.753 - 1.018)	0.950	(0.841 - 1.073)	1.135	(0.987 - 1.304)
area								
urban	Ref.		Ref.		Ref.		Ref.	
rural	1.091	(0.886 - 1.345)	0.918	(0.682 - 1.235)	0.775*	(0.631 - 0.953)	1.058	(0.837 - 1.336)
university hospital								
no	Ref.		Ref.		Ref.		Ref.	
yes	1.336	(0.855 - 2.087)	0.712	(0.347 - 1.461)	1.586	(0.999 - 2.520)	1.802*	(1.070 - 3.034)
ownership								
public	Ref.		Ref.		Ref.		Ref.	
non-profit	0.900	(0.688 - 1.177)	1.028	(0.696 - 1.519)	0.836	(0.638 - 1.096)	0.736*	(0.543 - 0.996)
private	1.284*	(1.015 - 1.626)	1.433*	(1.035 - 1.986)	1.893***	(1.505 - 2.380)	0.775	(0.601 - 1.001)

Note: Elixhauser Groups drug abuse, peptic ulcer disease and AIDS/ HIV were not included into the regression due to a low case numbers
*** p<0.001
** p <0.01
* p<0.05

For peer review only

S14: Multivariate analysis of case-, care- and hospital-related covariates of patient safety including 6.692 non-cancer rectum resections in 195 hospitals

	IN-HOSPITAL DEATH		POST-OPERATIVE RESPIRATORY FAILURE		RENAL FAILURE		POST-OPERATIVE WOUND INFECTION	
	OR	95% CI	OR	95% CI	OR	95% CI	OR	95% CI
**CASE COVARIATES**								
sex								
male	Ref.		Ref		Ref		Ref	
female	0.733*	(0.542 - 0.992)	0.739**	(0.601 - 0.908)	0.746**	(0.607 - 0.917)	0.651***	(0.539 - 0.786)
age	1.074***	(1.058 - 1.089)	1.013***	(1.006 - 1.021)	1.023***	(1.016 - 1.031)	1.000	(0.994 - 1.006)
alcohol abuse	0.809	(0.348 - 1.882)	1.801*	(1.008 - 3.219)	1.355	(0.754 - 2.434)	1.080	(0.601 - 1.941)
blood loss anemia	0.797	(0.305 - 2.082)	1.735	(0.877 - 3.432)	1.886	(0.970 - 3.668)	1.927*	(1.014 - 3.663)
cardiac arrhythmias	2.130***	(1.569 - 2.892)	1.573***	(1.243 - 1.990)	1.608***	(1.281 - 2.017)	1.015	(0.795 - 1.295)
chronic pulmonary disease	0.908	(0.601 - 1.371)	1.570**	(1.180 - 2.088)	0.977	(0.726 - 1.315)	1.402*	(1.066 - 1.845)
coagulopathy	5.767***	(4.191 - 7.937)	3.713***	(2.934 - 4.697)	3.703***	(2.949 - 4.650)	1.947***	(1.550 - 2.447)
congestive heart failure	1.851***	(1.309 - 2.617)	1.887***	(1.426 - 2.497)	1.543**	(1.180 - 2.017)	1.269	(0.943 - 1.708)
deficiency anemia	0.765	(0.362 - 1.616)	1.008	(0.606 - 1.675)	1.039	(0.640 - 1.689)	1.430	(0.911 - 2.244)
depression	0.591	(0.326 - 1.073)	1.043	(0.723 - 1.505)	1.220	(0.859 - 1.732)	1.208	(0.869 - 1.679)
diabetes, complicated	2.158**	(1.267 - 3.674)	0.990	(0.627 - 1.564)	2.155***	(1.423 - 3.265)	1.292	(0.821 - 2.032)
diabetes, uncomplicated	1.233	(0.838 - 1.815)	1.133	(0.849 - 1.513)	1.549**	(1.176 - 2.038)	0.950	(0.709 - 1.272)
fluid and electrolyte disorders	1.619**	(1.185 - 2.213)	3.048***	(2.458 - 3.779)	3.006***	(2.430 - 3.719)	1.944***	(1.594 - 2.370)
hypertension, complicated	0.345***	(0.189 - 0.628)	0.657	(0.419 - 1.031)	0.910	(0.596 - 1.390)	1.042	(0.663 - 1.638)
hypertension, uncomplicated	0.511***	(0.377 - 0.694)	0.928	(0.751 - 1.147)	0.813	(0.658 - 1.003)	1.041	(0.851 - 1.273)
hypothyroidism	0.742	(0.491 - 1.121)	0.919	(0.695 - 1.214)	1.051	(0.803 - 1.376)	1.093	(0.845 - 1.416)
liver disease	5.966***	(3.793 - 9.384)	1.550*	(1.068 - 2.252)	2.060***	(1.451 - 2.925)	0.795	(0.540 - 1.172)
lymphoma	0.685	(0.151 - 3.108)	0.930	(0.309 - 2.800)	0.656	(0.221 - 1.944)	0.884	(0.308 - 2.539)
metastatic cancer	-		-		-		-	
obesity	0.853	(0.538 - 1.352)	1.534**	(1.152 - 2.043)	1.400*	(1.054 - 1.861)	1.871***	(1.460 - 2.396)
other neurological disorders	2.558***	(1.586 - 4.126)	1.428	(0.968 - 2.106)	1.117	(0.755 - 1.652)	1.113	(0.754 - 1.642)
paralysis	0.798	(0.421 - 1.514)	1.276	(0.780 - 2.088)	1.508	(0.940 - 2.421)	1.158	(0.714 - 1.881)
peripheral vascular disorders	2.505***	(1.743 - 3.599)	1.502**	(1.119 - 2.018)	1.629***	(1.227 - 2.163)	1.048	(0.774 - 1.419)
psychoses	1.703	(0.166 - 17.483)	1.385	(0.317 - 6.052)	3.384	(0.925 - 12.382)	1.386	(0.359 - 5.356)
pulmonary circulation disorders	1.770	(0.965 - 3.246)	1.636	(0.983 - 2.725)	1.114	(0.667 - 1.859)	1.203	(0.725 - 1.997)
renal failure	1.472*	(1.047 - 2.068)	1.633***	(1.252 - 2.129)	1.755***	(1.368 - 2.250)	1.395*	(1.071 - 1.818)
rheumatoid arthritis/collagen vascular diseases	0.864	(0.358 - 2.087)	1.948*	(1.171 - 3.242)	1.083	(0.609 - 1.925)	1.001	(0.571 - 1.754)
solid tumor without metastasis	-		-		-		-	
valvular disease	1.236	(0.771 - 1.982)	0.999	(0.671 - 1.488)	1.300	(0.888 - 1.903)	0.719	(0.469 - 1.104)
weight loss	0.761	(0.507 - 1.143)	1.969***	(1.503 - 2.580)	1.944***	(1.504 - 2.513)	2.204***	(1.709 - 2.843)
**HEALTHCARE COVARIATES**								
admission reason								
referral	Ref.		Ref.		Ref.		Ref.	
emergency case	2.060***	(1.470 - 2.885)	1.590***	(1.253 - 2.018)	1.382**	(1.094 - 1.746)	0.992	(0.797 - 1.234)
transfer from other hospital	2.583***	(1.488 - 4.487)	1.510	(0.962 - 2.369)	1.949**	(1.269 - 2.993)	1.102	(0.713 - 1.704)
weekend surgery								
no	Ref.		Ref.		Ref.		Ref.	
yes	1.770**	(1.179 - 2.658)	1.391*	(1.012 - 1.912)	1.499*	(1.101 - 2.042)	0.870	(0.613 - 1.235)

colon and rectum resection								
no	Ref.		Ref.		Ref.		Ref.	
yes	2.967***	(2.184 - 4.030)	2.183***	(1.750 - 2.725)	2.190***	(1.771 - 2.709)	1.318*	(1.066 - 1.630)
**HOSPITAL COVARIATES**								
case volume	0.777*	(0.614 - 0.983)	0.793*	(0.642 - 0.978)	0.863	(0.733 - 1.015)	0.956	(0.795 - 1.150)
area								
urban	Ref.		Ref.		Ref.		Ref.	
rural	0.851	(0.536 - 1.351)	0.881	(0.580 - 1.337)	0.872	(0.646 - 1.176)	0.865	(0.613 - 1.220)
university hospital								
no	Ref.		Ref.		Ref.		Ref.	
yes	1.529	(0.712 - 3.283)	0.849	(0.354 - 2.034)	1.176	(0.701 - 1.972)	2.375**	(1.316 - 4.287)
ownership								
public	Ref.		Ref.		Ref.		Ref.	
non-profit	0.556*	(0.323 - 0.959)	1.098	(0.652 - 1.849)	0.702	(0.489 - 1.008)	0.775	(0.516 - 1.166)
private	0.981	(0.594 - 1.621)	1.614*	(1.020 - 2.556)	1.331	(0.960 - 1.846)	0.791	(0.546 - 1.147)

Note: Elixhauser Groups drug abuse, peptic ulcer disease and AIDS/ HIV were not included into the regression due to a low case numbers
*** p<0.001
** p <0.01
* p<0.05

For peer review only

S15 – Figure: Interactions between emergency admission and university hospital status

S16 – Figure: Interactions between transfer from other hospital admission and university hospital status

For peer review only

Reporting checklist for cross sectional study.

Based on the STROBE cross sectional guidelines.

Instructions to authors

Complete this checklist by entering the page numbers from your manuscript where readers will find each of the items listed below.

Your article may not currently address all the items on the checklist. Please modify your text to include the missing information. If you are certain that an item does not apply, please write "n/a" and provide a short explanation.

Upload your completed checklist as an extra file when you submit to a journal.

In your methods section, say that you used the STROBE cross sectionalreporting guidelines, and cite them as:

von Elm E, Altman DG, Egger M, Pocock SJ, Gotzsche PC, Vandenbroucke JP. The Strengthening the Reporting of Observational Studies in Epidemiology (STROBE) Statement: guidelines for reporting observational studies.

		Reporting Item	Page Number
**Title and abstract**			
Title	#1a	Indicate the study's design with a commonly used term in the title or the abstract	1
Abstract	#1b	Provide in the abstract an informative and balanced summary of what was done and what was found	2
**Introduction**			
Background / rationale	#2	Explain the scientific background and rationale for the investigation being reported	4
Objectives	#3	State specific objectives, including any prespecified hypotheses	4
**Methods**			
Study design	#4	Present key elements of study design early in the paper	4
Setting	#5	Describe the setting, locations, and relevant dates, including periods of recruitment, exposure, follow-up, and data collection	5
Eligibility criteria	#6a	Give the eligibility criteria, and the sources and methods of selection of participants.	4-6
	#7	Clearly define all outcomes, exposures, predictors, potential confounders, and effect modifiers. Give diagnostic criteria, if applicable	5-6
Data sources / measurement	#8	For each variable of interest give sources of data and details of methods of assessment (measurement). Describe comparability of assessment methods	5

For peer review only

		if there is more than one group. Give information separately for for exposed and unexposed groups if applicable.	
Bias	#9	Describe any efforts to address potential sources of bias	6-7
Study size	#10	Explain how the study size was arrived at	5-6
Quantitative variables	#11	Explain how quantitative variables were handled in the analyses. If applicable, describe which groupings were chosen, and why	6-7
Statistical methods	#12a	Describe all statistical methods, including those used to control for confounding	6-7
Statistical methods	#12b	Describe any methods used to examine subgroups and interactions	6-7
Statistical methods	#12c	Explain how missing data were addressed	N/A
Statistical methods	#12d	If applicable, describe analytical methods taking account of sampling strategy	N/A
Statistical methods	#12e	Describe any sensitivity analyses	7
**Results**			
Participants	#13a	Report numbers of individuals at each stage of study—eg numbers potentially eligible, examined for eligibility, confirmed eligible, included in the study, completing follow-up, and analysed. Give information separately for for exposed and unexposed groups if applicable.	7
Participants	#13b	Give reasons for non-participation at each stage	N/A
Participants	#13c	Consider use of a flow diagram	7/ Fig 1
Descriptive data	#14a	Give characteristics of study participants (eg demographic, clinical, social) and information on exposures and potential confounders. Give information separately for exposed and unexposed groups if applicable.	7-8, Table 1
Descriptive data	#14b	Indicate number of participants with missing data for each variable of interest	N/A
Outcome data	#15	Report numbers of outcome events or summary measures. Give information separately for exposed and unexposed groups if applicable.	7-8 / Fig 1, Table 1
Main results	#16a	Give unadjusted estimates and, if applicable, confounder-adjusted estimates and their precision (eg, 95% confidence interval). Make clear which confounders were adjusted for and why they were included	10-11 / Table 2-3, Supp. 7-16)
Main results	#16b	Report category boundaries when continuous variables were categorized	N/A
Main results	#16c	If relevant, consider translating estimates of relative risk into absolute risk for a meaningful time period	N/A

For peer review only

Other analyses	#17	Report other analyses done—e.g., analyses of subgroups and interactions, and sensitivity analyses	11
**Discussion**			
Key results	#18	Summarise key results with reference to study objectives	14
Limitations	#19	Discuss limitations of the study, taking into account sources of potential bias or imprecision. Discuss both direction and magnitude of any potential bias.	15
Interpretation	#20	Give a cautious overall interpretation considering objectives, limitations, multiplicity of analyses, results from similar studies, and other relevant evidence.	14-15
Generalisability	#21	Discuss the generalisability (external validity) of the study results	14-15
**Other Information**			
Funding	#22	Give the source of funding and the role of the funders for the present study and, if applicable, for the original study on which the present article is based	17

Notes:

- 15: 7-8 / Fig 1
- 16a: 10-11 / Table 2-3, Supp. 7-16) The STROBE checklist is distributed under the terms of the Creative Commons Attribution License CC-BY. This checklist was completed on 15. October 2021 using https://www.goodreports.org/, a tool made by the EQUATOR Network in collaboration with Penelope.ai

For peer review only

BMJ Open

The relationships between multiple patient safety outcomes and healthcare and hospital-related risk factors in colorectal resection cases: Cross-sectional evidence from a nationwide sample of 232 German hospitals

Journal:	*BMJ Open*
Manuscript ID	bmjopen-2021-058481.R2
Article Type:	Original research
Date Submitted by the Author:	06-Jul-2022
Complete List of Authors:	Walther, Felix; TU Dresden Faculty of Medicine Carl Gustav Carus, Center for Evidence-based Healthcare; University Hospital Carl Gustav Carus, Quality and Medical Risk Management<br>Schmitt, Jochen; TU Dresden Faculty of Medicine Carl Gustav Carus, Center for Evidence-based Healthcare<br>Eberlein-Gonska, Maria; University Hospital Carl Gustav Carus, Quality and Medical Risk Management<br>Kuhlen, Ralf; Initiative Qualitätsmedizin e.V.<br>Scriba, Peter; Initiative Qualitätsmedizin e.V.<br>Schoffer, Olaf; TU Dresden Faculty of Medicine Carl Gustav Carus, Center for Evidence-based Healthcare<br>Roessler, Martin; TU Dresden Faculty of Medicine Carl Gustav Carus, Center for Evidence-based Healthcare
**Primary Subject Heading**:	Health services research
Secondary Subject Heading:	Surgery
Keywords:	Quality in health care < HEALTH SERVICES ADMINISTRATION & MANAGEMENT, Colorectal surgery < SURGERY, Health & safety < HEALTH SERVICES ADMINISTRATION & MANAGEMENT

SCHOLARONE™
Manuscripts

I, the Submitting Author has the right to grant and does grant on behalf of all authors of the Work (as defined in the below author licence), an exclusive licence and/or a non-exclusive licence for contributions from authors who are: i) UK Crown employees; ii) where BMJ has agreed a CC-BY licence shall apply, and/or iii) in accordance with the terms applicable for US Federal Government officers or employees acting as part of their official duties; on a worldwide, perpetual, irrevocable, royalty-free basis to BMJ Publishing Group Ltd ("BMJ") its licensees and where the relevant Journal is co-owned by BMJ to the co-owners of the Journal, to publish the Work in this journal and any other BMJ products and to exploit all rights, as set out in our licence.

The Submitting Author accepts and understands that any supply made under these terms is made by BMJ to the Submitting Author unless you are acting as an employee on behalf of your employer or a postgraduate student of an affiliated institution which is paying any applicable article publishing charge ("APC") for Open Access articles. Where the Submitting Author wishes to make the Work available on an Open Access basis (and intends to pay the relevant APC), the terms of reuse of such Open Access shall be governed by a Creative Commons licence – details of these licences and which Creative Commons licence will apply to this Work are set out in our licence referred to above.

Other than as permitted in any relevant BMJ Author's Self Archiving Policies, I confirm this Work has not been accepted for publication elsewhere, is not being considered for publication elsewhere and does not duplicate material already published. I confirm all authors consent to publication of this Work and authorise the granting of this licence.

For peer review only

Long title: **The relationships between multiple patient safety outcomes and healthcare and hospital-related risk factors in colorectal resection cases: Cross-sectional evidence from a nationwide sample of 232 German hospitals**

Abbreviated title/ Short running head: **Patient safety in colorectal resections**

Felix Walther[1,2*], Jochen Schmitt[1], Maria Eberlein-Gonska[2], Ralf Kuhlen[3], Peter C. Scriba[3], Olaf Schoffer[1], Martin Roessler[1]

1 Center for Evidence-based Healthcare, Medical Faculty Carl Gustav Carus, TU Dresden, Dresden, Germany

2 Quality and Medical Risk Management, University Hospital Carl Gustav Carus, Dresden, Germany

3 Initiative Qualitätsmedizin e.V., Berlin, Germany

*** Corresponding author**

Felix.walther@ukdd.de

word (manuscript): 2979

Words (abstract): 300 / 300

For peer review only - http://bmjopen.bmj.com/site/about/guidelines.xhtml

Abstract

objectives: Studies analyzing colorectal resections usually focus on a specific outcome (e.g., mortality) and/ or specific risk factors at the individual (e.g., comorbidities) or hospital (e.g., volume) level. Comprehensive evidence across different patient safety outcomes, risk factors and patient groups is still scarce. Therefore the aim of this analysis was to investigate consistent relationships between multiple patient safety outcomes, healthcare and hospital risk factors in colorectal resection cases.

design: Cross-sectional study

setting: German inpatient routine care data of colorectal resections between 2016 to 2018

participants: We analyzed 54,168 colon resection and 20,395 rectum resection cases treated in German hospitals. The German Inpatient Quality Indicators were used to define colon resections and rectum resections transparently.

primary outcome measures: Additionally to in-hospital death, post-operative respiratory failure, renal failure and post-operative wound infections we included multiple patient safety outcomes as primary outcomes / dependent variables for our analysis. Healthcare (e.g., weekend surgery), hospital (e.g., volume) and case (e.g., age) characteristics served as independent covariates in a multilevel logistic regression model. The estimated regression coefficients were transferred into odds ratios (OR).

results: Weekend surgery, emergency admissions and transfers from other hospitals were significantly associated (ORs ranged from 1.1 to 2.6) with poor patient safety outcome (i.e., death, renal failure, post-operative respiratory failure) in colon resections and rectum resections. Hospital characteristics showed heterogeneous effects. In colon resections hospital volume was associated with insignificant or adverse associations (post-operative wound infections: OR 1.168 (95% CI 1.030 - 1.325)) to multiple patient safety outcomes. In rectum resections hospital volume was protectively associated with death, renal failure and post-operative respiratory failure (ORs ranged from 0.7 to 0.8).

conclusions: Transfer from other hospital and emergency admission are constantly associated with poor patient safety outcome. Hospital variables like volume, ownership or localization did not show consistent relationships to patient safety outcomes.

.

Keywords: patient safety; health services research; quality of care; colon surgery; rectum surgery

For peer review only - http://bmjopen.bmj.com/site/about/guidelines.xhtml
For peer review only

Strengths and limitations of this study

- Large and current sample providing a broad span of cases, hospital types, ownerships, and locations.
- Comprehensive analysis of multiple patient safety outcomes and multiple sets (case, healthcare, hospital) of risk factors
- Use of previously validated outcomes that were reported to occur most likely during hospitalization
- Accounting data lack information on patient history, medication, length of anesthesia, staff-to-patient ratios, surgeon volumes, centralization and which of the coded diagnoses had been present on admission.

For peer review only

For peer review only - http://bmjopen.bmj.com/site/about/guidelines.xhtml

Introduction

Measuring, assuring and improving patient safety are important objectives regarding patient outcome, payment, and accreditation in colorectal resections. One of the most frequently used outcome indicator in colorectal resections is in-hospital mortality.[1] However, it has been stressed that patient safety is reflected in both mortality and non-mortality outcomes.[2, 3] Therefore the measurement of outcomes beyond mortality is necessary for a comprehensive assessment of patient safety and care quality.[1] Additionally various risk factors for a poor patient safety outcome were analyzed in previous studies. Besides patient characteristics (e.g., age, sex, comorbidities), especially the influence of healthcare (e.g., weekend surgery, emergency, transfer from other hospital) and hospital variables (e.g., volume, urbanization degree) were widely discussed. Weekend surgery,[4-6] emergency admission,[7, 8] transfer from other hospitals,[9-11] and case volume[12, 13] were found to have significant effects on mortality in colorectal resections. Analogous to patient outcomes, previous studies usually considered only subsets of these risk factors without analyzing them together.

A comprehensive analysis of patient safety and its covariates in colorectal resections should take multiple outcomes and multiple risk factors into account.[14] To our knowledge, such comprehensive analyses have rarely been reported. Based on that assumption, our analysis aimed to investigate whether healthcare and hospital characteristics are associated with multiple patient safety outcomes in colorectal resections. Using a 3-year sample (2016-2018) of German inpatient claims data we investigated relationships between case, healthcare, and hospital characteristics and the patient safety outcomes in-hospital death, post-operative respiratory failure, renal failure and post-operative wound infections in colorectal resections.

Materials and Methods

This explorative cross-sectional analysis was embedded into the IMPRESS study. The IMPRESS study was a cluster-randomized trial evaluating the effects of clinical peer review on mortality in patients ventilated >24h nested in a prospective cohort study of 232 participating hospitals. Details, baseline, explorative and confirmatory results of the IMPRESS study were published previously.[15-18] The study has been registered at ISCRTN (ISRCTN10188560).[19] The identification of possible covariates of mortality and non-mortality outcomes in colorectal resections was a secondary aim of the IMPRESS study.

For peer review only - http://bmjopen.bmj.com/site/about/guidelines.xhtml

Data sources

The data used in this study were derived from two routine data sets. We used claims data according to German law regulating inpatient claims data (§21 Krankenhausentgeltgesetz) to gather information concerning age, sex, reason of admission, discharge destination, diagnoses/comorbidities (ICD-10-GM) and medical/ surgical procedures (OPS codes). We applied the predefined groups of the Elixhauser comorbidity index and its coding modifications for ICD-10 (supplement S1) to adjust for relevant comorbidities. The Elixhauser comorbidity index is a score used to adjust for chronic or non-acute comorbidities in routine data sets.[20, 21] To assess hospital characteristics (ownership, university hospital status, urbanization) we used data from the German hospital register („Deutsches Krankenhausverzeichnis").

Study participation, privacy, and ethics

All participating hospitals submitted a written consent regarding participation prior to the start of the IMPRESS study. The data trust site at Koordinierungszentrum für Klinische Studien (KKS) Dresden ensured the anonymization of the data. The Center for Evidence-Based Healthcare (ZEGV) Dresden analyzed the anonymized data. The ethics committee of the TU Dresden approved the study protocol. The study protocol has been officially registered at the institutional review board (IRB): Office for Human Research Protections (OHRP); identification numbers: IRB00001473 and IORG0001076; date of approval: 24/04/2017

Patient and Public Involvement

This cross-sectional analysis used observational routine data based on predefined outcomes and covariates without intervention and did not involve patients or the public in the design, conduct, reporting, or dissemination plans of our research.

Population

232 hospitals participated in the IMPRESS study (Figure 1). We included all cases with a colon and/ or rectum resection in the participating hospitals in 2016-2018. Due to anonymization, the data do not contain a patient-relation. Therefore patients admitted more than once entered the analysis as multiple hospital cases. For each hospital case, all of the documented information in terms of diagnoses and medical/ surgical procedures during hospitalization was available. We used the definitions of the German Inpatient Quality Indicators (GIQI) to define and distinguish partial colon resections (supplement S2), total colon resections (supplement S3) and rectum resections (supplement S4).[22] Hospitals without colon or rectum resections were excluded.

For peer review only - http://bmjopen.bmj.com/site/about/guidelines.xhtml

Outcomes and covariates

Following evidence from a previous study, in-hospital death, respiratory failure, renal failure and wound-infection can be validly operationalized in hospital discharge data.[23] Hence, we analyzed these outcomes in accordance with previously tested case definitions as presented in supplement S5. The outcomes death, post-operative respiratory failure, renal failure, and post-operative wound infections were selected as dependent variables.

The independent variables were classified into three groups:

1) case (age, sex, Elixhauser comorbidities)[20, 21],
2) healthcare (admission date, surgical procedures/OPS codes, reason for admission, discharge destination) and
3) hospital (case volume, ownership, university hospital status, urbanization degree rural/urban).

This study focused on healthcare and hospital variables. Case variables were primarily used for adjustment. To adjust for all potentially relevant risk factors available in the data, the estimations included the full set of independent case, healthcare, and hospital variables. Case level included age, sex and Elixhauser comorbidities.[21] Healthcare level included admission reason (referral/ emergency case/ transfer from other hospital), weekend surgery (identifiable via time stamp of the procedure), and total resection of the colon and resections of the colon and rectum. Hospital level included hospital case volume, degree of urbanization (rural/ urban), university hospital status, and ownership (public/ non-profit/ private). Case volume entered the regression models in logarithmic form. This transformation of hospital volume data captures that volume-outcome relationships may be more pronounced at low case volumes.[24]

Statistical methods

We described case, healthcare, and hospital characteristics using absolute and relative frequencies in case of categorical variables. For continuous variables, we reported median, 1st and 3rd quartile. Relationships between patient safety outcomes and case-level, healthcare-level, and hospital-level risk factors were estimated using multilevel logistic regression models. These models contained a random intercept at the hospital level to capture the correlation of patient outcomes within hospitals.[25] Estimations were conducted separately for cases with colon and rectum resection in bivariate and multivariate analyses. To improve interpretability of estimated effect sizes, we transformed the estimated regression coefficients into odds ratios. An estimated effect was considered statistically significant if its p-value was below 5%. Statistical analysis was performed using Stata 15.1.

For peer review only - http://bmjopen.bmj.com/site/about/guidelines.xhtml

Sensitivity analysis

In the preliminary research it became apparent that the literature distinguishes between colorectal cancer surgery and general colorectal resections.[1, 12, 13, 26, 27] Therefore, with respect to possible effect modifications, we explored differences between cases with and without a cancer diagnosis. The same applies to reported interactions between admission reason and the status of university hospitals compared to non-university hospitals.[16] Therefore we also reviewed these interactions to detect possible effect modifications.

Results

Overall, 71,060 cases with colon and/ or rectum resection were included in the analysis. Separating colon resections and rectum resections a total of 54,168 colon resection cases were treated in 209 hospitals. 20,395 cases of rectum resections were treated in 200 hospitals. If both colon and rectum resection were documented (3,503 cases), the case was analyzed for both groups.

>>>Figure 1 – Flowchart of hospitals included for analysis<<<

The minority of included cases received combined colorectal resections (partial and total colon, colon and rectum). Emergency case admission or transfer from other hospitals were less frequent than referral. Compared to rectum resections, colon resections were more often surgically treated on weekends (8.6% vs. 3.8%), admitted as an emergency case (29.7% vs. 18.3%) or transferred from other hospitals (3.5% vs. 1.9%). The same applies to the rate of poor patient safety outcomes. Colon resection cases revealed higher rates of in-hospital death (9.6% vs. 4.2%), post-operative respiratory failure (16.7% vs. 12.2%), renal failure (15.2% vs. 10.3%) and post-operative wound infections (11.3% vs. 11.2%) than rectum resection cases.

The majority of the analyzed hospitals were localized in urban regions (59%). Most were in private (40%) or public (40%) ownership. The annual median hospital case volume was 72 (Q1 =38; Q3=116) for colon and 26 (Q1=11; Q3=42) for rectum resections.

The median age ranged from 67-68 years (Table 1). Male and female sex in colon and rectum resections were approximately equally represented. For Elixhauser comorbidities, the most frequent codes were solid tumor without metastasis (colon: 47.3%, rectum: 67.0%), uncomplicated hypertension (colon and rectum: 47.6%) and fluid and electrolyte disorders (colon: 45.2%, rectum: 40.5%) in both procedure groups. Descriptive results for all Elixhauser comorbidities are presented in supplement S6.

For peer review only

For peer review only - http://bmjopen.bmj.com/site/about/guidelines.xhtml

Table 1 – Case and hospital characteristics of colon and rectum resections

	COLON RESECTION		RECTUM RESECTION	
	n	% / Q1; Q3	n	% / Q1; Q3
number of cases	54,168	(100.0%)	20,395	(100.0%)
**PATIENT SAFETY OUTCOMES**				
in-hospital death				
no	48,914	(90.31 %)	19,525	(95.73 %)
yes	5,254	(9.68 %)	870	(4.26 %)
post-operative respiratory failure				
no	45,074	(83.21 %)	17,901	(87.77 %)
yes	9,094	(16.78 %)	2,494	(12.22 %)
renal failure				
no	45,920	(84.77 %)	18,279	(89.62 %)
yes	8,248	(15.22 %)	2,116	(10.37 %)
post-operative wound infections				
no	48,013	(88.63 %)	18,109	(88.79 %)
yes	6,155	(11.36 %)	2,286	(11.20 %)
**HEALTHCARE CHARACTERISTICS**				
colon resection				
total	2,662	(4.91 %)	-	-
partial	51,310	(94.72 %)	-	-
both	196	(0.36 %)	-	-
rectum resection				
no	50,665	(93.53 %)	-	-
yes	3,503	(6.46 %)	20,395	(100.00 %)
colon and rectum resection				
no	50,665	(93.53 %)	16,892	(82.82 %)
yes	3,503	(6.46 %)	3,503	(17.17 %)
weekend surgery				
no	49,473	(91.33 %)	19,603	(96.11 %)
yes	4,695	(8.66 %)	792	(3.88 %)
admission reason				
referral	36,129	(66.69 %)	16,249	(79.67 %)
emergency case	16,116	(29.75 %)	3,744	(18.35 %)
transfer from other hospital	1,923	(3.55 %)	402	(1.97 %)
**HOSPITAL CHARACTERISTICS**				
hospitals included	209	(100.00%)	200	(100.00%)
annual volume				
colon resection cases (median)	72	(38; 119)	-	-
total colon resection (median)	1	(0;3)	-	-
rectum resections (median)	-	-	26	(11;42)
urbanization				
urban			119	(59.50 %)

For peer review only - http://bmjopen.bmj.com/site/about/guidelines.xhtml

rural	124	(59.33 %)	81	(40.50 %)
	85	(40.66 %)		
ownership				
public	82	(39.23 %)	80	(40.00 %)
non-profit	41	(19.61 %)	39	(19.50 %)
private	86	(41.14 %)	81	(40.50 %)
university hospital				
no	201	(96.17 %)	192	(96.00 %)
yes	8	(3.82 %)	8	(4.00 %)
**CASE CHARACTERISTICS**				
Age				
median	68	(56; 77)	67	(57; 77)
sex				
male	26,954	(49.76 %)	10,367	(50.83 %)
female	27,214	(50.23 %)	10,028	(49.16 %)
Elixhauser comorbidities […][a]				

Legend: Q1: 1st quartile Q3: 3rd quartile

[a] results of Elixhauser comorbidities (e.g. alcohol abuse, blood loss anemia, cardiac arrhythmias …) are presented in supplement S6

For peer review only

The bivariate analysis provided in supplements S7-S8 was performed to identify unadjusted effects of single covariates on the outcomes. The following multivariable analysis, focusing on healthcare and hospital level (Tables 2-3), was performed to achieve adjusted and robust effects.

Healthcare covariates

Admission as an emergency case or transfer from another hospital were associated to multiple poor patient safety outcomes in both groups. For example higher odds of in-hospital death were related to emergency admission in colon (OR 1.84 (95% CI, 1.69-2.01) and rectum resections (OR 2.02 (95% CI, 1.67-2.45) compared to referral hospital admissions. The same applies to transfer from other hospital, the odds of in-hospital death were higher in colon (OR 2.52 (95% CI, 2.19-2.91)) and rectum resections (OR 2.67 (95% CI, 1.87-3.82)). Except of post-operative wound infections, weekend surgery was associated with worsened patient safety outcome in both groups.

Hospital covariates

While most of the healthcare-level covariates showed similar associations in both groups, hospital covariates showed insignificant or heterogeneous effects.

A higher annual case volume of colon resections indicated a higher risk of post-operative wound infections (OR 1.16 (95% CI, 1.03-1.32)). The remaining associations between annual case volume of colon resections and patient safety outcomes were insignificant. A higher annual volume of rectum resections was associated with lower risks of in-hospital death (OR 0.70 (95% CI, 0.61-0.80)), post-operative respiratory failure (OR 0.84 (95% CI, 0.72-0.98)) and renal failure (OR 0.85 (95% CI, 0.76-0.95)).

Rural localization showed lower odds of renal failure (OR 0.77 (95% CI, 0.63-0.93)) in cases with only colon resections.

Treatment in university hospitals was associated with increased odds of post-operative wound infections in colon (OR 1.98 (95% CI, 1.17-3.35)) and rectum resections (OR 2.29 (95% CI, 1.35-3.86)) compared to treatment in non-university hospitals.

The hospital ownership revealed differences between both groups and patient safety outcomes. Non-profit (OR, 0.74 (95% CI, 0.55-0.99)) or private (OR 0.77, (95% CI, 0.60-0.99)) ownership was associated with lower risks of post-operative wound infections in colon resections. In contrast, odds of in-hospital death (OR 1.24, (95% CI, 1.02-1.50)) and renal failure (OR 1.93, (95% CI, 1.56-2.40)) in colon resections were higher in private

For peer review only - http://bmjopen.bmj.com/site/about/guidelines.xhtml

hospitals. Rectum resections did not show significant associations of ownership and patient safety outcomes except for higher odds of renal failure (OR 1.59, (95% CI, 1.25-2.03)) in private hospitals.

Case covariates

Female sex was consistently associated with better outcomes in both groups, except of a borderline-insignificant association with in-hospital death (OR 0.93, (95% CI, 0.87-1.00)) in colon resections. Age was associated with higher risks of in-hospital death, post-operative respiratory failure and renal failure in both groups. Regarding post-operative wound infections, age was a borderline-insignificant protective factor in colon (OR 0.99, (95% CI, 0.99-1.00)) and rectum (OR 0.99, (95% CI, 0.99-1.00)) resections. Of all Elixhauser comorbidities analysed, coagulopathies showed the highest ORs for poor patient safety outcomes including higher ORs of death in colon (OR 4.17 (95% CI 3.864 - 4.509)) or rectum (OR 4.30 (95% CI 3.600 - 5.158)) resections. The same applies to other patient safety outcomes like post-operative respiratory failure in colon (OR 3.117 (95% CI 2.920 - 3.327)) or rectum (OR 3.052 (95% CI 2.697 - 3.455)) resections, renal failure in colon (OR 3.332 (95% CI 3.118 - 3.561)) and rectum (OR 2.886 (95% CI 2.541 - 3.277)) resections and post-operative wound infections in colon (OR 1.644 (95% CI 1.531 - 1.764)) or rectum (OR 1.770 (95% CI 1.570 - 1.996)) resections. Along with coagulopathies, fluid and electrolyte disorders, peripheral vascular disorders, congestive heart failure, chronic pulmonary disease, cardiac arrhythmias and pulmonary circulation disorders were also associated with multiple poor patient outcomes in both procedure groups. The multivariate results for the remaining Elixhauser groups can be found in supplements S9-S10.

We reviewed differences in results of stratified analyses for cases with and without cancerous colon and rectum resections. Significant effect reversals were not observed (supplements S11-S14). The review also did not reveal differences between university and non-university hospitals in terms of emergency admission or transfer from other hospital (supplements S15-S16). Therefore, a stratification between cases with and without cancer or university and non-university hospitals has not been applied.

For peer review only - http://bmjopen.bmj.com/site/about/guidelines.xhtml

Table 2 – Multivariate analysis of patient safety outcomes in 54,168 colon resections in 209 hospitals

	IN-HOSPITAL DEATH		POST-OPERATIVE RESPIRATORY FAILURE		RENAL FAILURE		POST-OPERATIVE WOUND INFECTION	
	OR	95% CI	OR	95% CI	OR	95% CI	OR	95% CI
**HEALTHCARE COVARIATES**								
admission reason								
referral	Ref.		Ref.		Ref.		Ref.	
emergency case	1.847***	(1.692 - 2.015)	1.413***	(1.320 - 1.513)	1.453***	(1.349 - 1.566)	1.145***	(1.067 - 1.228)
transfer from other hospital	2.528***	(2.193 - 2.915)	1.982***	(1.749 - 2.245)	1.908***	(1.678 - 2.171)	1.223**	(1.071 - 1.397)
weekend surgery								
no	Ref.		Ref.		Ref.		Ref.	
yes	1.669***	(1.515 - 1.839)	1.426***	(1.312 - 1.550)	1.480***	(1.360 - 1.610)	1.080	(0.984 - 1.186)
total colon resection								
no	Ref.		Ref.		Ref.		Ref.	
yes	2.679***	(2.369 - 3.029)	1.639***	(1.472 - 1.825)	2.228***	(1.999 - 2.483)	1.022	(0.913 - 1.143)
colon and rectum resection								
no	Ref.		Ref.		Ref.		Ref.	
yes	1.103	(0.960 - 1.267)	1.524***	(1.378 - 1.686)	1.408***	(1.265 - 1.567)	1.579***	(1.426 - 1.748)
**HOSPITAL COVARIATES**								
case volume	0.968	(0.871 - 1.076)	0.919	(0.807 - 1.047)	0.992	(0.891 - 1.106)	1.168*	(1.030 - 1.325)
area								
urban	Ref.		Ref.		Ref.		Ref.	
rural	1.061	(0.893 - 1.261)	0.863	(0.648 - 1.149)	0.772**	(0.635 - 0.939)	1.032	(0.824 - 1.292)
university hospital								
no	Ref.		Ref.		Ref.		Ref.	
yes	1.303	(0.888 - 1.912)	0.687	(0.338 - 1.397)	1.412	(0.889 - 2.241)	1.981*	(1.171 - 3.352)
ownership								
public	Ref.		Ref.		Ref.		Ref.	
non-profit	1.012	(0.811 - 1.262)	1.057	(0.726 - 1.540)	0.867	(0.670 - 1.122)	0.744*	(0.555 - 0.998)
private	1.244*	(1.026 - 1.507)	1.329	(0.972 - 1.817)	1.937***	(1.563 - 2.400)	0.777*	(0.608 - 0.992)
**CASE COVARIATES**								
sex								
male	Ref.		Ref		Ref		Ref	
female	0.937	(0.873 - 1.006)	0.788***	(0.745 - 0.833)	0.683***	(0.645 - 0.725)	0.882***	(0.832 - 0.936)
age	1.050***	(1.046 - 1.053)	1.014***	(1.012 - 1.017)	1.024***	(1.021 - 1.026)	0.998	(0.996 - 1.000)
Elixhauser comorbidities […][a]								

Legend:
*** p<0.001
** p <0.01
* p<0.05
[a] results of Elixhauser comorbidities (e.g., alcohol abuse, blood loss anemia, cardiac arrhythmias, chronic pulmonary disease …) are presented in supplement S9

For peer review only - http://bmjopen.bmj.com/site/about/guidelines.xhtml

Table 3 – Multivariate analysis of patient safety outcomes in 20,395 rectum resections in 200 hospitals

	IN-HOSPITAL DEATH		POST-OPERATIVE RESPIRATORY FAILURE		RENAL FAILURE		POST-OPERATIVE WOUND INFECTION	
	OR	95% CI	OR	95% CI	OR	95% CI	OR	95% CI
**HEALTHCARE COVARIATES**								
admission reason								
referral	Ref.		Ref.		Ref.		Ref.	
emergency case	2.028***	(1.675 - 2.454)	1.335***	(1.170 - 1.523)	1.342***	(1.169 - 1.540)	1.291***	(1.138 - 1.466)
transfer from other hospital	2.679***	(1.874 - 3.828)	1.859***	(1.406 - 2.459)	1.927***	(1.461 - 2.541)	1.484**	(1.131 - 1.948)
weekend surgery								
no	Ref.		Ref.		Ref.		Ref.	
yes	1.960***	(1.483 - 2.591)	1.427**	(1.150 - 1.770)	1.391**	(1.127 - 1.717)	0.985	(0.784 - 1.238)
total colon resection								
no	Ref.		Ref.		Ref.		Ref.	
yes	2.579***	(2.163 - 3.074)	2.164***	(1.921 - 2.438)	1.859***	(1.645 - 2.100)	1.522***	(1.356 - 1.708)
**HOSPITAL COVARIATES**								
case volume	0.703***	(0.611 - 0.809)	0.844*	(0.725 - 0.982)	0.853**	(0.760 - 0.958)	0.973	(0.854 - 1.109)
area								
urban	Ref.		Ref.		Ref.		Ref.	
rural	1.072	(0.817 - 1.407)	0.904	(0.639 - 1.281)	0.834	(0.670 - 1.037)	0.854	(0.663 - 1.101)
university hospital								
no	Ref.		Ref.		Ref.		Ref.	
yes	1.616	(0.979 - 2.665)	0.853	(0.379 - 1.920)	1.299	(0.829 - 2.037)	2.292**	(1.358 - 3.869)
ownership								
public	Ref.		Ref.		Ref.		Ref.	
non-profit	0.851	(0.614 - 1.179)	1.059	(0.674 - 1.664)	0.762	(0.576 - 1.008)	0.761	(0.553 - 1.048)
private	0.925	(0.682 - 1.254)	1.267	(0.865 - 1.858)	1.597***	(1.256 - 2.030)	0.846	(0.642 - 1.117)
**CASE COVARIATES**								
sex								
male	Ref.		Ref		Ref		Ref	
female	0.842*	(0.710 - 0.998)	0.842**	(0.759 - 0.934)	0.685***	(0.613 - 0.765)	0.826***	(0.748 - 0.912)
age	1.068***	(1.059 - 1.078)	1.014***	(1.009 - 1.018)	1.021***	(1.016 - 1.026)	0.998	(0.995 - 1.002)
Elixhauser comorbidities […][a]								

Legend:
*** p<0.001
** p <0.01
* p<0.05
[a] results of Elixhauser comorbidities (e.g., alcohol abuse, blood loss anemia, cardiac arrhythmias, chronic pulmonary disease …) are presented in supplement S10

For peer review only - http://bmjopen.bmj.com/site/about/guidelines.xhtml

Discussion

This large cross-sectional analysis of 54,168 colon resections and 20,395 rectum resections presents new and comprehensive findings for patient safety.

Healthcare-level covariates were significant risk factors for multiple patient safety outcomes. Preoperative transfer from other hospitals and emergency admission as possible proxy for case urgency were precursors of poor patient safety outcome in both groups. These findings confirm recent literature reporting associations between emergency admissions or transfers from other hospitals and 30-day-mortality, 5-year survival, complications, length of stay or morbidities.[7-11] Weekend surgery was associated with higher risks for death, post-operative respiratory and renal failure in both groups supported by the literature of mortality in colon[4] and general surgery.[5, 6] Regarding rectum resections, the literature reported insignificant effects. These are most likely explained by a small number of included cases.[4] These findings underline the need for the consideration of healthcare contexts in risk-adjusted quality assurance.

The hospital covariates in this analysis showed conflicting effects. Inconclusive results were found for rural localization, university hospital status and hospital ownership. The estimated effects were insignificant (rural hospitals) or conflicting (volume, ownership) and therefore did not strongly affect the considered patient safety outcomes. The literature discusses the influence of case volume,[12, 13, 17, 28, 29] rural hospitals,[30-32] ownership[29, 33], university hospital status[34] or hospital size in general[29] with confirming or contradicting results often explained by e.g., patient case-mix, staffing or surgeon experience differing between hospital sizes.[29, 35] This may be due to outcome-relevant information like staffing,[36, 37] expertise[38] or certification[39]not being included in claims data. For example a German study reported insignificant associations between ownership and post-operative wound infections after colon surgery.[40] The differences compared to our analyses are the procedure-definitions (partial/ total colon resections vs. open/ laparoscopic colon procedure), the sample size (54,168 colon resections vs. 28,291 colon procedures) and the data. The claims data used in our analysis include individual information on age, sex and comorbidities. Infection surveillance data used by Schröder et al. does not include individual patient data on age, sex or severity of a patient's illness.[40]

Additionally, some studies did not stratify colon and rectum resections.[41, 42] However, the heterogeneous results for both procedure groups indicate the relevance of stratification as already reported for other indications.[16]

With respect to case covariates age, sex and comorbidities like coagulopathies, heart diseases, lung diseases or fluid and electrolyte disorders were risk factors for poor patient outcome in both groups in this analysis, which is supported by the literature as well.[26, 43-48]

For peer review only - http://bmjopen.bmj.com/site/about/guidelines.xhtml

There are several strengths to this study. This study analyzed a large and current sample providing a broad span of cases, hospital types, ownerships, and locations. While previous studies emphasized specific covariates and/or outcomes, we considered combined sets of previously solitarily analyzed outcomes and risk factors and, thus, provide a comprehensive analysis. The applied multilevel-regression model is able to simultaneously analyze individual covariates like comorbidities and hospital level covariates like annual case volume. It also considers relationships between covariates (e.g., weekend surgery and emergency admissions).[25]

There are several limitations to this study. Secondary data induce challenges for a reliable operationalization of outcomes. First, the data are anonymized. The anonymization makes it impossible to validate the coded diagnoses.[49] Secondly, claims data do not include information which of the coded diagnoses had been present on admission. To overcome these shortcomings, this study used a set of previously validated outcomes that were reported to occur most likely during hospitalization.[23] With respect to transfer from other hospitals, recent literature distinguishes between urgent and non-urgent inter-hospital transfers.[9-11] The data included in this analysis does not include details on the reasons for transfer from other hospital. However, our results were adjusted for age, sex, comorbidities and weekend surgery representing severity and complexity. The different results depending on adjustment, stratification, bi- and multivariate analyses underline the need for careful and comprehensive statistical analysis. One weakness of German hospital discharge data is a lack of information on patient history, medication, length of anesthesia, staff-to-patient ratios, surgeon volumes, acuity/ reasons for inter-hospital transfers, validity of coding, centralization and comorbidities present on admission.[37, 39, 50] This lacking information may lead to bias as these covariates may influence the outcome and could not be considered in our study. To overcome these challenges we sought to define colon and rectum resections,[22] outcomes[23] and comorbidities[20] based on study literature for transparency and consistency. The advantage of this process has its limits. These definitions do not involve specific distinctions referring to procedure (e.g., type, localization) or comorbidities (e.g., bowel disease). To ensure transparency we decided against creating our own definitions of procedures or comorbidities. An additional limitation is the limited possibility to analyze some specific subgroups (e.g., case volume stratified by ownership, weekend surgeries stratified by admission) in models using a large set of covariates. It poses the risk of separation due alone to the small sample size of specific subgroup-populations and outcomes.[51]

For peer review only - http://bmjopen.bmj.com/site/about/guidelines.xhtml

Conclusions

This study demonstrated that patient safety in colorectal resections is strongly related to specific healthcare covariates. Our results implicate a need to account for admission reasons and weekend surgery when measuring and comparing patient safety. Therefore a risk adjustment for these covariates in quality assurance measures should be pursued. Hospital volume, ownership, urbanization degree and university hospital status could not be shown to be strongly associated with all patient safety outcomes of colorectal resections. Given these insights from an analysis of a large dataset, this paper contributes reliable and comprehensive evidence to the ongoing debate on hospital- and healthcare-related influences on patient safety in general.

For peer review only

For peer review only - http://bmjopen.bmj.com/site/about/guidelines.xhtml

Funding: The IMPRESS study is funded by the Innovation Fund of the Joint Federal Committee (Gemeinsamer Bundesausschuss, G-BA), Germany. Funding number: 01VSF16013. Open Access Funding by the Publication Fund of the TU Dresden.

Author contributions:

FW designed the concept, methods and investigation, visualized results and wrote the draft of the underlying analysis. MR undertook the formal analysis. MR and OS curated the data, supervised the methodology and project administration in general and revised the drafts. JS, MEG, PS and RK acquired funding, supervised the concept and investigation and revised the drafts.

Competing interests: Peter C. Scriba and Ralf Kuhlen are members of the scientific advisory board of IQM. Maria Eberlein-Gonska serves as an external expert for IQM. The other authors declare that they have no conflict of interest.

Data availability statement: The data are not publicly available due containing information that could compromise research participant privacy/consent. The consent given from the research participants included the obligation to analyze the data anonymously without disclosure to other parties.

For peer review only

References

1 O'Brien BS, McNally MP, Duncan JE. Controversies surrounding quality measurement in colon and rectal surgery. *Clin Colon Rectal Surg* 2014;27(1):26-31. doi: 10.1055/s-0034-1366916 [published Online First: 2014/03/04]

2 Goodacre S, Campbell M, Carter A. What do hospital mortality rates tell us about quality of care? *Emerg Med J* 2015;32(3):244-7. doi: 10.1136/emermed-2013-203022 [published Online First: 2013/09/26]

3 Lilford R, Pronovost P. Using hospital mortality rates to judge hospital performance: a bad idea that just won't go away. *Bmj* 2010;340:c2016. doi: 10.1136/bmj.c2016 [published Online First: 2010/04/22]

4 Huijts DD, van Groningen JT, Guicherit OR, et al. Weekend Effect in Emergency Colon and Rectal Cancer Surgery: A Prospective Study Using Data From the Dutch ColoRectal Audit. *J Natl Compr Canc Netw* 2018;16(6):735-41. doi: 10.6004/jnccn.2018.7016 [published Online First: 2018/06/13]

5 McCallum IJ, McLean RC, Dixon S, et al. Retrospective analysis of 30-day mortality for emergency general surgery admissions evaluating the weekend effect. *Br J Surg* 2016;103(11):1557-65. doi: 10.1002/bjs.10261 [published Online First: 2016/08/16]

6 O'Leary JD, Wunsch H, Leo AM, et al. Hospital admission on weekends for patients who have surgery and 30-day mortality in Ontario, Canada: A matched cohort study. *PLoS Med* 2019;16(1):e1002731. doi: 10.1371/journal.pmed.1002731 [published Online First: 2019/01/30]

7 Anderson JH, Hole D, McArdle CS. Elective versus emergency surgery for patients with colorectal cancer. *Br J Surg* 1992;79(7):706-9. doi: 10.1002/bjs.1800790739 [published Online First: 1992/07/01]

8 Mullen MG, Michaels AD, Mehaffey JH, et al. Risk Associated With Complications and Mortality After Urgent Surgery vs Elective and Emergency Surgery: Implications for Defining "Quality" and Reporting Outcomes for Urgent Surgery. *JAMA Surgery* 2017;152(8):768-74. doi: 10.1001/jamasurg.2017.0918

9 Chow CJ, Gaertner WB, Jensen CC, et al. Does Hospital Transfer Impact Outcomes After Colorectal Surgery? *Diseases of the Colon & Rectum* 2017;60(2):194-201. doi: 10.1097/dcr.0000000000000765

10 Sharp SP, Ata A, Valerian BT, et al. Complications and surgical outcomes after interhospital transfer vs direct admission in colorectal surgery: A National Surgical Quality Improvement Program analysis. *Am J Surg* 2017;213(6):1031-37. doi: 10.1016/j.amjsurg.2016.08.013 [published Online First: 2016/10/25]

For peer review only - http://bmjopen.bmj.com/site/about/guidelines.xhtml

11 Sharp SP, Schuster DJ, Ata A, et al. Impact of Interhospital Transfer on Outcomes in Non-emergency Colorectal Surgery. *World J Surg* 2018;42(5):1542-50. doi: 10.1007/s00268-017-4313-z [published Online First: 2017/10/29]

12 Chioreso C, Del Vecchio N, Schweizer ML, et al. Association Between Hospital and Surgeon Volume and Rectal Cancer Surgery Outcomes in Patients With Rectal Cancer Treated Since 2000: Systematic Literature Review and Meta-analysis. *Dis Colon Rectum* 2018;61(11):1320-32. doi: 10.1097/dcr.0000000000001198 [published Online First: 2018/10/05]

13 Huo YR, Phan K, Morris DL, et al. Systematic review and a meta-analysis of hospital and surgeon volume/outcome relationships in colorectal cancer surgery. *J Gastrointest Oncol* 2017;8(3):534-46. doi: 10.21037/jgo.2017.01.25 [published Online First: 2017/07/25]

14 Almoudaris AM, Burns EM, Bottle A, et al. Single measures of performance do not reflect overall institutional quality in colorectal cancer surgery. *Gut* 2013;62(3):423. doi: 10.1136/gutjnl-2011-301489

15 Schmitt J, Schoffer O, Walther F, et al. Effectiveness of the IQM peer review procedure to improve in-patient care—a pragmatic cluster randomized controlled trial (IMPRESS): study design and baseline results. *Journal of Public Health* 2019 doi: 10.1007/s10389-019-01118-9

16 Schoffer O, Roessler M, Walther F, et al. Patient-Level and Hospital-Level Risk Factors for In-Hospital Mortality in Patients Ventilated for More Than 24 Hours: Results of a Nationwide Cohort Study. *Journal of Intensive Care Medicine* 2020;0(0):0885066620942182. doi: 10.1177/0885066620942182

17 Roessler M, Walther F, Eberlein-Gonska M, et al. Exploring relationships between in-hospital mortality and hospital case volume using random forest: results of a cohort study based on a nationwide sample of German hospitals, 2016-2018. *BMC Health Serv Res* 2022;22(1):1. doi: 10.1186/s12913-021-07414-z [published Online First: 2022/01/04]

18 Schmitt J, Roessler M, Scriba P, et al. Effect of clinical peer review on mortality in patients ventilated for more than 24 hours: a cluster randomised controlled trial. *BMJ Qual Saf* 2022 doi: 10.1136/bmjqs-2021-013864 [published Online First: 2022/04/07]

19 Walther F, Schoffer O, Schmitt J. Effectiveness of a collegial consultation procedure to improve in-patient care—a pragmatic cluster randomized controlled trial (ISRCTN10188560): ISRCTN; 2018 [Available from: http://www.isrctn.com/ISRCTN10188560 accessed 01/09/ 2020.

For peer review only - http://bmjopen.bmj.com/site/about/guidelines.xhtml

20 Elixhauser A, Steiner C, Harris DR, et al. Comorbidity measures for use with administrative data. *Med Care* 1998;36(1):8-27. doi: 10.1097/00005650-199801000-00004 [published Online First: 1998/02/07]

21 Quan H, Sundararajan V, Halfon P, et al. Coding algorithms for defining comorbidities in ICD-9-CM and ICD-10 administrative data. *Med Care* 2005;43(11):1130-9. doi: 10.1097/01.mlr.0000182534.19832.83 [published Online First: 2005/10/15]

22 Mansky T, Nimptsch U, Cools A, et al. G-IQI – German Inpatient Quality Indicators. Version 5.1 – Band 1. Berlin: Universitätsverlag der TU Berlin 2017.

23 Maass C, Kuske S, Lessing C, et al. Are administrative data valid when measuring patient safety in hospitals? A comparison of data collection methods using a chart review and administrative data. *Int J Qual Health Care* 2015;27(4):305-13. doi: 10.1093/intqhc/mzv045 [published Online First: 2015/07/03]

24 Hentschker C, Mennicken R. The Volume-Outcome Relationship Revisited: Practice Indeed Makes Perfect. *Health Serv Res* 2018;53(1):15-34. doi: 10.1111/1475-6773.12696 [published Online First: 2017/09/05]

25 Snijders TAB, Bosker RJ. Multilevel analysis : an introduction to basic and advanced multilevel modeling. 2nd ed. ed: Los Angeles ; London : SAGE 2012.

26 Byrne BE, Mamidanna R, Vincent CA, et al. Population-based cohort study comparing 30- and 90-day institutional mortality rates after colorectal surgery. *Br J Surg* 2013;100(13):1810-7. doi: 10.1002/bjs.9318 [published Online First: 2013/11/15]

27 Sheikh L, Croft R, Harmston C. Counting the costs of complications in colorectal surgery. *N Z Med J* 2019;132(1497):32-36. [published Online First: 2019/06/21]

28 Link KH, Coy P, Roitman M, et al. Minimum Volume Discussion in the Treatment of Colon and Rectal Cancer: A Review of the Current Status and Relevance of Surgeon and Hospital Volume regarding Result Quality and the Impact on Health Economics. *Visceral Medicine* 2017;33(2):140-47. doi: 10.1159/000456044

29 Malheiro R, Peleteiro B, Correia S. Beyond the operating room: do hospital characteristics have an impact on surgical site infections after colorectal surgery? A systematic review. *Antimicrob Resist Infect Control* 2021;10(1):139. doi: 10.1186/s13756-021-01007-5 [published Online First: 2021/10/02]

For peer review only

30 Hamidi M, Hanna K, Omesiete P, et al. Does it matter where you get your surgery for colorectal cancer? *International Journal of Colorectal Disease* 2019;34(12):2121-27. doi: 10.1007/s00384-019-03436-6

31 Knight M. The Effect of Hospital Organizational Characteristics on Postoperative Complications. *Journal of Patient Safety* 2013;9(4):198-202. doi: 10.1097/PTS.0b013e3182995e5b

32 Pandit V, Jehan F, Zeeshan M, et al. Failure to Rescue in Postoperative Patients With Colon Cancer: Time to Rethink Where You Get Surgery. *Journal of Surgical Research* 2019;234:1-6. doi: https://doi.org/10.1016/j.jss.2018.08.046

33 Morris M, Iacopetta B, Platell C. Comparing survival outcomes for patients with colorectal cancer treated in public and private hospitals. *Med J Aust* 2007;186(6):296-300. [published Online First: 2007/03/21]

34 Juillard C, Lashoher A, Sewell CA, et al. A National Analysis of the Relationship Between Hospital Volume, Academic Center Status, and Surgical Outcomes for Abdominal Hysterectomy Done for Leiomyoma. *Journal of the American College of Surgeons* 2009;208(4):599-606. doi: https://doi.org/10.1016/j.jamcollsurg.2009.01.003

35 Tserenpuntsag B, Haley V, Van Antwerpen C, et al. Surgical Site Infection Risk Factors Identified for Patients Undergoing Colon Procedures, New York State 2009–2010. *Infection Control & Hospital Epidemiology* 2014;35(8):1006-12. doi: 10.1086/677156 [published Online First: 2016/05/10]

36 Etzioni DA, Young-Fadok TM, Cima RR, et al. Patient survival after surgical treatment of rectal cancer: impact of surgeon and hospital characteristics. *Cancer* 2014;120(16):2472-81. doi: 10.1002/cncr.28746 [published Online First: 2014/05/08]

37 Yasunaga H, Hashimoto H, Horiguchi H, et al. Variation in cancer surgical outcomes associated with physician and nurse staffing: a retrospective observational study using the Japanese Diagnosis Procedure Combination Database. *BMC Health Services Research* 2012;12(1):129. doi: 10.1186/1472-6963-12-129

38 Hall GM, Shanmugan S, Bleier JI, et al. Colorectal specialization and survival in colorectal cancer. *Colorectal Dis* 2016;18(2):O51-60. doi: 10.1111/codi.13246 [published Online First: 2015/12/29]

39 Trautmann F, Reißfelder C, Pecqueux M, et al. Evidence-based quality standards improve prognosis in colon cancer care. *European Journal of Surgical Oncology* 2018;44(9):1324-30. doi: https://doi.org/10.1016/j.ejso.2018.05.013

40 Schröder C, Behnke M, Geffers C, et al. Hospital ownership: a risk factor for nosocomial infection rates? *Journal of Hospital Infection* 2018;100(1):76-82. doi: https://doi.org/10.1016/j.jhin.2018.01.019

For peer review only - http://bmjopen.bmj.com/site/about/guidelines.xhtml

41 Kolfschoten NE, Marang-van de Mheen PJ, Wouters MWJM, et al. A Combined Measure of Procedural Volume and Outcome to Assess Hospital Quality of Colorectal Cancer Surgery, a Secondary Analysis of Clinical Audit Data. *PLOS ONE* 2014;9(2):e88737. doi: 10.1371/journal.pone.0088737

42 Liu CJ, Chou YJ, Teng CJ, et al. Association of surgeon volume and hospital volume with the outcome of patients receiving definitive surgery for colorectal cancer: A nationwide population-based study. *Cancer* 2015;121(16):2782-90. doi: 10.1002/cncr.29356 [published Online First: 2015/04/22]

43 Mamidanna R, Burns EM, Bottle A, et al. Reduced Risk of Medical Morbidity and Mortality in Patients Selected for Laparoscopic Colorectal Resection in England: A Population-Based Study. *Archives of Surgery* 2012;147(3):219-27. doi: 10.1001/archsurg.2011.311

44 Marietta M, Facchini L, Pedrazzi P, et al. Pathophysiology of Bleeding in Surgery. *Transplantation Proceedings* 2006;38(3):812-14. doi: https://doi.org/10.1016/j.transproceed.2006.01.047

45 Miller TE, Mythen M, Shaw AD, et al. Association between perioperative fluid management and patient outcomes: a multicentre retrospective study. *Br J Anaesth* 2021;126(3):720-29. doi: 10.1016/j.bja.2020.10.031 [published Online First: 2020/12/16]

46 Flynn DE, Mao D, Yerkovich ST, et al. The impact of comorbidities on post-operative complications following colorectal cancer surgery. *PLOS ONE* 2020;15(12):e0243995. doi: 10.1371/journal.pone.0243995

47 Inokuchi M, Kato K, Sugita H, et al. Impact of comorbidities on postoperative complications in patients undergoing laparoscopy-assisted gastrectomy for gastric cancer. *BMC Surgery* 2014;14(1):97. doi: 10.1186/1471-2482-14-97

48 Chang H-R, Shih S-C, Lin F-M. Impact of Comorbidities on the Outcomes of Older Patients Receiving Rectal Cancer Surgery. *International Journal of Gerontology* 2012;6(4):285-89. doi: https://doi.org/10.1016/j.ijge.2012.05.006

49 Ghadban T, Reeh M, Bockhorn M, et al. Decentralized colorectal cancer care in Germany over the last decade is associated with high in-hospital morbidity and mortality. *Cancer Manag Res* 2019;11:2101-07. doi: 10.2147/cmar.S197865 [published Online First: 2019/03/19]

50 Renzulli P, Lowy A, Maibach R, et al. The influence of the surgeon's and the hospital's caseload on survival and local recurrence after colorectal cancer surgery. *Surgery* 2006;139(3):296-304. doi: https://doi.org/10.1016/j.surg.2005.08.023

51 Mansournia MA, Geroldinger A, Greenland S, et al. Separation in Logistic Regression: Causes, Consequences, and Control. *American Journal of Epidemiology* 2017;187(4):864-70. doi: 10.1093/aje/kwx299

For peer review only - http://bmjopen.bmj.com/site/about/guidelines.xhtml

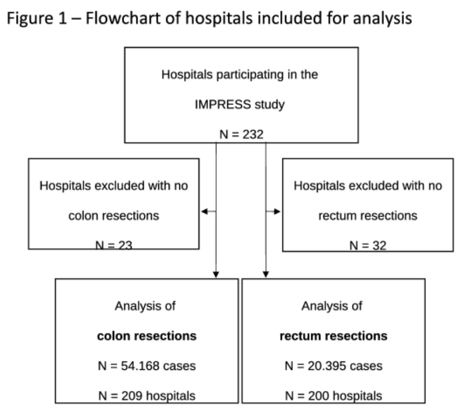

Figure 1 - Flowchart of hospitals included for analysis

338x190mm (600 x 600 DPI)

S1: ICD-10 Codes of Elixhauser Comorbidity Groups [20]

Congestive heart failure: I099, I110, I130, I132, I255, I420, I425, I426, I427, I428, I429, I43, I50, P290

Cardiac arrhythmias: I441, I442, I443, I456, I459, I47, I48, I49, R000, R001, R008, T821, Z450, Z950

Valvular disease : A520, I05, I06, I07, I08, I091, I098, I34, I35, I36, I37, I38, I39, Q230, Q231, Q232, Q233, Z952, Z953 Z954

Pulmonary circulation Disorders: I26, I27, I280, I288, I289

Peripheral vascular disorders: I70, I71, I731, I738, I739, I771, I790, I792, K551, K558, K559, Z958, Z959

Hypertension, uncomplicated: I10

Hypertension, complicated: I11, I12, I13, I15

Paralysis: G041, G114, G801, G802, G81, G82, G830, G831, G832, G833, G834, G839

Other neurological disorders: G10, G11, G12, G13, G20, G21, G22, G254, G255, G312, G318, G319, G32, G35, G36, G37, G40, G41, G931, G934, R470, R56

Chronic pulmonary disease: I278, I279, J40, J41, J42, J43, J44, J45, J46, J47, J60, J61, J62, J63, J64, J65, J66, J67, J684, J701, J703

Diabetes, uncomplicated: E100, E101, E109, E110, E111, E119, E120, E121, E129, E130, E131, E139, E140, E141, E149

Diabetes, complicated: E102, E103, E104, E105, E106, E107, E108, E112, E113, E114, E115, E116, E117, E118, E122, E123, E124, E125, E126, E127, E128, E132, E133, E134, E135, E136, E137, E138, E142, E143, E144, E145, E146, E147, E148

Hypothyroidism: E00, E01, E02, E03, E890

Renal failure: I120, I131, N18, N19, N250, Z490, Z491, Z492, Z940, Z992

Peptic ulcer disease excluding bleeding: K257, K259, K267, K269, K277, K279, K287, K289

AIDS/HIV: B20, B21, B22, B24

Lymphoma: C81, C82, C83, C84, C85, C88, C96, C900, C902

Metastatic cancer: C77, C78, C79, C80

Solid tumor without metastasis: C00, C01, C02, C03, C04, C05, C06, C07, C08, C09, C1 0, C11, C12, C13, C14, C15, C16, C17, C18, C19, C20, C21, C22, C23, C24, C25, C26, C30, C31, C32, C33, C34, C37, C38, C39, C40, C41, C43, C45, C46, C47, C48, C49, C50, C51, C52, C53, C54, C55, C56, C57, C58, C60, C61, C62, C63, C64, C65, C66, C67, C68, C69, C70, C71, C72, C73, C74, C75, C76, C97

Rheumatoid arthritis/collagen vascular diseases: L940, L941, L943, M05, M06, M08, M120, M123, M30, M310, M311, M312, M313, M32, M33, M34, M35, M45, M461, M468, M469

Coagulopathy: D65, D66, D67, D68, D691, D693, D694, D695, D696

Obesity: E66

Weight loss: E40, E41, E42, E43, E44, E45, E46, R634, R64

Fluid and electrolyte disorders: E222, E86, E87

Blood loss anemia: D500

For peer review only

S2: OPS-codes for partial colon resections according to GIQI [21]

OPS-CODE	TRANSLATION (BY AUTHOR)
5-455.01	Partial resection of the colon: segmental resection: open surgery with anastomosis
5-455.02	Partial resection of the colon: segmental resection: open surgery with enterostoma and blind closure
5-455.03	Partial Resection of the Colon: Segment Resection: Open Surgery with Two Enterostomata
5-455.04	Partial Resection of the Colon: Segment Resection: Open Surgery with Anastomosis Anus praeter
5-455.05	Partial Resection of the Colon: Segmental Resection: Laparoscopic with Anastomosis
5-455.06	Partial Resection of the Colon: Segmental Resection: Laparoscopic with Enterostomy
5-455.07	Partial resection of the colon: segmental resection: change laparoscopic - open surgical
5-455.0X	Partial resection of the colon: Segment resection: Other
5-455.11	Partial resection of the colon: multiple segment resections: Open surgery with anastomosis
5-455.12	Partial resection of the colon: multiple segment resections: Open surgery with enterostoma and blind closure
5-455.13	Partial resection of the colon: multiple segment resections: Open surgery with two enterostomata
5-455.14	Partial resection of the colon: multiple segment resections: Open surgery with anastomosis anus praeter
5-455.15	Partial resection of the colon: multiple segment resections: Laparoscopic with anastomosis
5-455.16	Partial resection of the colon: multiple segment resections: Laparoscopic with enterostomy
5-455.17	Partial resection of the colon: multiple segment resections: Transfer laparoscopic - open surgery
5-455.1X	Partial resection of the colon: multiple segment resections: Other
5-455.21	Partial Resection of the Colon: Ileocecal Resection: Open Surgery with Anastomosis
5-455.22	Partial Resection of the Colon: Ileocecal Resection: Open surgery with enterostoma and blind closure
5-455.23	Partial Resection of the Colon: Ileocecal Resection: Open Surgery with Two Enterostomata
5-455.24	Partial Resection of the Colon: Ileocecal Resection: Open Surgery with Anastomosis Anus praeter
5-455.25	Partial Resection of the Colon: Ileocecal Resection: Laparoscopic with Anastomosis
5-455.26	Partial Resection of the Colon: Ileocecal Resection: Laparoscopic with Enterostoma
5-455.27	Partial resection of the colon: ileocecal resection: change laparoscopic - open surgical
5-455.2X	Partial resection of the colon: Ileocecal resection: Other
5-455.31	Partial resection of the colon: Caecal resection: Open surgery
5-455.35	Partial resection of the colon: Caecal resection: Laparoscopic
5-455.37	Partial resection of the colon: Caecal resection: change laparoscopic - open surgical
5-455.41	Partial resection of the colon: Resection of the ascending colon with coecum and right flexure [hemicolectomy right]: Open surgery with anastomosis
5-455.42	Partial Resection of the Colon: Resection of the ascending colon with coecum and right flexure [Hemicolectomy right]: Open surgery with enterostoma and
5-455.43	Partial Resection of the Colon: Resection of the ascending colon with coecum and right flexure [Hemicolectomy right]: Open surgery with two enterostomata
5-455.44	Partial resection of the colon: Resection of the ascending colon with coecum and right flexure [hemicolectomy right]: Open surgery with anastomosis anus praeter
5-455.45	Partial resection of the colon: Resection of the ascending colon with coecum and right flexure [hemicolectomy right]: Laparoscopic with anastomosis
5-455.46	Partial resection of the colon: Resection of the ascending colon with coecum and right flexure [hemicolectomy right]: Laparoscopically with enterostoma
5-455.47	Partial resection of the colon: resection of the ascending colon with coecum and right flexure [hemicolectomy right]: change laparoscopic - open surgical
5-455.4X	Partial resection of the colon: Resection of the ascending colon with coecum and right flexure [right hemicolectomy]: Other
5-455.51	Partial resection of the colon: Resection of the transverse colon: Open surgery with anastomosis
5-455.52	Partial resection of the colon: Resection of the transverse colon: open surgery with enterostoma and blind closure
5-455.53	Partial Resection of the Colon: Resection of the Transverse Colon: Open Surgery with Two Enterostomata
5-455.54	Partial resection of the colon: resection of the transverse colon: open surgery with anastomosis anus praeter
5-455.55	Partial resection of the colon: Resection of the transverse colon: Laparoscopic with anastomosis
5-455.56	Partial Resection of the Colon: Resection of the Transverse Colon: Laparoscopic with Enterostoma
5-455.57	Partial resection of the colon: resection of the transverse colon: change laparoscopic - open surgical
5-455.5X	Partial resection of the colon: Resection of the transverse colon: Other
5-455.61	Partial resection of the colon: resection of the descending colon with left flexure [hemicolectomy left]: open surgery with anastomosis
5-455.62	Partial resection of the colon: resection of the descending colon with left flexure [hemicolectomy left]: open surgery with enterostoma and blind closure
5-455.63	Partial resection of the colon: resection of the descending colon with left flexure [hemicolectomy left]: open surgery with two enterostomata
5-455.64	Partial resection of the colon: resection of the descending colon with left flexure [hemicolectomy left]: open surgery with anastomosis anus praeter
5-455.65	Partial resection of the colon: Resection of the descending colon with left flexure [Hemicolectomy left]: Laparoscopic with anastomosis
5-455.66	Partial Resection of the Colon: Resection of the descending colon with left flexure [Hemicolectomy left]: Laparoscopic with Enterostoma
5-455.67	Partial resection of the colon: resection of the descending colon with left flexure [hemicolectomy left]: change laparoscopic - open surgical
5-455.6X	Partial resection of the colon: Resection of the descending colon with left flexure [Hemicolectomy left]: Other
5-455.71	Partial resection of the colon: sigmoid resection: open surgery with anastomosis
5-455.72	Partial resection of the colon: sigmoid resection: open surgery with enterostoma and blind closure
5-455.73	Partial resection of the colon: sigmoid resection: open surgery with two enterostomata
5-455.74	Partial resection of the colon: sigmoid resection: open surgery with anastomosis anus praeter

For peer review only - http://bmjopen.bmj.com/site/about/guidelines.xhtml

5-455.75	Partial resection of the colon: sigmoid resection: laparoscopic with anastomosis
5-455.76	Partial resection of the colon: Sigmoid resection: laparoscopic with enterostoma
5-455.77	Partial resection of the colon: sigmoid resection: change laparoscopic - open surgical
5-455.7X	Partial resection of the colon: Sigmoid resection: Other
5-455.91	Partial resection of the colon: Resection of the ascending colon with coecum and right flexure and transverse colon [right hemicolectomy with transverse resection]:
5-455.92	Partial resection of the colon: Resection of the ascending colon with coecum and right flexure and transverse colon [right hemicolectomy with transverse resection]:
5-455.93	Partial resection of the colon: Resection of the ascending colon with coecum and right flexure and transverse colon [right hemicolectomy with transverse resection]:
5-455.94	Partial resection of the colon: Resection of the ascending colon with coecum and right flexure and transverse colon [right hemicolectomy with transverse resection]:
5-455.95	Partial resection of the colon: Resection of the ascending colon with coecum and right flexure and transverse colon [right hemicolectomy with transverse resection]:
5-455.96	Partial resection of the colon: Resection of the ascending colon with coecum and right flexure and transverse colon [right hemicolectomy with transverse resection]:
5-455.97	Partial resection of the colon: Resection of the ascending colon with coecum and right flexure and transverse colon [right hemicolectomy with transverse resection]:
5-455.9X	Partial resection of the colon: Resection of the ascending colon with coecum and right flexure and transverse colon [right hemicolectomy with transverse resection]:
5-455.A1	Partial resection of the colon: Resection of the descending colon with left flexure and transverse colon [left hemicolectomy with transverse resection]: Open
5-455.A2	Partial resection of the colon: Resection of the descending colon with left flexure and transverse colon [left hemicolectomy with transverse resection]: Open
5-455.A3	Partial resection of the colon: Resection of the descending colon with left flexure and transverse colon [left hemicolectomy with transverse resection]: Open
5-455.A4	Partial resection of the colon: Resection of the descending colon with left flexure and transverse colon [left hemicolectomy with transverse resection]: Open
5-455.A5	Partial Resection of the Colon: Resection of the descending colon with left flexure and transverse colon [Hemicolectomy left with transverse resection]: Laparoscopic
5-455.A6	Partial Resection of the Colon: Resection of the descending colon with left flexure and transverse colon [Hemicolectomy left with transverse resection]: Laparoscopic
5-455.A7	Partial resection of the colon: resection of the descending colon with left flexure and transverse colon [hemicolectomy left with transverse resection]: change
5-455.AX	Partial resection of the colon: Resection of the descending colon with left flexure and transverse colon [left hemicolectomy with transverse resection]: Other
5-455.B1	Partial resection of the colon: resection of the descending colon and sigmoid colon: open surgery with anastomosis
5-455.B2	Partial resection of the colon: resection of the descending colon and sigmoid colon: open surgery with enterostoma and blind closure
5-455.B3	Partial Resection of the Colon: Resection of the descending colon and sigmoid colon: Open surgery with two enterostomata
5-455.B4	Partial Resection of the Colon: Resection of the descending colon and sigmoid colon: Open surgery with anastomosis anus praeter
5-455.B5	Partial resection of the colon: resection of the descending colon and sigmoid colon: laparoscopic with anastomosis
5-455.B6	Partial resection of the colon: resection of the descending colon and sigmoid colon: laparoscopic with enterostoma
5-455.B7	Partial Resection of the Colon: Resection of the descending colon and sigmoid colon: transfer laparoscopically - open surgery
5-455.BX	Partial resection of the colon: Resection of the descending colon and sigmoid colon: Other
5-455.C1	Partial resection of the colon: resection of the ascending, transverse and descending colon with coecum and right and left flexure [hemicolectomy right and left
5-455.C2	Partial resection of the colon: resection of the ascending, transverse and descending colon with coecum and right and left flexure [hemicolectomy right and left
5-455.C3	Partial resection of the colon: resection of the ascending, transverse and descending colon with coecum and right and left flexure [hemicolectomy right and left
5-455.C4	Partial resection of the colon: resection of the ascending, transverse and descending colon with coecum and right and left flexure [hemicolectomy right and left
5-455.C5	Partial resection of the colon: resection of the ascending, transverse and descending colon with coecum and right and left flexure [hemicolectomy right and left
5-455.C6	Partial resection of the colon: resection of the ascending, transverse and descending colon with coecum and right and left flexure [hemicolectomy right and left
5-455.C7	Partial resection of the colon: resection of the ascending, transverse and descending colon with coecum and right and left flexure [hemicolectomy right and left
5-455.CX	Partial resection of the colon: Resection of the ascending, transverse and descending colon with coecum and right and left flexure [hemicolectomy right and left
5-455.D1	Partial resection of the colon: resection of the transverse colon, descending colon with left flexure and sigmoid colon [hemicolectomy left with
5-455.D2	Partial resection of the colon: resection of the transverse colon, descending colon with left flexure and sigmoid colon [hemicolectomy left with
5-455.D3	Partial resection of the colon: resection of the transverse colon, descending colon with left flexure and sigmoid colon [hemicolectomy left with
5-455.D4	Partial resection of the colon: resection of the transverse colon, descending colon with left flexure and sigmoid colon [hemicolectomy left with
5-455.D5	Partial resection of the colon: resection of the transverse colon, descending colon with left flexure and sigmoid colon [hemicolectomy left with
5-455.D6	Partial resection of the colon: resection of the transverse colon, descending colon with left flexure and sigmoid colon [hemicolectomy left with
5-455.D7	Partial resection of the colon: resection of the transverse colon, descending colon with left flexure and sigmoid colon [hemicolectomy left with
5-455.DX	Partial resection of the colon: resection of the transverse colon, descending colon with left flexure and sigmoid colon [left hemicolectomy with
5-455.X1	Partial resection of the colon: Other: Open surgery with anastomosis
5-455.X2	Partial resection of the colon: Other: Open surgery with enterostoma and blind closure
5-455.X3	Partial resection of the colon: Other: Open surgery with two enterostomata
5-455.X4	Partial resection of the colon: Other: Open surgery with anastomosis anus praeter
5-455.X5	Partial resection of the colon: Other: Laparoscopic with anastomosis
5-455.X6	Partial resection of the colon: Other: Laparoscopic with enterostoma
5-455.X7	Partial resection of the colon: Other: transfer laparoscopic - open surgical
5-455.XX	Partial resection of the colon: Other: Other
5-455.Y	Partial resection of the colon: N.n.ref.

S3: OPS-codes for total colon resections according to GIQI [21]

OPS-CODE	TRANSLATION (BY AUTHOR)
5-456.00	(Total) Colectomy and Proctocolectomy: Colectomy: Open surgery with ileostoma
5-456.01	(Total) Colectomy and Proctocolectomy: Colectomy: Open surgery with ileorectal anastomosis with reservoir (pouch)
5-456.02	(Total) Colectomy and Proctocolectomy: Colectomy: Open surgery with ileorectal anastomosis without reservoir (pouch)
5-456.03	(Total) Colectomy and Proctocolectomy: Colectomy: Open surgery with ileoanal anastomosis with reservoir (pouch)
5-456.04	(Total) Colectomy and Proctocolectomy: Colectomy: Open surgery with ileoanal anastomosis without reservoir (pouch)
5-456.05	(Total) Colectomy and Proctocolectomy: Colectomy: Laparoscopic with reservoir anastomosis (Pouch)
5-456.06	(Total) Colectomy and Proctocolectomy: Colectomy: Laparoscopic with Anastomosis without reservoir (Pouch)
5-456.07	(Total) Colectomy and Proctocolectomy: Colectomy: Laparoscopic with Ileostoma
5-456.08	(Total) Colectomy and Proctocolectomy: Colectomy: change laparoscopic - open surgical
5-456.0X	(Total) Colectomy and Proctocolectomy: Colectomy: Other
5-456.10	(Total) Colectomy and Proctocolectomy: Proctocolectomy: Open surgery with ileostoma
5-456.11	(Total) Colectomy and Proctocolectomy: Proctocolectomy: Open surgery with ileorectal anastomosis with reservoir (pouch)
5-456.12	(Total) Colectomy and Proctocolectomy: Proctocolectomy: Open surgery with ileorectal anastomosis without reservoir (pouch)
5-456.13	(Total) Colectomy and Proctocolectomy: Proctocolectomy: Open surgery with ileoanal anastomosis with reservoir (pouch)
5-456.14	(Total) Colectomy and Proctocolectomy: Proctocolectomy: Open surgery with ileoanal anastomosis without reservoir (pouch)
5-456.15	(Total) colectomy and proctocolectomy: Proctocolectomy: Laparoscopic with reservoir anastomosis (pouch)
5-456.16	(Total) Colectomy and Proctocolectomy: Proctocolectomy: Laparoscopic with anastomosis without reservoir (pouch)
5-456.17	(Total) colectomy and proctocolectomy: proctocolectomy: laparoscopic with ileostomy
5-456.18	(Total) colectomy and proctocolectomy: proctocolectomy: change laparoscopic - open surgical
5-456.1X	(Total) colectomy and proctocolectomy: Proctocolectomy: Other
5-456.20	(Total) colectomy and proctocolectomy: colectomy with proctomucosectomy: open surgery with ileostoma
5-456.21	(Total) colectomy and proctocolectomy: colectomy with proctomucosectomy: open surgery with ileorectal anastomosis with reservoir (pouch)
5-456.22	(Total) Colectomy and Proctocolectomy: Colectomy with Proctomucosectomy: Open surgery with ileorectal anastomosis without reservoir (pouch)
5-456.23	(Total) Colectomy and Proctocolectomy: Colectomy with proctomucosectomy: Open surgery with ileoanal anastomosis with reservoir (pouch)
5-456.24	(Total) colectomy and proctocolectomy: colectomy with proctomucosectomy: open surgery with ileoanal anastomosis without reservoir (pouch)
5-456.25	(Total) colectomy and proctocolectomy: colectomy with proctomucosectomy: laparoscopic with reservoir anastomosis (pouch)
5-456.26	(Total) colectomy and proctocolectomy: colectomy with proctomucosectomy: laparoscopic with anastomosis without reservoir (pouch)
5-456.27	(Total) colectomy and proctocolectomy: colectomy with proctomucosectomy: laparoscopic with ileostomy
5-456.28	(Total) colectomy and proctocolectomy: colectomy with proctomucosectomy: change laparoscopic - open surgical
5-456.2X	(total) colectomy and proctocolectomy: colectomy with proctomucosectomy: other
5-456.X0	(Total) colectomy and proctocolectomy: Other: Open surgery with ileostomy
5-456.X1	(Total) colectomy and proctocolectomy: Other: Open surgery with ileorectal anastomosis with reservoir (pouch)
5-456.X2	(total) colectomy and proctocolectomy: Other: Open surgery with ileorectal anastomosis without reservoir (pouch)
5-456.X3	(Total) colectomy and proctocolectomy: Other: Open surgery with ileoanal anastomosis with reservoir (pouch)
5-456.X4	(Total) colectomy and proctocolectomy: Other: Open surgery with ileoanal anastomosis without reservoir (pouch)
5-456.X5	(total) colectomy and proctocolectomy: Other: Laparoscopic with reservoir anastomosis (pouch)
5-456.X6	(Total) colectomy and proctocolectomy: Other: Laparoscopic with anastomosis without reservoir (pouch)
5-456.X7	(Total) colectomy and proctocolectomy: Other: Laparoscopic with ileostomy
5-456.X8	(total) colectomy and proctocolectomy: Other: change laparoscopic - open surgical
5-456.XX	(Total) colectomy and proctocolectomy: Other: Other
5-456.Y	(Total) colectomy and proctocolectomy: N.n.d.

S4: OPS-codes for rectum resections according to GIQI [21]

OPS-CODE	TRANSLATION (BY AUTHOR)
5-484.01	Rectum resection with sphincter preservation: Anterior cuff resection: Open surgery with anastomosis
5-484.02	Rectum resection with sphincter preservation: Anterior cuff resection: Open surgery with enterostoma and blind closure
5-484.05	Rectum resection with sphincter preservation: Anterior cuff resection: Laparoscopic with anastomosis
5-484.06	Rectum resection with sphincter preservation: Anterior cuff resection: Laparoscopic with enterostomy and blind closure
5-484.08	Rectum resection with sphincter preservation: Anterior cuff resection: change laparoscopic - open surgery with anastomosis
5-484.09	Rectum resection with sphincter preservation: Anterior cuff resection: change laparoscopic - open surgery with enterostoma and blind closure
5-484.0X	Rectum resection with sphincter preservation: Anterior cuff resection: Other
5-484.11	Rectum resection with sphincter preservation: Posterior rectotomy [Rectotomia posterior]: Open surgery with anastomosis
5-484.12	Rectum resection with sphincter preservation: Posterior rectotomy [Rectotomia posterior]: Open surgery with enterostoma and blind closure
5-484.15	Rectum resection with sphincter preservation: Posterior cuff resection [Rectotomia posterior]: Laparoscopic with anastomosis
5-484.16	Rectum resection with sphincter preservation: Posterior cuff resection [Rectotomia posterior]: Laparoscopic with enterostoma and blind closure
5-484.18	Rectum resection with sphincter preservation: posterior cuff resection [Rectotomia posterior]: change laparoscopic - open surgery with anastomosis
5-484.19	Rectum resection with sphincter preservation: posterior cuff resection [rectotomia posterior]: change laparoscopically - open surgically with enterostoma and
5-484.1X	Rectum resection with sphincter preservation: Posterior cuff resection [Rectotomia posterior]: Other
5-484.21	Rectum resection with preservation of sphincter: Tubular resection with paraproctica left intact: Open surgery with anastomosis
5-484.22	Rectum resection with preservation of sphincter: Tubular resection with paraproktium left in place: Open surgery with enterostoma and blind closure
5-484.25	Rectum resection with preservation of sphincter: Tubular resection with paraproctica left in place: Laparoscopic with anastomosis
5-484.26	Rectum resection with preservation of sphincter: Tubular resection with paraproktium: Laparoscopic with enterostoma and blind closure
5-484.27	Rectum resection with sphincter preservation: Tubular resection with paraprotic left in place: Peranal
5-484.28	Rectum resection with sphincter preservation: Tubular resection with paraproctium left in place: change laparoscopic - open surgery with anastomosis
5-484.29	Rectum resection with preservation of sphincter: Tubular resection with preservation of paraproktium: change laparoscopically - open surgically with enterostoma and
5-484.2X	Rectum resection with preservation of sphincter: Tubular resection with preservation of paraproktium: Other
5-484.31	Rectum resection with sphincter preservation: Anterior resection: Open surgery with anastomosis
5-484.32	Rectum resection with sphincter preservation: Anterior resection: open surgery with enterostoma and blind closure
5-484.35	Rectum resection with sphincter preservation: Anterior resection: Laparoscopic with anastomosis
5-484.36	Rectum resection with sphincter preservation: Anterior resection: Laparoscopic with enterostoma and blind closure
5-484.38	Rectum resection with sphincter preservation: Anterior resection: change laparoscopic - open surgical with anastomosis
5-484.39	Rectum resection with sphincter preservation: anterior resection: change laparoscopically - open surgically with enterostoma and blind closure
5-484.3X	Rectum resection with sphincter preservation: Anterior resection: Other
5-484.51	Rectum resection with sphincter preservation: deep anterior resection: open surgery with anastomosis
5-484.52	Rectum resection with sphincter preservation: deep anterior resection: open surgery with enterostoma and blind closure
5-484.55	Rectum resection with sphincter preservation: Deep anterior resection: Laparoscopic with anastomosis
5-484.56	Rectum resection with sphincter preservation: Deep anterior resection: Laparoscopic with enterostoma and blind closure
5-484.58	Rectum resection with sphincter preservation: deep anterior resection: change laparoscopic - open surgery with anastomosis
5-484.59	Rectum resection with sphincter preservation: deep anterior resection: change laparoscopic - open surgical with enterostoma and blind closure
5-484.5X	Rectum resection with sphincter preservation: Deep anterior resection: Other
5-484.61	Rectum resection with sphincter preservation: deep anterior resection with peranal anastomosis: open surgery with anastomosis
5-484.65	Rectum resection with sphincter preservation: deep anterior
5-484.68	Rectum resection with sphincter preservation: deep anterior resection with peranal anastomosis: change laparoscopic - open surgical with anastomosis
5-484.6X	Rectum resection with sphincter preservation: Deep anterior resection with peranal anastomosis: Other
5-484.X1	Rectum resection with sphincter preservation: Other: Open surgery with anastomosis
5-484.X2	Rectum resection with sphincter preservation: Other: Open surgery with enterostoma and blind closure
5-484.X5	Rectum resection with sphincter preservation: Other: Laparoscopic with anastomosis
5-484.X6	Rectum resection with sphincter preservation: Other: Laparoscopic with enterostoma and blind closure

For peer review only - http://bmjopen.bmj.com/site/about/guidelines.xhtml

5-484.X8	Rectum resection with sphincter preservation: Other: change laparoscopic - open surgery with anastomosis
5-484.X9	Rectum resection with sphincter preservation: Other: change laparoscopically - open surgically with enterostoma and blind closure
5-484.XX	Rectum resection with sphincter preservation: Other: Other
5-484.Y	Rectum resection with sphincter preservation: N.n.ref.
5-485.01	Rectum resection without preservation of sphincter: Abdominoperineal: Open surgery
5-485.02	Rectum resection without sphincter preservation: Abdominoperineal: Combined open surgical-laparoscopic
5-485.0X	Rectum resection without sphincter preservation: Abdominoperineal: Other
5-485.1	Rectum resection without preservation of sphincter: Abdominoperineal with removal of adjacent organs
5-485.21	Rectum resection without sphincter preservation: Sacroiliac abdomen: Open surgery
5-485.22	Rectum resection without sphincter preservation: Sacroiliac abdomen: Combined open surgical-laparoscopic
5-485.2X	Rectum resection without sphincter preservation: Sacroiliac abdomen: Other
5-485.3	Rectum resection without sphincter preservation: Sacral abdominosacral with removal of neighboring organs
5-485.4	Rectum resection without sphincter preservation: sacroperineal
5-485.5	Rectum resection without sphincter preservation: Perineal
5-485.X	Rectum resection without sphincter preservation: Other
5-485.Y	Rectum resection without sphincter preservation: N.n.ref.

For peer review only

S5: Definitions of patient safety outcomes

PATIENT SAFETY OUTCOME	DEFINITION ACCORDING TO ICD-10 GM
IN-HOSPITAL DEATH	Discharge destination: death (included in accounting data derived)
POST-OPERATIVE RESPIRATORY FAILURE [15]	ICD-10: J96 .0 - Respiratory failure, not elsewhere classified
POST-OPERATIVE WOUND INFECTION [15]	ICD-10: T81.4 - Infection following a procedure, not elsewhere classified
	ICD-10: T82.6 - Infection and inflammatory reaction due to cardiac valve prosthesis
	ICD-10: T82.7 - Infection and inflammatory reaction due to other cardiac and vascular devices, implants and grafts
	ICD-10: T83.5 - Infection and inflammatory reaction due to prosthetic device, implant and graft in urinary system
	ICD-10: T83.6 - Infection and inflammatory reaction due to prosthetic device, implant and graft in genital tract
	ICD-10: T84.5 - Infection and inflammatory reaction due to internal joint prosthesis
	ICD-10: T84.6 - Infection and inflammatory reaction due to internal fixation device [any site]
	ICD-10: T84.7 - Infection and inflammatory reaction due to other internal orthopaedic prosthetic devices, implants and grafts
	ICD-10: T87.4 - Infection of amputation stump
RENAL FAILURE [15]	ICD-10: N17.0 - Acute renal failure with tubular necrosis
	ICD-10: N17.1 - Acute renal failure with acute cortical necrosis
	ICD-10: N17.2 - Acute renal failure with medullary necrosis
	ICD-10: N17.8 - Other acute renal failure
	ICD-10: N17.9 - Acute renal failure, unspecified
	ICD-10: N99.0- Postprocedural renal failure
	Procedure code: 8-853.7- Continuous, venovenous, pump-driven (CVVH), anticoagulation with heparin or without anticoagulation

For peer review only

S6: Case-, care- and hospital related characteristics

	COLON RESECTIONS		RECTUM RESECTIONS	
**PATIENT SAFETY OUTCOMES**	n	% / Q1; Q3	n	% / Q1; Q3
Outcome in-hospital death				
no	48,914	(90.30 %)	19,525	(95.73 %)
yes	5,254	(9.69 %)	870	(4.26 %)
Outcome post-operative respiratory failure				
no	45,074	(83.21 %)	17,901	(87.77 %)
yes	9,094	(16.78 %)	2,494	(12.22 %)
Outcome post-operative wound infections				
no	48,013	(88.63 %)	18,109	(88.79 %)
yes	6,155	(11.36 %)	2,286	(11.2 %)
Outcome renal failure				
no	45,920	(84.77 %)	18,279	(89.62 %)
yes	8,248	(15.22 %)	2,116	(10.37 %)
**CASE CHARACTERISTICS**				
age (median)	68	(56; 77)	67	(57; 77)
elixhauser groups per case (median)	3	(2; 5)	3	(2; 5)
sex				
male	26,954	(49.76 %)	10,367	(50.83 %)
female	27,214	(50.23 %)	10,028	(49.16 %)
AIDS/HIV				
no	54,142	(99.95 %)	20,383	(99.94 %)
yes	26	(0.04 %)	12	(0.05 %)
alcohol abuse				
no	53,025	(97.88 %)	20,088	(98.49 %)
yes	1,143	(2.11 %)	307	(1.5 %)
blood loss anemia				
no	53,022	(97.88 %)	20,134	(98.72 %)
yes	1,146	(2.11 %)	261	(1.27 %)
cardiac arrhythmias				
no	43,241	(79.82 %)	17,135	(84.01 %)
yes	10,927	(20.17 %)	3,260	(15.98 %)
chronic pulmonary disease				
no	49,042	(90.53 %)	18,792	(92.14 %)
yes	5,126	(9.46 %)	1,603	(7.85 %)
coagulopathy				
no	44,935	(82.95 %)	17,598	(86.28 %)
yes	9,233	(17.04 %)	2,797	(13.71 %)
congestive heart failure				
no	47,447	(87.59 %)	18,608	(91.23 %)
yes	6,721	(12.4 %)	1,787	(8.76 %)
deficiency anemia				
no	51,887	(95.78 %)	19,852	(97.33 %)
yes	2,281	(4.21 %)	543	(2.66 %)
depression				
no	51,232	(94.57 %)	19,318	(94.71 %)
yes	2,936	(5.42 %)	1,077	(5.28 %)
diabetes, complicated				
no	51,887	(95.78 %)	19,754	(96.85 %)
yes	2,281	(4.21 %)	641	(3.14 %)
diabetes, uncomplicated				
no	46,491	(85.82 %)	17,664	(86.6 %)
yes	7,677	(14.17 %)	2,731	(13.39 %)
drug abuse				
no	53,911	(99.52 %)	20,321	(99.63 %)
yes	257	(0.47 %)	74	(0.36 %)
fluid and electrolyte disorders				
no	29,678	(54.78 %)	12,122	(59.43 %)
yes	24,490	(45.21 %)	8,273	(40.56 %)
hypertension, complicated				
no	51,632	(95.31 %)	19,680	(96.49 %)
yes	2,536	(4.68 %)	715	(3.51 %)
hypertension, uncomplicated				
no	28,369	(52.37 %)	10,692	(52.42 %)
yes	25,799	(47.62 %)	9,703	(47.57 %)

hypothyroidism				
no	47,235	(87.2 %)	18,008	(88.29 %)
yes	6,933	(12.79 %)	2,387	(11.7 %)
liver disease				
no	50,277	(92.81 %)	19,433	(95.28 %)
yes	3,891	(7.18 %)	962	(4.71 %)
lymphoma				
no	53,762	(99.25 %)	20,315	(99.6 %)
yes	406	(0.74 %)	80	(0.39 %)
metastatic cancer				
no	43,637	(80.55 %)	15,149	(74.27 %)
yes	10,531	(19.44 %)	5,246	(25.72 %)
obesity				
no	47,727	(88.11 %)	18,046	(88.48 %)
yes	6,441	(11.89 %)	2,349	(11.51 %)
other neurological disorders				
no	51,956	(95.91 %)	19,747	(96.82 %)
yes	2,212	(4.08 %)	648	(3.17 %)
paralysis				
no	52,765	(97.4 %)	19,989	(98.01 %)
yes	1,403	(2.59 %)	406	(1.99 %)
peptic ulcer disease excluding bleeding				
no	54,004	(99.69 %)	20,348	(99.76 %)
yes	164	(0.31 %)	47	(0.23 %)
peripheral vascular disorders				
no	49,190	(90.81 %)	18,228	(89.37 %)
yes	4,978	(9.18 %)	2,167	(10.62 %)
psychoses				
no	53,889	(99.48 %)	20,330	(99.68 %)
yes	279	(0.51 %)	65	(0.31 %)
pulmonary circulation disorders				
no	52,476	(96.87 %)	19,929	(97.71 %)
yes	1,692	(3.12 %)	466	(2.28 %)
renal Diseases				
no	45,739	(84.43 %)	17,896	(87.74 %)
yes	8,429	(15.56 %)	2,499	(12.25 %)
rheumatoid arthritis/ collagen vascular diseases				
no	53,262	(98.32 %)	20,115	(98.62 %)
yes	906	(1.67 %)	280	(1.37 %)
solid tumor without metastasis				
no	28,520	(52.65 %)	6,722	(32.95 %)
yes	25,648	(47.34 %)	13,673	(67.04 %)
valvular disease				
no	51,473	(95.02 %)	19,591	(96.05 %)
yes	2,695	(4.97 %)	804	(3.94 %)
weight loss				
no	46,134	(85.16 %)	17,442	(85.52 %)
yes	8,034	(14.83 %)	2,953	(14.47 %)
**HEALTHCARE CHARACTERISTICS**				
total colon resections				
no	51,310	(94.72 %)	20,173	(98.91 %)
yes	2,858	(5.27 %)	222	(1.08 %)
partial colon resections				
no	2,662	(4.91 %)	17,088	(83.78 %)
yes	51,506	(95.08 %)	3,307	(16.21 %)
rectum resections				
no	50,665	(93.53 %)	-	-
yes	3,503	(6.46 %)	20,395	(100.00 %)
colon and rectum resection				
no	50,665	(93.53 %)	16,892	(82.82 %)
yes	3,503	(6.46 %)	3,503	(17.17 %)
weekend surgery				
no	49,473	(91.33 %)	19,603	(96.11 %)
yes	4,695	(8.66 %)	792	(3.88 %)
admission				
referral	36,129	(66.69 %)	16,249	(79.67 %)
emergency case	16,116	(29.75 %)	3,744	(18.35 %)
transfer from other hospital	1,923	(3.55 %)	402	(1.97 %)

For peer review only - http://bmjopen.bmj.com/site/about/guidelines.xhtml

HOSPITAL CHARACTERISTICS				
annual volume				
colon resection cases (median)	72	(38; 119)	-	-
total colon resection (median)	1	(0;3)	-	-
rectum resections (median)	-	-	26	(11;42)
urbanization				
urban	124	(59.33 %)	119	(59.50 %)
rural	85	(40.66 %)	81	(40.50 %)
ownership				
public	82	(39.23 %)	80	(40.00 %)
non-profit	41	(19.61 %)	39	(19.50 %)
private	86	(41.14 %)	81	(40.50 %)
university hospital				
no	201	(96.17 %)	192	(96.00 %)
yes	8	(3.82 %)	8	(4.00 %)

For peer review only

S7: Bivariate analysis of case-, care- and hospital-related covariates of patient safety including all 54.168 colon resections in 209 hospitals

	IN-HOSPITAL DEATH		POST-OPERATIVE RESPIRATORY FAILURE		RENAL FAILURE		POST-OPERATIVE WOUND INFECTION	
	OR	95% CI	OR	95% CI	OR	95% CI	OR	95% CI
**PATIENT COVARIATES**								
sex								
male	Ref.		Ref.		Ref.		Ref.	
female	0.876***	(0.827 - 0.928)	0.828***	(0.790 - 0.868)	0.742***	(0.707 - 0.778)	0.931**	(0.881 - 0.983)
age	1.052***	(1.049 - 1.055)	1.030***	(1.028 - 1.031)	1.037***	(1.035 - 1.039)	1.008***	(1.006 - 1.009)
alcohol abuse	3.845***	(3.363 - 4.396)	3.862***	(3.400 - 4.387)	3.860***	(3.406 - 4.374)	1.945***	(1.669 - 2.266)
blood loss anemia	0.715**	(0.570 - 0.896)	1.355***	(1.159 - 1.585)	1.200*	(1.025 - 1.405)	1.387***	(1.159 - 1.659)
cardiac arrhythmias	4.338***	(4.084 - 4.608)	3.544***	(3.364 - 3.734)	3.995***	(3.791 - 4.209)	1.766***	(1.660 - 1.878)
chronic pulmonary disease	2.025***	(1.867 - 2.196)	2.120***	(1.978 - 2.273)	1.793***	(1.668 - 1.927)	1.554***	(1.431 - 1.689)
coagulopathy	9.015***	(8.439 - 9.630)	6.370***	(6.011 - 6.751)	7.207***	(6.798 - 7.640)	2.483***	(2.331 - 2.646)
congestive heart failure	4.645***	(4.342 - 4.969)	4.294***	(4.042 - 4.563)	4.382***	(4.127 - 4.652)	1.899***	(1.765 - 2.044)
deficiency anemia	1.013	(0.878 - 1.169)	1.448***	(1.302 - 1.610)	1.416***	(1.268 - 1.582)	1.250***	(1.101 - 1.418)
depression	0.888	(0.779 - 1.012)	1.827***	(1.669 - 2.000)	1.372***	(1.244 - 1.513)	1.923***	(1.741 - 2.125)
diabetes, complicated	2.766***	(2.481 - 3.084)	2.339***	(2.121 - 2.578)	2.946***	(2.678 - 3.240)	1.705***	(1.514 - 1.921)
diabetes, uncomplicated	1.485***	(1.378 - 1.600)	1.559***	(1.465 - 1.659)	1.594***	(1.497 - 1.698)	1.300***	(1.208 - 1.399)
fluid and electrolyte disorders	4.072***	(3.809 - 4.354)	4.463***	(4.229 - 4.709)	5.033***	(4.756 - 5.328)	2.650***	(2.497 - 2.812)
hypertension, complicated	2.124***	(1.896 - 2.380)	2.156***	(1.957 - 2.375)	2.446***	(2.225 - 2.688)	1.453***	(1.288 - 1.638)
hypertension, uncomplicated	0.961	(0.906 - 1.020)	1.266***	(1.207 - 1.329)	1.294***	(1.232 - 1.360)	1.198***	(1.132 - 1.267)
hypothyroidism	0.964	(0.883 - 1.052)	1.227***	(1.147 - 1.312)	1.176***	(1.096 - 1.261)	1.316***	(1.219 - 1.421)
liver disease	6.486***	(6.007 - 7.004)	2.844***	(2.635 - 3.069)	4.573***	(4.254 - 4.917)	1.345***	(1.223 - 1.478)
lymphoma	1.869***	(1.441 - 2.424)	1.319*	(1.030 - 1.690)	1.850***	(1.470 - 2.327)	1.246	(0.939 - 1.654)
metastatic cancer	0.948	(0.881 - 1.021)	1.004	(0.946 - 1.065)	0.827***	(0.777 - 0.881)	1.069	(1.000 - 1.143)
obesity	0.780***	(0.707 - 0.860)	1.173***	(1.092 - 1.259)	1.419***	(1.324 - 1.521)	1.656***	(1.533 - 1.789)
other neurological disorders	2.854***	(2.567 - 3.174)	3.417***	(3.109 - 3.754)	2.834***	(2.575 - 3.119)	1.860***	(1.660 - 2.084)
paralysis	2.709***	(2.373 - 3.092)	3.030***	(2.692 - 3.410)	2.435***	(2.156 - 2.751)	1.874***	(1.629 - 2.156)
peripheral vascular disorders	3.572***	(3.316 - 3.848)	2.745***	(2.562 - 2.941)	3.001***	(2.804 - 3.213)	1.741***	(1.606 - 1.889)
psychoses	1.004	(0.676 - 1.492)	2.578***	(1.978 - 3.361)	1.232	(0.903 - 1.679)	1.845***	(1.352 - 2.517)
pulmonary circulation disorders	3.533***	(3.150 - 3.964)	3.959***	(3.562 - 4.401)	3.185***	(2.863 - 3.543)	1.721***	(1.511 - 1.960)
renal failure	2.772***	(2.592 - 2.966)	2.261***	(2.132 - 2.398)	2.982***	(2.815 - 3.158)	1.524***	(1.419 - 1.636)
rheumatoid arthritis/collagen vascular diseases	1.393**	(1.141 - 1.702)	1.283**	(1.079 - 1.524)	1.373***	(1.156 - 1.630)	1.196	(0.980 - 1.461)
solid tumor without metastasis	0.660***	(0.622 - 0.701)	0.845***	(0.806 - 0.886)	0.760***	(0.724 - 0.798)	0.949	(0.898 - 1.002)
valvular disease	2.580***	(2.332 - 2.855)	2.355***	(2.151 - 2.579)	2.632***	(2.409 - 2.876)	1.426***	(1.275 - 1.594)
weight loss	1.439***	(1.328 - 1.560)	2.418***	(2.269 - 2.576)	2.091***	(1.963 - 2.228)	2.230***	(2.073 - 2.398)
**HEALTHCARE COVARIATES**								
admission								
referral	Ref.		Ref.		Ref.		Ref.	
emergency case	3.066***	(2.852 - 3.297)	2.263***	(2.133 - 2.401)	2.409***	(2.260 - 2.567)	1.461***	(1.368 - 1.559)
transfer from other hospital	5.722***	(5.105 - 6.412)	4.200***	(3.768 - 4.680)	4.308***	(3.870 - 4.795)	1.865***	(1.645 - 2.115)
weekend Surgery								
no	Ref.		Ref.		Ref.		Ref.	
yes	2.704***	(2.498 - 2.927)	2.148***	(1.999 - 2.308)	2.254***	(2.099 - 2.419)	1.382***	(1.265 - 1.509)

total colon resection								
no	Ref.		Ref.		Ref.		Ref.	
yes	3.687***	(3.351 - 4.057)	2.395***	(2.186 - 2.625)	2.954***	(2.704 - 3.227)	1.340***	(1.204 - 1.493)
colon and rectum resection								
no	Ref.		Ref.		Ref.		Ref.	
yes	1.114	(0.991 - 1.252)	1.563***	(1.429 - 1.710)	1.390***	(1.267 - 1.525)	1.757***	(1.593 - 1.937)
**HOSPITAL COVARIATES**								
case volume	1.071	(0.978 - 1.173)	0.934	(0.837 - 1.043)	1.044	(0.944 - 1.155)	1.265***	(1.117 - 1.432)
area								
urban	Ref.		Ref.		Ref.		Ref.	
rural	0.830*	(0.711 - 0.969)	0.849	(0.665 - 1.085)	0.760**	(0.624 - 0.927)	0.837	(0.661 - 1.059)
university hospital								
no	Ref.		Ref.		Ref.		Ref.	
yes	1.946***	(1.411 - 2.683)	1.003	(0.555 - 1.814)	1.767*	(1.113 - 2.806)	3.178***	(1.894 - 5.333)
ownership								
public	Ref.		Ref.		Ref.		Ref.	
non-profit	0.774*	(0.634 - 0.945)	0.915	(0.657 - 1.272)	0.691**	(0.534 - 0.895)	0.615**	(0.452 - 0.835)
private	0.748***	(0.636 - 0.880)	1.025	(0.785 - 1.338)	1.226	(0.999 - 1.504)	0.662**	(0.518 - 0.847)

Note: Elixhauser Groups drug abuse, peptic ulcer disease and AIDS/ HIV were not included into the regression due to low case numbers
*** p<0.001
** p <0.01
* p<0.05

For peer review only

S8: Bivariate analysis of case-, care- and hospital-related covariates of patient safety including all 20.395 rectum resections in 200 hospitals

-	IN-HOSPITAL DEATH		POST-OPERATIVE RESPIRATORY FAILURE		RENAL FAILURE		POST-OPERATIVE WOUND INFECTION	
	OR	95% CI	OR	95% CI	OR	95% CI	OR	95% CI
**PATIENT COVARIATES**								
sex								
male	Ref.		Ref.		Ref.		Ref.	
female	0.915	(0.796 - 1.050)	0.913*	(0.836 - 0.998)	0.787***	(0.717 - 0.864)	0.877**	(0.801 - 0.960)
age	1.075***	(1.067 - 1.082)	1.031***	(1.027 - 1.034)	1.039***	(1.035 - 1.043)	1.007***	(1.004 - 1.010)
alcohol abuse	2.767***	(1.915 - 3.997)	2.705***	(2.050 - 3.570)	2.531***	(1.916 - 3.345)	1.826***	(1.356 - 2.459)
blood loss anemia	1.984**	(1.270 - 3.100)	1.769**	(1.256 - 2.493)	2.637***	(1.942 - 3.581)	2.264***	(1.619 - 3.165)
cardiac arrhythmias	5.465***	(4.741 - 6.299)	3.457***	(3.124 - 3.825)	3.777***	(3.410 - 4.183)	1.564***	(1.399 - 1.748)
chronic pulmonary disease	2.213***	(1.820 - 2.690)	2.185***	(1.905 - 2.506)	1.835***	(1.587 - 2.122)	1.690***	(1.463 - 1.952)
coagulopathy	9.352***	(8.020 - 10.906)	5.781***	(5.171 - 6.463)	5.702***	(5.091 - 6.386)	2.680***	(2.401 - 2.993)
congestive heart failure	5.853***	(4.995 - 6.858)	4.275***	(3.781 - 4.834)	4.272***	(3.787 - 4.820)	1.793***	(1.560 - 2.060)
deficiency anemia	1.741**	(1.241 - 2.442)	2.119***	(1.696 - 2.648)	2.275***	(1.810 - 2.858)	1.893***	(1.499 - 2.391)
depression	0.872	(0.637 - 1.192)	1.675***	(1.409 - 1.991)	1.686***	(1.411 - 2.014)	1.701***	(1.436 - 2.014)
diabetes, complicated	3.196***	(2.455 - 4.160)	2.073***	(1.692 - 2.540)	3.187***	(2.635 - 3.854)	1.730***	(1.390 - 2.154)
diabetes, uncomplicated	1.653***	(1.388 - 1.969)	1.613***	(1.435 - 1.813)	1.660***	(1.472 - 1.873)	1.341***	(1.184 - 1.518)
fluid and electrolyte disorders	3.961***	(3.387 - 4.633)	4.043***	(3.660 - 4.467)	4.586***	(4.122 - 5.102)	2.563***	(2.324 - 2.826)
hypertension, complicated	2.656***	(2.033 - 3.470)	2.366***	(1.947 - 2.876)	2.681***	(2.225 - 3.230)	1.480***	(1.188 - 1.844)
hypertension, uncomplicated	1.094	(0.951 - 1.257)	1.358***	(1.241 - 1.486)	1.449***	(1.318 - 1.593)	1.159**	(1.057 - 1.271)
hypothyroidism	0.980	(0.790 - 1.215)	1.164*	(1.019 - 1.329)	1.170*	(1.019 - 1.344)	1.125	(0.981 - 1.290)
liver disease	5.887***	(4.864 - 7.124)	2.298***	(1.937 - 2.725)	3.606***	(3.084 - 4.216)	1.539***	(1.279 - 1.850)
lymphoma	2.350*	(1.112 - 4.967)	1.618	(0.882 - 2.967)	1.766	(0.972 - 3.208)	1.683	(0.946 - 2.992)
metastatic cancer	0.979	(0.835 - 1.148)	1.298***	(1.177 - 1.431)	0.978	(0.879 - 1.087)	1.187***	(1.075 - 1.311)
obesity	0.908	(0.726 - 1.135)	1.500***	(1.320 - 1.704)	1.632***	(1.435 - 1.855)	1.653***	(1.455 - 1.878)
other neurological disorders	3.942***	(3.107 - 5.001)	3.107***	(2.569 - 3.757)	2.277***	(1.860 - 2.788)	1.887***	(1.530 - 2.327)
paralysis	3.060***	(2.244 - 4.173)	2.958***	(2.336 - 3.745)	2.816***	(2.220 - 3.572)	1.849***	(1.425 - 2.399)
peripheral vascular disorders	2.521***	(2.118 - 3.000)	1.673***	(1.469 - 1.905)	1.924***	(1.690 - 2.189)	1.531***	(1.341 - 1.748)
psychoses	0.943	(0.291 - 3.056)	0.843	(0.371 - 1.914)	1.865	(0.969 - 3.592)	1.337	(0.660 - 2.707)
pulmonary circulation disorders	6.535***	(5.123 - 8.336)	4.514***	(3.663 - 5.563)	3.734***	(3.022 - 4.613)	2.041***	(1.619 - 2.573)
renal failure	2.984***	(2.532 - 3.518)	2.416***	(2.147 - 2.718)	3.393***	(3.031 - 3.797)	1.688***	(1.488 - 1.915)
rheumatoid arthritis/collagen vascular diseases	1.521	(0.932 - 2.482)	1.582**	(1.140 - 2.196)	1.471*	(1.043 - 2.076)	0.868	(0.580 - 1.301)
solid tumor without metastasis	0.571***	(0.494 - 0.659)	1.002	(0.906 - 1.107)	0.836***	(0.755 - 0.924)	1.090	(0.984 - 1.207)
valvular disease	3.682***	(2.935 - 4.619)	2.693***	(2.257 - 3.214)	2.803***	(2.351 - 3.341)	1.364**	(1.113 - 1.672)
weight loss	1.408***	(1.165 - 1.702)	2.522***	(2.244 - 2.834)	2.072***	(1.842 - 2.331)	2.079***	(1.843 - 2.346)
**HEALTHCARE COVARIATES**								
admission reason								
referral	Ref.		Ref.		Ref.		Ref.	
emergency case	4.038***	(3.450 - 4.727)	2.200***	(1.963 - 2.465)	2.272***	(2.013 - 2.563)	1.656***	(1.474 - 1.862)
transfer from other hospital	6.550***	(4.887 - 8.780)	3.944***	(3.088 - 5.038)	4.248***	(3.337 - 5.406)	2.208***	(1.705 - 2.861)
weekend surgery								
no	Ref.		Ref.		Ref.		Ref.	
yes	3.693***	(2.927 - 4.659)	2.215***	(1.831 - 2.678)	2.177***	(1.809 - 2.620)	1.281*	(1.030 - 1.593)
colon and rectum resection								

no	Ref.		Ref.		Ref.		Ref.	
yes	4.015***	(3.458 - 4.663)	3.095***	(2.781 - 3.444)	2.706***	(2.428 - 3.016)	2.014***	(1.805 - 2.247)
**HOSPITAL COVARIATES**								
case volume	0.743***	(0.661 - 0.835)	0.828**	(0.730 - 0.940)	0.830***	(0.746 - 0.924)	1.079	(0.946 - 1.232)
area								
urban	Ref.		Ref.		Ref.		Ref.	
rural	0.882	(0.680 - 1.143)	0.925	(0.684 - 1.251)	0.895	(0.709 - 1.129)	0.726*	(0.554 - 0.951)
university hospital								
no	Ref.		Ref.		Ref.		Ref.	
yes	1.439	(0.890 - 2.324)	0.857	(0.435 - 1.690)	1.134	(0.696 - 1.847)	3.037***	(1.778 - 5.186)
ownership								
public	Ref.		Ref.		Ref.		Ref.	
non-profit	0.691*	(0.500 - 0.954)	0.887	(0.594 - 1.324)	0.633**	(0.472 - 0.848)	0.605**	(0.428 - 0.854)
private	0.631***	(0.480 - 0.830)	1.070	(0.773 - 1.482)	1.245	(0.988 - 1.571)	0.673**	(0.508 - 0.893)

Note: Elixhauser Groups drug abuse, peptic ulcer disease and AIDS/ HIV were not included into the regression due to a low case numbers
*** p<0.001
** p <0.01
* p<0.05

For peer review only

S9: Multivariate analysis of case-, care- and hospital-related covariates of patient safety including all 54.168 colon resections in 209 hospitals

	IN-HOSPITAL DEATH		POST-OPERATIVE RESPIRATORY FAILURE		RENAL FAILURE		POST-OPERATIVE WOUND INFECTION	
	OR	95% CI	OR	95% CI	OR	95% CI	OR	95% CI
**CASE COVARIATES**								
sex								
male	Ref.		Ref		Ref		Ref	
female	0.937	(0.873 - 1.006)	0.788***	(0.745 - 0.833)	0.683***	(0.645 - 0.725)	0.882***	(0.832 - 0.936)
age	1.050***	(1.046 - 1.053)	1.014***	(1.012 - 1.017)	1.024***	(1.021 - 1.026)	0.998	(0.996 - 1.000)
alcohol abuse	1.484***	(1.250 - 1.761)	1.948***	(1.677 - 2.263)	1.574***	(1.352 - 1.833)	1.304**	(1.106 - 1.539)
blood loss anemia	0.472***	(0.365 - 0.609)	0.982	(0.823 - 1.172)	0.831*	(0.695 - 0.995)	1.154	(0.958 - 1.390)
cardiac arrhythmias	1.640***	(1.518 - 1.772)	1.585***	(1.487 - 1.690)	1.627***	(1.524 - 1.737)	1.183***	(1.100 - 1.271)
chronic pulmonary disease	1.362***	(1.234 - 1.503)	1.459***	(1.347 - 1.581)	1.113*	(1.022 - 1.212)	1.242***	(1.138 - 1.356)
coagulopathy	4.174***	(3.864 - 4.509)	3.117***	(2.920 - 3.327)	3.332***	(3.118 - 3.561)	1.644***	(1.531 - 1.764)
congestive heart failure	1.748***	(1.597 - 1.914)	1.902***	(1.759 - 2.056)	1.678***	(1.551 - 1.815)	1.218***	(1.114 - 1.332)
deficiency anemia	0.778**	(0.660 - 0.918)	1.058	(0.937 - 1.194)	1.096	(0.965 - 1.245)	1.024	(0.897 - 1.169)
depression	0.658***	(0.566 - 0.765)	1.425***	(1.286 - 1.580)	1.070	(0.956 - 1.197)	1.569***	(1.414 - 1.741)
diabetes, complicated	1.297***	(1.131 - 1.487)	1.181**	(1.051 - 1.328)	1.274***	(1.135 - 1.431)	1.191**	(1.045 - 1.358)
diabetes, uncomplicated	1.128*	(1.029 - 1.237)	1.201***	(1.117 - 1.291)	1.151***	(1.067 - 1.241)	1.113**	(1.028 - 1.204)
fluid and electrolyte disorders	1.884***	(1.743 - 2.038)	2.561***	(2.412 - 2.719)	2.882***	(2.704 - 3.072)	1.991***	(1.867 - 2.123)
hypertension, complicated	0.507***	(0.437 - 0.589)	0.735***	(0.649 - 0.832)	0.805***	(0.712 - 0.911)	0.928	(0.807 - 1.067)
hypertension, uncomplicated	0.551***	(0.511 - 0.595)	0.945	(0.890 - 1.003)	0.921*	(0.865 - 0.981)	1.045	(0.980 - 1.115)
hypothyroidism	0.795***	(0.716 - 0.884)	1.110**	(1.027 - 1.200)	1.046	(0.963 - 1.137)	1.206***	(1.112 - 1.309)
liver disease	4.411***	(3.999 - 4.865)	1.373***	(1.253 - 1.506)	2.588***	(2.368 - 2.829)	0.831***	(0.749 - 0.923)
lymphoma	0.983	(0.717 - 1.349)	0.810	(0.613 - 1.070)	1.111	(0.846 - 1.458)	1.029	(0.767 - 1.382)
metastatic cancer	1.524***	(1.376 - 1.687)	1.120**	(1.036 - 1.211)	0.984	(0.906 - 1.070)	1.058	(0.974 - 1.149)
obesity	0.766***	(0.682 - 0.862)	1.165***	(1.073 - 1.265)	1.498***	(1.379 - 1.627)	1.634***	(1.506 - 1.773)
other neurological disorders	1.504***	(1.319 - 1.714)	1.903***	(1.706 - 2.123)	1.515***	(1.352 - 1.699)	1.259***	(1.114 - 1.422)
paralysis	1.481***	(1.261 - 1.740)	1.468***	(1.281 - 1.682)	1.142	(0.989 - 1.318)	1.231**	(1.060 - 1.429)
peripheral vascular disorders	1.926***	(1.757 - 2.111)	1.493***	(1.378 - 1.618)	1.518***	(1.399 - 1.647)	1.239***	(1.136 - 1.351)
psychoses	0.707	(0.449 - 1.113)	2.052***	(1.516 - 2.778)	0.859	(0.600 - 1.229)	1.493*	(1.081 - 2.061)
pulmonary circulation disorders	1.593***	(1.380 - 1.838)	1.864***	(1.649 - 2.108)	1.336***	(1.174 - 1.519)	1.071	(0.933 - 1.231)
renal failure	1.308***	(1.200 - 1.425)	1.112**	(1.035 - 1.196)	1.505***	(1.401 - 1.616)	1.067	(0.984 - 1.156)
rheumatoid arthritis/collagen vascular diseases	1.001	(0.793 - 1.264)	0.971	(0.798 - 1.182)	0.982	(0.806 - 1.197)	1.056	(0.858 - 1.298)
solid tumor without metastasis	0.573***	(0.525 - 0.625)	0.764***	(0.715 - 0.816)	0.707***	(0.660 - 0.757)	0.907**	(0.845 - 0.975)
valvular disease	0.817**	(0.719 - 0.929)	0.862**	(0.772 - 0.961)	0.927	(0.831 - 1.033)	0.927	(0.820 - 1.048)
weight loss	0.891*	(0.809 - 0.980)	1.658***	(1.542 - 1.783)	1.426***	(1.324 - 1.535)	1.774***	(1.643 - 1.916)
**HEALTHCARE COVARIATES**								
admission reason								
referral	Ref.		Ref.		Ref.		Ref.	
emergency case	1.847***	(1.692 - 2.015)	1.413***	(1.320 - 1.513)	1.453***	(1.349 - 1.566)	1.145***	(1.067 - 1.228)
transfer from other hospital	2.528***	(2.193 - 2.915)	1.982***	(1.749 - 2.245)	1.908***	(1.678 - 2.171)	1.223**	(1.071 - 1.397)
weekend surgery								
no	Ref.		Ref.		Ref.		Ref.	
yes	1.669***	(1.515 - 1.839)	1.426***	(1.312 - 1.550)	1.480***	(1.360 - 1.610)	1.080	(0.984 - 1.186)

total colon resection								
no	Ref.		Ref.		Ref.		Ref.	
yes	2.679***	(2.369 - 3.029)	1.639***	(1.472 - 1.825)	2.228***	(1.999 - 2.483)	1.022	(0.913 - 1.143)
colon and rectum resection								
no	Ref.		Ref.		Ref.		Ref.	
yes	1.103	(0.960 - 1.267)	1.524***	(1.378 - 1.686)	1.408***	(1.265 - 1.567)	1.579***	(1.426 - 1.748)
**HOSPITAL COVARIATES**								
case volume	0.968	(0.871 - 1.076)	0.919	(0.807 - 1.047)	0.992	(0.891 - 1.106)	1.168*	(1.030 - 1.325)
area								
urban	Ref.		Ref.		Ref.		Ref.	
rural	1.061	(0.893 - 1.261)	0.863	(0.648 - 1.149)	0.772**	(0.635 - 0.939)	1.032	(0.824 - 1.292)
university hospital								
no	Ref.		Ref.		Ref.		Ref.	
yes	1.303	(0.888 - 1.912)	0.687	(0.338 - 1.397)	1.412	(0.889 - 2.241)	1.981*	(1.171 - 3.352)
ownership								
public	Ref.		Ref.		Ref.		Ref.	
non-profit	1.012	(0.811 - 1.262)	1.057	(0.726 - 1.540)	0.867	(0.670 - 1.122)	0.744*	(0.555 - 0.998)
private	1.244*	(1.026 - 1.507)	1.329	(0.972 - 1.817)	1.937***	(1.563 - 2.400)	0.777*	(0.608 - 0.992)

Note: Elixhauser Groups drug abuse, peptic ulcer disease and AIDS/ HIV were not included into the regression due to a low case numbers

*** p<0.001

** p <0.01

* p<0.05

For peer review only

S10: Multivariate analysis of case-, care- and hospital-related covariates of patient safety including all 20.395 rectum resections in 200 hospitals

	IN-HOSPITAL DEATH		POST-OPERATIVE RESPIRATORY FAILURE		RENAL FAILURE		POST-OPERATIVE WOUND INFECTION	
	OR	95% CI	OR	95% CI	OR	95% CI	OR	95% CI
**CASE COVARIATES**								
sex								
male	Ref.		Ref.		Ref.		Ref.	
female	0.842*	(0.710 - 0.998)	0.842**	(0.759 - 0.934)	0.685***	(0.613 - 0.765)	0.826***	(0.748 - 0.912)
age	1.068***	(1.059 - 1.078)	1.014***	(1.009 - 1.018)	1.021***	(1.016 - 1.026)	0.998	(0.995 - 1.002)
alcohol abuse	1.410	(0.891 - 2.231)	1.656**	(1.200 - 2.286)	1.207	(0.869 - 1.676)	1.143	(0.828 - 1.578)
blood loss anemia	0.829	(0.486 - 1.413)	0.890	(0.607 - 1.305)	1.383	(0.976 - 1.959)	1.579*	(1.113 - 2.241)
cardiac arrhythmias	1.832***	(1.535 - 2.188)	1.662***	(1.471 - 1.878)	1.639***	(1.448 - 1.854)	1.034	(0.909 - 1.176)
chronic pulmonary disease	1.303*	(1.030 - 1.648)	1.510***	(1.291 - 1.766)	1.156	(0.979 - 1.366)	1.369***	(1.175 - 1.594)
coagulopathy	4.309***	(3.600 - 5.158)	3.052***	(2.697 - 3.455)	2.886***	(2.541 - 3.277)	1.770***	(1.570 - 1.996)
congestive heart failure	1.825***	(1.481 - 2.248)	1.849***	(1.583 - 2.159)	1.536***	(1.317 - 1.792)	1.099	(0.929 - 1.300)
deficiency anemia	0.880	(0.593 - 1.304)	1.242	(0.967 - 1.595)	1.357*	(1.052 - 1.750)	1.332*	(1.043 - 1.700)
depression	0.629*	(0.439 - 0.900)	1.222*	(1.005 - 1.486)	1.290*	(1.056 - 1.577)	1.386***	(1.161 - 1.654)
diabetes, complicated	1.584**	(1.145 - 2.192)	1.045	(0.824 - 1.326)	1.441**	(1.153 - 1.801)	1.172	(0.922 - 1.490)
diabetes, uncomplicated	1.124	(0.911 - 1.388)	1.194*	(1.043 - 1.368)	1.191*	(1.036 - 1.369)	1.164*	(1.018 - 1.331)
fluid and electrolyte disorders	1.704***	(1.425 - 2.039)	2.432***	(2.181 - 2.712)	2.855***	(2.542 - 3.206)	1.952***	(1.759 - 2.166)
hypertension, complicated	0.461***	(0.324 - 0.656)	0.757*	(0.591 - 0.970)	0.836	(0.659 - 1.060)	0.911	(0.704 - 1.178)
hypertension, uncomplicated	0.584***	(0.490 - 0.695)	0.980	(0.878 - 1.093)	0.999	(0.890 - 1.121)	0.993	(0.893 - 1.104)
hypothyroidism	0.812	(0.632 - 1.043)	0.993	(0.854 - 1.155)	1.002	(0.856 - 1.174)	1.035	(0.895 - 1.198)
liver disease	4.027***	(3.155 - 5.139)	1.268*	(1.036 - 1.553)	2.312***	(1.920 - 2.783)	1.036	(0.848 - 1.267)
lymphoma	1.308	(0.563 - 3.042)	1.028	(0.532 - 1.986)	0.930	(0.476 - 1.814)	1.320	(0.718 - 2.427)
metastatic cancer	1.376**	(1.118 - 1.694)	1.211**	(1.073 - 1.367)	0.983	(0.863 - 1.121)	1.051	(0.937 - 1.179)
obesity	0.922	(0.708 - 1.202)	1.501***	(1.296 - 1.738)	1.563***	(1.348 - 1.812)	1.638***	(1.431 - 1.876)
other neurological disorders	2.306***	(1.722 - 3.089)	1.879***	(1.507 - 2.343)	1.184	(0.936 - 1.498)	1.376**	(1.098 - 1.725)
paralysis	1.153	(0.785 - 1.695)	1.391*	(1.056 - 1.833)	1.320	(0.999 - 1.744)	1.110	(0.839 - 1.469)
peripheral vascular disorders	1.908***	(1.548 - 2.353)	1.253**	(1.083 - 1.450)	1.369***	(1.181 - 1.587)	1.283***	(1.117 - 1.475)
psychoses	0.867	(0.205 - 3.672)	0.618	(0.246 - 1.551)	2.075	(0.986 - 4.369)	1.072	(0.507 - 2.264)
pulmonary circulation disorders	2.816***	(2.076 - 3.821)	1.935***	(1.522 - 2.460)	1.460**	(1.140 - 1.870)	1.208	(0.941 - 1.551)
renal failure	1.124	(0.917 - 1.377)	1.252**	(1.087 - 1.442)	1.766***	(1.544 - 2.020)	1.278***	(1.108 - 1.473)
rheumatoid arthritis/collagen vascular diseases	0.954	(0.531 - 1.713)	1.248	(0.860 - 1.812)	1.014	(0.683 - 1.507)	0.761	(0.501 - 1.158)
solid tumor without metastasis	0.599***	(0.491 - 0.731)	0.962	(0.846 - 1.094)	0.798***	(0.700 - 0.909)	1.070	(0.946 - 1.211)
valvular disease	1.000	(0.750 - 1.333)	1.075	(0.870 - 1.328)	1.042	(0.844 - 1.287)	0.893	(0.713 - 1.117)
weight loss	0.906	(0.729 - 1.127)	1.810***	(1.590 - 2.060)	1.448***	(1.268 - 1.653)	1.679***	(1.479 - 1.905)
**HEALTHCARE COVARIATES**								
admission reason								
referral	Ref.		Ref.		Ref.		Ref.	
emergency case	2.028***	(1.675 - 2.454)	1.335***	(1.170 - 1.523)	1.342***	(1.169 - 1.540)	1.291***	(1.138 - 1.466)
transfer from other hospital	2.679***	(1.874 - 3.828)	1.859***	(1.406 - 2.459)	1.927***	(1.461 - 2.541)	1.484**	(1.131 - 1.948)
weekend surgery								
no	Ref.		Ref.		Ref.		Ref.	
yes	1.960***	(1.483 - 2.591)	1.427**	(1.150 - 1.770)	1.391**	(1.127 - 1.717)	0.985	(0.784 - 1.238)

colon and rectum resection								
no	Ref.		Ref.		Ref.		Ref.	
yes	2.579***	(2.163 - 3.074)	2.164***	(1.921 - 2.438)	1.859***	(1.645 - 2.100)	1.522***	(1.356 - 1.708)
**HOSPITAL COVARIATES**								
case volume	0.703***	(0.611 - 0.809)	0.844*	(0.725 - 0.982)	0.853**	(0.760 - 0.958)	0.973	(0.854 - 1.109)
area								
urban	Ref.		Ref.		Ref.		Ref.	
rural	1.072	(0.817 - 1.407)	0.904	(0.639 - 1.281)	0.834	(0.670 - 1.037)	0.854	(0.663 - 1.101)
university hospital								
no	Ref.		Ref.		Ref.		Ref.	
yes	1.616	(0.979 - 2.665)	0.853	(0.379 - 1.920)	1.299	(0.829 - 2.037)	2.292**	(1.358 - 3.869)
ownership								
public	Ref.		Ref.		Ref.		Ref.	
non-profit	0.851	(0.614 - 1.179)	1.059	(0.674 - 1.664)	0.762	(0.576 - 1.008)	0.761	(0.553 - 1.048)
private	0.925	(0.682 - 1.254)	1.267	(0.865 - 1.858)	1.597***	(1.256 - 2.030)	0.846	(0.642 - 1.117)

Note: Elixhauser Groups drug abuse, peptic ulcer disease and AIDS/ HIV were not included into the regression due to a low case numbers

*** $p<0.001$

** $p<0.01$

* $p<0.05$

For peer review only

S11: Multivariate analysis of case-, care- and hospital-related covariates of patient safety including 25.805 colon cancer resections in 201 hospitals

	IN-HOSPITAL DEATH		POST-OPERATIVE RESPIRATORY FAILURE		RENAL FAILURE		POST-OPERATIVE WOUND INFECTION	
	OR	95% CI	OR	95% CI	OR	95% CI	OR	95% CI
**CASE COVARIATES**								
sex								
male	Ref.		Ref		Ref		Ref	
female	0.867**	(0.780 - 0.963)	0.810***	(0.747 - 0.878)	0.623***	(0.572 - 0.678)	0.879**	(0.807 - 0.959)
age	1.039***	(1.034 - 1.045)	1.008***	(1.005 - 1.012)	1.018***	(1.014 - 1.022)	0.998	(0.994 - 1.002)
alcohol abuse	1.448*	(1.087 - 1.928)	1.273	(0.982 - 1.650)	1.297*	(1.006 - 1.672)	1.222	(0.919 - 1.624)
blood loss anemia	0.552***	(0.405 - 0.752)	0.926	(0.743 - 1.153)	0.720**	(0.576 - 0.899)	1.199	(0.953 - 1.507)
cardiac arrhythmias	1.616***	(1.440 - 1.814)	1.560***	(1.422 - 1.712)	1.639***	(1.492 - 1.800)	1.229***	(1.108 - 1.363)
chronic pulmonary disease	1.329***	(1.142 - 1.546)	1.450***	(1.288 - 1.632)	1.059	(0.931 - 1.204)	1.213**	(1.064 - 1.384)
coagulopathy	4.057***	(3.617 - 4.550)	3.121***	(2.837 - 3.434)	3.066***	(2.780 - 3.383)	1.679***	(1.515 - 1.861)
congestive heart failure	1.766***	(1.539 - 2.026)	1.848***	(1.649 - 2.071)	1.629***	(1.453 - 1.827)	1.143*	(1.000 - 1.306)
deficiency anemia	0.687***	(0.550 - 0.858)	1.027	(0.880 - 1.198)	0.894	(0.756 - 1.057)	0.945	(0.793 - 1.126)
depression	0.760*	(0.610 - 0.946)	1.409***	(1.212 - 1.637)	1.053	(0.891 - 1.243)	1.557***	(1.336 - 1.813)
diabetes, complicated	1.213	(0.986 - 1.491)	0.969	(0.817 - 1.149)	1.351***	(1.150 - 1.587)	1.077	(0.891 - 1.302)
diabetes, uncomplicated	1.100	(0.965 - 1.253)	1.125*	(1.017 - 1.243)	1.090	(0.982 - 1.210)	1.074	(0.963 - 1.198)
fluid and electrolyte disorders	1.873***	(1.670 - 2.100)	2.338***	(2.145 - 2.547)	2.755***	(2.512 - 3.021)	2.038***	(1.857 - 2.236)
hypertension, complicated	0.511***	(0.409 - 0.639)	0.846	(0.711 - 1.008)	0.795*	(0.667 - 0.947)	0.941	(0.770 - 1.151)
hypertension, uncomplicated	0.624***	(0.559 - 0.698)	0.960	(0.881 - 1.047)	0.996	(0.909 - 1.090)	0.997	(0.909 - 1.094)
hypothyroidism	0.879	(0.752 - 1.028)	1.071	(0.955 - 1.201)	1.018	(0.900 - 1.151)	1.200**	(1.064 - 1.354)
liver disease	3.393***	(2.941 - 3.914)	1.375***	(1.202 - 1.571)	2.346***	(2.065 - 2.664)	0.906	(0.777 - 1.056)
lymphoma	0.816	(0.463 - 1.437)	0.766	(0.477 - 1.231)	1.366	(0.891 - 2.093)	1.283	(0.798 - 2.063)
metastatic cancer	1.414***	(1.273 - 1.571)	1.089*	(1.005 - 1.181)	0.986	(0.905 - 1.073)	1.053	(0.966 - 1.148)
obesity	0.745**	(0.621 - 0.894)	1.123	(0.994 - 1.269)	1.431***	(1.266 - 1.616)	1.513***	(1.337 - 1.713)
other neurological disorders	1.337**	(1.080 - 1.655)	1.818***	(1.532 - 2.157)	1.512***	(1.263 - 1.810)	1.214	(0.997 - 1.479)
paralysis	1.754***	(1.367 - 2.250)	1.402**	(1.135 - 1.733)	1.114	(0.889 - 1.395)	1.209	(0.951 - 1.537)
peripheral vascular disorders	1.644***	(1.420 - 1.904)	1.295***	(1.144 - 1.465)	1.290***	(1.138 - 1.462)	1.217**	(1.068 - 1.387)
psychoses	1.170	(0.607 - 2.253)	1.520	(0.948 - 2.437)	1.222	(0.731 - 2.045)	1.359	(0.820 - 2.252)
pulmonary circulation disorders	1.840***	(1.511 - 2.240)	1.977***	(1.675 - 2.333)	1.360***	(1.141 - 1.620)	1.088	(0.898 - 1.318)
renal failure	1.248***	(1.098 - 1.420)	1.162**	(1.047 - 1.289)	1.538***	(1.389 - 1.702)	1.143*	(1.018 - 1.282)
rheumatoid arthritis/collagen vascular diseases	0.883	(0.559 - 1.395)	0.647*	(0.441 - 0.949)	0.894	(0.626 - 1.277)	0.884	(0.599 - 1.305)
solid tumor without metastasis	0.329***	(0.212 - 0.511)	0.806	(0.520 - 1.252)	0.507**	(0.332 - 0.773)	1.526	(0.894 - 2.605)
valvular disease	0.671***	(0.548 - 0.821)	0.875	(0.747 - 1.024)	0.914	(0.781 - 1.069)	0.920	(0.769 - 1.100)
weight loss	1.007	(0.882 - 1.150)	1.586***	(1.434 - 1.753)	1.366***	(1.233 - 1.514)	1.583***	(1.421 - 1.763)
**HEALTHCARE COVARIATES**								
admission reason								
referral	Ref.		Ref.		Ref.		Ref.	
emergency case	1.822***	(1.613 - 2.058)	1.202***	(1.089 - 1.326)	1.407***	(1.266 - 1.564)	1.091	(0.983 - 1.211)
transfer from other hospital	2.491***	(1.962 - 3.161)	1.697***	(1.371 - 2.101)	1.556***	(1.247 - 1.941)	1.389**	(1.112 - 1.734)
weekend surgery								
no	Ref.		Ref.		Ref.		Ref.	
yes	1.949***	(1.650 - 2.303)	1.377***	(1.187 - 1.598)	1.578***	(1.365 - 1.824)	1.049	(0.885 - 1.244)

total colon resection								
no	Ref.		Ref.		Ref.		Ref.	
yes	2.046***	(1.669 - 2.509)	1.691***	(1.420 - 2.014)	1.703***	(1.422 - 2.038)	1.182	(0.986 - 1.417)
colon and rectum resection								
no	Ref.		Ref.		Ref.		Ref.	
yes	1.025	(0.853 - 1.232)	1.617***	(1.422 - 1.837)	1.394***	(1.214 - 1.600)	1.753***	(1.542 - 1.993)
**HOSPITAL COVARIATES**								
case volume	0.926	(0.810 - 1.058)	0.939	(0.795 - 1.110)	0.994	(0.865 - 1.142)	1.146	(0.975 - 1.348)
area								
urban	Ref.		Ref.		Ref.		Ref.	
rural	1.042	(0.857 - 1.267)	0.815	(0.605 - 1.099)	0.793*	(0.639 - 0.983)	0.964	(0.750 - 1.239)
university hospital								
no	Ref.		Ref.		Ref.		Ref.	
yes	1.301	(0.877 - 1.931)	0.650	(0.318 - 1.328)	1.280	(0.790 - 2.076)	2.209**	(1.281 - 3.807)
ownership								
public	Ref.		Ref.		Ref.		Ref.	
non-profit	1.146	(0.898 - 1.463)	1.098	(0.745 - 1.618)	0.876	(0.660 - 1.162)	0.767	(0.557 - 1.056)
private	1.207	(0.970 - 1.502)	1.316	(0.951 - 1.821)	1.993***	(1.572 - 2.526)	0.776	(0.591 - 1.018)

Note: Elixhauser Groups drug abuse, peptic ulcer disease and AIDS/ HIV were not included into the regression due to a low case numbers
*** p<0.001
** p <0.01
* p<0.05

For peer review only

S12: Multivariate analysis of case-, care- and hospital-related covariates of patient safety including 13.703 rectum cancer resections in 197 hospitals

	IN-HOSPITAL DEATH		POST-OPERATIVE RESPIRATORY FAILURE		RENAL FAILURE		POST-OPERATIVE WOUND INFECTION	
	OR	95% CI	OR	95% CI	OR	95% CI	OR	95% CI
**CASE COVARIATES**								
sex								
male	Ref.		Ref.		Ref.		Ref.	
female	0.912	(0.738 - 1.127)	0.892	(0.789 - 1.009)	0.685***	(0.599 - 0.783)	0.888	(0.789 - 1.000)
age	1.066***	(1.054 - 1.079)	1.013***	(1.007 - 1.019)	1.019***	(1.013 - 1.026)	0.998	(0.993 - 1.003)
alcohol abuse	1.807*	(1.034 - 3.156)	1.603*	(1.076 - 2.389)	1.156	(0.770 - 1.736)	1.138	(0.768 - 1.687)
blood loss anemia	0.823	(0.426 - 1.589)	0.677	(0.415 - 1.102)	1.231	(0.812 - 1.867)	1.466	(0.961 - 2.236)
cardiac arrhythmias	1.704***	(1.361 - 2.135)	1.714***	(1.484 - 1.981)	1.628***	(1.402 - 1.890)	1.050	(0.901 - 1.223)
chronic pulmonary disease	1.540**	(1.149 - 2.065)	1.473***	(1.218 - 1.781)	1.209	(0.986 - 1.482)	1.389***	(1.153 - 1.673)
coagulopathy	3.691***	(2.941 - 4.632)	2.783***	(2.396 - 3.233)	2.487***	(2.126 - 2.909)	1.723***	(1.493 - 1.990)
congestive heart failure	1.784***	(1.363 - 2.335)	1.798***	(1.488 - 2.172)	1.557***	(1.287 - 1.885)	1.028	(0.837 - 1.262)
deficiency anemia	1.008	(0.631 - 1.610)	1.404*	(1.049 - 1.879)	1.527**	(1.129 - 2.066)	1.269	(0.945 - 1.703)
depression	0.665	(0.418 - 1.058)	1.299*	(1.026 - 1.646)	1.359*	(1.060 - 1.742)	1.449***	(1.170 - 1.793)
diabetes, complicated	1.285	(0.830 - 1.990)	1.061	(0.798 - 1.409)	1.190	(0.906 - 1.562)	1.146	(0.860 - 1.528)
diabetes, uncomplicated	1.060	(0.817 - 1.375)	1.194*	(1.022 - 1.396)	1.075	(0.913 - 1.267)	1.243**	(1.067 - 1.448)
fluid and electrolyte disorders	1.693***	(1.353 - 2.117)	2.162***	(1.903 - 2.456)	2.720***	(2.367 - 3.126)	1.966***	(1.738 - 2.223)
hypertension, complicated	0.590*	(0.379 - 0.918)	0.844	(0.627 - 1.137)	0.844	(0.630 - 1.130)	0.894	(0.652 - 1.226)
hypertension, uncomplicated	0.625***	(0.502 - 0.780)	0.994	(0.873 - 1.132)	1.106	(0.962 - 1.272)	0.995	(0.877 - 1.129)
hypothyroidism	0.878	(0.633 - 1.217)	1.032	(0.860 - 1.239)	0.965	(0.791 - 1.177)	0.995	(0.832 - 1.191)
liver disease	3.342***	(2.461 - 4.538)	1.126	(0.879 - 1.443)	2.363***	(1.894 - 2.949)	1.171	(0.924 - 1.486)
lymphoma	1.440	(0.468 - 4.431)	1.006	(0.429 - 2.360)	1.225	(0.519 - 2.893)	1.918	(0.891 - 4.126)
metastatic cancer	1.280*	(1.035 - 1.581)	1.212**	(1.071 - 1.371)	1.015	(0.889 - 1.158)	1.041	(0.925 - 1.172)
obesity	0.953	(0.684 - 1.330)	1.476***	(1.241 - 1.755)	1.607***	(1.349 - 1.915)	1.570***	(1.333 - 1.849)
other neurological disorders	2.294***	(1.562 - 3.369)	2.146***	(1.634 - 2.818)	1.198	(0.885 - 1.621)	1.598**	(1.207 - 2.116)
paralysis	1.418	(0.866 - 2.321)	1.422*	(1.014 - 1.993)	1.249	(0.878 - 1.776)	1.130	(0.799 - 1.600)
peripheral vascular disorders	1.561**	(1.190 - 2.047)	1.151	(0.970 - 1.367)	1.259*	(1.055 - 1.503)	1.423***	(1.214 - 1.669)
psychoses	0.417	(0.045 - 3.841)	0.477	(0.147 - 1.541)	1.904	(0.756 - 4.792)	1.012	(0.413 - 2.481)
pulmonary circulation disorders	3.547***	(2.491 - 5.049)	2.146***	(1.633 - 2.819)	1.567**	(1.179 - 2.082)	1.234	(0.923 - 1.650)
renal failure	0.942	(0.721 - 1.231)	1.125	(0.949 - 1.334)	1.750***	(1.489 - 2.056)	1.233*	(1.039 - 1.462)
rheumatoid arthritis/collagen vascular diseases	1.041	(0.443 - 2.445)	0.825	(0.462 - 1.476)	0.962	(0.544 - 1.701)	0.600	(0.312 - 1.154)
solid tumor without metastasis	0.105***	(0.039 - 0.282)	0.960	(0.353 - 2.613)	1.074	(0.327 - 3.534)	1.648	(0.542 - 5.013)
valvular disease	0.808	(0.550 - 1.185)	1.099	(0.855 - 1.413)	0.922	(0.712 - 1.193)	0.969	(0.743 - 1.265)
weight loss	0.967	(0.745 - 1.255)	1.761***	(1.517 - 2.045)	1.333***	(1.142 - 1.558)	1.557***	(1.345 - 1.803)
**HEALTHCARE COVARIATES**								
admission reason								
referral	Ref.		Ref.		Ref.		Ref.	
emergency case	1.937***	(1.526 - 2.460)	1.199*	(1.018 - 1.413)	1.257*	(1.055 - 1.498)	1.468***	(1.255 - 1.718)
transfer from other hospital	2.503***	(1.487 - 4.213)	2.160***	(1.497 - 3.118)	1.731**	(1.180 - 2.540)	1.805**	(1.263 - 2.578)
weekend surgery								
no	Ref.		Ref.		Ref.		Ref.	
yes	2.137***	(1.417 - 3.224)	1.384*	(1.024 - 1.870)	1.267	(0.940 - 1.708)	1.148	(0.845 - 1.560)

colon and rectum resection								
no	Ref.		Ref.		Ref.		Ref.	
yes	2.422***	(1.940 - 3.024)	2.085***	(1.806 - 2.408)	1.711***	(1.470 - 1.991)	1.627***	(1.415 - 1.871)
**HOSPITAL COVARIATES**								
case volume	0.678***	(0.577 - 0.796)	0.907	(0.764 - 1.075)	0.835**	(0.730 - 0.955)	0.988	(0.851 - 1.147)
area								
urban	Ref.		Ref.		Ref.		Ref.	
rural	1.164	(0.862 - 1.572)	0.951	(0.661 - 1.369)	0.808	(0.632 - 1.033)	0.843	(0.638 - 1.113)
university hospital								
no	Ref.		Ref.		Ref.		Ref.	
yes	1.512	(0.889 - 2.571)	0.807	(0.355 - 1.837)	1.374	(0.840 - 2.246)	2.195**	(1.267 - 3.801)
ownership								
public	Ref.		Ref.		Ref.		Ref.	
non-profit	1.034	(0.725 - 1.473)	1.089	(0.682 - 1.740)	0.797	(0.580 - 1.095)	0.733	(0.518 - 1.037)
private	0.900	(0.635 - 1.276)	1.238	(0.828 - 1.850)	1.807***	(1.375 - 2.376)	0.853	(0.629 - 1.158)

Note: Elixhauser Groups drug abuse, peptic ulcer disease and AIDS/ HIV were not included into the regression due to a low case numbers

*** $p<0.001$

** $p<0.01$

* $p<0.05$

For peer review only

S13: Multivariate analysis of case-, care- and hospital-related covariates of patient safety including 28.363 non-cancer colon resections in 208 hospitals

	IN-HOSPITAL DEATH		POST-OPERATIVE RESPIRATORY FAILURE		RENAL FAILURE		POST-OPERATIVE WOUND INFECTION	
	OR	95% CI	OR	95% CI	OR	95% CI	OR	95% CI
**CASE COVARIATES**								
sex								
male	Ref.		Ref		Ref		Ref	
female	0.988	(0.896 - 1.090)	0.746***	(0.691 - 0.807)	0.748***	(0.689 - 0.811)	0.872***	(0.804 - 0.946)
age	1.056***	(1.051 - 1.060)	1.016***	(1.014 - 1.019)	1.026***	(1.023 - 1.030)	0.997	(0.995 - 1.000)
alcohol abuse	1.483***	(1.190 - 1.847)	2.400***	(1.985 - 2.902)	1.745***	(1.435 - 2.121)	1.350**	(1.099 - 1.657)
blood loss anemia	0.388***	(0.246 - 0.612)	1.296	(0.945 - 1.777)	1.140	(0.831 - 1.565)	1.150	(0.828 - 1.597)
cardiac arrhythmias	1.666***	(1.499 - 1.851)	1.627***	(1.487 - 1.781)	1.605***	(1.464 - 1.760)	1.166**	(1.053 - 1.291)
chronic pulmonary disease	1.405***	(1.231 - 1.603)	1.463***	(1.310 - 1.633)	1.154*	(1.028 - 1.296)	1.264***	(1.123 - 1.422)
coagulopathy	4.166***	(3.747 - 4.632)	2.920***	(2.665 - 3.198)	3.515***	(3.209 - 3.851)	1.602***	(1.451 - 1.769)
congestive heart failure	1.764***	(1.562 - 1.993)	1.972***	(1.770 - 2.197)	1.733***	(1.555 - 1.932)	1.282***	(1.135 - 1.448)
deficiency anemia	0.943	(0.732 - 1.214)	1.219	(0.995 - 1.493)	1.428***	(1.166 - 1.749)	1.188	(0.967 - 1.459)
depression	0.582***	(0.472 - 0.717)	1.488***	(1.288 - 1.719)	1.071	(0.918 - 1.249)	1.564***	(1.354 - 1.807)
diabetes, complicated	1.332**	(1.105 - 1.606)	1.430***	(1.211 - 1.688)	1.187*	(1.005 - 1.404)	1.326**	(1.104 - 1.593)
diabetes, uncomplicated	1.170*	(1.026 - 1.334)	1.304***	(1.172 - 1.450)	1.226***	(1.098 - 1.368)	1.172**	(1.046 - 1.314)
fluid and electrolyte disorders	1.828***	(1.641 - 2.037)	2.715***	(2.495 - 2.955)	2.930***	(2.681 - 3.202)	1.929***	(1.764 - 2.110)
hypertension, complicated	0.527***	(0.430 - 0.645)	0.680***	(0.570 - 0.812)	0.847	(0.712 - 1.008)	0.965	(0.794 - 1.172)
hypertension, uncomplicated	0.489***	(0.440 - 0.543)	0.933	(0.858 - 1.015)	0.861***	(0.789 - 0.940)	1.107*	(1.011 - 1.210)
hypothyroidism	0.729***	(0.631 - 0.842)	1.152*	(1.034 - 1.283)	1.064	(0.949 - 1.192)	1.210***	(1.081 - 1.354)
liver disease	5.708***	(4.967 - 6.559)	1.408***	(1.238 - 1.601)	2.827***	(2.491 - 3.207)	0.802**	(0.694 - 0.925)
lymphoma	1.016	(0.683 - 1.513)	0.858	(0.602 - 1.223)	0.895	(0.624 - 1.284)	0.978	(0.668 - 1.433)
metastatic cancer	-		-		-		-	
obesity	0.768***	(0.657 - 0.897)	1.180**	(1.055 - 1.321)	1.557***	(1.390 - 1.745)	1.737***	(1.557 - 1.938)
other neurological disorders	1.612***	(1.360 - 1.912)	1.949***	(1.686 - 2.253)	1.498***	(1.289 - 1.740)	1.313***	(1.123 - 1.536)
paralysis	1.284*	(1.039 - 1.587)	1.482***	(1.237 - 1.777)	1.124	(0.930 - 1.358)	1.238*	(1.021 - 1.500)
peripheral vascular disorders	2.129***	(1.886 - 2.405)	1.626***	(1.458 - 1.813)	1.675***	(1.500 - 1.871)	1.266***	(1.125 - 1.426)
psychoses	0.518*	(0.279 - 0.963)	2.462***	(1.639 - 3.698)	0.660	(0.405 - 1.074)	1.516	(0.994 - 2.313)
pulmonary circulation disorders	1.348**	(1.094 - 1.662)	1.737***	(1.445 - 2.088)	1.296**	(1.070 - 1.570)	1.040	(0.849 - 1.274)
renal failure	1.381***	(1.228 - 1.553)	1.073	(0.969 - 1.189)	1.494***	(1.351 - 1.653)	0.996	(0.889 - 1.117)
rheumatoid arthritis/collagen vascular diseases	1.046	(0.791 - 1.384)	1.120	(0.885 - 1.416)	0.981	(0.770 - 1.250)	1.143	(0.893 - 1.462)
solid tumor without metastasis	-		-		-		-	
valvular disease	0.954	(0.803 - 1.133)	0.861	(0.738 - 1.004)	0.944	(0.810 - 1.102)	0.942	(0.795 - 1.116)
weight loss	0.799**	(0.696 - 0.917)	1.717***	(1.543 - 1.910)	1.520***	(1.365 - 1.693)	2.013***	(1.803 - 2.248)
**HEALTHCARE COVARIATES**								
admission reason								
referral	Ref.		Ref.		Ref.		Ref.	
emergency case	1.936***	(1.706 - 2.197)	1.727***	(1.564 - 1.906)	1.479***	(1.330 - 1.645)	1.197***	(1.086 - 1.320)
transfer from other hospital	2.610***	(2.169 - 3.140)	2.299***	(1.959 - 2.698)	2.027***	(1.718 - 2.391)	1.202*	(1.015 - 1.423)
weekend surgery								
no	Ref.		Ref.		Ref.		Ref.	
yes	1.577***	(1.397 - 1.780)	1.436***	(1.296 - 1.590)	1.443***	(1.299 - 1.604)	1.095	(0.978 - 1.225)

total colon resection								
no	Ref.		Ref.		Ref.		Ref.	
yes	3.173***	(2.705 - 3.723)	1.604***	(1.395 - 1.843)	2.545***	(2.213 - 2.927)	0.958	(0.830 - 1.107)
colon and rectum resection								
no	Ref.		Ref.		Ref.		Ref.	
yes	1.159	(0.936 - 1.436)	1.320**	(1.116 - 1.562)	1.404***	(1.183 - 1.667)	1.314**	(1.107 - 1.560)
**HOSPITAL COVARIATES**								
case volume	0.961	(0.844 - 1.094)	0.876	(0.753 - 1.018)	0.950	(0.841 - 1.073)	1.135	(0.987 - 1.304)
area								
urban	Ref.		Ref.		Ref.		Ref.	
rural	1.091	(0.886 - 1.345)	0.918	(0.682 - 1.235)	0.775*	(0.631 - 0.953)	1.058	(0.837 - 1.336)
university hospital								
no	Ref.		Ref.		Ref.		Ref.	
yes	1.336	(0.855 - 2.087)	0.712	(0.347 - 1.461)	1.586	(0.999 - 2.520)	1.802*	(1.070 - 3.034)
ownership								
public	Ref.		Ref.		Ref.		Ref.	
non-profit	0.900	(0.688 - 1.177)	1.028	(0.696 - 1.519)	0.836	(0.638 - 1.096)	0.736*	(0.543 - 0.996)
private	1.284*	(1.015 - 1.626)	1.433*	(1.035 - 1.986)	1.893***	(1.505 - 2.380)	0.775	(0.601 - 1.001)

Note: Elixhauser Groups drug abuse, peptic ulcer disease and AIDS/ HIV were not included into the regression due to a low case numbers

*** p<0.001

** p <0.01

* p<0.05

For peer review only

S14: Multivariate analysis of case-, care- and hospital-related covariates of patient safety including 6.692 non-cancer rectum resections in 195 hospitals

	IN-HOSPITAL DEATH		POST-OPERATIVE RESPIRATORY FAILURE		RENAL FAILURE		POST-OPERATIVE WOUND INFECTION	
	OR	95% CI	OR	95% CI	OR	95% CI	OR	95% CI
**CASE COVARIATES**								
sex								
male	Ref.		Ref		Ref		Ref	
female	0.733*	(0.542 - 0.992)	0.739**	(0.601 - 0.908)	0.746**	(0.607 - 0.917)	0.651***	(0.539 - 0.786)
age	1.074***	(1.058 - 1.089)	1.013***	(1.006 - 1.021)	1.023***	(1.016 - 1.031)	1.000	(0.994 - 1.006)
alcohol abuse	0.809	(0.348 - 1.882)	1.801*	(1.008 - 3.219)	1.355	(0.754 - 2.434)	1.080	(0.601 - 1.941)
blood loss anemia	0.797	(0.305 - 2.082)	1.735	(0.877 - 3.432)	1.886	(0.970 - 3.668)	1.927*	(1.014 - 3.663)
cardiac arrhythmias	2.130***	(1.569 - 2.892)	1.573***	(1.243 - 1.990)	1.608***	(1.281 - 2.017)	1.015	(0.795 - 1.295)
chronic pulmonary disease	0.908	(0.601 - 1.371)	1.570**	(1.180 - 2.088)	0.977	(0.726 - 1.315)	1.402*	(1.066 - 1.845)
coagulopathy	5.767***	(4.191 - 7.937)	3.713***	(2.934 - 4.697)	3.703***	(2.949 - 4.650)	1.947***	(1.550 - 2.447)
congestive heart failure	1.851***	(1.309 - 2.617)	1.887***	(1.426 - 2.497)	1.543**	(1.180 - 2.017)	1.269	(0.943 - 1.708)
deficiency anemia	0.765	(0.362 - 1.616)	1.008	(0.606 - 1.675)	1.039	(0.640 - 1.689)	1.430	(0.911 - 2.244)
depression	0.591	(0.326 - 1.073)	1.043	(0.723 - 1.505)	1.220	(0.859 - 1.732)	1.208	(0.869 - 1.679)
diabetes, complicated	2.158**	(1.267 - 3.674)	0.990	(0.627 - 1.564)	2.155***	(1.423 - 3.265)	1.292	(0.821 - 2.032)
diabetes, uncomplicated	1.233	(0.838 - 1.815)	1.133	(0.849 - 1.513)	1.549**	(1.176 - 2.038)	0.950	(0.709 - 1.272)
fluid and electrolyte disorders	1.619**	(1.185 - 2.213)	3.048***	(2.458 - 3.779)	3.006***	(2.430 - 3.719)	1.944***	(1.594 - 2.370)
hypertension, complicated	0.345***	(0.189 - 0.628)	0.657	(0.419 - 1.031)	0.910	(0.596 - 1.390)	1.042	(0.663 - 1.638)
hypertension, uncomplicated	0.511***	(0.377 - 0.694)	0.928	(0.751 - 1.147)	0.813	(0.658 - 1.003)	1.041	(0.851 - 1.273)
hypothyroidism	0.742	(0.491 - 1.121)	0.919	(0.695 - 1.214)	1.051	(0.803 - 1.376)	1.093	(0.845 - 1.416)
liver disease	5.966***	(3.793 - 9.384)	1.550*	(1.068 - 2.252)	2.060***	(1.451 - 2.925)	0.795	(0.540 - 1.172)
lymphoma	0.685	(0.151 - 3.108)	0.930	(0.309 - 2.800)	0.656	(0.221 - 1.944)	0.884	(0.308 - 2.539)
metastatic cancer	-		-		-		-	
obesity	0.853	(0.538 - 1.352)	1.534**	(1.152 - 2.043)	1.400*	(1.054 - 1.861)	1.871***	(1.460 - 2.396)
other neurological disorders	2.558***	(1.586 - 4.126)	1.428	(0.968 - 2.106)	1.117	(0.755 - 1.652)	1.113	(0.754 - 1.642)
paralysis	0.798	(0.421 - 1.514)	1.276	(0.780 - 2.088)	1.508	(0.940 - 2.421)	1.158	(0.714 - 1.881)
peripheral vascular disorders	2.505***	(1.743 - 3.599)	1.502**	(1.119 - 2.018)	1.629***	(1.227 - 2.163)	1.048	(0.774 - 1.419)
psychoses	1.703	(0.166 - 17.483)	1.385	(0.317 - 6.052)	3.384	(0.925 - 12.382)	1.386	(0.359 - 5.356)
pulmonary circulation disorders	1.770	(0.965 - 3.246)	1.636	(0.983 - 2.725)	1.114	(0.667 - 1.859)	1.203	(0.725 - 1.997)
renal failure	1.472*	(1.047 - 2.068)	1.633***	(1.252 - 2.129)	1.755***	(1.368 - 2.250)	1.395*	(1.071 - 1.818)
rheumatoid arthritis/collagen vascular diseases	0.864	(0.358 - 2.087)	1.948*	(1.171 - 3.242)	1.083	(0.609 - 1.925)	1.001	(0.571 - 1.754)
solid tumor without metastasis	-		-		-		-	
valvular disease	1.236	(0.771 - 1.982)	0.999	(0.671 - 1.488)	1.300	(0.888 - 1.903)	0.719	(0.469 - 1.104)
weight loss	0.761	(0.507 - 1.143)	1.969***	(1.503 - 2.580)	1.944***	(1.504 - 2.513)	2.204***	(1.709 - 2.843)
**HEALTHCARE COVARIATES**								
admission reason								
referral	Ref.		Ref.		Ref.		Ref.	
emergency case	2.060***	(1.470 - 2.885)	1.590***	(1.253 - 2.018)	1.382**	(1.094 - 1.746)	0.992	(0.797 - 1.234)
transfer from other hospital	2.583***	(1.488 - 4.487)	1.510	(0.962 - 2.369)	1.949**	(1.269 - 2.993)	1.102	(0.713 - 1.704)
weekend surgery								
no	Ref.		Ref.		Ref.		Ref.	
yes	1.770**	(1.179 - 2.658)	1.391*	(1.012 - 1.912)	1.499*	(1.101 - 2.042)	0.870	(0.613 - 1.235)

colon and rectum resection								
no	Ref.		Ref.		Ref.		Ref.	
yes	2.967***	(2.184 - 4.030)	2.183***	(1.750 - 2.725)	2.190***	(1.771 - 2.709)	1.318*	(1.066 - 1.630)
**HOSPITAL COVARIATES**								
case volume	0.777*	(0.614 - 0.983)	0.793*	(0.642 - 0.978)	0.863	(0.733 - 1.015)	0.956	(0.795 - 1.150)
area								
urban	Ref.		Ref.		Ref.		Ref.	
rural	0.851	(0.536 - 1.351)	0.881	(0.580 - 1.337)	0.872	(0.646 - 1.176)	0.865	(0.613 - 1.220)
university hospital								
no	Ref.		Ref.		Ref.		Ref.	
yes	1.529	(0.712 - 3.283)	0.849	(0.354 - 2.034)	1.176	(0.701 - 1.972)	2.375**	(1.316 - 4.287)
ownership								
public	Ref.		Ref.		Ref.		Ref.	
non-profit	0.556*	(0.323 - 0.959)	1.098	(0.652 - 1.849)	0.702	(0.489 - 1.008)	0.775	(0.516 - 1.166)
private	0.981	(0.594 - 1.621)	1.614*	(1.020 - 2.556)	1.331	(0.960 - 1.846)	0.791	(0.546 - 1.147)

Note: Elixhauser Groups drug abuse, peptic ulcer disease and AIDS/ HIV were not included into the regression due to a low case numbers
*** p<0.001
** p <0.01
* p<0.05

For peer review only

S15 – Figure: Interactions between emergency admission and university hospital status

For peer review only

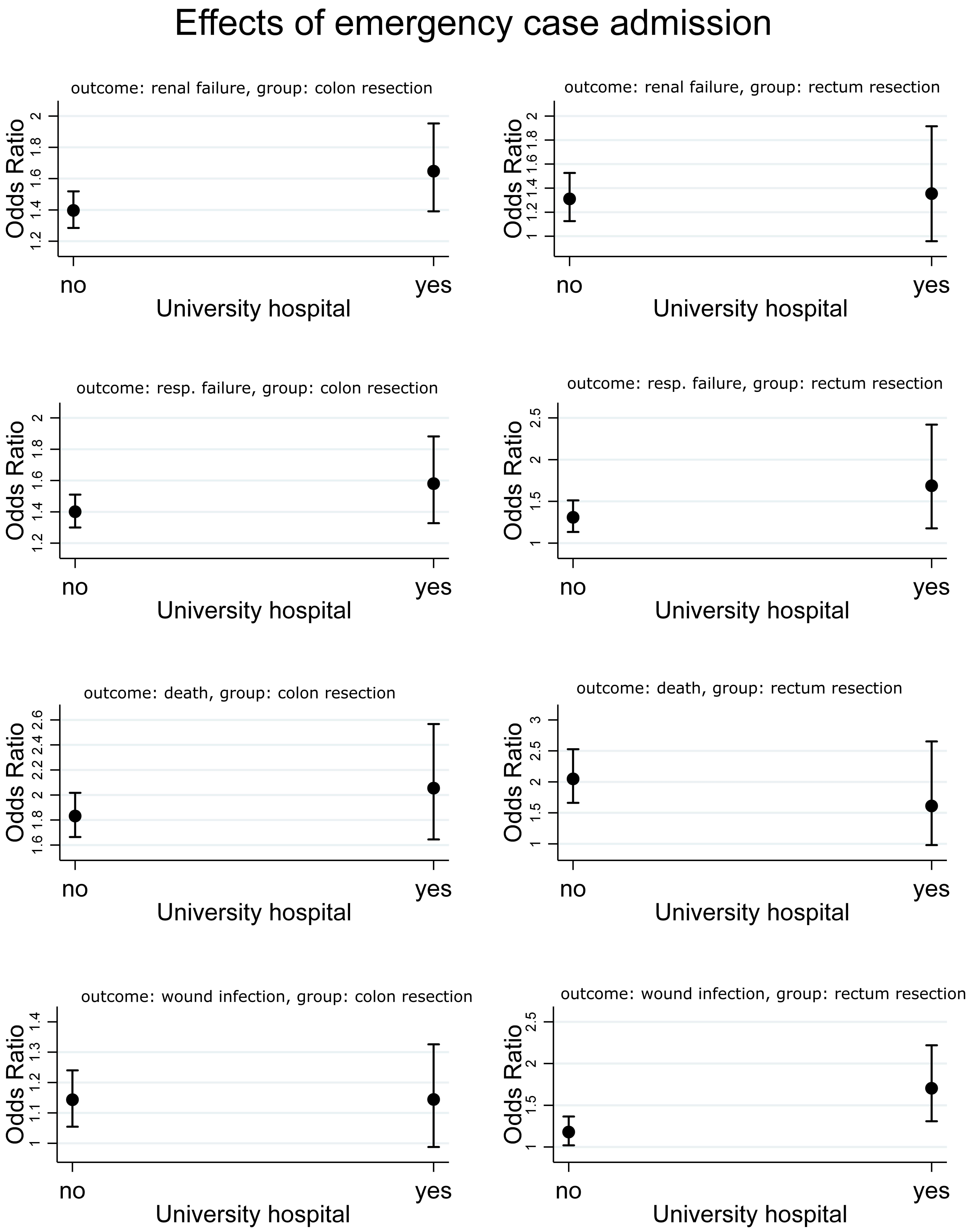
Effects of emergency case admission
outcome: renal failure, group: colon resection
outcome: renal failure, group: rectum resection
outcome: resp. failure, group: colon resection
outcome: resp. failure, group: rectum resection
outcome: death, group: colon resection
outcome: death, group: rectum resection
outcome: wound infection, group: colon resection
outcome: wound infection, group: rectum resection
Odds Ratio
University hospital
no
yes

S16 – Figure: Interactions between transfer from other hospital admission and university hospital status

For peer review only

Effects of transfer from other hospitals

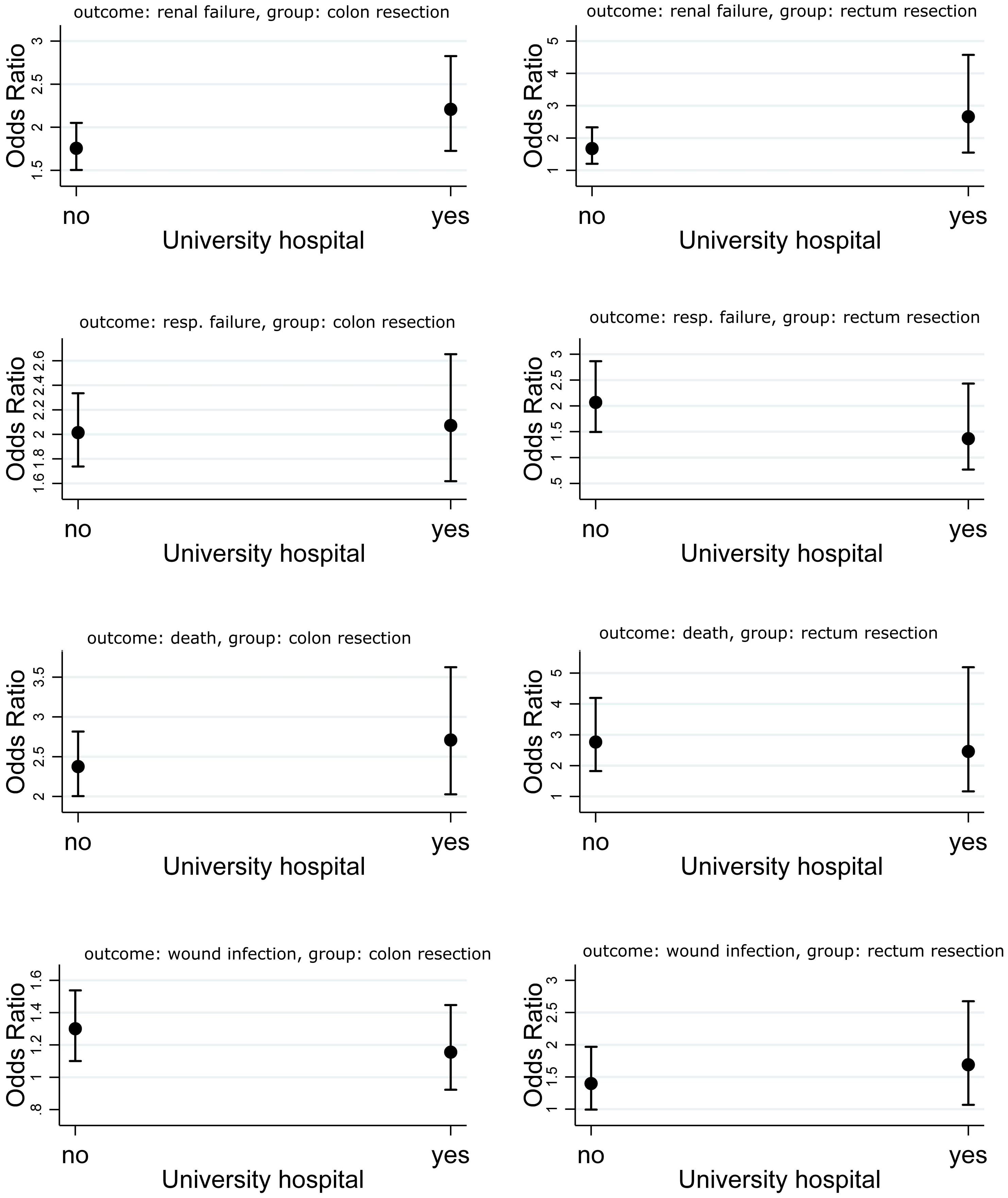

Reporting checklist for cross sectional study.

Based on the STROBE cross sectional guidelines.

Instructions to authors

Complete this checklist by entering the page numbers from your manuscript where readers will find each of the items listed below.

Your article may not currently address all the items on the checklist. Please modify your text to include the missing information. If you are certain that an item does not apply, please write "n/a" and provide a short explanation.

Upload your completed checklist as an extra file when you submit to a journal.

In your methods section, say that you used the STROBE cross sectionalreporting guidelines, and cite them as:

von Elm E, Altman DG, Egger M, Pocock SJ, Gotzsche PC, Vandenbroucke JP. The Strengthening the Reporting of Observational Studies in Epidemiology (STROBE) Statement: guidelines for reporting observational studies.

		Reporting Item	Page Number
**Title and abstract**			
Title	#1a	Indicate the study’s design with a commonly used term in the title or the abstract	1
Abstract	#1b	Provide in the abstract an informative and balanced summary of what was done and what was found	2
**Introduction**			
Background / rationale	#2	Explain the scientific background and rationale for the investigation being reported	4
Objectives	#3	State specific objectives, including any prespecified hypotheses	4
**Methods**			
Study design	#4	Present key elements of study design early in the paper	4
Setting	#5	Describe the setting, locations, and relevant dates, including periods of recruitment, exposure, follow-up, and data collection	5
Eligibility criteria	#6a	Give the eligibility criteria, and the sources and methods of selection of participants.	4-6
	#7	Clearly define all outcomes, exposures, predictors, potential confounders, and effect modifiers. Give diagnostic criteria, if applicable	5-6
Data sources / measurement	#8	For each variable of interest give sources of data and details of methods of assessment (measurement). Describe comparability of assessment methods	5

For peer review only

		if there is more than one group. Give information separately for for exposed and unexposed groups if applicable.	
Bias	#9	Describe any efforts to address potential sources of bias	6-7
Study size	#10	Explain how the study size was arrived at	5-6
Quantitative variables	#11	Explain how quantitative variables were handled in the analyses. If applicable, describe which groupings were chosen, and why	6-7
Statistical methods	#12a	Describe all statistical methods, including those used to control for confounding	6-7
Statistical methods	#12b	Describe any methods used to examine subgroups and interactions	6-7
Statistical methods	#12c	Explain how missing data were addressed	N/A
Statistical methods	#12d	If applicable, describe analytical methods taking account of sampling strategy	N/A
Statistical methods	#12e	Describe any sensitivity analyses	7
**Results**			
Participants	#13a	Report numbers of individuals at each stage of study—eg numbers potentially eligible, examined for eligibility, confirmed eligible, included in the study, completing follow-up, and analysed. Give information separately for for exposed and unexposed groups if applicable.	7
Participants	#13b	Give reasons for non-participation at each stage	N/A
Participants	#13c	Consider use of a flow diagram	7/ Fig 1
Descriptive data	#14a	Give characteristics of study participants (eg demographic, clinical, social) and information on exposures and potential confounders. Give information separately for exposed and unexposed groups if applicable.	7-8, Table 1
Descriptive data	#14b	Indicate number of participants with missing data for each variable of interest	N/A
Outcome data	#15	Report numbers of outcome events or summary measures. Give information separately for exposed and unexposed groups if applicable.	7-8 / Fig 1, Table 1
Main results	#16a	Give unadjusted estimates and, if applicable, confounder-adjusted estimates and their precision (eg, 95% confidence interval). Make clear which confounders were adjusted for and why they were included	10-11 / Table 2-3, Supp. 7-16)
Main results	#16b	Report category boundaries when continuous variables were categorized	N/A
Main results	#16c	If relevant, consider translating estimates of relative risk into absolute risk for a meaningful time period	N/A

For peer review only

Other analyses	#17	Report other analyses done—e.g., analyses of subgroups and interactions, and sensitivity analyses	11
**Discussion**			
Key results	#18	Summarise key results with reference to study objectives	14
Limitations	#19	Discuss limitations of the study, taking into account sources of potential bias or imprecision. Discuss both direction and magnitude of any potential bias.	15
Interpretation	#20	Give a cautious overall interpretation considering objectives, limitations, multiplicity of analyses, results from similar studies, and other relevant evidence.	14-15
Generalisability	#21	Discuss the generalisability (external validity) of the study results	14-15
**Other Information**			
Funding	#22	Give the source of funding and the role of the funders for the present study and, if applicable, for the original study on which the present article is based	17

Notes:

- 15: 7-8 / Fig 1
- 16a: 10-11 / Table 2-3, Supp. 7-16) The STROBE checklist is distributed under the terms of the Creative Commons Attribution License CC-BY. This checklist was completed on 15. October 2021 using https://www.goodreports.org/, a tool made by the EQUATOR Network in collaboration with Penelope.ai

For peer review only
